# Genomic analysis of sewage from 101 countries reveals global landscape of antimicrobial resistance

**DOI:** 10.1038/s41467-022-34312-7

**Published:** 2022-12-01

**Authors:** Patrick Munk, Christian Brinch, Frederik Duus Møller, Thomas N. Petersen, Rene S. Hendriksen, Anne Mette Seyfarth, Jette S. Kjeldgaard, Christina Aaby Svendsen, Bram van Bunnik, Fanny Berglund, Global Sewage Surveillance Consortium, Artan Bego, Pablo Power, Catherine Rees, Dionisia Lambrinidis, Elizabeth Heather Jakobsen Neilson, Karen Gibb, Kris Coventry, Peter Collignon, Susan Cassar, Franz Allerberger, Anowara Begum, Zenat Zebin Hossain, Carlon Worrell, Olivier Vandenberg, Ilse Pieters, Dougnon Tamègnon Victorien, Angela Daniela Salazar Gutierrez, Freddy Soria, Vesna Rudić Grujić, Nataša Mazalica, Teddie O. Rahube, Carlos Alberto Tagliati, Dalia Rodrigues, Guilherme Oliveira, Larissa Camila Ribeiro de Souza, Ivan Ivanov, Bonkoungou Isidore Juste, Traoré Oumar, Thet Sopheak, Yith Vuthy, Antoinette Ngandjio, Ariane Nzouankeu, Ziem A. Abah Jacques Olivier, Christopher K. Yost, Pratik Kumar, Satinder Kaur Brar, Djim-Adjim Tabo, Aiko D. Adell, Esteban Paredes-Osses, Maria Cristina Martinez, Sara Cuadros-Orellana, Changwen Ke, Huanying Zheng, Li Baisheng, Lok Ting Lau, Teresa Chung, Xiaoyang Jiao, Yongjie Yu, Zhao JiaYong, Johan F. Bernal Morales, Maria Fernanda Valencia, Pilar Donado-Godoy, Kalpy Julien Coulibaly, Jasna Hrenovic, Matijana Jergović, Renáta Karpíšková, Zozo Nyarukweba Deogratias, Bodil Elsborg, Lisbeth Truelstrup Hansen, Pernille Erland Jensen, Mohamed Abouelnaga, Mohamed Fathy Salem, Marliin Koolmeister, Mengistu Legesse, Tadesse Eguale, Annamari Heikinheimo, Soizick Le Guyader, Julien Schaeffer, Jose Eduardo Villacis, Bakary Sanneh, Lile Malania, Andreas Nitsche, Annika Brinkmann, Sara Schubert, Sina Hesse, Thomas U. Berendonk, Courage Kosi Setsoafia Saba, Jibril Mohammed, Patrick Kwame Feglo, Regina Ama Banu, Charalampos Kotzamanidis, Efthymios Lytras, Sergio A. Lickes, Bela Kocsis, Norbert Solymosi, Thorunn R. Thorsteinsdottir, Abdulla Mohamed Hatha, Mamatha Ballal, Sohan Rodney Bangera, Fereshteh Fani, Masoud Alebouyeh, Dearbhaile Morris, Louise O’Connor, Martin Cormican, Jacob Moran-Gilad, Antonio Battisti, Elena Lavinia Diaconu, Gianluca Corno, Andrea Di Cesare, Patricia Alba, Junzo Hisatsune, Liansheng Yu, Makoto Kuroda, Motoyuki Sugai, Shizuo Kayama, Zeinegul Shakenova, Ciira Kiiyukia, Eric Ng’eno, Lul Raka, Kazi Jamil, Saja Adel Fakhraldeen, Tareq Alaati, Aivars Bērziņš, Jeļena Avsejenko, Kristina Kokina, Madara Streikisa, Vadims Bartkevics, Ghassan M. Matar, Ziad Daoud, Asta Pereckienė, Ceslova Butrimaite-Ambrozeviciene, Christian Penny, Alexandra Bastaraud, Tiavina Rasolofoarison, Jean-Marc Collard, Luc Hervé Samison, Mala Rakoto Andrianarivelo, Daniel Lawadi Banda, Arshana Amin, Heraa Rajandas, Sivachandran Parimannan, David Spiteri, Malcolm Vella Haber, Sunita J. Santchurn, Aleksandar Vujacic, Dijana Djurovic, Brahim Bouchrif, Bouchra Karraouan, Delfino Carlos Vubil, Pushkar Pal, Heike Schmitt, Mark van Passel, Gert-Jan Jeunen, Neil Gemmell, Stephen T. Chambers, Fania Perez Mendoza, Jorge Huete-Pιrez, Samuel Vilchez, Akeem Olayiwola Ahmed, Ibrahim Raufu Adisa, Ismail Ayoade Odetokun, Kayode Fashae, Anne-Marie Sørgaard, Astrid Louise Wester, Pia Ryrfors, Rune Holmstad, Mashkoor Mohsin, Rumina Hasan, Sadia Shakoor, Natalie Weiler Gustafson, Claudia Huber Schill, Maria Luz Zamudio Rojas, Jorge Echevarria Velasquez, Bonifacio B. Magtibay, Kris Catangcatang, Ruby Sibulo, Felipe Campos Yauce, Dariusz Wasyl, Celia Manaia, Jaqueline Rocha, Jose Martins, Pedro Álvaro, Doris Di Yoong Wen, Hanseob Shin, Hor-Gil Hur, Sukhwan Yoon, Golubinka Bosevska, Mihail Kochubovski, Radu Cojocaru, Olga Burduniuc, Pei-Ying Hong, Meghan Rose Perry, Amy Gassama, Vladimir Radosavljevic, Moon Y. F. Tay, Rogelio Zuniga-Montanez, Stefan Wuertz, Dagmar Gavačová, Katarína Pastuchová, Peter Truska, Marija Trkov, Karen Keddy, Kerneels Esterhuyse, Min Joon Song, Marcos Quintela-Baluja, Mariano Gomez Lopez, Marta Cerdà-Cuéllar, R. R. D. P. Perera, N. K. B. K. R. G. W. Bandara, H. I. Premasiri, Sujatha Pathirage, Kareem Charlemagne, Carolin Rutgersson, Leif Norrgren, Stefan Örn, Renate Boss, Tanja Van der Heijden, Yu-Ping Hong, Happiness Houka Kumburu, Robinson Hammerthon Mdegela, Yaovi Mahuton Gildas Hounmanou, Kaknokrat Chonsin, Orasa Suthienkul, Visanu Thamlikitkul, Ana Maria de Roda Husman, Bawimodom Bidjada, Berthe-Marie Njanpop-Lafourcade, Somtinda Christelle Nikiema-Pessinaba, Belkis Levent, Cemil Kurekci, Francis Ejobi, John Bosco Kalule, Jens Thomsen, Ouidiane Obaidi, Laila Mohamed Jassim, Andrew Moore, Anne Leonard, David W. Graham, Joshua T. Bunce, Lihong Zhang, William H. Gaze, Brett Lefor, Drew Capone, Emanuele Sozzi, Joe Brown, John Scott Meschke, Mark D. Sobsey, Michael Davis, Nicola Koren Beck, Pardi Sukapanpatharam, Phuong Truong, Ronald Lilienthal, Sanghoon Kang, Thomas E. Wittum, Natalia Rigamonti, Patricia Baklayan, Chinh Dang Van, Doan Minh Nguyen Tran, Nguyen Do Phuc, Geoffrey Kwenda, D. G. Joakim Larsson, Marion Koopmans, Mark Woolhouse, Frank M. Aarestrup

**Affiliations:** 1 Research Group for Genomic Epidemiology, Technical University of Denmark, Kgs, Lyngby, Denmark; 2 Centre for Immunity, Infection and Evolution, University of Edinburgh, Edinburgh, UK; 3 Centre for Antibiotic Resistance Research (CARe), University of Gothenburg, Gothenburg, Sweden; 4 Department of Viroscience, Erasmus MC, Rotterdam, The Netherlands; 5 Institute of Public Health, Tirana, Albania; 6 Universidad de Buenos Aires, Buenos Aires, Argentina; 7 Melbourne Water Corporation, Melbourne, Australia; 8 Charles Darwin University, Darwin, Australia; 9 University of Copenhagen, Frederiksberg C, Denmark; 10 Charles Darwin University, Darwin Northern Territory, Australia; 11 Canberra Hospital, Canberra, Australia; 12 ALS Water, Scoresby, Australia; 13 Austrian Agency for Health and Food Safety (AGES), Vienna, Austria; 14 University of Dhaka, Dhaka, Bangladesh; 15 Environmental Protection Department, Bridgetown, St. Michael, Barbados; 16 Laboratoire Hospitalier Universitaire de Bruxelles (LHUB-ULB), Brussels, Belgium; 17 AQUAFIN NV, Aartselaar, Belgium; 18 Polytechnic School of Abomey-Calavi, Abomey-Calavi, Benin; 19 Universidad Catσlica Boliviana San Pablo, La Paz, Bolivia; 20 Public Health Institute of the Republic of Srpska, Faculty of Medicine University of Banja Luka, Banja Luka, Bosnia and Herzegovina; 21 Public Health Institute of the Republic of Srpska, Banja Luka, Bosnia and Herzegovina; 22 Botswana International University of Science and Technology, Palapye, Botswana; 23 Universidade Federal de Minas Gerais, Belo Horizonte, Brazil; 24 Oswaldo Cruz Institute, Rio de Janeiro, Brazil; 25 Vale Institute of Technology, Belιm, PA Brazil; 26 National Center of Infectious and Parasitic Diseases, Sofia, Bulgaria; 27 University of Ouagadougou, Ouagadougou, Burkina Faso; 28 Institut Pasteur du Cambodge, Phnom Penh, Cambodia; 29 Centre Pasteur du Cameroun, Yaoundι, Cameroon; 30 University of Regina, Regina, Canada; 31 Eau Terre Environnement Research Centre (INRS-ETE), Quebec City G1K 9A9, Canada and Indian Institute of Technology, Jammu, India; 32 Eau Terre Environnement Research Centre (INRS-ETE), Quebec City G1K 9A9, Canada and Lassonde School of Enginerring, York University, Toronto, Canada; 33 University of N’Djamena, N’Djamena, Chad; 34 Escuela de Medicina Veterinaria, Facultad de Ciencias de la Vida, Universidad Andres Bello, Santiago, Chile; 35 Institute of Public Health, Santiago, Chile; 36 Universidad Catolica del Maule, Centro de Biotecnología de los Recursos Naturales, Facultad de Ciencias Agrarias y Forestales, Talca, Chile; 37 Guangdong Provincial Center for Disease Control and Prevention, Guangzhou, China; 38 The Hong Kong Polytechnic University, Hong Kong, China; 39 Shantou University Medical College, Shantou, China; 40 Nanjing University of Information Science and Technology, Nanjing, China; 41 Center for Disease Control and Prevention of Henan province, Zhengzhou, China; 42 Colombian Integrated Program for Antimicrobial Resistance Surveillance - Coipars, CI Tibaitatα, Corporaciσn Colombiana de Investigaciσn Agropecuaria (AGROSAVIA), Tibaitatα - Mosquera, Cundinamarca Colombia; 43 Institut Pasteur de Côte d’Ivoire, Abidjan, Côte d’Ivoire; 44 University of Zagreb, Zagreb, Croatia; 45 Andrija Stampar Teaching Institute of Public Health, Zagreb, Croatia; 46 Veterinary Research Institute, Brno, Czech Republic; 47 Centre de Recherche en Sciences Naturelles de Lwiro (CRSN-LWIRO), Bukavu, Democratic Republic of Congo; 48 BIOFOS A/S, Copenhagen K, Denmark; 49 Technical University of Denmark, Kgs., Lyngby, Denmark; 50 Suez Canal University, Ismailia, Egypt; 51 University of Sadat City, Sadat City, Egypt; 52 Ministry of Health, Environmental Microbiology, Tallinn, Estonia; 53 Addis Ababa University, Addis Ababa, Ethiopia; 54 University of Helsinki, Helsinki, Finland; 55 French Institute Search Pour L’exploitation De La Mer (Ifremer), Nantes, France; 56 Instituto Nacional de Investigaciσn en Salud Pϊblica-INSPI (CRNRAM), Galαpagos, Quito, Ecuador; 57 National Public Health Laboratories, Ministry of Health and Social Welfare, Kotu, Gambia; 58 National Center for Disease Control and Public Health, Tbilisi, Georgia; 59 Robert Koch Institute, Berlin, Germany; 60 Technische Universitδt Dresden, Institute of Hydrobiology, Dresden, Germany; 61 University for Development Studies, Tamale, Ghana; 62 University of Ghana, Accra, Ghana; 63 Kwame Nkrumah University of Science and Technology, Kumasi, PMB Ghana; 64 Council for Scientific and Industrial Research Water Research Institute, Accra, Ghana; 65 Veterinary Research Institute of Thessaloniki, Hellenic Agricultural Organisation-DEMETER, Thermi, Greece; 66 Athens Water Supply and Sewerage Company (EYDAP S.A.), Athens, Greece; 67 Universidad de San Carlos de Guatemala, Guatemala City, Guatemala; 68 Semmelweis University, Institute of Medical Microbiology, Budapest, Hungary; 69 University of Veterinary Medicine, Budapest, Hungary; 70 University of Iceland, Reykjavνk, Iceland; 71 Cochin University of Science and Technology, Cochin, India; 72 Kasturba Medical College, Manipal, India; 73 Apollo Diagnostics, Mangalore, India; 74 Shiraz University of Medical Sciences, Shiraz, Iran; 75 Shahid Beheshti University of Medical Sciences, Tehran, Iran; 76 National University of Ireland Galway, Galway, Ireland; 77 Ben Gurion University of the Negev and Ministry of Health, Beer-Sheva, Israel; 78 Istituto Zooprofilattico Sperimentale del Lazio e della Toscana, Rome, Italy; 79 CNR - Water Research Institute, Verbania, Italy; 80 National Institute of Infectious Diseases, Tokyo, Japan; 81 National Center of Expertise, Taldykorgan, Kazakhstan; 82 Mount Kenya University, Thika, Kenya; 83 Kenya Medical Research Institute, Nairobi, Kenya; 84 University of Prishtina “Hasan Prishtina” & National Institute of Public Health of Kosovo, Pristina, Kosovo; 85 Kuwait Institute for Scientific Research, Kuwait City, Kuwait; 86 Institute of Food Safety, Riga, Latvia; 87 American University of Beirut, Beirut, Lebanon; 88 Central Michigan University & Michigan Health Clinics, Saginaw, MI USA; 89 National Food and Veterinary Risk Assessment Institute, Vilnius, Lithuania; 90 Luxembourg Institute of Science and Technology, Belvaux, Luxembourg; 91 Institut Pasteur de Madagascar, Antananarivo, Madagascar; 92 University of Antananarivo, Centre d’Infectiologie Charles Mιrieux, Antananarivo, Madagascar; 93 University of Malawi, Blantyre, Malawi; 94 Malaysian Genomics Resource Centre Berhad, Kuala Lumpur, Malaysia; 95 AIMST University, COMBio, Kedah, Malaysia; 96 Water Services Corporation, Luqa, Malta; 97 Environmental Health Directorate, St. Venera, Malta; 98 University of Mauritius, Reduit, Mauritius; 99 Institute for Public Health Montenegro, Podgorica, Montenegro; 100 Institut Pasteur du Maroc, Casablanca, Morocco; 101 Centro de Investigaηγo em Saϊde de Manhiηa (CISM), Maputo, Mozambique; 102 Agriculture and Forestry University, Kathmandu, Nepal; 103 National Institute for Public, Health and the Environment (RIVM), Bilthoven, The Netherlands; 104 University of Otago, Dunedin, New Zealand; 105 University of Otago, Christchurch, New Zealand; 106 University of Central America, Managua, Nicaragua; 107 Universidad Nacional Autσnoma de Nicaragua-Leσn, Leσn, Nicaragua; 108 University of Ilorin, Ilorin, Nigeria; 109 University of Ibadan, Ibadan, Nigeria; 110 Norwegian Institute of Public Health, Oslo, Norway; 111 VEAS, Slemmestad, Norway; 112 University of Agriculture, Faisalabad, Pakistan; 113 Aga Khan University, Karachi, Pakistan; 114 Laboratorio Central de Salud Publica, Asuncion, Paraguay; 115 Instituto Nacional de Salud, Lima, Peru; 116 Universidad de Piura, Piura, Peru; 117 WHO Environmental and Occupational Health, Manila, Philippines; 118 Maynilad Water Services, Inc., Quezon City, Philippines; 119 National Veterinary Research Institute, Pulawy, Poland; 120 Universidade Catσlica Portuguesa, CBQF - Centro de Biotecnologia e Quνmica Fina - Laboratσrio Associado, Escola Superior de Biotecnologia, Porto, Portugal; 121 Aguas do Tejo Atlantico, Lisboa, Portugal; 122 Gwangju Institute of Science and Technology, Gwangju, Republic of Korea; 123 Korea Advanced Institute of Science and Technology, Daejeon, Republic of Korea; 124 Institute of Public Health of the Republic of North Macedonia, Skopje, Republic of North Macedonia; 125 State Medical and Pharmaceutical University, Chișinău, Republic of Moldova; 126 National Agency for Public Health, Chișinău, Republic of Moldova; 127 King Abdullah University of Science and Technology, Thuwal, Saudi Arabia; 128 University of Edinburgh, Edinburgh, Scotland UK; 129 Institut Pasteur de Dakar, Dakar, Senegal; 130 Institute of Veterinary Medicine of Serbia, Belgrade, Serbia; 131 Nanyang Technological University, Singapore, Singapore; 132 Public Health Authority of the Slovak Republic, Bratislava, Slovakia; 133 National Laboratory of Health, Environment and Food, Ljubljana, Slovenia; 134 Independent consultant, Johannesburg, South Africa; 135 Daspoort Waste Water Treatment Works, Pretoria, South Africa; 136 Korea Advanced Institute of Science and Technology, Daejeon, South Korea; 137 School of Veterinary Sciences, Lugo, Spain; 138 Labaqua, Santiago de Compostela, Spain; 139 IRTA, Centre de Recerca en Sanitat Animal (CReSA, IRTA-UAB), Campus de la Universitat Autonoma de Barcelona, Bellaterra, Spain; 140 University of Kelaniya, Ragama, Sri Lanka; 141 Medical Research Institute, Colombo, Sri Lanka; 142 Caribbean Public Health Agency, Catries, Saint Lucia; 143 The Sahlgrenska Academy at the University of Gothenburg, Gothenburg, Sweden; 144 Swedish University of Agricultural Sciences, Uppsala, Sweden; 145 Federal Food Safety and Veterinary Office (FSVO), Bern, Switzerland; 146 Ara Region Bern AG, Herrenschwanden, Switzerland; 147 Centers for Disease Control, Taipei, Taiwan; 148 Kilimanjaro Clinical Research Institute, Moshi, Tanzania; 149 Sokoine University of Agriculture, Morogoro, Tanzania; 150 Faculty of Science and Technology, Suratthani Rajabhat University, Surat Thani, Thailand; 151 Faculty of Public Health, Mahidol University, Bangkok, Thailand; 152 Faculty of Medicine Siriraj Hospital, Bangkok, Thailand; 153 National Institute for Public Health and the Environment (RIVM), Bilthoven, Netherlands; 154 National Institute of Hygiene, Lomι, Togo; 155 Agence de Mιdecine Prιventive, Dapaong, Togo; 156 Division of Integrated Surveillance of Health Emergencies and Response, Lomι, Togo; 157 Public Health Institution of Turkey, Ankara, Turkey; 158 Hatay Mustafa Kemal University, Hatay, Turkey; 159 Makerere University, Kampala, Uganda; 160 Abu Dhabi Public Health Center, Abu Dhai, United Arab Emirates; 161 Dubai municipality, WWTP Al Aweer, Dubai, UAE; 162 Rashid Hospital, Dubai, UAE; 163 Northumbrian Water, Northumbria House, Abbey Road, Pity Me, Durham, UK; 164 University of Exeter Medical School, Cornwall, UK; 165 Newcastle University, Newcastle upon Tyne, UK; 166 Brightwater Treatment Plant, Woodinville, WA USA; 167 Department of Environmental Sciences and Engineering, University of North Carolina at Chapel Hill, Chapel Hill, NC USA; 168 University of North Carolina, Chapel Hill, USA; 169 University of Washington, Seattle, WA USA; 170 Baylor University, Waco, USA; 171 Columbia Boulevard WWTP, Portland, USA; 172 Eastern Illinois University, Charleston, USA; 173 The Ohio State University, Columbus Ohio, USA; 174 Laboratorio Tecnolσgico del Uruguay, Montevideo, Uruguay; 175 Institute of Public Health in Ho Chi Minh City, Ho Chi Minh, Vietnam; 176 University of Zambia, Lusaka, Zambia

**Keywords:** Antimicrobial resistance, Microbial ecology, Metagenomics, Policy and public health in microbiology

## Abstract

Antimicrobial resistance (AMR) is a major threat to global health. Understanding the emergence, evolution, and transmission of individual antibiotic resistance genes (ARGs) is essential to develop sustainable strategies combatting this threat. Here, we use metagenomic sequencing to analyse ARGs in 757 sewage samples from 243 cities in 101 countries, collected from 2016 to 2019. We find regional patterns in resistomes, and these differ between subsets corresponding to drug classes and are partly driven by taxonomic variation. The genetic environments of 49 common ARGs are highly diverse, with most common ARGs carried by multiple distinct genomic contexts globally and sometimes on plasmids. Analysis of flanking sequence revealed ARG-specific patterns of dispersal limitation and global transmission. Our data furthermore suggest certain geographies are more prone to transmission events and should receive additional attention.

## Abstract (web summary)

Understanding the emergence, evolution, and transmission of antibiotic resistance genes (ARGs) is essential to combat antimicrobial resistance. Here, Munk et al. analyse ARGs in hundreds of sewage samples from 101 countries and describe regional patterns, diverse genetic environments of common ARGs, and ARG-specific transmission patterns.

## Introduction

The need for global genomic pathogen surveillance and understanding their global ecology, epidemiology and evolution is now larger than ever. Most recently, this was exemplified by the COVID-19 pandemic, which is the most devastating respiratory disease outbreak since the 1918 Spanish flu^1^. Rapid sequencing and sharing of genomic data has enabled researchers from around the world to study the evolution and spread of SARS-CoV-2 variants^2–4^.

Genomic monitoring should not be reserved for acute pandemics. It should be applied continuously also to endemic infections and silent epidemics, including antimicrobial resistance (AMR), which grows progressively worse and by some is predicted to result in an annual death toll of 10 million by 2050^5^. A large, recent study found that bacterial AMR caused an additional 1.27 million deaths in 2019 in the world, compared to a model scenario where all infections were susceptible to treatment^6^. Understanding the epidemiology underlying the global and local emergence, selection and transmission of AMR, and the individual antibiotic resistance genes (ARGs), is essential to develop sustainable strategies combatting this threat.

We and others have previously proposed sewage as a convenient, ethical AMR monitoring matrix and used it to determine the worldwide diversity and abundance of ARGs using metagenomics^7,8^. Exactly because sewage has the possibility to monitor both humans, their animals, and their immediate environments, it offers a good cost-effective matrix to survey entire cities for the ARGs fluctuating. Indeed, it was recently shown that all the major clinical SARS-CoV-2 variants can be found in sewage, but so can variants underrepresented in clinical samples^9^. This could indicate the presence of alternative reservoirs that are missed by hospital surveillance, further highlighting the usefulness of sewage surveillance. Globally, more than two billion people still lack clean water with faeces/sewage being the most common contaminant. Having a single connected ecosphere means sewage offers a simple way to survey both the ARG variants that we carry and those we may soon clinically encounter.

There are several recent examples of ARGs that have emerged clinically and swept globally through the environment, and human and animal populations, including the New Delhi metallo-beta-lactamase gene (*bla*_NDM_), *bla*_CTX-M_, and variants of the mobile colistin resistance gene *mcr*^10–12^. Several examples of global transmission of specific antimicrobial resistant clinical strains have also been documented, such as the global transmission of *Escherichia coli* ST131, *Salmonella enterica* serovar Typhi H-58 and separate MRSA lineages^13–15^. These rapid, global transmission events support the Baas Becking hypothesis stating everything is everywhere, but the environment selects^16^. It therefore appears that drug usage-based selection, to a higher degree than dispersal opportunities, has been the primary cause for the successful spread of ARGs. The effects of drug-based selection on AMR will then be further amplified by increased transmission, which is linked to educational, economic and infrastructural factors among others^7,17^.

The acceptance of the Baas Becking hypothesis has fostered the general belief that ARGs can emerge virtually anywhere in the world and be selected for given antimicrobial usage. This implies that, with enhanced surveillance, early emergence events could be detected prior to global spread. However, local predominance of specific, mainly recently emerged ARGs within pathogenic bacterial strains in individual countries and/or regions have been observed. An example is found in clinical carbapenemase-producing*Enterobacteriaceae* (CPE) isolates, where *bla*_OXA-48_ is the dominant gene in certain countries, especially European, the *bla*_KPC_ is most frequent in the USA and *bla*_NDM_ alleles have become the dominant in India^18–21^. For cephalosporinases, similar patterns are observed with FOX primarily being isolated in the USA, whereas MOX and CMY are associated with Asia and European livestock. This has been attributed to local selection or spread of specific community- or nosocomial-derived clones but may also reflect a recent emergence that have not yet transmitted globally, perhaps due to competition from phenotypically similar proteins. While such geographically separated regions of transmission in culturable pathogens may suggest future risk of global transmission, comparatively little is known about ARG transmission in complex microbiomes. We have previously observed ARGs with apparent regional restrictions, which could not be attributed to antimicrobial usage but potentially to host distribution or dispersal limitations^7^.

Recent studies are confirming that the microbiome composition is mainly shaped by biotic and abiotic factors and not by geography directly^22,23^. This is also seen in livestock, where strict diets are easier to enforce and known to influence both the resulting microbiome and resistome^24,25^. A limited number of microbial studies have however, contested this universality theory and suggested dispersal limitations based on observations of distance-decay relationships^26–28^.

Surveillance-solutions based on shotgun metagenomic sequencing have the advantage of being relatively hypothesis-agnostic. While AMR, is itself an extremely important monitoring target, the possibility for additional monitoring of e.g., emergent infectious disease agents, give the method significant additional value compared to more targeted approaches. A clear example of this, is the recent use of the Global Sewage metagenomics data for monitoring of known human pathogenic viruses^29^. We therefore hope this unique genomic dataset will also prove valuable to future monitoring efforts.

In our previous study, we found that sample resistome profiles cluster significantly according to geography. This finding was later expanded upon with the observation that some bacterial species show global phylogeographic signatures^30^.

In this study, we aim to provide a comprehensive sewage-based overview of global ARG abundance, diversity, and genomic backgrounds. We analyze and present comparable resistomes from most countries on Earth, greatly expanding the global geographical coverage. Using flanking sequences around assembled ARGs, we wanted to develop a better strategy for tracking the global transmission patterns of individual ARGs, allowing better-resolved epidemiological insights. Flank-based analyses has the potential to both quantify slow SNP accumulation and dramatic context changes, potentially giving better resolution than analysing individual ARGs.

The dataset contains many ARG variants not in current databases, variable gene syntenies, and global distribution patterns highlighting the spatial and genomic components of AMR. For many urban environments, cities and even some countries, there was a complete lack of microbiome and resistome data which we here seek to rectify. Our analyses also reveal the problem of the historic under-sampling of LMIC countries in current AMR monitoring efforts and suggest that fighting AMR will need more locally tailored solutions.

## Results

### Summary of the dataset and reads

A total of 757 urban sewage samples were collected from 243 cities in 101 countries covering 7 major geographical regions (See Supplementary Data 1). Paired-end sequencing reads from all the metagenomes were searched against known ARGs and the small ribosomal subunit genes, common to all life. The average number of sequencing fragments per sample was 45.9 million (range: 4.1–187.3 M, SD: 15.4 M), yielding a total of 34.7 billion sequencing fragments spanning more than 4 × 10^12^ nucleotides. An average of 0.05% of the reads were assigned to ARGs. Across all datasets, 0.3% of the reads were assigned to 16/18 S SSU rRNA, and of these, 96.8% and 2.9% were mapped to bacteria and eukaryotes, respectively. Among sequencing fragments assigned to vertebrates, species of fishes, primates, rodents, sheep, poultry, and pig were the most abundant. Even though the 18 S rRNA gene is not optimal for eukaryote species assignment, the composition appeared consistent with humans, food animals and some common sewer inhabitants.

Rarefaction of the reads mapping to bacterial Silva fragments showed some samples starting to saturate in terms of found genera, but there still being much uncovered diversity (Supplementary Fig. 1).

The average number of bacterial genera detected in samples was 969 (range: 419–1690. SD: 166). The genera with most assigned sequencing fragments include *Streptococcus*, *Acinetobacter*, *Klebsiella*, *Pseudomonas* and *Acidovorax*. Among the top twenty most common genera, were also others with members of pathogenic potential such as *Staphylococcus*, *Enterococcus* and *Neisseria*.

Across the included samples, we found evidence of 557 different ARGs. Thirteen of the ARGs were universal and found in every single sample and include *mph*(E), *msr*(E)*, tet*(A)*, tet*(C), *tet*(W)*, sul1* and *sul2*. A total of 127 ARGs were detected in at least half of the samples. Sequencing depth seemed appropriate as many samples had plateaued in rarefaction analyses and additional ResFinder hits resulted only few extra ARGs (Supplementary Fig. 2).

Resistome alpha-diversity was highly variable between samples, with high intra-region dispersion (Supplementary Fig. 3). Higher bacterial genus diversity was associated with higher ARG diversity (Spearman’s rho: 0.45, *p* = 2.2^−16^), but higher estimated genus richness was not associated with ARG richness (Spearman’s rho: 0.044, *p* = 0.28) (Supplementary Fig. 4).

### Resistomes are geographically stable in time

**Fig. 1:**
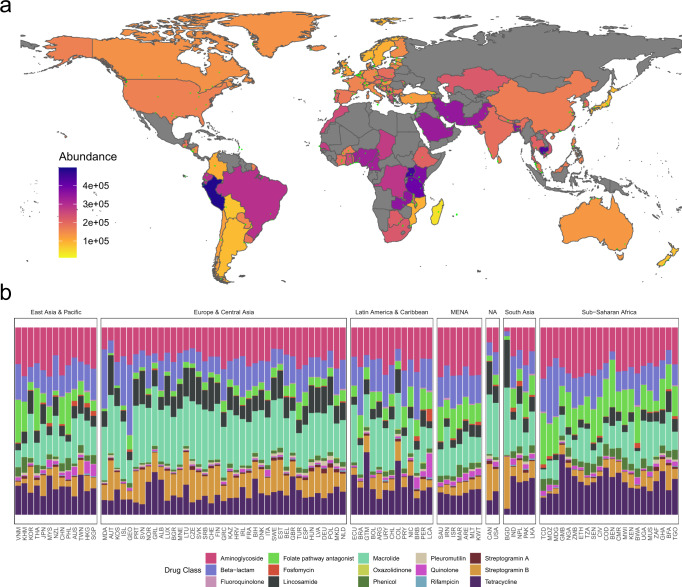
The global resistome based on sewage-based monitoring. **a** Choropleth of the world coloured by the country-wise average total AMR load (see methods). Small green dots show unique sampling sites contributing to the average. Some areas are disputed, and we realize that exact border placement is difficult due to geopolitical issues. **b** Stacked bar chart of relative abundances per drug class per country. Each panel represent countries in a World Bank region and is ordered by the Shannon diversity of class-level AMR.

The ARG abundances varied across sites and continents. The highest total ARG loads were on average observed in Sub-Saharan Africa, which also includes The Gambia and Madagascar that interestingly had the lowest ARG loads (Fig. 1a). There was generally a strong association between measured total ARG loads and the results of our previous predictive model based on our 2016 samples alone (Spearman’s rho: 0.56, *p* < 0.01, Supplementary Fig. 5)^7^. A few interesting observations, however, stand out i.e., the previously mentioned Gambia and Madagascar were among largest outliers, with very low relative AMR loads, while other Sub-Saharan African countries like Uganda and Zambia had higher ARG loads than anticipated. Europe and North America appeared very homogenous in terms of total ARG load, which could be expected due to their similarity in World Bank Data that the model was built on. Africa, The Middle East, and South America on the other hand, contained both very high and very low values, with pronounced contrasts between neighbouring countries in more variable regions.

Even though we did not receive samples from all the same sites in each round, for 46 countries, there were samples from 2016, 2017, and 2018. Samples from 2017 and 2018 appeared particularly similar, whereas, in 2016, values were generally higher in Africa and South America (Supplementary Fig. 6). It is worth noting that we averaged abundances over each year and 2016 contained only one major sampling round, as opposed to both winter and summer rounds in 2017 and 2018, which could be influential.

### Resistomes reflect world geography but differ by drug class

**Fig. 2:**
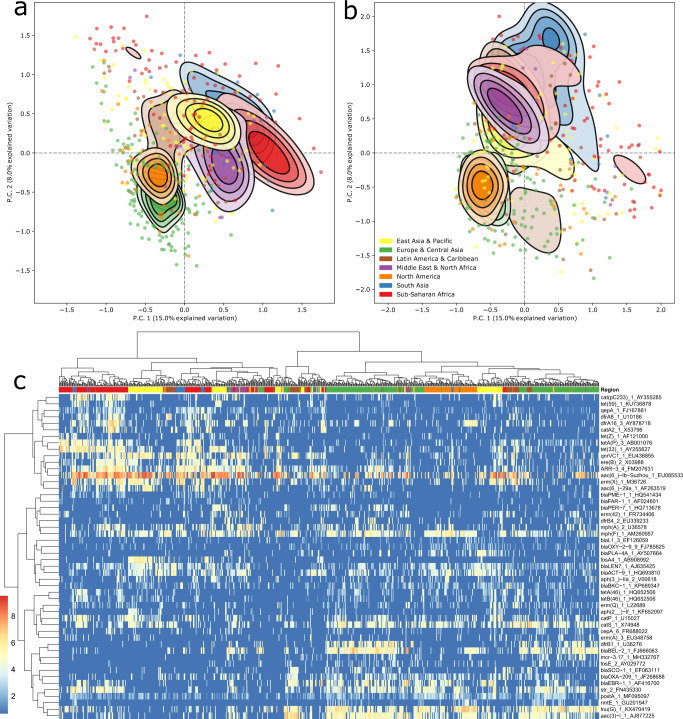
World regional effects of both bacteriomes and resistomes. **a** Resistance gene (90% homology grouping) PCA clustering. **b** Bacterial genus PCA clustering. The principal components in both panels are calculated from centered log-ratio (CLR) transformed size-adjusted counts. The contours show the group-wise sample density and are truncated at 50% of the peak value. Samples outside this range are drawn as individual points. **c** Clustered resistome heatmap showing the additive log ratio (ALR) abundance of the 50 most variable ARGs in the dataset. Hierarchical clustering of sample columns is based on all ARGs, not just the visualized ones. The darkest blue indicates no assigned reads in sample-ARG combination.

A hierarchical clustering of the sample resistomes, resulted in relatively even two-split of the samples, with Europe, central Asia, and North America heavily overrepresented in the right-hand cluster (Fig. 2c). A heatmap of the most variable genes shows which of them specifically are driving the two-split and further sub-clustering to regions and even individual countries. For example, *bla*_BEL-2_ was prevalent in European and North American samples in the right-hand cluster. This cephalosporinase is normally associated with *P. aeruginosa*. Despite this, *Pseudomonas* had similar relative abundances in the affected regions and the rest of the world.

The left cluster was associated with African and Middle Eastern samples. Macrolide resistance genes such as *erm(*B)*, mef*(A)*, msr*(D)*, msr*(E) *and mph*(E) were more abundant in the right-hand cluster, whereas the sulfonamide resistance gene *sul1* had higher load in the left cluster (Supplementary Fig. 7).

A total of 16.2% resistome beta-diversity was explained by overall regional grouping (permanova, *p* = 0.001, Fig. 2a). For the bacteriome (assessed on genus level), a similar amount (11.9% of the variance) was explained by regionality (permanova, *p* = 0.001, Fig. 2b). Seasonality (Q4 + Q1 vs Q2 + Q3) explained only 0.2% beta-diversity, which was non-significant (permanova, *p* = 0.051). When correcting for hemisphere effects and changing season for samples with a negative latitude, there was a significant, but only modest 1.8% explanatory effect (permanova, *p* = 0.001).

Bacterial composition seems to split the world’s region in roughly two major groups, whereas the resistomes have more unique profiles and have slightly more separate density peaks. Using Procrustes analyses, we found the bacteriome and resistomes were closely associated (0.871, *p* = 0.001, *n* = 757).

**Fig. 3:**
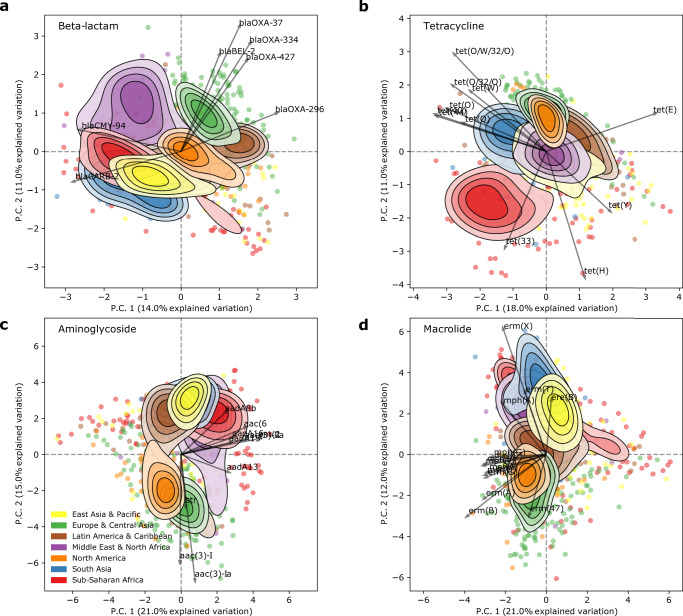
Resistome clustering differs by drug class. PCA of the resistome were carried out individually on subsets of ARGs providing resistance to different drug classes. The contours are defined in Fig. 2. Arrows show the ARGs with the highest variance in each drug class. **a** Beta-lactam. **b** Tetracycline. **c** Aminoglycoside. **d** Macrolide. Plots for the remaining drug classes are shown in Supplementary Fig. 8.

Geographical separations were also observed when analysing the resistome at class-level, but importantly the patterns and separations varied between drug classes (Fig. 3 and Supplementary Fig. 8). Some regions were often, but not always, co-spatial, e.g., Europe and North America, South Asia, and East Asia, and sometimes regions split into two distinct clusters, e.g., Latin America for Beta-lactams. Sub-Saharan Africa was frequently distinct from North Africa and the Middle East. Interestingly, for tetracyclines the ARGs separating the regions were clearly grouped into those encoding efflux pumps and those encoding 16 S rRNA methylases.

### Most common sewage ARGs are sometimes carried by plasmids

In the metagenomic assemblies, we found 55,391 ARGs. Across 5814 instances, 336 different ARGs were flanked with at least 1 Kb flanks both up- and down-stream. The metagenomic scaffolds with flanks were classified to bacteria in 92% of cases. 7% of the contigs could not be assigned a superkingdom, and the remaining (<0.4%) were on contigs assigned to either Archaea, Viruses (e.g., *Klebsiella* phages) or Eukaryota (e.g., humans).

However, since many ARGs are known to associate with plasmids, taxonomic scaffold assignment might not reflect the bacterial host background in which they are currently sitting. To shed light on the scope of ARG-plasmid connection in sewage, we used a combination of three techniques to determine which contigs were plasmidic and what their association to the ARGs were. See Supplementary Info for more details on this result, but in summary 2102 of the contigs encoding 134 unique ARGs were identified as plasmidic, suggesting a large proportion of ARGs are sometimes carried by plasmids in our samples (Supplementary Data 2, Supplementary Fig. 9). Interestingly, plasmid assignment frequency was much higher (41% vs 20%) among ARGs observed on ten or more contigs (Pearson’s Chi-squared test, *n* = 5814, *p* = 4.3 × 10^−16^), which is evidence of just how important plasmids are in global transmission.

Very few ARGs, like *bla*DHA-1 and *qnr*S1 always appeared to be plasmid-associated, while others like *lnu*(D) and *cat*Q were never associated with plasmids, regardless of geography. However, most common ARGs had mixed backgrounds with some instances of plasmid carriage (Supplementary Fig. 10).

ARGs in major geographical regions had similar frequency of plasmid assignment (39%), varying between 35% and 44% per region, but within-regions there were still major differences between countries (Supplementary Fig. 11). Sub-Saharan Africa had both the highest and lowest values, from 55% in DR Congo to 26% in Burkina Faso and Tanzania.

### ARGs have wide host ranges in certain regions

When selecting just non-plasmid scaffolds with at least 1 kb up- and down-stream flank, 2994 Kraken-assigned ARGs contigs remained for further analysis. These were almost exclusively from *Proteobacteria* and *Firmicutes*, *Bacteroidetes* and *Actinobacteria*.

The intersection of non-plasmid ARGs with three or more assigned hosts and genera with three or more assigned ARGs can be seen in Supplementary Fig. 12. *Escherichia* was especially prominent in thus subset, carrying genes often shared with other taxa.

**Fig. 4:**
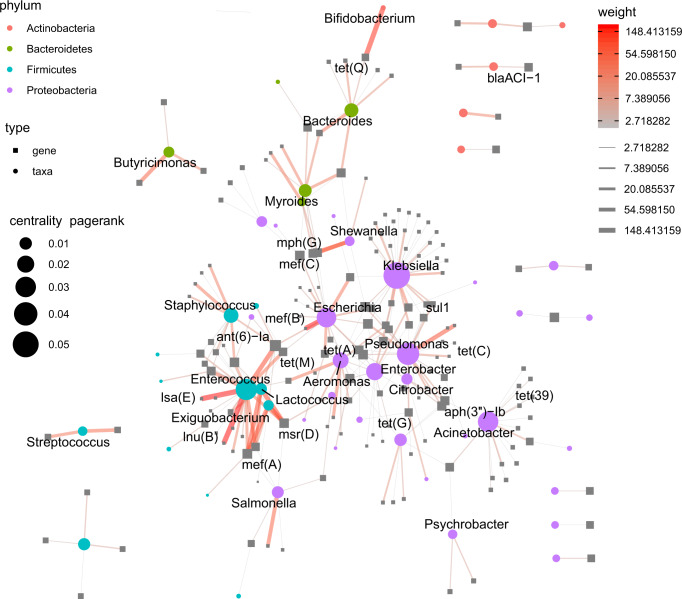
Gene-sharing network between bacterial genera. Edges link ARGs to the genera which their contigs were taxonomically assigned to. Only flanked, non-plasmidic contigs were used. The backbone algorithm was used to compute the graph layout. Color and thickness of edges denote the number of observed taxa-gene co-occurrences. Nodes are ARGs and genera which are visualized as grey boxes and colored circles respectively. Node size denote the centrality of the individual nodes to the overall network. Smaller subgraphs were manually moved for space efficiency, so relative distances between those mean nothing.

A network graph of genus-ARG co-occurrence revealed a major separation according to high-level taxonomy (Fig. 4). See Supplementary Fig. 13 for a version with more annotation. Several proteobacterial genera (purple circles) including *Klebsiella*, *Escherichia, Pseudomonas* and were each host to many different ARGs. Some of these were shared with other *Proteobacteria* (on edges tying the purple cluster together), while many others were uniquely seen in their respective genera (unconnected edges extending outside the cluster).

*Firmicutes* similarly formed a (blue) phylum-level cluster with the *Enterococcus*, *Staphylococcus* and *Streptococcus* as genera with important pathogen members. Also, *Lactococcus* and *Exiguobacterium* were part of the gene-sharing cluster where they shared specifically *mef*(A) and *msr*(D) with *Enterococcus* frequently.

Amongst the Gram-positive bacteria, *Enterococcus* was assigned most different ARGs, and mainly shared these with other known Gram-positive species, but also the Gram-negative genus *Campylobacter*. This largely confirms observations from clinical isolates and suggests that the current choices of *Escherichia coli* and *Enterococcus faecalis* as indicator species for surveillance of AMR is appropriate^31^.

Interestingly, stratifying the dataset by geographical region, networks revealed that cross-phylum edges were more common in Sub-Saharan Africa (Supplementary Fig. 14). This could indicate that some regional conditions facilitate cross-phylum transmission. In fact, sharing of ARGs between *Proteobacteria* and *Firmicutes* seen in the larger network, stems mostly from the inclusion of samples from this region.

### Very few ARGs remain stable and have fixed syntenies

Detailed analyses of ARG variant and gene flanking regions including the exact gene synteny and the association with bacterial plasmid, taxa geographical regions were conducted for the 49 most common ARGs.

A specific ARG and its neighbouring genes that share a host organism and only transmit vertically through time will have the same amount of time to diverge from their respective orthologs in a cousin lineage. Therefore, one might expect to derive a similar phylogenetic relationship of the ARG and the neighbouring genes. A frequently mobilized ARG on the other hand, could be very similar across all samples, even if its surrounding sequence varies considerably. This is also the basis of gene synteny analysis, where the surrounding genes’ order and identities are used to infer evolutionary events. Frequent gene shuffling could create seemingly different flanking regions for identical genes, whereas equal divergence in gene and flank could suggest long-term stable association and shared divergence.

**Fig. 5:**
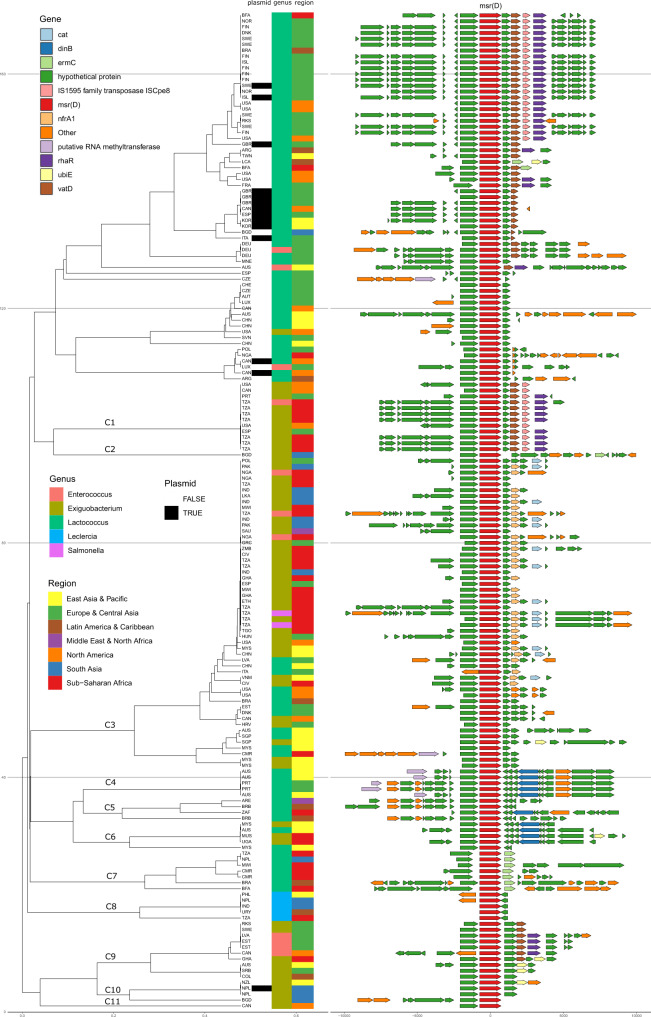
ARG Flanks show diverse genomic contexts. Flanking sequence for *msr*(D) variants in the sewage were used to calculate a kmer distance UPGMA tree. The tree shows contig-level annotation: plasmid annotation status, predicted genera and World Bank region. For ARG synteny analyses, all variants of the ARG were oriented and centered, with up- and down-stream predicted features drawn in. A subset of clusters is labelled to allow referencing. All common ARGs are clustered like this according to flank and gene (Supplementary Data 3 and 4) and so is the subset with 5Kb flanks (Supplementary Data 5 and 6).

To elucidate the diversity of genomic backgrounds of ARGs, we clustered their immediate 1 Kb flanking regions based on sequence k-mer dissimilarity, separately for each of the 49 most frequently assembled ARGs (Supplementary Data 3). We carried out the same analysis for the actual gene variant sequences (Supplementary Data 4). The association between each ARGs’ dissimilarity matrix and its corresponding flank dissimilarity matrix (Supplementary Fig. 15), as well as the gene synteny (Fig. 5) was determined.

When evaluating the 49 ARGs, it becomes obvious that no common evolutionary trajectory can account for ARGs generally. The strength of association between gene and flank varied drastically between ARGs. Some ARGs, like *fosA*, showed practically identical clustering based on ARG and flank, suggesting long-term stable association and co-evolution (Supplementary Fig. 15b, d). Other ARGs, like *qnrS2*, were almost identical across all samples, but had highly variable flanks and was found in multiple bacterial taxa, and sometimes on plasmids, indicating recent mobilization followed by frequent genomic transfer and re-organization (Supplementary Fig. 15a, c).

*msr*(D) is an example of an ARG with many different genetic and taxonomic contexts in a wide geographic range (Fig. 5). In Cluster 1 (C1) for example, it appears to have been captured by the IS1595 transposase and spread across Africa, Europe, and North America, mostly while in *Exiguobacterium*. C3 was also mostly *Exiguobacterium*-assigned and was frequently observed with the chloramphenicol resistance gene *cat*. These sequences were also seen in *Lactococcus* and in a few instances in *Enterococcus* and *Salmonella* in Tanzania and Nigeria. Interestingly, Tanzania contained highly identical contigs with *msr*(D) scaffolds assigned to both *Exiguobacterium*, *Salmonella* and *Enterococcus*. The presence of identical ARGs in identical immediate contexts but in several different hosts suggest proximity to a transmission hotspot, which warrants further investigation. Indeed, *catQ* was also found in Tanzania in very similar local contexts but assigned to variable hosts, supporting this concern.

C7 was exclusively in *Lactococcus* and mostly in Africa, the ARG had somewhat variable upstream flanks, but consistently had the macrolide resistance gene *erm*(C) downstream. C8 sequences were all assigned to *Leclercia* and from diverse geographical regions. C9 sequences were assigned to *Exiguobacterium* and in a highly identical European subset, it appeared to have spread to *Enterococcus*. In summary, several sequence clusters, geography and taxonomy co-occurred, suggesting geographical selection of specific bacterial taxa in accordance with the observations that the microbes and resistome are shaped mainly by biotic factors^22,23^.

For most ARGs, we observed that clusters of variants were confined in the same gene synteny and that different gene syntenies were associated with different genetic variants (Supplementary Data 3 and 4). This suggests that once mobilized and established in a new host, and even on a global scale, re-organization and shuffling of ARGs and their surrounding genes does not frequently occur. If it did, we would not have that kind of globally widespread conservation. Exceptions might be some of the ARGs associated with macrolide resistance, such as *erm*(B), *mef(*C), *mph*(G) and *lnu*(D).

For some genes, like *lsa*(E), there were clear geographical clustering of one or more of the sub-variants, suggesting geographical dispersal limitations. A large cluster of sequences, which were frequently plasmid-associated, however seem to have spread more quickly across multiple Asian and African countries.

Other examples were observed among the 49 ARGs more closely studied. Please see the Supplementary Notes for further discussion of the individual ARGs, flanking variation, including gene synteny, their clustering and association to each other. However, despite some ARGs displaying signs of archetypical transmission modes, there were frequent outliers, highlighting that biology is full of exceptions.

Taken together, the results highlight the fact that a plethora of evolutionary roads have been trodden by the modern ARGs that make up global resistomes. Being on very different evolutionary trajectories, we should not expect a single model to explain them all. Different studies that have reached slightly different conclusions, and favoured different evolutionary models, are not necessarily contradicting each other, since the overarching pattern will depend on the specific ARG, its geography and genomic background.

## Discussion

Continuous and comparable AMR surveillance through time is essential to determine the effects of interventions, identify national and global priorities and identify areas for further research. We have previously identified urban sewage as a potential area for standardized metagenomic surveillance which can support other surveillance activities^7,32^. In a pilot study we showed the feasibility of the concept and found that the resistome to a large degree correlated to major geographic and geopolitical groupings.

In this study we confirmed the high degree of local and regional stability in the resistome even when sampled over a three-year period. No obvious trends in time were observed, similar to recent results from Copenhagen and supporting the view that broad changes in AMR take place over several years or even decades, and that any significant impact of interventions aimed at reducing AMR may equally be delayed^33^. Seasonality also had a very modest effect (<2%) on the resistome composition. Importantly, however, we observed that the global patterns and clusters were heavily dependent on the antimicrobial class in question. This highlights the many underlying patterns that are lost when naively considering the entire resistome as a single entity.

In our data, we did observe spread of ARGs, sometimes in many different genomic backgrounds and in many countries. Though culture-independent surveillance like this is clearly needed to discover novel ARG contexts and synteny, *E. coli* appears to be well-chosen monitoring target for culture-based surveillance, given its centrality in our networks and frequent ARG assignments. The importance of *E. coli* as a facilitator for increased ARG transmission has also been noted by others investigating infant gut resistomes^34^.

Our data could indicate that crossing vast evolutionary boundaries happen more in Sub-Saharan Africa. This should be confirmed with a different approach that can make more definitive taxonomic classifications though. Horizontal transfer of ARGs is much more likely to occur between phylogenetically closely related bacteria^35,36^. This is for multiple reasons, including codon usage, restriction-modification systems, and limitations in host range of both conjugative elements and bacteriophages that make up important dissemination vectors for ARGs^37^. It was therefore expected to see strong phylum-separation in our ARG-sharing network. Exceptions to the rule could however, help provide insight into ARG transmission and *Campylobacter* is an obvious and prominent exception to this rule.

*Campylobacter* is a proteobacterial genus and the single most common cause for bacterial gastroenteritis. In this sewage-based network, it clustered with *Firmicutes* genera, rather than other members of its phylum. When *erm*(B) was first observed in *Campylobacter*, it was indeed on a large multi-drug resistance genomic island of Gram-positive origin, carrying multiple ARGs^38^. The flux of ARGs from Gram-positive cocci to Gram-negative bacteria has been previously discussed, with broad-range conjugative plasmids being deemed the most likely culprits^39^. Whether *Campylobacter*’s natural competence helps explain its phylum-defying position in the network is yet to be determined.

Zhang et al. looked at a comprehensive collection of public genomes and MAGs and found that most ARGs are carried by less than 10 different MGEs and are either exclusively carried by commensals or pathogens^40^. A developed risk index stated that 24% of the metagenomic ARGs were a risk to human health. Among the thousands of ARGs uncovered in our data, many of them are variants not in ResFinder, are mobile and known to occur in human pathogens. Continued and extended analysis of ARGs in their flanking environments across public metagenomes should help us get better ideas about how individual ARGs, MGEs, and strains disperse. We hope that sharing of our sewage sequence data, especially from countries previously lacking microbiome data, will greatly help facilitate such future cross-consortium projects.

One might wonder whether variable transport lengths from households to treatment plants affect the measured resistome. Pehrsson et al. found that street-access sewage resistomes were very similar to downstream WWTP influent, while both sample types were compositionally different from human feces and sewage treatment effluent^41^. The same could not be said for the bacteriome, indicating resistome analysis at the point of the WWTP is not particularly sensitive to in-sewage perturbations. Measurements on microbiomes/resistomes are also sensitive to storage conditions, time before freezing and freeze-thaw cycles and it appears that even sample-specific conditions can change the microbiome profile differently over time^42^.

The more qualitative (as opposed to quantitative) analyses, such as epidemiological comparisons of ARG variants and their synteny, are likely less sensitive to minor perturbations. Hospitals, farms and industry (including antimicrobial-producing) present in the catchment area will result in different selective environments and microbiome changes and it is also important to keep in mind that not all ARGs will be from human city dwellers, which is potentially a feature for combined urban monitoring.

Large differences in ARG richness per city was observed in a recent global study including thousands of microbes from mass transit system surfaces in 60 cities by Danko et al.^43^. In that study, same as here and in our pilot study, the AMR class composition also showed a significant geographical signature, both at the city and world region level. In the study by Danko et al., there were thousands of samples without any observed AMR, and there was no set of core ARGs. Each sampling type has its own pros and cons, and it is not surprising that these two fundamentally different proxies to the urban environment also have differences in outcome. Sewage has the benefit of much larger DNA quantities and not being restricted to cities with mass transit. One might also expect a specific and biased subset of a population are users of the public transport system. Every person in a city, regardless of age, financial status, and transport needs, contribute to the sewage system out of biological necessity. With the growing importance of microbiome data, more studies generating more datasets with novel surveillance methods across more matrices should be encouraged.

While sewage has benefits as a surveillance matrix, it also has some inherent characteristics that should be considered. Differences in the natural or built environments that sewage flow through, chemical pollutants etc. will influence the final microbiome. Indeed, the local climate, environmental conditions and surface material sampled, in addition to geography, have proven important in other comprehensive microbiome studies^23,43^. Indeed, some of our lowest AMR measurements were from a location in Madagascar, where there is no formal sewage treatment and the sewage flow into a lake. It is almost certain that some of our variation is due to unobserved conditions at sampling sites. Danko et al. showed that climate was an important driver of urban microbiome and resistome differences in transport systems, which of course should also be true for sewage^43^.

All partners here used a shared protocol and we verified samples were still frozen at the time of arrival, but variations in package travel time could be another unwanted source of variation. Even though we did not see obvious systematic differences, geographical distances between the sampling sites and Denmark naturally confounded package travel duration. Future studies would do well in prioritizing very careful and systematic collection of metadata that can systematically highlight confounders, as we constantly learn of new things that can affect microbiomes.

Even though our metagenomic assemblies revealed a lot of information on probable taxonomic origin and the immediate context, it too has limitations. Given the many city-dwellers contributing to the wastewater system, sewage is naturally home to many different strains of the same species, which is difficult for the assembly process. The metagenome was highly fragmented and even though, genome binning was performed, high fragmentation may cast doubt on whether binned ARGs were assigned to their correct genome bins. We therefore did not use binning results here and instead relied on scaffold-level taxonomic assignment. This is obviously database-dependent and biased by our current knowledge, same as in any other metagenomic classification study. A known transposon sitting in the genome of a novel species, would, for example be classified to the known host, if the assembler and scaffolder were unable to link the genome and transposon. For broad host-range plasmids, no assembly-based method can assign them to a given host. Our taxonomic assignments thus represent our most qualified guess, but are likely to include some conservative misclassifications, especially among the plasmid-annotated contigs, which we therefore excluded from certain analyses.

Future supplementing sequencing with long Nanopore reads of sewage could potentially enable more robust assignment of ARGs to novel backgrounds. Indeed such a strategy on plasmid extractions from a subset of the Global Sewage samples, was able to reconstruct many plasmids, some of which encoded ARGs^44^. A web server specifically aimed at resolving ARG context based on metagenomic Nanopore reads was also recently published, and could be of help in such cases^45^. For plasmid-borne ARGs, methods involving genomic crosslinking (Hi-C) or single-cell sequencing, have also proven helpful in hosts to genes with higher certainty^46,47^. Indeed, the former has previously been used to show discrete ARG transfer networks in individual patients over merely weeks of antimicrobial therapy^48^.

Going forward, it would be valuable to determine exactly how closely the spatial variations in the sewage microbiome and resistome are mirrored in human faecal samples. Pehrsson et al. previously found a significant shift from the donor hosts in rural South America compared to nearby street-level sewage, but whether such shifts are generalizable and globally true is unknown^41^.

It is tempting to compare our resistome composition to previously generated faecal resistomes, but a major challenge for cross-study comparisons is wet lab protocol differences. Our DNA extraction SOP and workflow used here was optimized for bacterial diversity and validated for sewage, human and pig faeces, but different studies and consortia have opted for different protocols with different biases^49^. Epidemiological and qualitative comparisons of ARG contexts and synteny will be more suited for such future inter-study comparisons, even though the lack of a genetic feature will have to be carefully interpreted.

Based on our data, we believe that the same ARGs will respond very differently to the same intervention worldwide as geography confounds both differences in genomic background, plasmid carriage, co-resistance, and mobilization potential to a much larger degree than we anticipated. This suggests that solutions will need to be adapted to local conditions. National Action Plans should be supported by local genomic surveillance that can inform interventions and be used to evaluate outcomes, whether they be local, national, or even regional. These could involve the tried and tested avoidance of specific antibiotics, but alternative anthropogenic interventions such as dietary changes, which modulate the human microbiome, as well as factors leading to increased transmission are also of potential interest. No matter the chosen intervention, sewage genomics can enable monitoring of the local AMR load, variants, diversity, and syntenies in the urban reservoir. For simple complementary passive surveillance of the global AMR burden, annual samples from major cities may be sufficient, but to separate geographical from seasonal effects over both hemispheres, we suggest at least two annual samples per site are required.

## Methods

### Sampling, DNA-purification, and sequencing

Collection of sewage from global sites, DNA-purification, and sequencing were organized and conducted similarly to the previous study^7^. Briefly, on request, empty bottles were shipped to worldwide project partners, who were briefed on sample inclusion criteria. Only samples of raw, untreated, urban sewage prior to any processing were requested. For locations without any sewage treatment, samples were gathered at the sewage outlet into e.g., a river or lake. This was conducted bi-annually in June and November over the study period to account for seasonal differences which are important at the global scale. Additionally, a subset of sites across six geographical regions were longitudinally sampled from 2017–2018. We also included samples from an additional longitudinal Tanzanian campaign and from confirmatory samples taken the day after the original 2016 sampling round (Supplementary Fig. 16).

Additional photos and metadata were collected about the sites, samples, and shipping condition. Analyses in the pilot study did not reveal obvious confounders variables, but the data sources were used to identify and exclude a number of samples that were deemed inappropriate or outside the scope of the study, e.g., a number of samples from non-municipal sewage systems like schools and hotels.

Bottles containing frozen sewage were shipped back to Technical University of Denmark (DTU) for QC, DNA extraction and vacuum up-concentration when needed. Aliquots of 100+ ng Qubit-quantified metagenomic DNA were then shipped on dry ice for library preparation and DNA sequencing at Admera Health (New Jersey, USA).

All newer samples, absent in the earlier pilot study, were sequenced on the Illumina NovaSeq6000 instrument as opposed to the HiSeq4000. The same fragment size, read lengths and sequencing depth were targeted and the choice of PCR-free Kapa Hyper library prep was also kept constant throughout the project. A comparison of resistomes analysed on the two different machines, did not reveal any detectable instrument effect (Supplementary Fig. 17). See Supplementary Data 1 for metadata on the sequencing datasets and samples used, as well as ENA accessions.

### Bioinformatics and QC

All bioinformatics analysis was performed on the Danish National Life science supercomputer, Computerome2 on nodes each equipped with dual 20-core Intel Xeon Gold 6230 CPUs and either 192GB or 1.5TB RAM. The latter high-memory nodes were used for assembly, which required >500GB memory for the most diverse samples.

Each of the paired-end sequencing libraries were quality- and adapter-trimmed with an in-house wrapper script utilizing BBduk2, which is part of the BBmap suite of NGS tools^50^. We removed common adapters and trimmed the 3′-end of the reads to a sliding window, using a Phred score of Q20, corresponding to a 1% error rate.

General data manipulation was carried out in R.

### Genomic and taxonomic context of ARGs

The paired and singleton reads from the individual sequence runs were then subjected to metagenomic assembly using metaSPAdes (SPAdes v. 3.13.0) with the following k-mer sizes: 27,47,67,87,107,127 and the “pre-correction” flag set^51^. This ends on the largest allowed k-mer, is still below the read length and uses the default increment of 20. The settings were chosen to promote larger unbroken contigs, minimize the risk of chimeric variants and maximize the chance to bridge known repetitive regions near ARGs. To avoid issues with different fragment size distributions, the deepest sequence run was used for samples with multiple sequence runs. Scaffolds smaller than 1 Kb were filtered away. The scaffolded contigs were searched for ARGs using the ResFinder tool with default settings (>90% identity, >60% covered) and without a specific taxa selected^52^.

To determine the genomic diversity of AMR gene backgrounds, we extracted the flanking regions of ResFinder hits in the following manner. ResFinder hits within an assembled contig were masked with N’s and 1 Kb up- and down-stream of the masked region were extracted with BEDtools (v 2.28.0)^53^. ResFinder hits with shorter than 1 Kb to a contig end were excluded from flank-based analyses. While somehow arbitrary, the 1 Kb threshold was a compromise between retaining many ARGs and simultaneously requiring enough k-mers for detailed resolution. The threshold was also set to roughly correspond to a single gene on either site of the target and prohibit comparisons between unrelated genome positions on larger scaffolds. Synteny analyses were also run for observed ARGs that could satisfy 5 Kb flanking regions. Per-ARG plots for those can be found clustered on flank dissimilarity (Supplementary Data 5) and ARG dissimilarity (Supplementary Data 6).

The metagenomic scaffolds with ResFinder hits were assigned taxonomically using Kraken 2 (v. 2.0.8) to the premade Base collection database (2020/9)^54^. Taxa were evaluated at taxonomic levels from genus to phylum.

### Clustering of ARGs and flanking sequences

The number of k-mers shared between the flanking sequence (1 Kb) was used to compute the Szymkiewicz–Simpson dissimilarity index using the KMA dist subcommand (v. 1.3.3)^55^. This index gives the proportion of k-mers in a contig that are contained within another.

The Szymkiewicz–Simpson dissimilarity matrix was also computed for the variants of each ARG and compared to the corresponding flank Szymkiewicz–Simpson dissimilarity matrix. For each gene, UPGMA dendrograms were constructed from the flank and gene matrices and were compared to each other using tanglegrams, in order to determine whether tree topology was similar for flanking and gene sequence. For each gene, up to 100 iterations of the step2side method was used to untangle the tanglegram as much as possible, after which the entanglement score was calculated using the dendextend R package (v. 1.14.0)^56^. In additional to that, the flank and gene dissimilarity matrices for each gene were ordinated to two dimensions using classical multidimensional scaling (PCoA) and correlated using Mantel and Procrustes correlation using the vegan R package (v. 2.6.2)^57^. This was conducted to obtain correlation coefficients, which should be more robust to identical sequences in a subset of the samples, which result in random tree topology. For a schematic of the workflow for comparing gene sequences and their immediate flanking regions, see Supplementary Fig. 18.

### Classification of plasmid scaffolds

Metagenomic scaffolds with one or more ResFinder hits with at least 1Kb flank on either side were then subjected to additional screening for potential plasmid origin. We downloaded the PLSDB database (v. 2020_11_19) and queried the contigs against it using BLASTn, requiring at both 90% identity and query coverage^58^.

The scaffolds were also queried against a custom Kraken 2 database with the plasmids in an artificial domain-level plasmid group^54^. This allows each sequence to be classified as chromosomal or plasmidic based on its k-mer content.

Lastly, we ran PPR-Meta (v. 1.1) with default parameters on the scaffolds^59^. Scaffolds which had at least two classifiers agreeing on plasmid origin, were labelled as plasmidic.

### Sequence clustering association to sample geography and season

The degree and significance of clustering based on World Bank regions and seasonality was determined individually using permutational ANOVA (adonis2, vegan^57^). Samples were classified depending on if which half-year they were sampled in, and the sign was then changed depending on positive/negative latitude. For each ARG, the effect of region on flanking dissimilarity was separately established for flanks assigned the most frequent genus.

### Antimicrobial resistance quantification

Trimmed reads of all libraries were aligned with KMA (v. 1.2.17a) to the ResFinder database of known and acquired resistance genes (Commit 813679d) and the Silva (Silva version 138) database made from more than 13 million sequences 16/18 S rRNA, which attempts to cover all cellular life and include thousands of bacterial species across 89 phyla^52,60^. KMA was used to generate mapstat files summarizing abundance in each sample using the trimmed paired-end and singleton reads per sample as described below.

For ResFinder, KMA was used with the following arguments: -mem_mode -ef −1t1 -cge -nf -vcf -shm 1 -t 1. A total of 20.3 M sequence fragments were assigned to ResFinder ARGs with 19.9 M ( >98%) of them aligned to templates at least half covered. In the alignments, the read-reference identities ranged between 90.44 and 100% (median: 99.68%).

The sewage samples contain variable proportions of non-bacterial DNA, which is not relevant to ARG quantification. Since we want a measure to reflect how much resistance is carried by the bacteria in the sample, we adjusted the number of ARG-assigned fragments to a proxy for bacterial load per sample. To accomplish this, we aligned reads to the Silva 16/18 S rRNA gene database with the following arguments: -mem_mode -ef −1t1 -apm f -nf -t 3.

The Silva fragments assigned to the Bacteria domain were summed up per sample for use as a normalization factor. To calculate a relative abundance (other than for PCA), aligned ARG fragment counts per sample were adjusted both for their ResFinder reference template and the sample-specific number of fragments assigned to the superkingdom Bacteria. This was done according to Eq. (1) in order to obtain a relative abundance of Fragments Per Kilobase reference per Million bacterial fragments:

1 $${{{\rm{Relative}}}}\; {{{\rm{Abundance}}}}=\frac{{{{\rm{ARG}}}}_{{{\rm{Fragments}}}}}{{{{{\rm{ARG}}}}_{{{\rm{Length}}}}}\times {{{\rm{Bacteria}}}}_{{{\rm{Depth}}}}}\times {10}^{9}$$

Where ARG_Fragments_ is the number of sequencing fragments assigned to a reference sequence, ARG_Length_ is the length of the ARG reference and Bacteria_Depth_ is the sum of fragments assigned specifically to Silva sequences in the superkingdom Bacteria.

### AMR abundance at higher levels

With the Conclave winner-takes-it-all strategy, KMA assigns unanimous reads to the most parsimonious reference sequence, but given very low abundance, the algorithm might assign reads from the same ARG to variable reference bins in different samples, obscuring meaningful inter-sample comparison. To identify close homologs that KMA could potentially assign differently across samples, we used USEARCH (v. 11.0.7) to homology reduce ResFinder ARG collection to 90% nucleotide identity, with query and target coverage thresholds of 0.9^61^. These homology groups were then used to bin the abundance of minor variant-assigned reads to their representative sequences, which was also used to name the groups.

In addition to binning abundance of variants to ARG level, abundance was also binned to the level of drug classes and phenotypes as annotated in “ResFinder [https://bitbucket.org/genomicepidemiology/resfinder_db/src/master/phenotypes.txt]”.

### Principal components analysis

The principal component analysis (PCA) was carried out in respect to the compositional nature of metagenomic data. Briefly, zeros were imputed using the method highlighted in the supplement of previous work^62^. For each biplot, the subset of length-corrected counts was centered by the geometric sample mean and scaled by the total log-ratio variance followed by a centered log-ratio (CLR) transformation. The resulting CLR coefficient matrix was eigen-decomposed into eigen-vectors and -values from which the principal components were obtained and plotted as previously recommended^63^. The analysis was performed using “the PyCoDa package [https://bitbucket.org/genomicepidemiology/pycoda]” in Python (v. 3.7).

### Alpha diversity analysis

For calculating resistome alpha diversity, samples with less than 1000 ResFinder-assigned fragments were filtered away. The ResFinder counts were randomly subsampled without replacement to the depth of the new sample with fewest counts using the vegan R package (v. 2.6.2)^57^. Alpha diversity for bacterial genera was performed identically, with the exception that 10,000 counts were required per sample.

### Network analysis of bacteria and ARGs

Scaffolds that satisfied the following criteria were considered for network analysis: (1) Were Kraken 2-assigned to the superkingdom Bacteria, (2) Encoded an ARG according to the ResFinder tool result, (3) Had at least 1 Kb sequence up- and down-stream from the found ARG, (4) had Kraken 2 taxonomic assignment at the genus level. Additionally, we required at least 2/3 plasmid-annotation programs agreed the scaffold was not of plasmid origin.

In the set of scaffolds surviving the above-mentioned filtering, we counted the number of ResFinder ARGs occurring on sequences from each genus and used these as edge weights. Subgraphs of connected bacteria and ARG nodes were then laid out and visualized using the backbone algorithm and the ggraph R package (v. 2.0.5).

### Reporting summary

Further information on research design is available in the Nature Research Reporting Summary linked to this article.

## Supplementary information

Supplementary Info

Description of Additional Supplementary Files

Supplementary Dataset 1, Supplementary Dataset 2

Supplementary Dataset 3, Supplementary Dataset 4, Supplementary Dataset 5, Supplementary Dataset 6

Reporting Summary

## Data Availability

The raw sequencing data (FASTQ) generated in this study have been deposited in the European Nucleotide Archive and can be accessed without restrictions. The data from major sampling rounds have the following project accession numbers: PRJEB40798, PRJEB40816, PRJEB40815 and PRJEB27621. Sequencing data from the longitudinal city sampling sites are deposited under PRJEB51229, while the included datasets from the previous study are deposited under ERP015409. See Supplementary Data 1 for exact sample, experiment and run accessions. Source data are provided with this paper. This study also utilized the publicly available databases of ResFinder, PLSDB, Silva and Kraken 2.
