# Author Correction: Genomic analysis of sewage from 101 countries reveals global landscape of antimicrobial resistance

**DOI:** 10.1038/s41467-023-35890-w

**Published:** 2023-01-12

**Authors:** Patrick Munk, Christian Brinch, Frederik Duus Møller, Thomas N. Petersen, Rene S. Hendriksen, Anne Mette Seyfarth, Jette S. Kjeldgaard, Christina Aaby Svendsen, Bram van Bunnik, Fanny Berglund, Global Sewage Surveillance Consortium, Artan Bego, Pablo Power, Catherine Rees, Dionisia Lambrinidis, Elizabeth Heather Jakobsen Neilson, Karen Gibb, Kris Coventry, Peter Collignon, Susan Cassar, Franz Allerberger, Anowara Begum, Zenat Zebin Hossain, Carlon Worrell, Olivier Vandenberg, Ilse Pieters, Dougnon Tamègnon Victorien, Angela Daniela Salazar Gutierrez, Freddy Soria, Vesna Rudić Grujić, Nataša Mazalica, Teddie O. Rahube, Carlos Alberto Tagliati, Dalia Rodrigues, Guilherme Oliveira, Larissa Camila Ribeiro de Souza, Ivan Ivanov, Bonkoungou Isidore Juste, Traoré Oumar, Thet Sopheak, Yith Vuthy, Antoinette Ngandjio, Ariane Nzouankeu, Ziem A. Abah Jacques Olivier, Christopher K. Yost, Pratik Kumar, Satinder Kaur Brar, Djim-Adjim Tabo, Aiko D. Adell, Esteban Paredes-Osses, Maria Cristina Martinez, Sara Cuadros-Orellana, Changwen Ke, Huanying Zheng, Li Baisheng, Lok Ting Lau, Teresa Chung, Xiaoyang Jiao, Yongjie Yu, Zhao JiaYong, Johan F. Bernal Morales, Maria Fernanda Valencia, Pilar Donado-Godoy, Kalpy Julien Coulibaly, Jasna Hrenovic, Matijana Jergović, Renáta Karpíšková, Zozo Nyarukweba Deogratias, Bodil Elsborg, Lisbeth Truelstrup Hansen, Pernille Erland Jensen, Mohamed Abouelnaga, Mohamed Fathy Salem, Marliin Koolmeister, Mengistu Legesse, Tadesse Eguale, Annamari Heikinheimo, Soizick Le Guyader, Julien Schaeffer, Jose Eduardo Villacis, Bakary Sanneh, Lile Malania, Andreas Nitsche, Annika Brinkmann, Sara Schubert, Sina Hesse, Thomas U. Berendonk, Courage Kosi Setsoafia Saba, Jibril Mohammed, Patrick Kwame Feglo, Regina Ama Banu, Charalampos Kotzamanidis, Efthymios Lytras, Sergio A. Lickes, Bela Kocsis, Norbert Solymosi, Thorunn R. Thorsteinsdottir, Abdulla Mohamed Hatha, Mamatha Ballal, Sohan Rodney Bangera, Fereshteh Fani, Masoud Alebouyeh, Dearbhaile Morris, Louise O’Connor, Martin Cormican, Jacob Moran-Gilad, Antonio Battisti, Elena Lavinia Diaconu, Gianluca Corno, Andrea Di Cesare, Patricia Alba, Junzo Hisatsune, Liansheng Yu, Makoto Kuroda, Motoyuki Sugai, Shizuo Kayama, Zeinegul Shakenova, Ciira Kiiyukia, Eric Ng’eno, Lul Raka, Kazi Jamil, Saja Adel Fakhraldeen, Tareq Alaati, Aivars Bērziņš, Jeļena Avsejenko, Kristina Kokina, Madara Streikisa, Vadims Bartkevics, Ghassan M. Matar, Ziad Daoud, Asta Pereckienė, Ceslova Butrimaite-Ambrozeviciene, Christian Penny, Alexandra Bastaraud, Tiavina Rasolofoarison, Jean-Marc Collard, Luc Hervé Samison, Mala Rakoto Andrianarivelo, Daniel Lawadi Banda, Arshana Amin, Heraa Rajandas, Sivachandran Parimannan, David Spiteri, Malcolm Vella Haber, Sunita J. Santchurn, Aleksandar Vujacic, Dijana Djurovic, Brahim Bouchrif, Bouchra Karraouan, Delfino Carlos Vubil, Pushkar Pal, Heike Schmitt, Mark van Passel, Gert-Jan Jeunen, Neil Gemmell, Stephen T. Chambers, Fania Perez Mendoza, Jorge Huete-Pιrez, Samuel Vilchez, Akeem Olayiwola Ahmed, Ibrahim Raufu Adisa, Ismail Ayoade Odetokun, Kayode Fashae, Anne-Marie Sørgaard, Astrid Louise Wester, Pia Ryrfors, Rune Holmstad, Mashkoor Mohsin, Rumina Hasan, Sadia Shakoor, Natalie Weiler Gustafson, Claudia Huber Schill, Maria Luz Zamudio Rojas, Jorge Echevarria Velasquez, Bonifacio B. Magtibay, Kris Catangcatang, Ruby Sibulo, Felipe Campos Yauce, Dariusz Wasyl, Celia Manaia, Jaqueline Rocha, Jose Martins, Pedro Álvaro, Doris Di Yoong Wen, Hanseob Shin, Hor-Gil Hur, Sukhwan Yoon, Golubinka Bosevska, Mihail Kochubovski, Radu Cojocaru, Olga Burduniuc, Pei-Ying Hong, Meghan Rose Perry, Amy Gassama, Vladimir Radosavljevic, Moon Y. F. Tay, Rogelio Zuniga-Montanez, Stefan Wuertz, Dagmar Gavačová, Katarína Pastuchová, Peter Truska, Marija Trkov, Karen Keddy, Kerneels Esterhuyse, Min Joon Song, Marcos Quintela-Baluja, Mariano Gomez Lopez, Marta Cerdà-Cuéllar, R. R. D. P. Perera, N. K. B. K. R. G. W. Bandara, H. I. Premasiri, Sujatha Pathirage, Kareem Charlemagne, Carolin Rutgersson, Leif Norrgren, Stefan Örn, Renate Boss, Tanja Van der Heijden, Yu-Ping Hong, Happiness Houka Kumburu, Robinson Hammerthon Mdegela, Yaovi Mahuton Gildas Hounmanou, Kaknokrat Chonsin, Orasa Suthienkul, Visanu Thamlikitkul, Ana Maria de Roda Husman, Bawimodom Bidjada, Berthe-Marie Njanpop-Lafourcade, Somtinda Christelle Nikiema-Pessinaba, Belkis Levent, Cemil Kurekci, Francis Ejobi, John Bosco Kalule, Jens Thomsen, Ouidiane Obaidi, Laila Mohamed Jassim, Andrew Moore, Anne Leonard, David W. Graham, Joshua T. Bunce, Lihong Zhang, William H. Gaze, Brett Lefor, Drew Capone, Emanuele Sozzi, Joe Brown, John Scott Meschke, Mark D. Sobsey, Michael Davis, Nicola Koren Beck, Pardi Sukapanpatharam, Phuong Truong, Ronald Lilienthal, Sanghoon Kang, Thomas E. Wittum, Natalia Rigamonti, Patricia Baklayan, Chinh Dang Van, Doan Minh Nguyen Tran, Nguyen Do Phuc, Geoffrey Kwenda, D. G. Joakim Larsson, Marion Koopmans, Mark Woolhouse, Frank M. Aarestrup

**Affiliations:** 1 Research Group for Genomic Epidemiology, Technical University of Denmark, Kgs, Lyngby, Denmark; 2 Centre for Immunity, Infection and Evolution, University of Edinburgh, Edinburgh, UK; 3 Centre for Antibiotic Resistance Research (CARe), University of Gothenburg, Gothenburg, Sweden; 4 Department of Viroscience, Erasmus MC, Rotterdam, The Netherlands; 5 Institute of Public Health, Tirana, Albania; 6 Universidad de Buenos Aires, Buenos Aires, Argentina; 7 Melbourne Water Corporation, Melbourne, Australia; 8 Charles Darwin University, Darwin, Australia; 9 University of Copenhagen, Frederiksberg C, Denmark; 10 Charles Darwin University, Darwin Northern Territory, Australia; 11 Canberra Hospital, Canberra, Australia; 12 ALS Water, Scoresby, Australia; 13 Austrian Agency for Health and Food Safety (AGES), Vienna, Austria; 14 University of Dhaka, Dhaka, Bangladesh; 15 Environmental Protection Department, Bridgetown, St. Michael, Barbados; 16 Laboratoire Hospitalier Universitaire de Bruxelles (LHUB-ULB), Brussels, Belgium; 17 AQUAFIN NV, Aartselaar, Belgium; 18 Polytechnic School of Abomey-Calavi, Abomey-Calavi, Benin; 19 Universidad Catσlica Boliviana San Pablo, La Paz, Bolivia; 20 Public Health Institute of the Republic of Srpska, Faculty of Medicine University of Banja Luka, Banja Luka, Bosnia and Herzegovina; 21 Public Health Institute of the Republic of Srpska, Banja Luka, Bosnia and Herzegovina; 22 Botswana International University of Science and Technology, Palapye, Botswana; 23 Universidade Federal de Minas Gerais, Belo Horizonte, Brazil; 24 Oswaldo Cruz Institute, Rio de Janeiro, Brazil; 25 Vale Institute of Technology, Belιm, PA Brazil; 26 National Center of Infectious and Parasitic Diseases, Sofia, Bulgaria; 27 University of Ouagadougou, Ouagadougou, Burkina Faso; 28 Institut Pasteur du Cambodge, Phnom Penh, Cambodia; 29 Centre Pasteur du Cameroun, Yaoundι, Cameroon; 30 University of Regina, Regina, Canada; 31 Eau Terre Environnement Research Centre (INRS-ETE), Quebec City G1K 9A9, Canada and Indian Institute of Technology, Jammu, India; 32 Eau Terre Environnement Research Centre (INRS-ETE), Quebec City G1K 9A9, Canada and Lassonde School of Enginerring, York University, Toronto, Canada; 33 University of N’Djamena, N’Djamena, Chad; 34 Escuela de Medicina Veterinaria, Facultad de Ciencias de la Vida, Universidad Andres Bello, Santiago, Chile; 35 Institute of Public Health, Santiago, Chile; 36 Universidad Catolica del Maule, Centro de Biotecnología de los Recursos Naturales, Facultad de Ciencias Agrarias y Forestales, Talca, Chile; 37 Guangdong Provincial Center for Disease Control and Prevention, Guangzhou, China; 38 The Hong Kong Polytechnic University, Hong Kong, China; 39 Shantou University Medical College, Shantou, China; 40 Nanjing University of Information Science and Technology, Nanjing, China; 41 Center for Disease Control and Prevention of Henan province, Zhengzhou, China; 42 Colombian Integrated Program for Antimicrobial Resistance Surveillance - Coipars, CI Tibaitatα, Corporaciσn Colombiana de Investigaciσn Agropecuaria (AGROSAVIA), Tibaitatα - Mosquera, Cundinamarca Colombia; 43 Institut Pasteur de Côte d’Ivoire, Abidjan, Côte d’Ivoire; 44 University of Zagreb, Zagreb, Croatia; 45 Andrija Stampar Teaching Institute of Public Health, Zagreb, Croatia; 46 Veterinary Research Institute, Brno, Czech Republic; 47 Centre de Recherche en Sciences Naturelles de Lwiro (CRSN-LWIRO), Bukavu, Democratic Republic of Congo; 48 BIOFOS A/S, Copenhagen K, Denmark; 49 Technical University of Denmark, Kgs., Lyngby, Denmark; 50 Suez Canal University, Ismailia, Egypt; 51 University of Sadat City, Sadat City, Egypt; 52 Ministry of Health, Environmental Microbiology, Tallinn, Estonia; 53 Addis Ababa University, Addis Ababa, Ethiopia; 54 University of Helsinki, Helsinki, Finland; 55 French Institute Search Pour L’exploitation De La Mer (Ifremer), Nantes, France; 56 Instituto Nacional de Investigaciσn en Salud Pϊblica-INSPI (CRNRAM), Galαpagos, Quito, Ecuador; 57 National Public Health Laboratories, Ministry of Health and Social Welfare, Kotu, Gambia; 58 National Center for Disease Control and Public Health, Tbilisi, Georgia; 59 Robert Koch Institute, Berlin, Germany; 60 Technische Universitδt Dresden, Institute of Hydrobiology, Dresden, Germany; 61 University for Development Studies, Tamale, Ghana; 62 University of Ghana, Accra, Ghana; 63 Kwame Nkrumah University of Science and Technology, Kumasi, PMB Ghana; 64 Council for Scientific and Industrial Research Water Research Institute, Accra, Ghana; 65 Veterinary Research Institute of Thessaloniki, Hellenic Agricultural Organisation-DEMETER, Thermi, Greece; 66 Athens Water Supply and Sewerage Company (EYDAP S.A.), Athens, Greece; 67 Universidad de San Carlos de Guatemala, Guatemala City, Guatemala; 68 Semmelweis University, Institute of Medical Microbiology, Budapest, Hungary; 69 University of Veterinary Medicine, Budapest, Hungary; 70 University of Iceland, Reykjavνk, Iceland; 71 Cochin University of Science and Technology, Cochin, India; 72 Kasturba Medical College, Manipal, India; 73 Apollo Diagnostics, Mangalore, India; 74 Shiraz University of Medical Sciences, Shiraz, Iran; 75 Shahid Beheshti University of Medical Sciences, Tehran, Iran; 76 National University of Ireland Galway, Galway, Ireland; 77 Ben Gurion University of the Negev and Ministry of Health, Beer-Sheva, Israel; 78 Istituto Zooprofilattico Sperimentale del Lazio e della Toscana, Rome, Italy; 79 CNR - Water Research Institute, Verbania, Italy; 80 National Institute of Infectious Diseases, Tokyo, Japan; 81 National Center of Expertise, Taldykorgan, Kazakhstan; 82 Mount Kenya University, Thika, Kenya; 83 Kenya Medical Research Institute, Nairobi, Kenya; 84 University of Prishtina “Hasan Prishtina” & National Institute of Public Health of Kosovo, Pristina, Kosovo; 85 Kuwait Institute for Scientific Research, Kuwait City, Kuwait; 86 Institute of Food Safety, Riga, Latvia; 87 American University of Beirut, Beirut, Lebanon; 88 Central Michigan University & Michigan Health Clinics, Saginaw, MI USA; 89 National Food and Veterinary Risk Assessment Institute, Vilnius, Lithuania; 90 Luxembourg Institute of Science and Technology, Belvaux, Luxembourg; 91 Institut Pasteur de Madagascar, Antananarivo, Madagascar; 92 University of Antananarivo, Centre d’Infectiologie Charles Mιrieux, Antananarivo, Madagascar; 93 University of Malawi, Blantyre, Malawi; 94 Malaysian Genomics Resource Centre Berhad, Kuala Lumpur, Malaysia; 95 AIMST University, COMBio, Kedah, Malaysia; 96 Water Services Corporation, Luqa, Malta; 97 Environmental Health Directorate, St. Venera, Malta; 98 University of Mauritius, Reduit, Mauritius; 99 Institute for Public Health Montenegro, Podgorica, Montenegro; 100 Institut Pasteur du Maroc, Casablanca, Morocco; 101 Centro de Investigaηγo em Saϊde de Manhiηa (CISM), Maputo, Mozambique; 102 Agriculture and Forestry University, Kathmandu, Nepal; 103 National Institute for Public, Health and the Environment (RIVM), Bilthoven, The Netherlands; 104 University of Otago, Dunedin, New Zealand; 105 University of Otago, Christchurch, New Zealand; 106 University of Central America, Managua, Nicaragua; 107 Universidad Nacional Autσnoma de Nicaragua-Leσn, Leσn, Nicaragua; 108 University of Ilorin, Ilorin, Nigeria; 109 University of Ibadan, Ibadan, Nigeria; 110 Norwegian Institute of Public Health, Oslo, Norway; 111 VEAS, Slemmestad, Norway; 112 University of Agriculture, Faisalabad, Pakistan; 113 Aga Khan University, Karachi, Pakistan; 114 Laboratorio Central de Salud Publica, Asuncion, Paraguay; 115 Instituto Nacional de Salud, Lima, Peru; 116 Universidad de Piura, Piura, Peru; 117 WHO Environmental and Occupational Health, Manila, Philippines; 118 Maynilad Water Services, Inc., Quezon City, Philippines; 119 National Veterinary Research Institute, Pulawy, Poland; 120 Universidade Catσlica Portuguesa, CBQF - Centro de Biotecnologia e Quνmica Fina - Laboratσrio Associado, Escola Superior de Biotecnologia, Porto, Portugal; 121 Aguas do Tejo Atlantico, Lisboa, Portugal; 122 Gwangju Institute of Science and Technology, Gwangju, Republic of Korea; 123 Korea Advanced Institute of Science and Technology, Daejeon, Republic of Korea; 124 Institute of Public Health of the Republic of North Macedonia, Skopje, Republic of North Macedonia; 125 State Medical and Pharmaceutical University, Chișinău, Republic of Moldova; 126 National Agency for Public Health, Chișinău, Republic of Moldova; 127 King Abdullah University of Science and Technology, Thuwal, Saudi Arabia; 128 University of Edinburgh, Edinburgh, Scotland UK; 129 Institut Pasteur de Dakar, Dakar, Senegal; 130 Institute of Veterinary Medicine of Serbia, Belgrade, Serbia; 131 Nanyang Technological University, Singapore, Singapore; 132 Public Health Authority of the Slovak Republic, Bratislava, Slovakia; 133 National Laboratory of Health, Environment and Food, Ljubljana, Slovenia; 134 Independent consultant, Johannesburg, South Africa; 135 Daspoort Waste Water Treatment Works, Pretoria, South Africa; 136 Korea Advanced Institute of Science and Technology, Daejeon, South Korea; 137 School of Veterinary Sciences, Lugo, Spain; 138 Labaqua, Santiago de Compostela, Spain; 139 IRTA, Centre de Recerca en Sanitat Animal (CReSA, IRTA-UAB), Campus de la Universitat Autonoma de Barcelona, Bellaterra, Spain; 140 University of Kelaniya, Ragama, Sri Lanka; 141 Medical Research Institute, Colombo, Sri Lanka; 142 Caribbean Public Health Agency, Catries, Saint Lucia; 143 The Sahlgrenska Academy at the University of Gothenburg, Gothenburg, Sweden; 144 Swedish University of Agricultural Sciences, Uppsala, Sweden; 145 Federal Food Safety and Veterinary Office (FSVO), Bern, Switzerland; 146 Ara Region Bern AG, Herrenschwanden, Switzerland; 147 Centers for Disease Control, Taipei, Taiwan; 148 Kilimanjaro Clinical Research Institute, Moshi, Tanzania; 149 Sokoine University of Agriculture, Morogoro, Tanzania; 150 Faculty of Science and Technology, Suratthani Rajabhat University, Surat Thani, Thailand; 151 Faculty of Public Health, Mahidol University, Bangkok, Thailand; 152 Faculty of Medicine Siriraj Hospital, Bangkok, Thailand; 153 National Institute for Public Health and the Environment (RIVM), Bilthoven, Netherlands; 154 National Institute of Hygiene, Lomι, Togo; 155 Agence de Mιdecine Prιventive, Dapaong, Togo; 156 Division of Integrated Surveillance of Health Emergencies and Response, Lomι, Togo; 157 Public Health Institution of Turkey, Ankara, Turkey; 158 Hatay Mustafa Kemal University, Hatay, Turkey; 159 Makerere University, Kampala, Uganda; 160 Abu Dhabi Public Health Center, Abu Dhai, United Arab Emirates; 161 Dubai municipality, WWTP Al Aweer, Dubai, UAE; 162 Rashid Hospital, Dubai, UAE; 163 Northumbrian Water, Northumbria House, Abbey Road, Pity Me, Durham, UK; 164 University of Exeter Medical School, Cornwall, UK; 165 Newcastle University, Newcastle upon Tyne, UK; 166 Brightwater Treatment Plant, Woodinville, WA USA; 167 Department of Environmental Sciences and Engineering, University of North Carolina at Chapel Hill, Chapel Hill, NC USA; 168 University of North Carolina, Chapel Hill, USA; 169 University of Washington, Seattle, WA USA; 170 Baylor University, Waco, USA; 171 Columbia Boulevard WWTP, Portland, USA; 172 Eastern Illinois University, Charleston, USA; 173 The Ohio State University, Columbus Ohio, USA; 174 Laboratorio Tecnolσgico del Uruguay, Montevideo, Uruguay; 175 Institute of Public Health in Ho Chi Minh City, Ho Chi Minh, Vietnam; 176 University of Zambia, Lusaka, Zambia

**Keywords:** Antimicrobial resistance, Microbial ecology, Metagenomics, Policy and public health in microbiology

Correction to: *Nature Communications* 10.1038/s41467-022-34312-7, published online 01 December 2022

In this article, the author name Antoinette Ngandjio was incorrectly written as Antoinette Ngandijo.

In this article, the affiliation details for Author Sara Cuadros-Orellana were incorrectly given as ‘Centro de Biotecnologνa de los Recursos Naturales, Facultad de Ciencias Agrarias y Forestales, Talca, Chile’ but should have been ‘Universidad Catolica del Maule, Centro de Biotecnología de los Recursos Naturales, Facultad de Ciencias Agrarias y Forestales, Talca, Chile’.

The original article has been corrected.

