# Supplementary material for: Genomic analysis of sewage from 101 countries reveals global landscape of antimicrobial resistance
Source: Nat Commun. 2022 Dec 1;13:7251. doi: 10.1038/s41467-022-34312-7 (PMC9715550; doi:10.1038/s41467-022-34312-7)
Supplement: Supplementary file 1 — Supplementary Info [file 41467_2022_34312_MOESM1_ESM.pdf]

Genomic analysis of sewage from 101 countries reveals global landscape of antimicrobial resistance

Supplementary Information

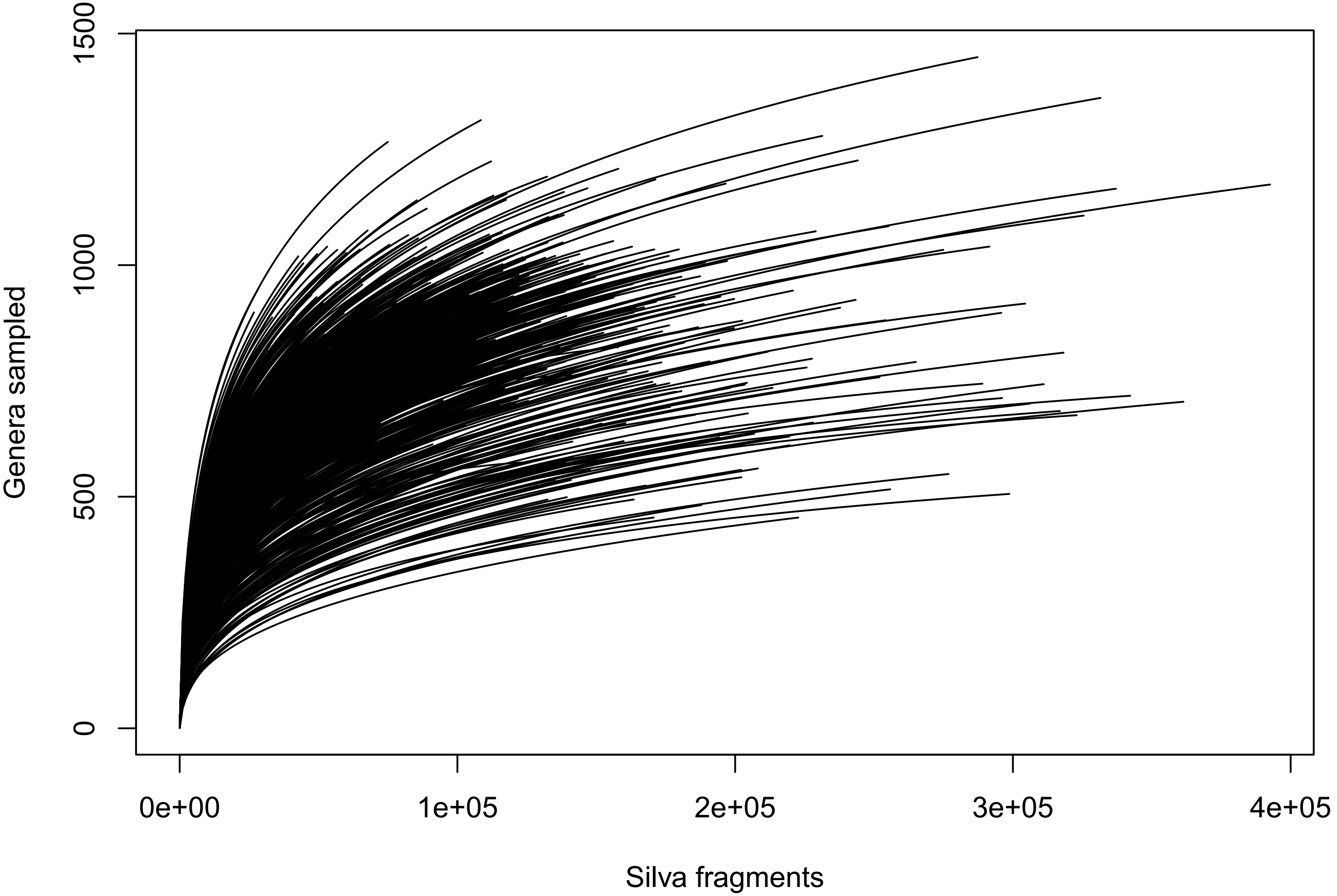

Supplementary Figure 1 Rarefaction curves of bacterial genera. Random sub-sampling without replacement was used to count the observed number of genera per sample at different sampling efforts. The step size along the x-axis was the median of the number of Silva fragments per sample, divided by a hundred, rounded to nearest integer. Source data are provided as a Source Data file.

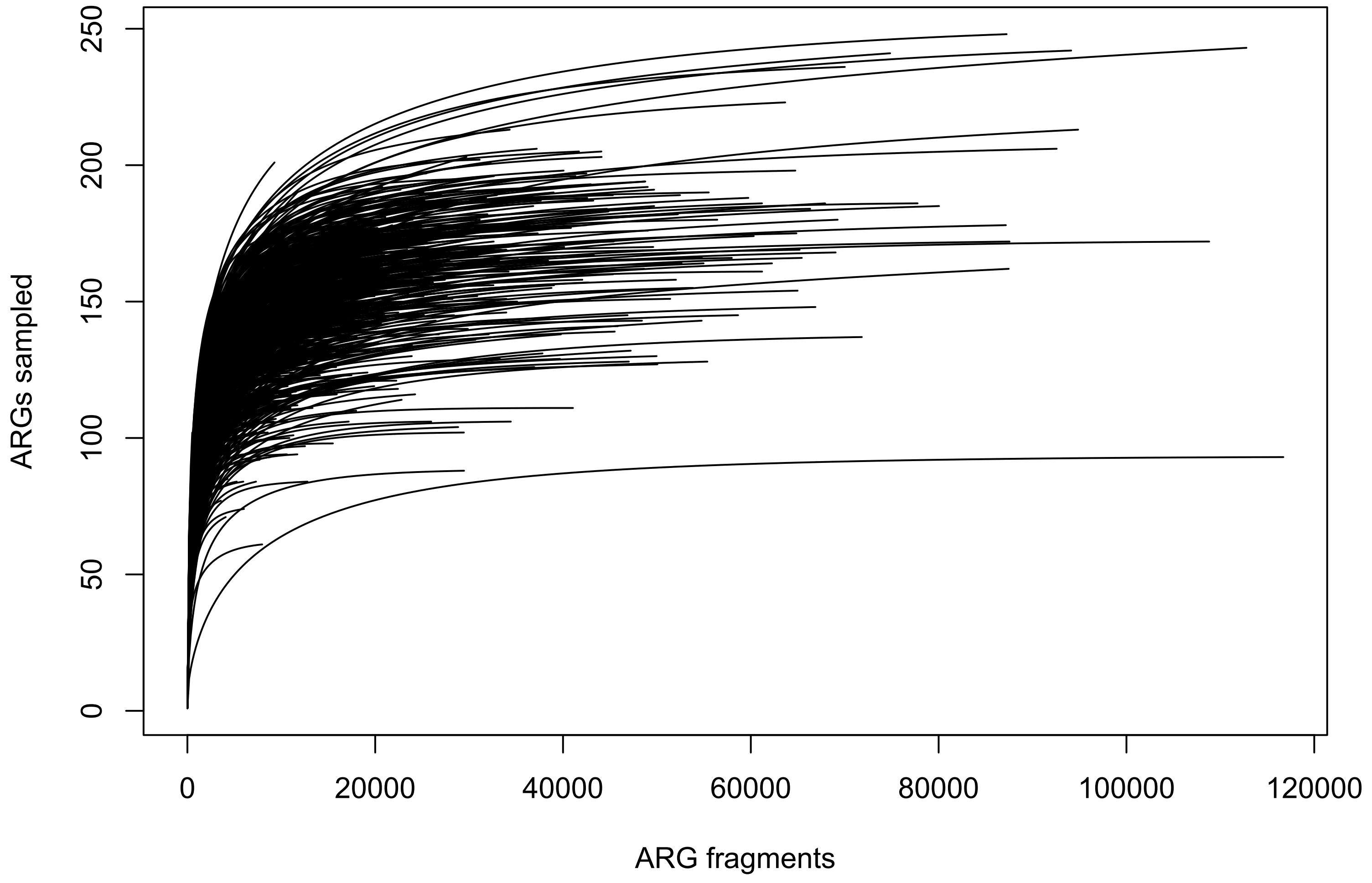

Supplementary Figure 2 Rarefaction curves of ARGs. Random sub-sampling without replacement was used to count the number of different ARGs (90% homology-reduced) at different sampling efforts. The step size along the x-axis was the median of the number of ARG fragments per sample, divided by a hundred, rounded to nearest integer. Source data are provided as a Source Data file.

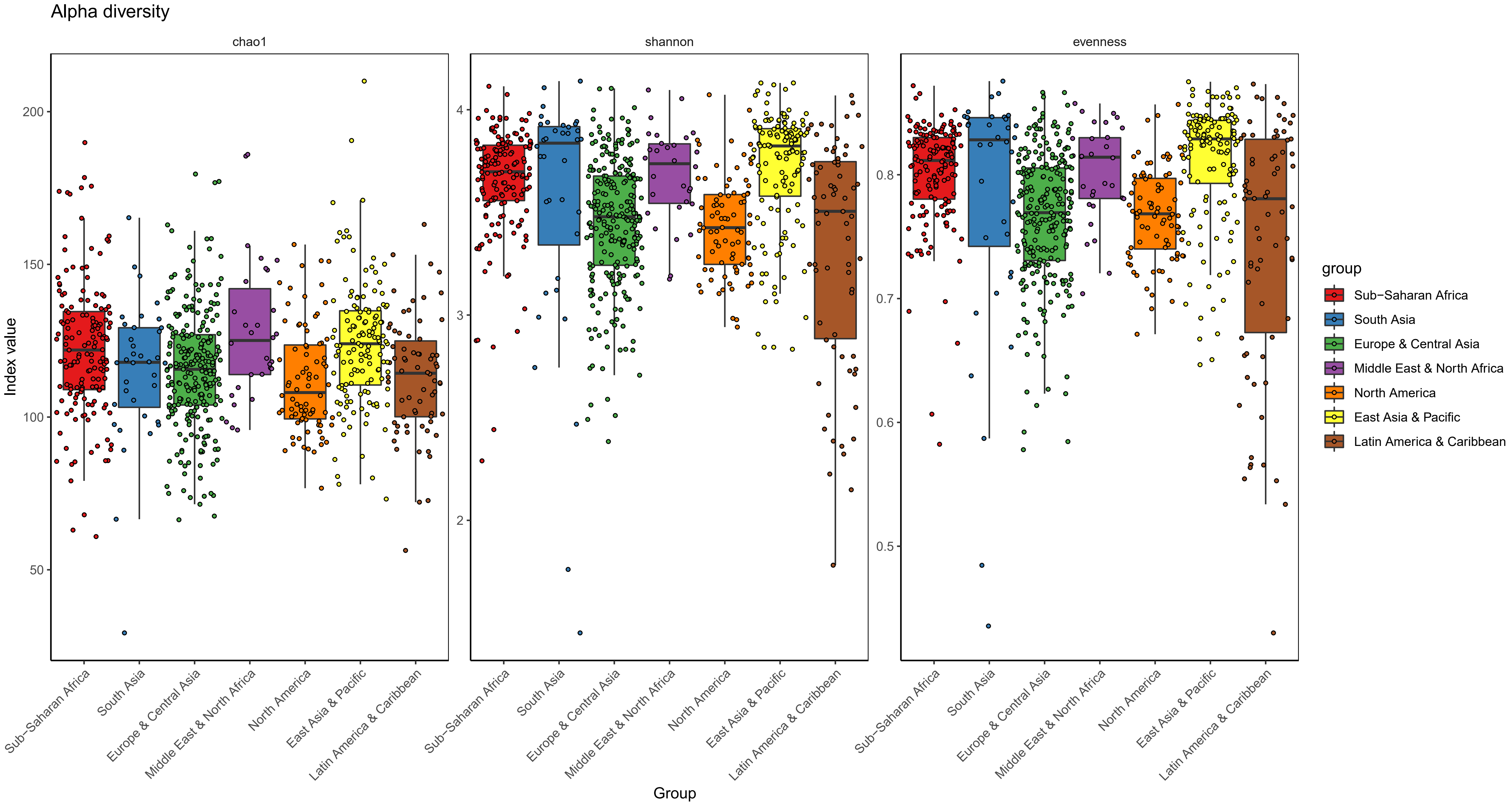

Supplementary Figure 3 ARG alpha diversity indeces separated by region. The Chao1 estimated richness (left), the Shannon diversity (middle) and Pileou's evenness. The individual dots represent the samples and are jittered sidewars to reduce overplotting. n=750 biologically independent samples (135, 244, 70, 30, 80, 36, 155 from left to right). For this analysis, samples with less than 1,000 ResFinder hits were excluded and the rest were rarified to the new lowest (1,035). The horizontal box lines represent the first quartile, the median and the third quartile. Whiskers denote the range of points within the firrst quartile − 1.5× the interquartile range and the third quartile + 1.5× the interquartile range. Source data are provided as a Source Data file.

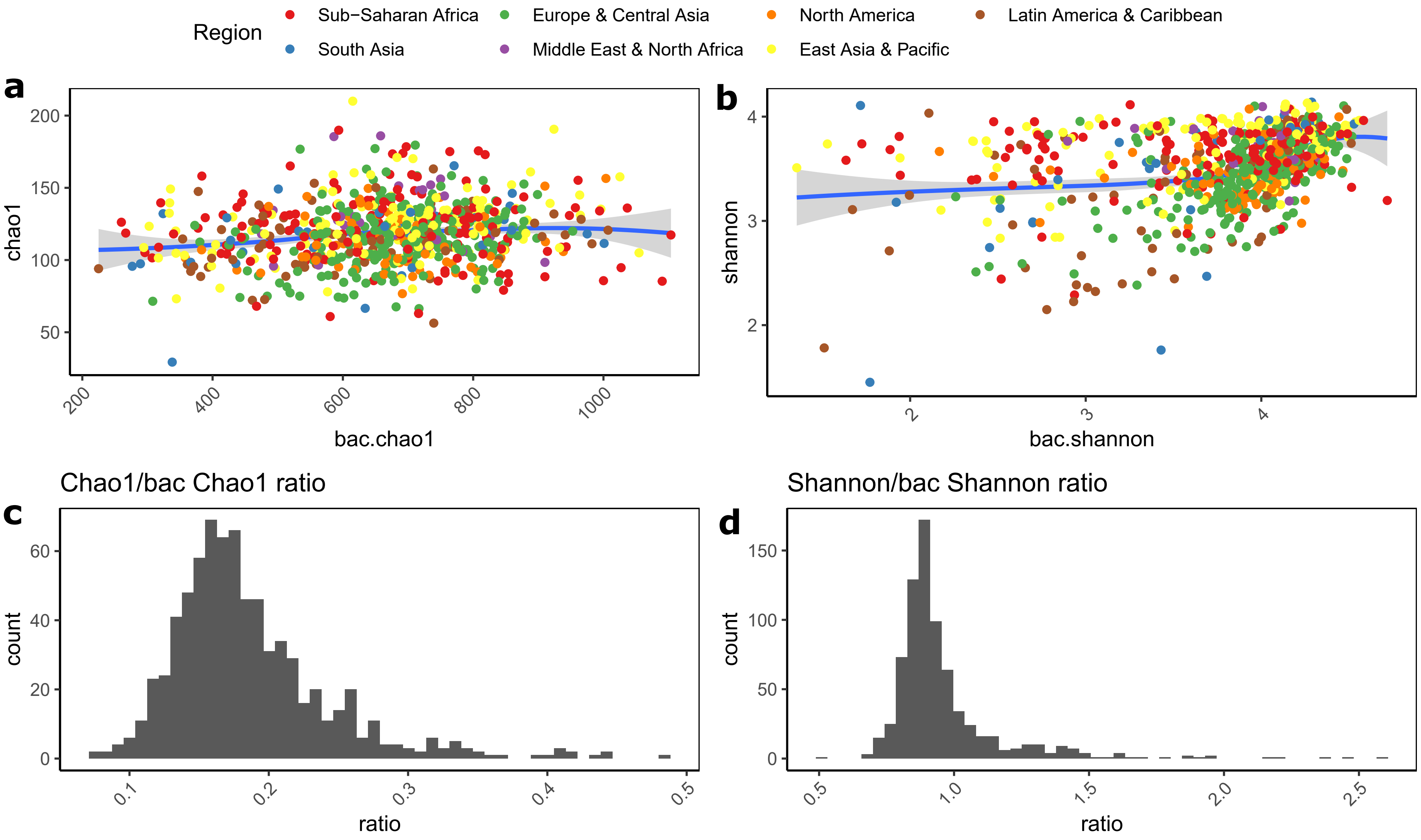

Supplementary Figure 4 Association between alpha diversity in the resistome and bacteriome. a: Chao1 richness association between bacterial genera and ARGs. b: Shannon diversity association between bacterial genera and ARGs. a/b: A local polynomial regression fit (loess) is drawn in with the 95% confidence intervals shaded around it. c: Histogram of resistome/bacteriome Chao1 richness ratio. d: Histogram of resistome/bacteriome Shannon diversity ratio. Source data are provided as a Source Data file.

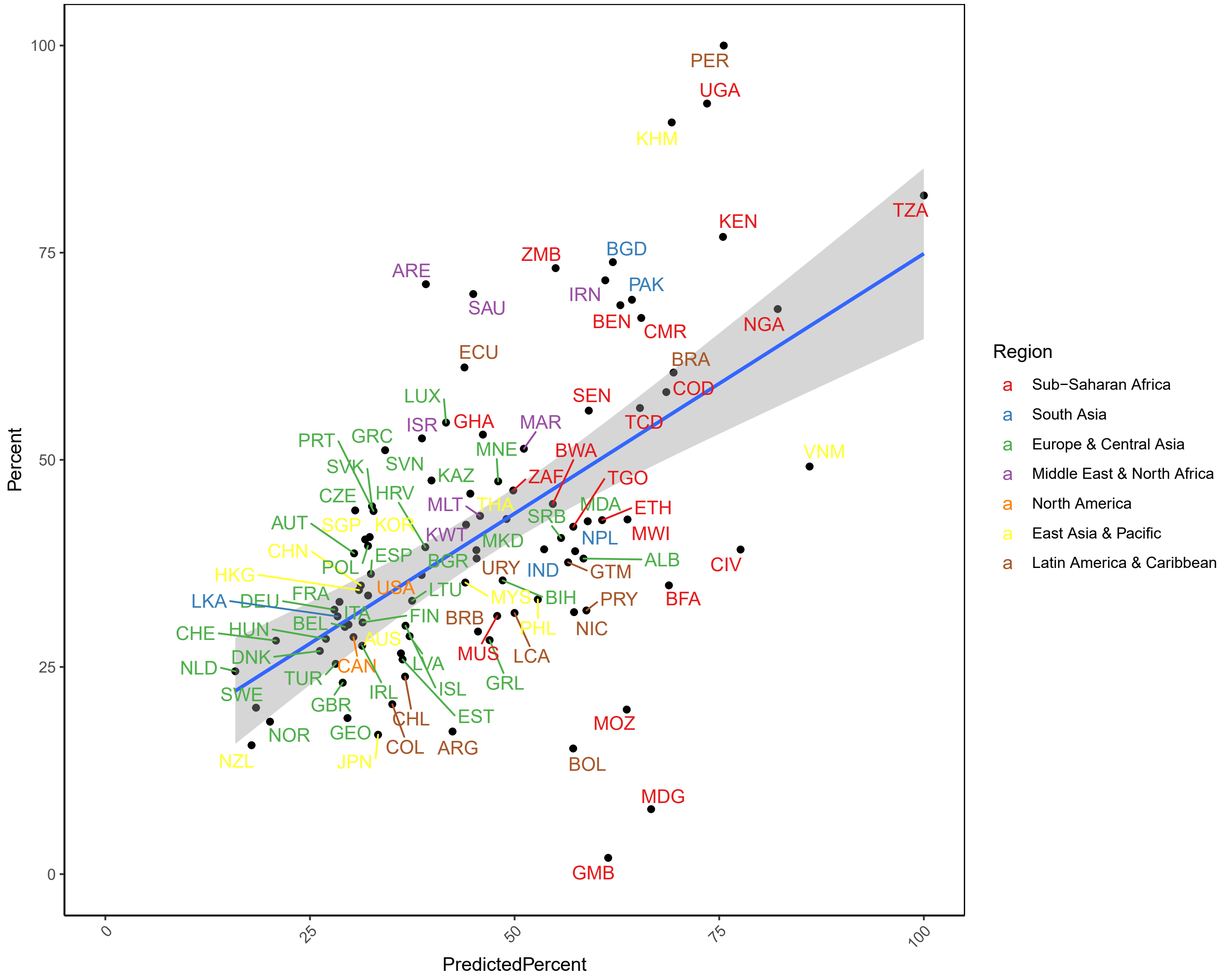

Supplementary Figure 5 Association between average measured ARG load per country and previous prediction. Each variable is relative to the maximum value within the variable (100%). The x-axis thus shows the predicted proportion based on the pilot study, while the y-axis shows the actually observed relative total ARG load. A linear regression line with 95% confidence intervals as shade is also drawn in. Source data are provided as a Source Data file.

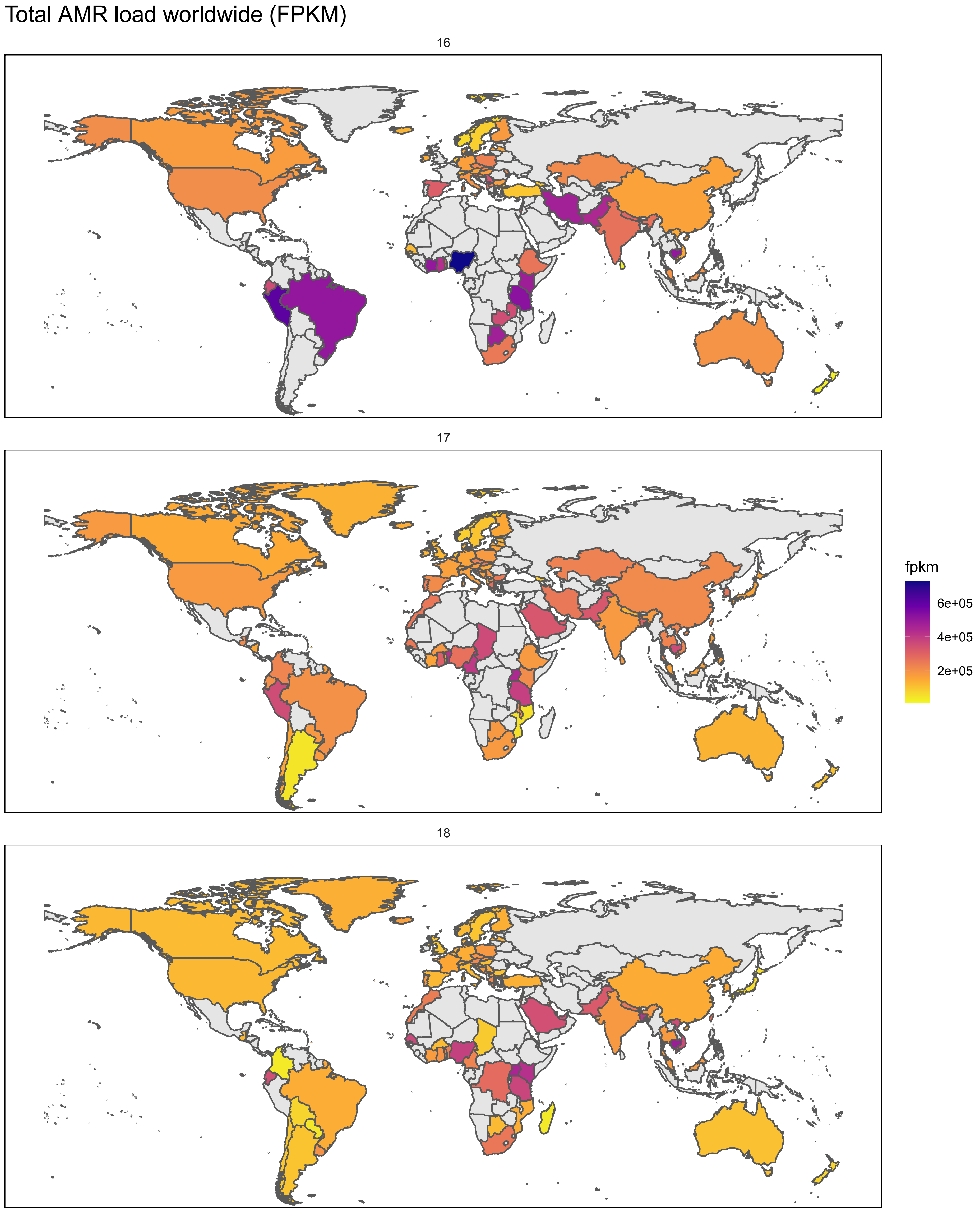

Supplementary Figure 6 World-wide total AMR load through time. Total AMR loads (FPKM) were stratified by year (2016-2018) and country and visualized as a choropleth. Note that 2016 only had a single summer sampling round, versus two seasons sampled in the two latter years. Source data are provided as a Source Data file.

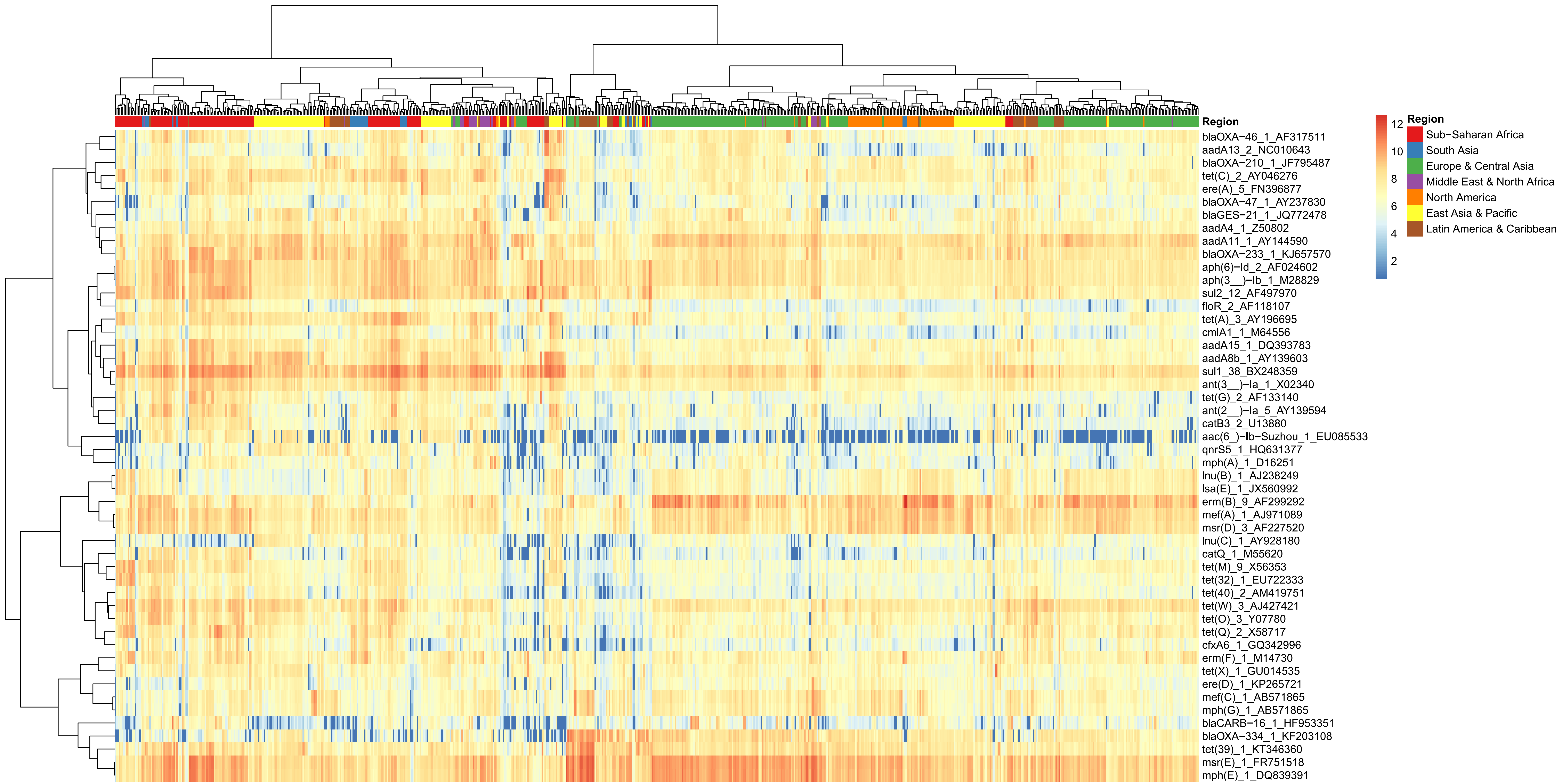

Supplementary Figure 7 Heatmap of most abundant ARGs. Clustered resistome heatmap showing the relative (additive log ratio) abundance of the 50 most abundant ARGs in the dataset. Hierarchical clustering of sample columns is based on all ARGs, not just the visualized ones. Dark blue indicates no assigned reads in sample-ARG combination. Source data are provided as a Source Data file.

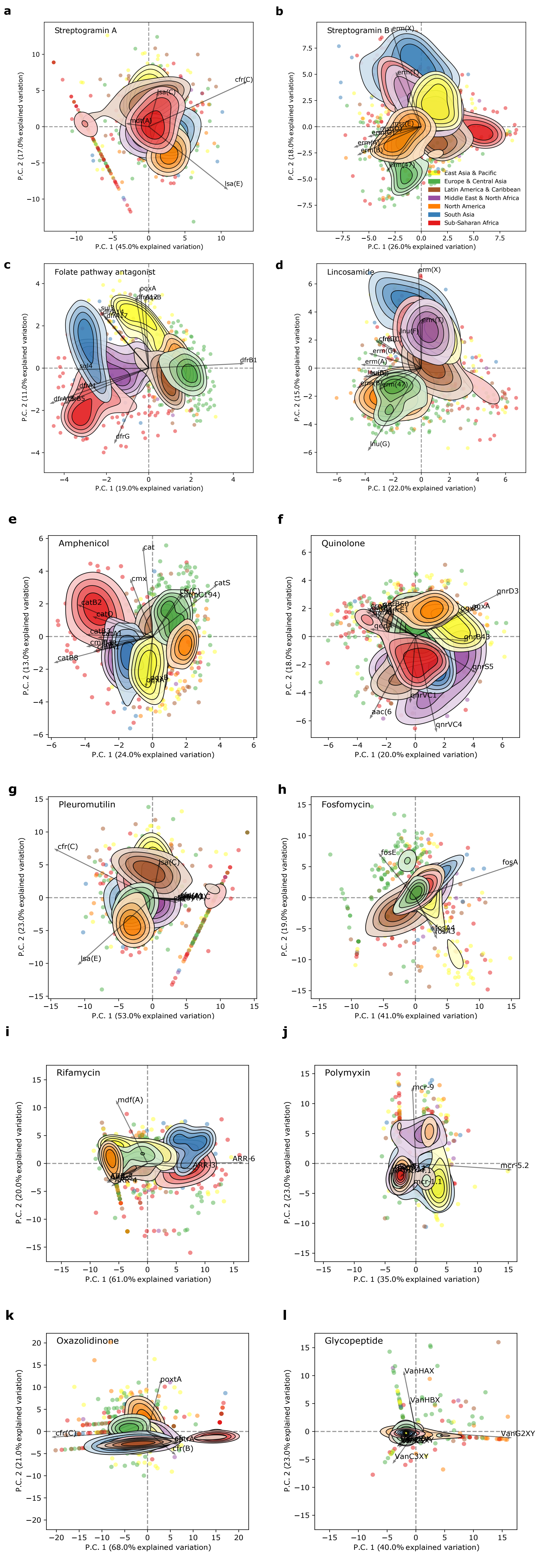

Supplementary Figure 8 PCA ordinations of class separated ARG abundances. Contour density plots show the highest density areas of samples, stratified by World Bank region. a: Streptogramin A, b: Streptogramin B, c: Folate pathway antagonist, d: Lincosamide, e: Amphenicol, f: Quinolone, g: Pleuromutilin, h: Fosfomycin, i: Rifamycin, j: Polymyxin, k: Oxazolidinone, l: Glycopeptide. See Figure 3 for remaining drug classes and see panel b for the legend. Each panel represent genes encoding resistance to a class of drugs (upper left corner). Source data are provided as a Source Data file.

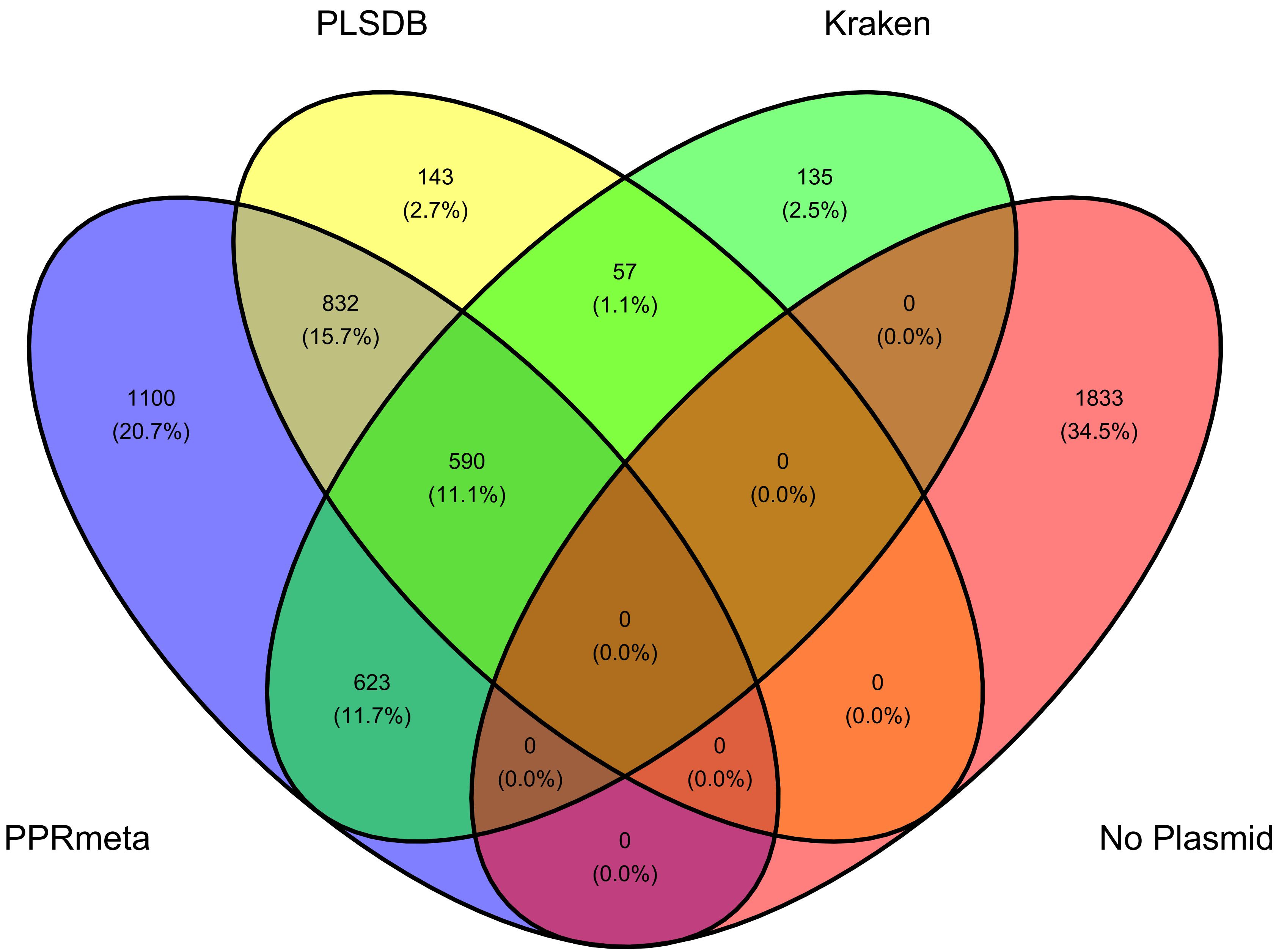

Supplementary Figure 9 Venn Diagram for Plasmid classification. Three different plasmid classification tools were used on contigs with ResFinder ARGs containing flanking sequence on either side. Contigs with at least two methods suggesting plasmid origin were annotated as such. Source data are provided as a Source Data file.

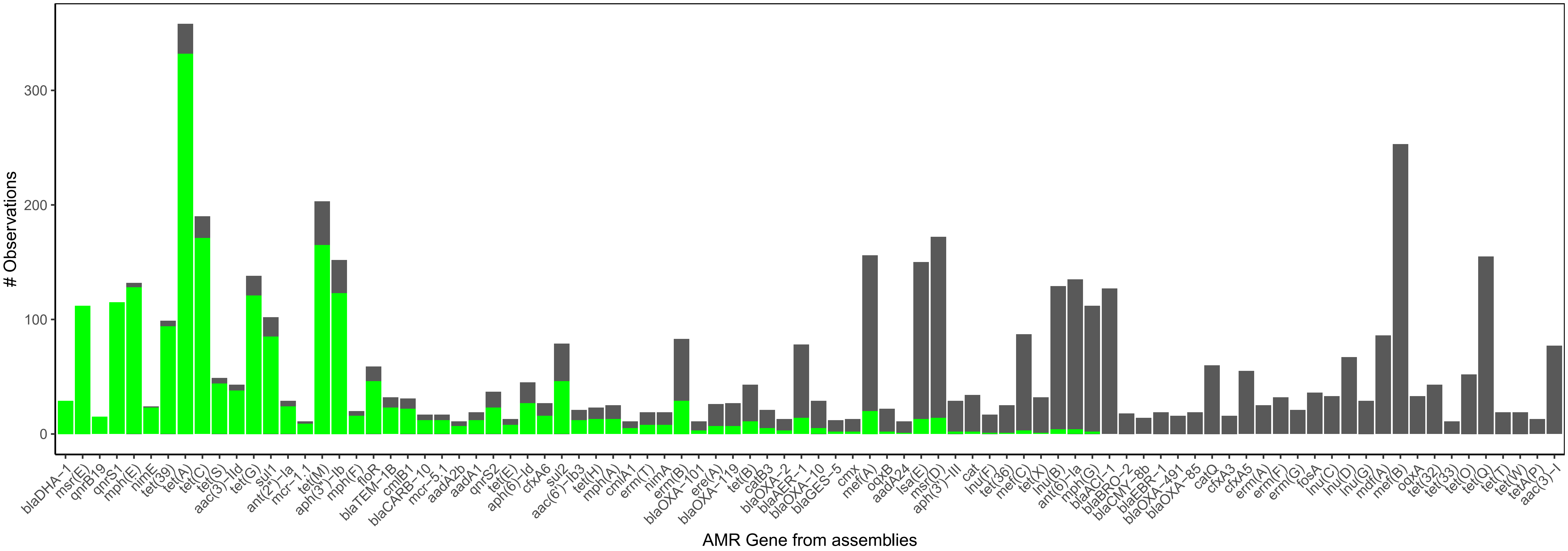

Supplementary Figure 10 ARGs with flanks identified in the metagenomic assemblies. Height of each bar reflect the number of times a flanked ARG was retrieved across all samples. Height of the green bar shows the subset that was classified as plasmidic. Source data are provided as a Source Data file.

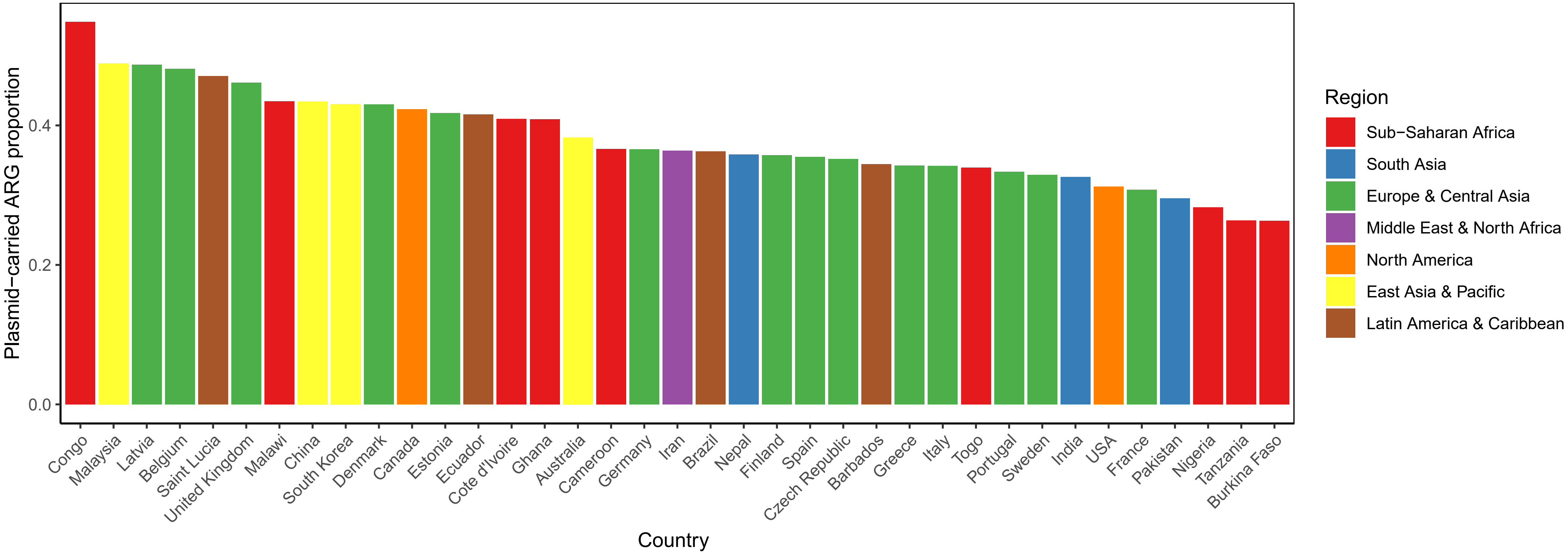

Supplementary Figure 11 Proportion of plasmid-associated ARGs per country. Only countries with more than 50 flanked ARGs were used for calculating shown proportions. Source data are provided as a Source Data file.

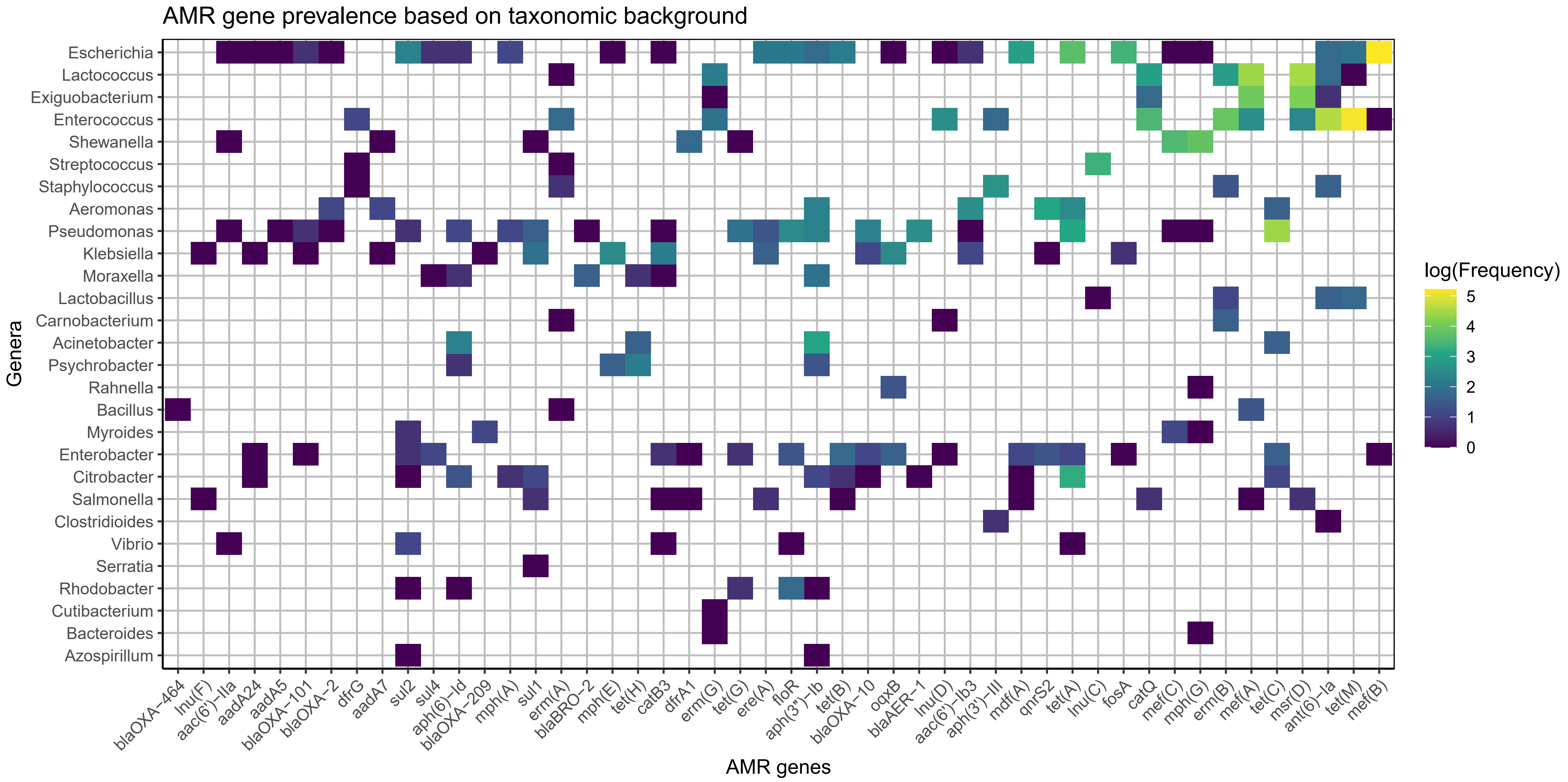

Supplementary Figure 12 Variation in taxonomic assignment per ARG. Heatmap showing the (log) times an individual flanked non-plasmid ARG was found on a metagenomic scaffold assigned to a genus. Only the intersection between ARGs assigned to >3 taxa and taxa with >3 ARGs assigned is plotted. Source data are provided as a Source Data file.

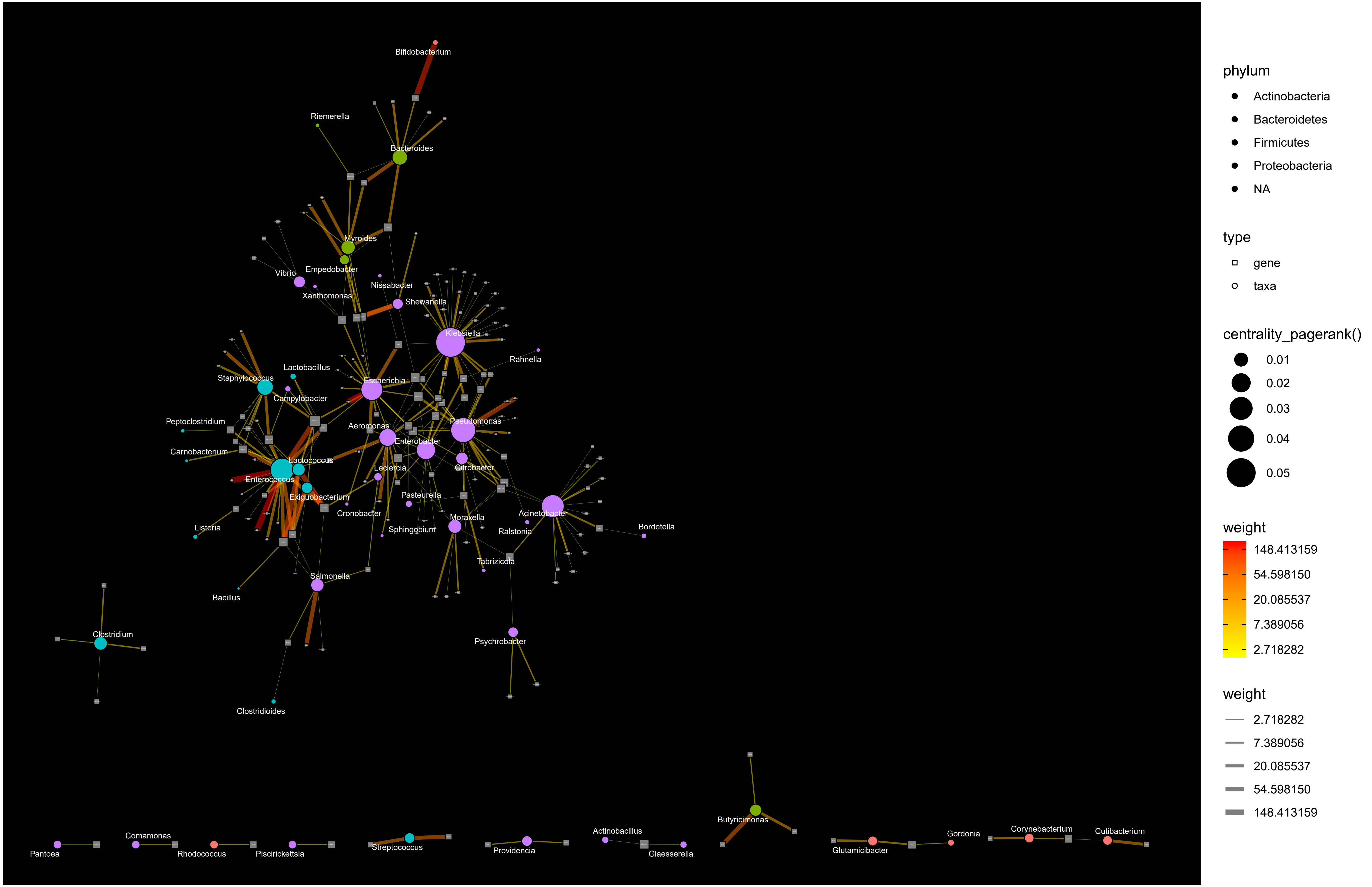

Supplementary Figure 13 Gene-sharing network between bacterial genera. The 'backbone' algorithm was used to compute the graph layout. Edges link ARGs to the genera which their contigs were taxonomically assigned to. Only flanked, non-plasmidic contigs were used. Color and thickness of edges denote the number of observed taxa-gene co-occurrences. Nodes are ARGs and genera which are visualized as grey boxes and colored circles respectively. Node size denote the centrality of the individual nodes to the overall network. Source data are provided as a Source Data file.

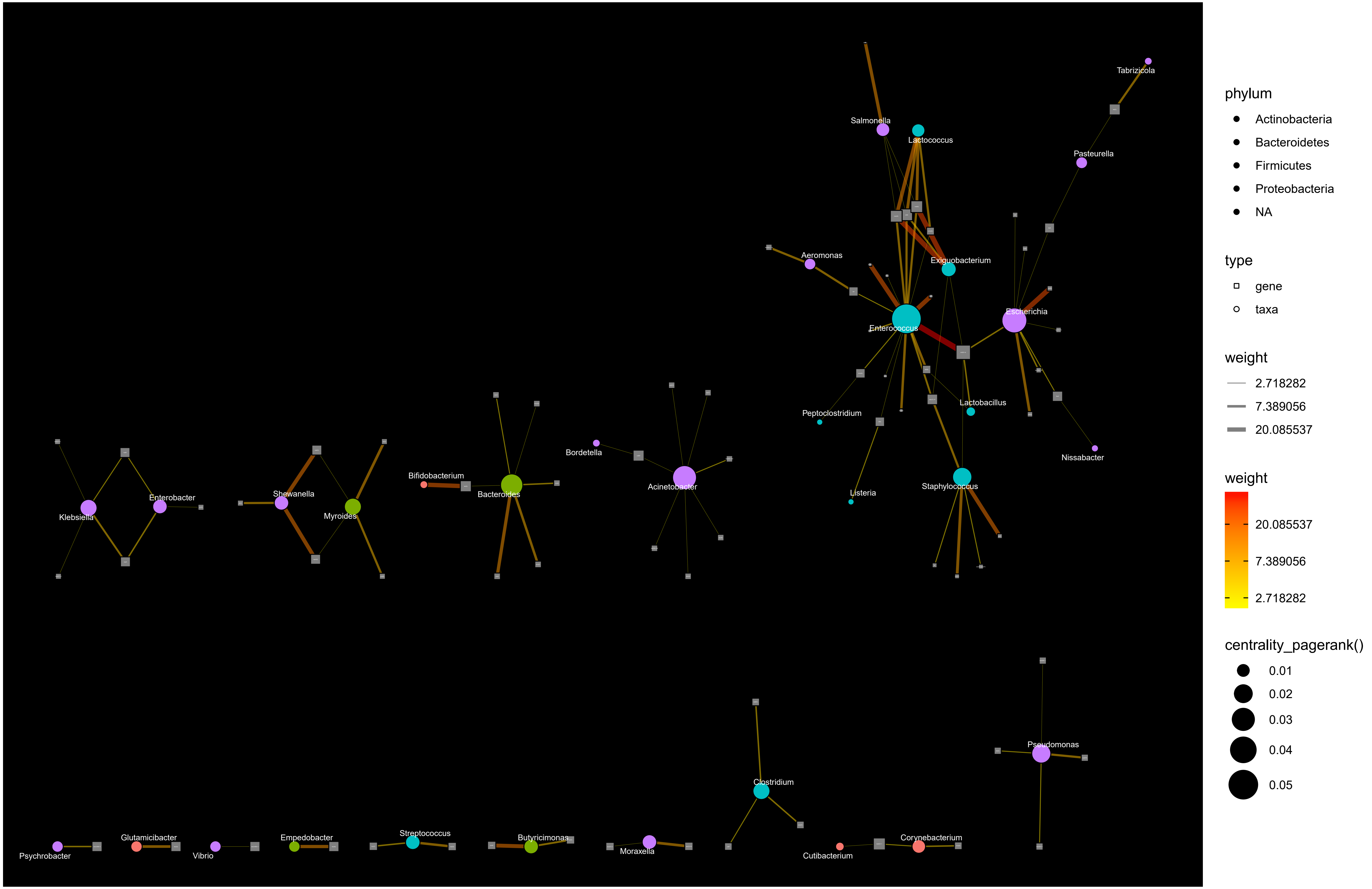

Supplementary Figure 14 Gene-sharing network between bacterial genera in Sub-Saharan Africa. The 'backbone' algorithm was used to compute the graph layout. Edges link ARGs to the genera which their contigs were taxonomically assigned to. Only flanked, non-plasmidic contigs from Sub-Saharan Africa were used. Color and thickness of edges denote the number of observed taxa-gene co-occurrences. Nodes are ARGs and genera which are visualized as grey boxes and colored circles respectively. Node size denote the centrality of the individual nodes to the overall network. Source data are provided as a Source Data file.

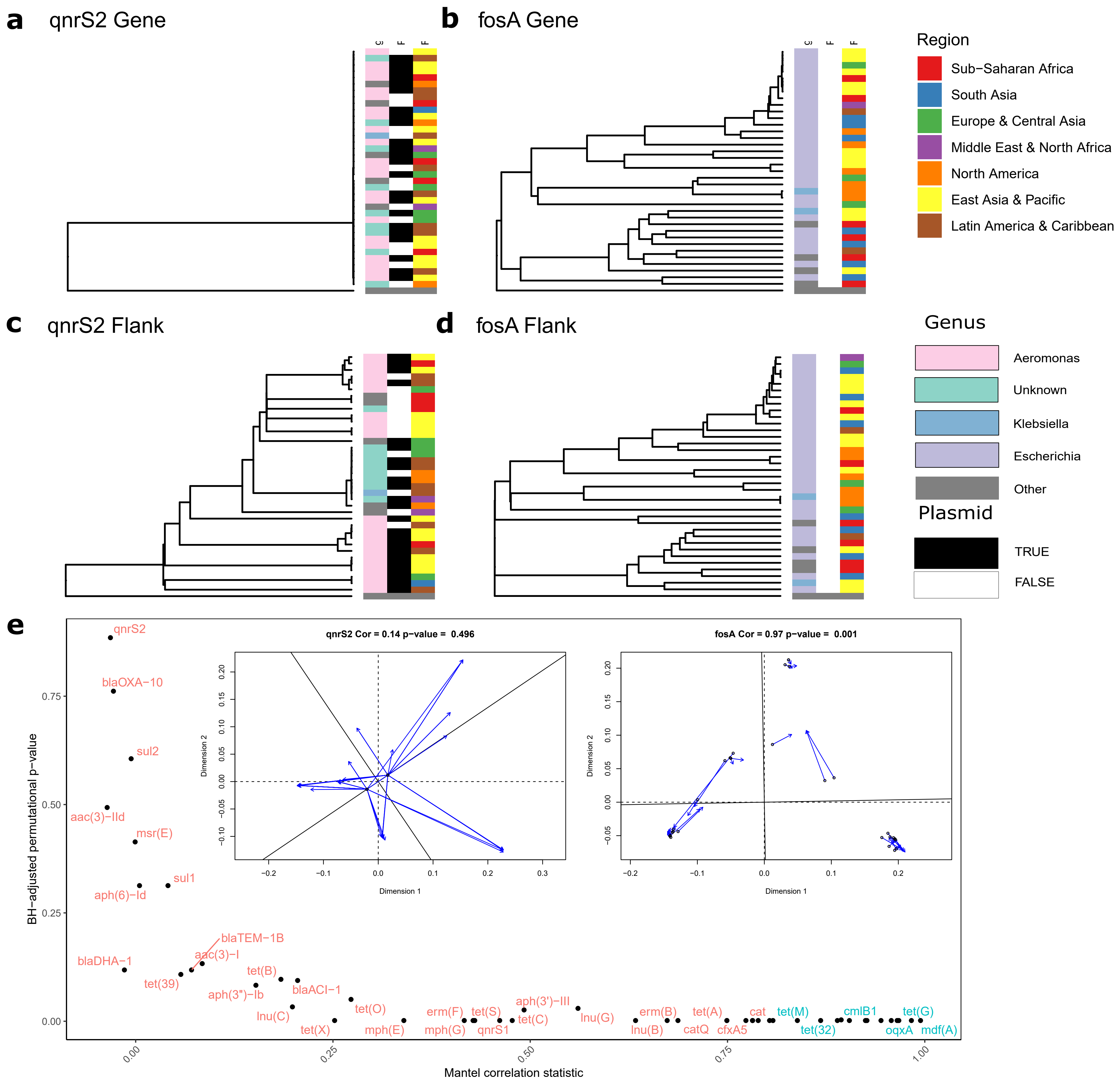

Supplementary Figure 15 ARG-Flank association strength varies drastically between ARGs. (a) Clustering of qnrS2 gene sequences: an example of a gene with low variation and no correlation to its 1 kb flanks. (b) Clustering of fosA gene sequences: an example of a gene with deep branches that correlate to its flanking sequence. (c) Clustering of flanking sequences for qnrS2. (d) Clustering of flanking sequences for fosA. The annotation bars (left-to-right) in a-d signal different genera, plasmid status and WHO regions. (e) Genes with high Mantel correlation to their flanking sequence (>0.8) and low multiple-adjusted p-values (<0.05) are colored blue in the scatter plothave blue text labels. The permutational Mantel test statistic is the proportion of times a randomly permutated dissimilarity matrix increases the Pearson correlation coefficient to the other (static) dissimilarity matrix. P-values were then adjusted for the false discovery rate using the Benjamini Hochberg approach. Procrustes plots of the non-significant (red) qnrS2 and significant (blue) fosA are embedded for visualization. For ARGs like fosA, dissimilarity matrices built on ARGs and flanks result in similar clustering and high Procrustes correlations. Source data are provided as a Source Data file.

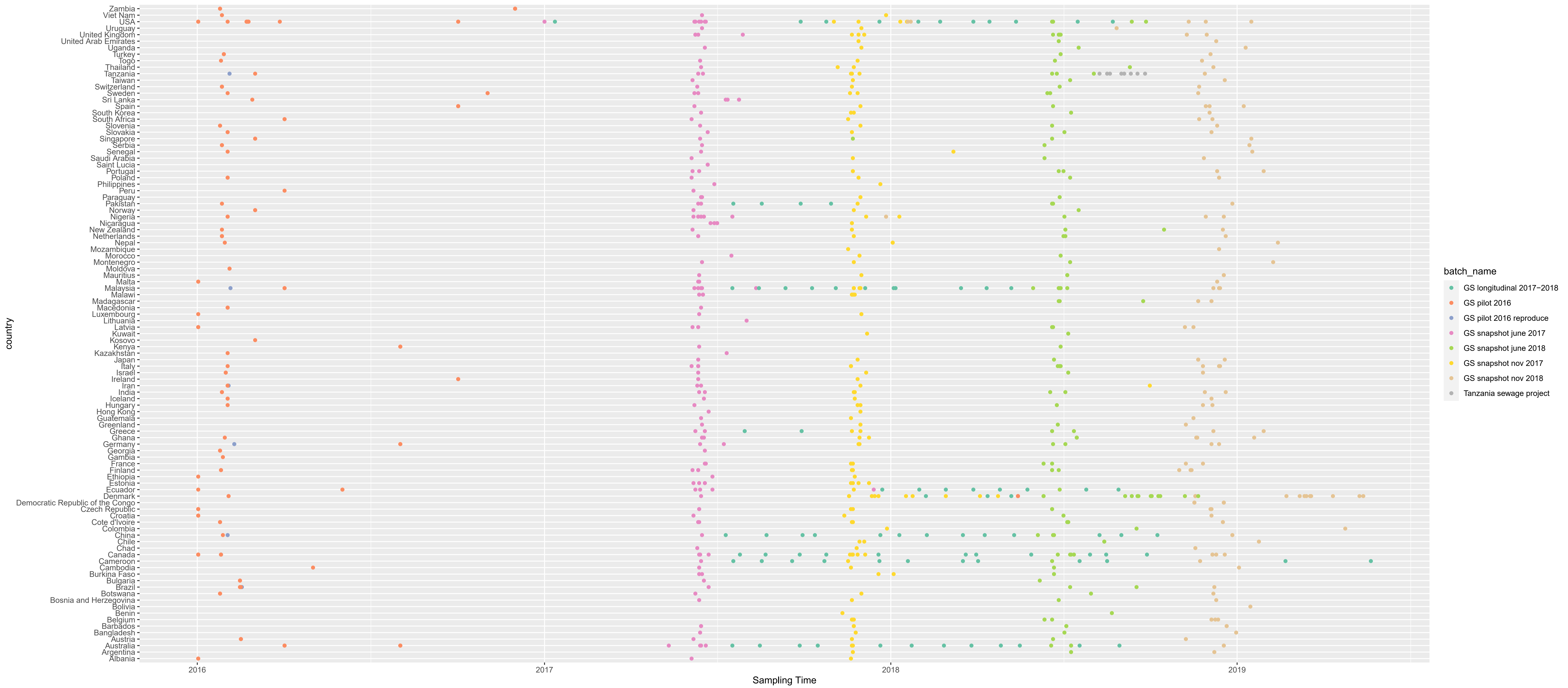

Supplementary Figure 16 Sampling overview. Samples included in the study, shown as a function of sampling time (x-axis) and country of origin (y-axis). Colors denote sample sub-project. Source data are provided in Supplementary Data 1.

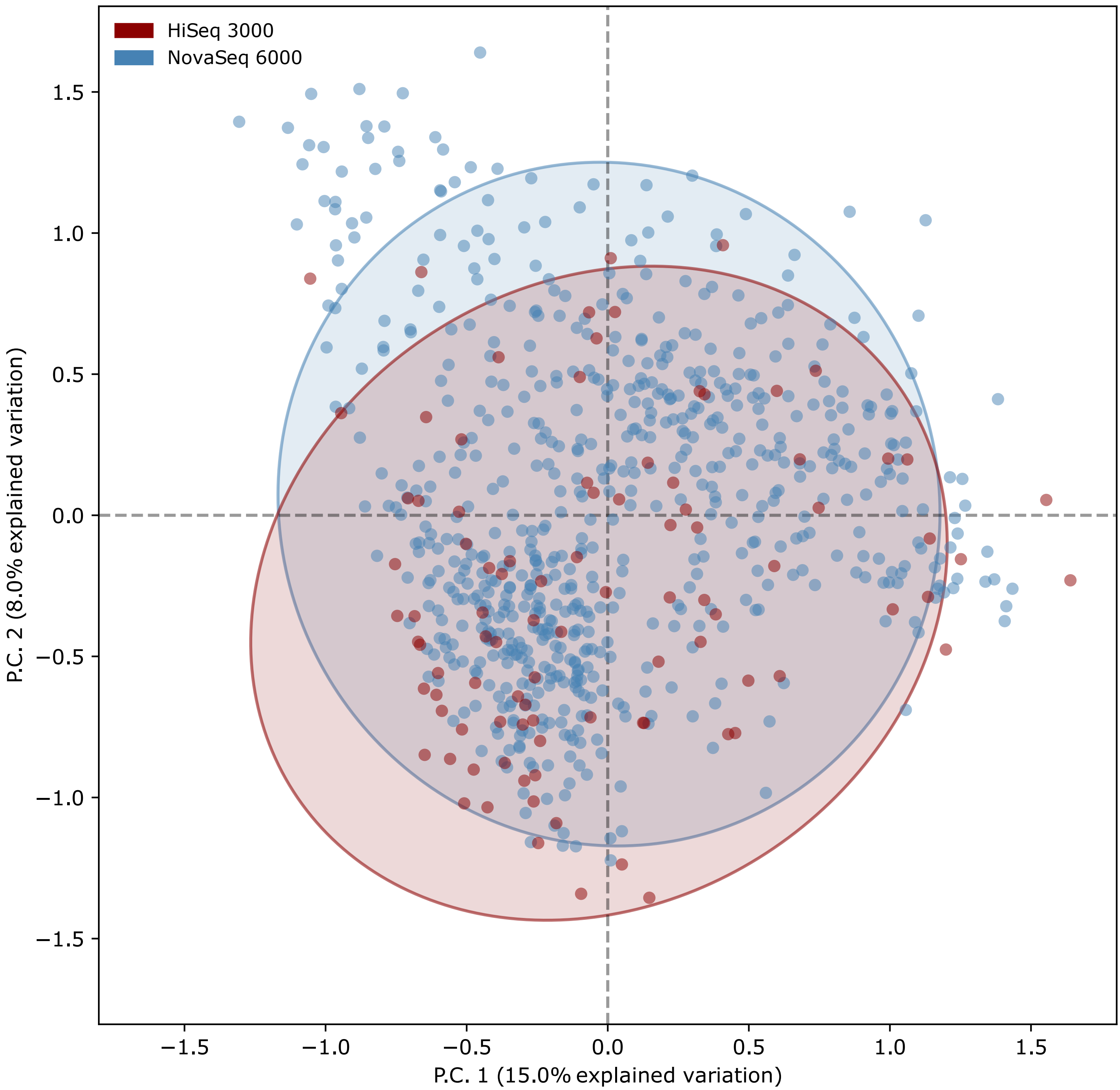

Supplementary Figure 17 Lack of systematic effect of sequencing instrument on resulting resistome composition. Principal components analysis was used on the centered and scaled abundance matrix (CLR) of resistance gene clusters (90% identity) across the sample set. The 90% covariance ellipses are drawn in for the two separate sequencing instruments. Source data are provided as a Source Data file.

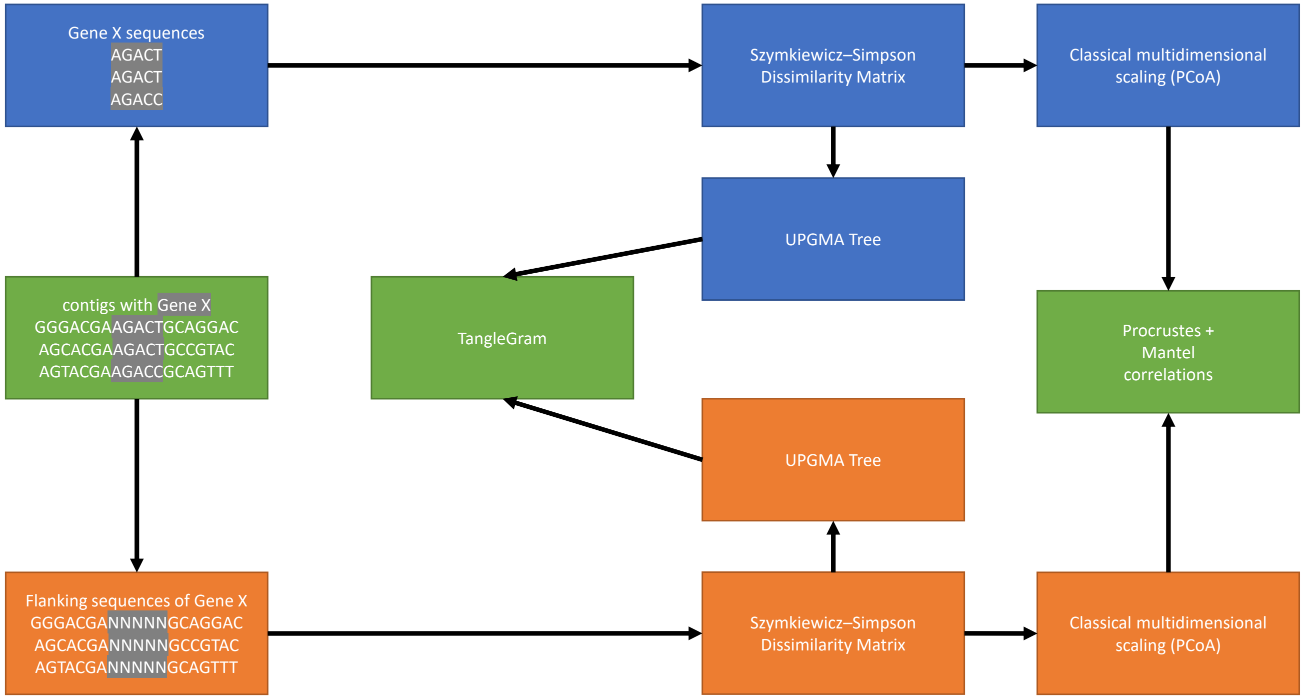

Supplementary Figure 18 Workflow for comparison of each gene to its flanking sequence. The analysis was carried out separately for alleles of each identified ARG. Blue boxes are specific to the gene sequence, orange boxes are specific to the flanking sequence and green boxes are relevant to both.

Supplementary Notes

Plasmid classification for scaffolds

We BLAST'ed scaffolds against known plasmids. Of the 5,313 contigs containing the 5,814 double-flanked ARGs, a total of 1,622 had positive PLSDB BLAST (90% query identity and coverage) hits, corresponding to 30.5%. Using a custom plasmid-amended Kraken2 database, we found 1,405 contigs were assigned to plasmids, rather than chromosomes. Lastly, using the Neural Net-based PPR-Meta, 3,468 contigs corresponding to 59.6% had plasmid as their most probable source. 1,833 (34.5%) of scaffolds had no indication of plasmid origin and only 590 (11.1%) had plasmid agreement between all three methods. 2,102 (39.6%) scaffolds had two or more methods suggesting plasmid origin, a threshold we used for considering scaffolds plasmidic.

ARG and flank clustering and gene synteny

Summary of the genomic localization of resistance genes, phylogeny of flanking regions and associations to bacterial genera and geographic regions. This chapter is a description of the results visualized in supplement 1 & 2.

tet(A) has previously been found widespread among mainly Gram-negative bacterial species[1] which we also observe in our data. Most *tet*(A) genes as well as their flanking regions were very similar and seemingly randomly distributed across bacterial taxa and regions. Gene synteny analyses also suggested a in general highly conserved flanking gene organization. Despite the limited variation of *tet*(A), three clusters of gene variants were identified. Two with the same gene synteny, with the most commonly found observed in all regions, but the rarer seemingly associated with Asia and Africa. In three samples, all from Africa, a very different variant was observed, in a completely different gene synteny and very different flanks.

mef(B) was, as expected, primarily associated with *Escherichia coli* [2], but with a few findings in *Enterococcus* and *Enterobacter* and mostly share very similar genetic backgrounds[2]. In a separate genetic context, and with a separate *mef*(B) gene variant, we found it in several genera of Proteobacteria.

tet(M) was mainly associated with *Enterococcus,* but a few findings in other cocci and in most sites, we observed similar genes and highly similar genetic contexts. A separate cluster of more rare variants were found in a different gene synteny more commonly associated with *Lactococcus* and the variation of *tet*(M) and the flanking regions were seemingly a bit larger in samples from Africa/Asia/South America.

tet(C) genes were almost all identical. However, when looking at the flanking regions, 6-7 different clusters were identified; two were mostly associated with *Pseudomona*s and were predominant in Europe and North America, and four were predominant in other unclassified genera frequently in Asia and Africa. Gene synteny analyses suggested a similar organization of the immediate flanking genes, whereas a cluster specific organization was observed longer away from the *tet*(C) gene.

Our analyses confirm the genetic co-location of *mef*(A) and *msr*(D)[3], and show relatively large genetic variation within the genes, as well as the flanking regions, as shown by both kmer- and gene synteny analyses. Our data suggest that this locus has followed different evolutionary trajectories; perhaps it has been mobilized as different variants multiple times, but also occasionally undergone gene synteny re-organization or having other genes jumping in close to them. The two genes were primarily found in *Exiguobacterium* and *Lactococcus* in all regions of the World and one gene and flank variant was associated with *Lactococcus* mainly in Europe, while another primarily with *Exiguobacterium* in Asia and Africa and 4-5 clusters among *Lactococcus, Enterococcus* and *Exiguobacterium* in all regions of the world.

Two different genetic variants of *tet*(Q) were observed on two different clusters of flanking regions and almost exclusively in *Bifidobacterium*, with a few instances of *Bacteroides*. Gene synteny analyses showed that this difference in clustering was caused by difference in the upstream region. Studies based on cultured bacteria have previously identified *tet*(Q) in *Bacteroides*, *Porphyromonas* and *Prevotella*, as well as *Bifidobacterium*[4–6].

aph(3'')-Ib (also known as *strA*) was found widely distributed across multiple bacterial genera mainly belonging to Proteobacteria and across all regions. The genes were in most cases almost identical and the immediate flanking regions with limited variation. A limited number of variants were observed in samples from Asia, Sub-Saharan Africa and Middle East. *aph*(3'')-Ib is most often found co-located with *aph*(6')-Id (also known

as *strB*). In our study, this gene retained its flank more infrequently and showed some gene synteny changes downstream, but a similar distribution across bacterial genera and regions. These genes have been observed very widespread among several bacterial species in human, animal and environmental environments[7].

lnu(B) and *lsa*(E) are normally considered to be genetically co-located and observed in streptococci and staphylococci[8,9]. We exclusively assigned contigs with these genes to *Enterococcus* and our data suggest the occurrence of at least four different genetic variants localized in a varying genetic context and some regional clustering for the different variants. Two clusters contained almost all samples from North America and one cluster most samples from Africa and Asia, suggesting regional specific dispersal of the variants.

tet(G) has been reported from multiple Gram-negative species[1]. For most of the samples we did not get genus-specific assignment, with the majority assigned to Gamma- and Alpha-proteobacteria. *Pseudomonas*, *Klebsiella* and *Tabriziola* carrying the gene mainly formed separate clusters based on gene-variation, flanking variation and gene synteny and also seemed to cluster regionally.

ant(6)-Ia (also known as *aadE*) has previously been detected in various Gram-positive species and *Campylobacter*[10–13] and was in our study mainly associated with *Enterococcus*, but in some cases also *Lactococcus* and *Staphylococcus*. A few clusters of highly similar genes in similar backgrounds, mainly differing in the gene synteny, were observed. Also, there was a smaller cluster with a completely different gene synteny including *tet(*M), which was mostly *Escherichia*-assigned and exclusive to samples from Africa, Asia and Latin America.

We also confirmed the tandem arrangement of the macrolide efflux and phosphotransferase genes *msr*(E) and *mph*(E), respectively. We did not find multiple variants of these genes, except for a single Belgian variant not co-located with *msr*(E) and completely different neighbor genes. For a minority, some variation of the flanking gene organization was observed. They were in our study assigned mainly to *Acinetobacter* and *Klebsiella*. While originally discovered in *Pasteurella*[14], these genes have previously been detected also in *Acinetobacter* and *Vibrio*[15,16].

We assigned a few of the $bla_{\text{ACI-1}}$ contigs to the Actinobacteria genera of *Corynebacterium* and *Cutibacterium*, but the majority had no good classification.

bla$_{ACI-1}$ was originally identified in *Acidaminococcus fermentans*[17] and even though this was originally suggested to have been acquired by horizontal transfer, our findings of this gene being highly similar and observed in a similar genetic context, suggest that in the sewage environment, it is probably stably embedded in the chromosome of an uncharacterized Actinobacteria. The gene synteny did however, differ.

*qnr*S1 has a wide geographic distribution and has mainly been observed in *Klebsiella*, *Escherichia* and *Salmonella*[18]. In our study the *qnrS1* gene was found widely distributed in all regions, with identical genes and very similar flanking regions and mainly in *Klebsiella* and a few cases assigned to *Escherichia*. Some variation in gene synteny was observed and it is noteworthy that the most variation was observed among samples from Asia and Africa.

*qnr*S2 has, in addition to the three species above, also be reported from *Aeromonas*[18] and in our data it was mainly observed on contigs assigned to *Aeromonas*, but also to *Klebsiella* and *Enterobacter* in multiple cases. All genes were nearly identical, but the flanking regions varied a lot also in gene synteny, and clustered both according to bacterial taxonomy and region.

The *mef*(C)-*mph*(G) genes are considered tandem genes and have been found widely distributed among marine and waste-water bacteria[19,20]. In our study, five different highly variable clusters of gene variants were observed; two mainly on contigs assigned to *Shewanella* and three assigned to an undetermined *Gammaproteobacteria.* A clear regional separation was observed, with the *Shewanella* clusters observed in all regions. One of the other Gammaproteobacteria clusters was predominantly in North American samples, and the last cluster was predominantly in European samples. The variation in the flanking regions followed the gene variants, but with much more variability. Part of this high variability was due to differences in gene synteny. A possible explanation for the almost smooth spectrum of variability in the flanks, could be the genes remaining within their major geographic regions for a very long time, accumulating mutations in parallel and not outcompeting each other, due to immobility.

The *sul*1 gene is considered an integrated part of the class 1 integrons[21] and has been found widespread in multiple Gram-negative bacterial species. Two highly similar gene variants were observed often flanked by other resistance genes. A taxonomic

and geographical widespread occurrence was also observed in our study, but we also observed that the flanking regions were more similar in East Asia and Pacific, suggesting more recent, clonal dissemination.

The only tetracycline resistance gene normally considered unique to environmental bacteria is *tet*(39), which has previously been identified in *Acinetobacter*[1]. We also observed this gene mainly in *Acinetobacter,* with some cases in undetermined genera and a single case of *Psychrobacter*. Two gene variants were observed with no taxonomic or geographical clustering, but the flanking regions formed three clusters, of which the most identical included all North American samples. A more variable cluster included samples from all regions except North America, and the last cluster almost exclusively contained Asian and African samples.

mdf(A) is a broad-spectrum antimicrobial resistance gene originally described from *Escherichia*[22], and in our study mainly found on contigs with conserved gene synteny and unassigned to phyla, and various proteobacteria with *Escherichia* and a *Klebsiella* peduovirus phage being the most frequent. Besides some outliers, six gene clusters were observed with one cluster mainly associated with *Escherichia* and *Klebsiella* peduovirus and found in Europe, whereas the remaining five clusters were from large unclassified phyla, but still with some degree of regional clustering.

erm(B) has been reported from a very large range of Gram-positive and Gram-negative bacterial species[23]. We mainly found *erm*(B) on contigs assigned to *Enterococcus*, *Lactococcus*, *Carnobacterium* and *Staphylococcus,* in decreasing frequency. Most genes were similar with a smaller deviating cluster. The flanking regions varied, with most samples from Europe and North America being relatively identical. The gene had much more variable flanks among Asian and African samples. This variation seems mainly to be caused by differences in gene synteny, rather than mutation accumulation.

While *sul*1 is linked to integrons, *sul*2 has mainly been reported on small plasmids[24]. In our study, many very similar or identical copies of *sul*2 were found in a broad range of bacterial genera across all regions. However, when analyzing the flanking regions, two major clusters could be identified. A cluster of very similar contigs with similar gene synteny, predominantly from Europe and North America. This was divided into two sub-clusters of which one was exclusively assigned to *Aeromonas.* Secondly, a much

more diverse cluster with more variable gene organization was predominantly found in Asian and African samples.

bla$_{\mathrm{AER-1}}$ was originally isolated from *Aeromonas hydrophila* from India[25], but was in our study manly observed among undetermined and *Proteobacterial* genera. In terms of flanking regions, they had highly similar gene synteny, but formed, except for a three-sample outgroup, two clusters: one exclusively with Asian and African members, and another with predominantly European and North American sequences.

*aac(*3)-I was originally identified on a plasmid in *Pseudomonas aeruginosa*[26]. All flanked versions of the gene were indeed assigned to *Pseudomonas* and the flanking regions were generally very similar.

lnu(D) has been described from *Streptococcus uberis*[27]. We observed seven major gene clusters that each had little within-cluster variability. The genes were split between unassigned and assigned to *Gammaproteobacteria* or *Bacilli*. Among the latter, the majority were assigned to *Enterococcus*, which was the most frequently assigned genus. The flanking regions were more variable within the clusters mainly formed by differences in gene synteny and showing some regional predominance and taxonomic clustering.

catQ has been observed in streptococci and *Clostridium*[28–30]. We observed this gene on contigs with very similar gene synteny assigned to *Enterococcus, Lactococcus, Exiguobacterium* and *Salmonella.* Again, more genetic variation was observed among samples from Asia and Africa. Some highly similar flanking regions were exclusively assigned to *Enterococcus* and observed in all regions, while more highly variable flanking regions were assigned to the other three genera and were predominantly in African samples. Flanks suggested multiple possible transfer events and a possible introduction to *Salmonella* from *Exiguobacterium*.

The *floR* gene has been detected in multiple Gram-negative bacterial species[31]. In our data, four clusters of genetic variants were discovered; one highly similar with multiple genera and across all regions and other more variable clusters mainly associated to *Pseudomonas* and predominantly in samples from Asia and Africa. A similar clustering was observed for the flanking regions suggesting evolution of the gene within its genetic context.

cfxA5 was cloned from the chromosome of *Bacteroides distasonis,* and all copies were in our data assigned to *Butyricimonas*; a known human gut inhabitant of the same order. There was no noticeable clustering of genes or flanks across the regions.

tet(O) has been observed in multiple Gram-positive bacterial species and *Campylobacter*[32]. We observed highly similar gene variants exclusively assigned to *Salmonella* and with no regional clustering for gene or flanking regions. The unanimous taxonomic assignment was surprising, given that it is an unusual host, not normally associated with *tet*(O).

tet(S) has been described from Gram-positive cocci and *Listeria* and were in our data exclusively on contigs assigned to *Lactobacillales* members, including *Enterococcus*, *Lactococcus* and *Streptococcus*. No regional clustering was observed for gene variants or flanking regions.

aac(3)-IId has been observed in several bacterial species belonging to *Enterobacteriaceae*[33–36]. We found this gene mainly assigned to contigs of *Escherichia* and without clustering of genes or flanking regions. The variation in the flanking regions was seemingly not associated with differences in gene synteny.

tet(32) has previously been detected in *Clostridium*[32], but also recently observed in *Streptococcus pneumonia*[37]. All but one t*et*(32) were indeed discovered on *Streptococcus* contigs, with the outlier belonging to the Actinobacterium genus *Cutibacterium,* which was surprising. Both genes and flanking regions revealed two separate clusters; one observed in Asia and Africa, and another spread across all regions of the world, except South Asia. The flanking gene synteny differed between the two clusters.

tet(B) has been described from multiple Gram-negative species[32] and we also found this gene in multiple genera, with no noteworthy clustering based on gene or flanks.

fosA has mainly been observed on plasmids in *Enterobacteriaceae*[38]. We observed the gene on *Proteobacteria* contigs, most frequently belonging to *Escherichia*. Multiple different low-similarity clusters and deep branches in both the gene and flank trees were seen, where some of the major changes likely are due to changes in gene synteny.

The *cat* gene was originally cloned from *Proteus mirabillis*[39]. We found this gene mainly in samples from Africa and Europe and mainly on *Aeromonas* contigs. One *Aeromonas* group comprised mainly European samples, while another smaller was exclusively from Africa. A smaller, distinct group of African *cat* genes on *Enterococcus* and *Staphylococcus* contigs and different gene synteny was also observed.

lnu(C) has been detected in *Streptococcus*, *Brachyspira* and *Campylobacter*[23,40–42] and was in our study indeed mostly assigned to *Streptococcus,* with single instances of *Campylobacter*- and *Lactobacillus*-assigned genes. The genes had almost identical sequence, but varying flanking regions across all regions, mainly due to differences in gene synteny.

oqaX was cloned from *Escherichia coli*[43] and was by us observed mainly on *Klebsiella* contigs, with fewer instances on *Enterobacter* and *Escherichia* contigs. It had variable flanking contexts but seemingly in two main clusters, with some regional predominance. Samples with more similar gene synteny and flanking regions also had similar gene variants.

bla$_{\text{TEM-1}}$ was one of the first beta-lactamases to be discovered and while originally from a plasmid in *Pseudomonas aeruginosa*[44], it has been observed across multiple Gram-negative species. We also observed this gene in multiple *Proteobacteria* genera and with varying flanking regions and mainly in samples from Asia and Africa, but with no apparent geographic clustering.

erm(F) has been observed in multiple Gram-positive species[23]. We found the gene assigned to *Bacteroides* and *Myroides* with some variation in the flanking regions associated to differences in gene synteny, but no obvious regional clustering. The gene variants assigned to *Myroides* were more similar than those assigned to *Bacteroides.*

tet(X) was observed in *Myroides* and *Bacteroides* and has previously exclusively been reported in *Bacteroides*[32]. Two highly similar gene variants were observed located on two much more variable flanking gene clusters with differences in gene synteny; one mainly associated with *Bacteroides* in Europe, and another mainly associated with *Myroides* in North America and Asia.

cmlB was cloned from *Bordetella bronchispetica*[45] and was by us observed mainly in *Acinetobacter* without obvious regional clustering. In two assemblies, the contigs were indeed assigned to *Bordetella*. The observed flanking gene variation was due to differences in gene synteny.

The *ant*(2'')-Ia gene has been detected in different Gram-negative phyla[46–48] and was observed by us in two major variants, based on gene variation, flanking gene variation and gene synteny. One variant was associated with *Acinetobacter* and another with *Klebsiella*, *Pseudomonas*, *Aeromonas* and other Gammaproteobacterial genera. No obvious regional clustering could be observed.

aph(3')-III has been described from both *Campylobacter* and streptococci[49–51], and was by us observed mostly in *Staphylococcus*, *Enterococcus* and *Campylobacter*, with no apparent regional clustering based on genes or flanking regions. The flanking regions of contigs assigned to *Staphylococcus* did however seem more similar.

bla$_{\text{DHA-1}}$ was originally cloned from *Salmonella*[52], but has also been found in other *Enterobacteriaceae*[53–55]. We found it almost exclusively assigned to *Enterobacte*r and with very similar flanking regions, without major regional clustering.

bla$_{\text{OXA-10}}$ has been found in multiple Gram-negative species[56,57]. We also found this gene associated with multiple different Gammaproteobacterial genera. The gene sequences had very high identity, whereas the flanks were very divergent also in gene synteny.

While originally detected on a transposon in *Enterococcus faecalis*[58], lnu(G) has also been observed in *Campylobacte*r and *Staphylococcus*[59,60]. In our study, *lnu*(G) was almost always assigned to *Staphylococcus* from North America and Europe. The single exception was a divergent variant assigned to African *Proteus mirabilis*. While the genes shared high identity, a very clear separation was observed for the flanking regions, including differences in gene synteny with most samples from North America forming one cluster, and those from Europe, another. The African *Proteus* gene variant shared high identity with one of the European *Staphylococcus* variants, highlighting the cross-phylum jump.

Supplementary References

1. Roberts, M. C. Environmental macrolide-lincosamide-streptogramin and tetracycline resistant bacteria. *Front. Microbiol.* **2**, 1–8 (2011).
2. Liu, J., Keelan, P., Bennett, P. M. & Enne, V. I. Characterization of a novel macrolide efflux gene, mef (B), found linked to sul3 in porcine Escherichia coli. *J. Antimicrob. Chemother.* **63**, 423–426 (2009).
3. Vitali, L. A., Di Luca, M. C., Prenna, M. & Petrelli, D. Correlation between genetic features of the mef(A)-msr(D) locus and erythromycin resistance in Streptococcus pyogenes. *Diagn. Microbiol. Infect. Dis.* **84**, 57–62 (2016).
4. Lepine, G., Lacroix, J. M., Walker, C. B. & Progulske-Fox, A. Sequencing of a tet(Q) gene isolated from Bacteroides fragilis 1126. *Antimicrob. Agents Chemother.* **37**, 2037–2041 (1993).
5. Chung, W. O., Gabany, J., Persson, G. R. & Roberts, M. C. Distribution of erm(F) and tet(Q) genes in 4 oral bacterial species and genotypic variation between resistant and susceptible isolates. *J. Clin. Periodontol.* **29**, 152–158 (2002).
6. Ma, Y., Xie, T. T., Hu, Q., Qiu, Z. & Song, F. Sequencing analysis and characterization of the plasmid pBIF10 isolated from Bifidobacterium longum. *Can. J. Microbiol.* **61**, 124–130 (2015).
7. Sundin, G. W. & Bender, C. L. Dissemination of the strA-strB streptomycin-resistance genes among commensal and pathogenic bacteria from humans, animals, and plants. *Mol. Ecol.* **5**, 133–143 (1996).
8. Berbel, D. *et al.* A novel genomic island harbouring lsa(E) and lnu(B) genes and a defective prophage in a Streptococcus pyogenes isolate resistant to lincosamide, streptogramin A and pleuromutilin antibiotics. *Int. J. Antimicrob. Agents* **54**, 647–651 (2019).
9. Feßler, A. T., Wang, Y., Wu, C. & Schwarz, S. Mobile lincosamide resistance genes in staphylococci. *Plasmid* **99**, 22–31 (2018).
10. O'Connor, E. B. *et al.* pEOC01: A plasmid from Pediococcus acidilactici which encodes an identical streptomycin resistance (aadE) gene to that found in

Campylobacter jejuni. *Plasmid* **58**, 115–126 (2007).

11. Werner, G., Hildebrandt, B. & Witte, W. Aminoglycoside-streptothricin resistance gene cluster aadE-sat4-aphA-3 disseminated among multiresistant isolates of enterococcus faecium. *Antimicrob. Agents Chemother.* **45**, 3267–3269 (2001).

12. Pinto-Alphandary, H., Mabilat, C. & Courvalin, P. Emergence of aminoglycoside resistance genes aadA and aadE in the genus Campylobacter. *Antimicrob. Agents Chemother.* **34**, 1294–1296 (1990).

13. Derbise, A., Dyke, K. G. H. & Solh, N. El. Located within Tn 5404 and Carrying the Aminoglycoside Resistance Genes , aphA-3 and aadE. *Plasmid* **188**, 174–188 (1996).

14. Desmolaize, B., Rose, S., Wilhelm, C., Warrass, R. & Douthwaite, S. Combinations of macrolide resistance determinants in field isolates of Mannheimia haemolytica and Pasteurella multocida. *Antimicrob. Agents Chemother.* **55**, 4128–4133 (2011).

15. Mohanraj, R. S., Samanta, P., Mukhopadhyay, A. K. & Mandal, J. Haitian-like genetic traits with creeping MIC of Azithromycin in Vibrio cholerae O1 isolates from Puducherry, India. *J. Med. Microbiol.* **69**, 372–378 (2020).

16. Tang, L. *et al.* Whole-Genome Analysis of Two Copies of bla NDM-1 Gene Carrying Acinetobacter johnsonii. 855–865 (2020).

17. Galan, J. C., Reig, M., Navas, A., Baquero, F. & Blazquez, J. ACI-1 from Acidaminococcus fermentans: Characterization of the first β-lactamase in anaerobic cocci. *Antimicrob. Agents Chemother.* **44**, 3144–3149 (2000).

18. Rodríguez-Martínez, J. M., Cano, M. E., Velasco, C., Martínez-Martínez, L. & Pascual, Á. Plasmid-mediated quinolone resistance: An update. *J. Infect. Chemother.* **17**, 149–182 (2011).

19. Sugimoto, Y. *et al.* The novel mef(C)–mph(G) macrolide resistance genes are conveyed in the environment on various vectors. *J. Glob. Antimicrob. Resist.* **10**, 47–53 (2017).

20. Nonaka, L., Maruyama, F., Suzuki, S. & Masuda, M. Novel macrolide-resistance

genes, mef(C) and mph(G), carried by plasmids from Vibrio and Photobacterium isolated from sediment and seawater of a coastal aquaculture site. *Lett. Appl. Microbiol.* **61**, 1–6 (2015).

21. Stokes, H. W. & Hall, R. M. A novel family of potentially mobile DNA elements encoding site-specific gene-integration functions: integrons. *Mol. Microbiol.* **3**, 1669–1683 (1989).

22. Edgar, R. & Bibi, E. MdfA, an Escherichia coli multidrug resistance protein with an extraordinarily broad spectrum of drug recognition. *J. Bacteriol.* **179**, 2274–2280 (1997).

23. Roberts, M. C. Update on macrolide-lincosamide-streptogramin, ketolide, and oxazolidinone resistance genes. *FEMS Microbiol. Lett.* **282**, 147–159 (2008).

24. Van Treeck, U., Schmidt, F. & Wiedemann, B. Molecular nature of a streptomycin and sulfonamide resistance plasmid (pBP1) prevalent in clinical Escherichia coli strains and integration of an ampicillin resistance transposon (TnA). *Antimicrob. Agents Chemother.* **19**, 371–380 (1981).

25. Sanschagrin, F., Bejaoui, N. & Levesque, R. C. Structure of CARB-4 and AER-1 Carbenicillin- Hydrolyzing ␤ -Lactamases. **42**, 1966–1972 (1998).

26. Haines, A. S., Jones, K., Cheung, M. & Thomas, C. M. The IncP-6 plasmid Rms149 consists of a small mobilizable backbone with multiple large insertions. *J. Bacteriol.* **187**, 4728–4738 (2005).

27. Petinaki, E. *et al.* Lincomycin resistance gene lnu(D) in Streptococcus uberis. *Antimicrob. Agents Chemother.* **52**, 626–630 (2008).

28. Mingoia, M., Morici, E., Brenciani, A., Giovanetti, E. & Varaldo, P. E. Genetic basis of the association of resistance genes mef(I) (macrolides) and catQ (chloramphenicol) in streptococci. *Front. Microbiol.* **6**, 1–5 (2015).

29. Morici, E. *et al.* A new mosaic integrative and conjugative element from Streptococcus agalactiae carrying resistance genes for chloramphenicol (catQ) and macrolides [mef(I) and erm(TR)]. *J. Antimicrob. Chemother.* **72**, 64–67 (2017).

30. Bannam, T. L. & Rood, J. I. Relationship between the Clostridium perfringens

catQ gene product and chloramphenicol acetyltransferases from other bacteria. *Antimicrob. Agents Chemother.* **35**, 471–476 (1991).

31. *Antimicrobial resistance in bacteria of animal origin*. (ASM Press, 2006).

32. Roberts, M. C. Update on acquired tetracycline resistance genes. *FEMS Microbiol. Lett.* **245**, 195–203 (2005).

33. Mairi, A., Barraud, O., Muggeo, A., de Champs, C. & Touati, A. Genomic analysis of a multidrug-resistant Klebsiella pneumoniae ST11 strain recovered from Barbary deer (Cervus elaphus barbarus) in Akfadou Forest, Algeria. *J. Glob. Antimicrob. Resist.* **22**, 515–518 (2020).

34. Li, Z. *et al.* Genetic characterisation of a complex class 1 integron in an NDM-1-producing Citrobacter freundii ST396 clinical strain isolated from a urine sample. *J. Glob. Antimicrob. Resist.* **23**, 64–66 (2020).

35. Ho, P. L. *et al.* Genetic identity of aminoglycoside-resistance genes in Escherichia coli isolates from human and animal sources. *J. Med. Microbiol.* **59**, 702–707 (2010).

36. Liao, Y.-S., Chen, B.-H., Hong, Y.-P., Teng, R.-H. & Wang, Y.-W. Emergence of Multidrug-Resistant Salmonella enterica Serovar Goldcoast Strains in Taiwan and International Spread of the ST358 Clone. *Epidemiol. Surveill.* **63**, 1–7 (2019).

37. Nikolaou, E. *et al.* Antibiotic resistance is associated with integrative and conjugative elements and genomic islands in naturally circulating streptococcus pneumoniae isolates from adults in liverpool, uk. *Genes (Basel).* **11**, 1–9 (2020).

38. Yang, T. Y., Lu, P. L. & Tseng, S. P. Update on fosfomycin-modified genes in Enterobacteriaceae. *J. Microbiol. Immunol. Infect.* **52**, 9–21 (2019).

39. Charles, I. G., Keyte, J. W. & Shaw, W. V. Nucleotide sequence analysis of the cat gene of Proteus mirabilis: Comparison with the type I (Tn9) cat gene. *J. Bacteriol.* **164**, 123–129 (1985).

40. De Luca, S. *et al.* Transposon-associated lincosamide resistance lnu(C) gene identified in Brachyspira hyodysenteriae ST83. *Vet. Microbiol.* **214**, 51–55 (2018).

41. Wu, G. *et al.* Diminution of the gut resistome after a gut microbiota-targeted dietary intervention in obese children. *Sci. Rep.* **6**, 24030 (2016).

42. Zhao, S. *et al.* Whole-genome sequencing analysis accurately predicts antimicrobial resistance phenotypes in Campylobacter spp. *Appl. Environ. Microbiol.* **82**, 459–466 (2016).

43. Hansen, L. H., Johannesen, E., Burmølle, M., Sørensen, A. H. & Sørensen, S. J. Plasmid-encoded multidrug efflux pump conferring resistance to olaquindox in Escherichia coli. *Antimicrob. Agents Chemother.* **48**, 3332–3337 (2004).

44. Datta, N., Hedges, R. W., Shaw, E. J., Sykes, R. B. & Richmond, M. H. Properties of an R factor from Pseudomonas aeruginosa. *J. Bacteriol.* **108**, 1244–1249 (1971).

45. Kadlec, K., Kehrenberg, C. & Schwarz, S. Efflux-mediated resistance to florfenicol and/or chloramphenicol in Bordetella bronchiseptica: Identification of a novel chloramphenicol exporter. *J. Antimicrob. Chemother.* **59**, 191–196 (2007).

46. Rocha, D. J. P. *et al.* Whole-genome sequencing reveals misidentification of a multidrug-resistant urine clinical isolate as Corynebacterium urealyticum. *J. Glob. Antimicrob. Resist.* **23**, 16–19 (2020).

47. Ahmed, A. M., Nakano, H. & Shimamoto, T. Molecular characterization of integrons in non-typhoid Salmonella serovars isolated in Japan: Description of an unusual class 2 integron. *J. Antimicrob. Chemother.* **55**, 371–374 (2005).

48. Cameron, F. H., Groot Obbink, D. J., Ackerman, V. P. & Hall, R. M. Nucleotide sequence of the AAD(2′) aminoglycoside adenylyltransferase determinant aadB. Evolutionary relationship of this region with those surrounding aadA in R538-1 and dhfrII in R388. *Nucleic Acids Res.* **14**, 8625–8635 (1986).

49. Courvalin, P. & Carlier, C. Transposable multiple antibiotic resistance in Streptococcus pneumoniae. *MGG Mol. Gen. Genet.* **205**, 291–297 (1986).

50. Papadopoulou, B. & Courvalin, P. Dispersal in Campylobacter spp. of aphA-3, a kanamycin resistance determinant from gram-positive cocci. *Antimicrob. Agents Chemother.* **32**, 945–948 (1988).

51. Granlund, M. *et al.* Antimicrobial resistance in colonizing group B Streptococci before the implementation of a Swedish intrapartum antibiotic prophylaxis program. *Eur. J. Clin. Microbiol. Infect. Dis.* **29**, 1195–1201 (2010).

52. Barnaud, G. *et al.* Salmonella enteritidis: AmpC plasmid-mediated inducible β-lactamase (DHA-1) with an ampR gene from Morganella morganii. *Antimicrob. Agents Chemother.* **42**, 2352–2358 (1998).

53. Xie, Y. *et al.* Emergence of the third-generation cephalosporin-resistant hypervirulent klebsiella pneumoniae due to the acquisition of a self-transferable blaDHA-1-carrying plasmid by an ST23 strain. *Virulence* **9**, 838–844 (2018).

54. Kao, C. Y. *et al.* Plasmid-mediated quinolone resistance determinants in quinolone-resistant Escherichia coli isolated from patients with bacteremia in a university hospital in Taiwan, 2001-2015. *Sci. Rep.* **6**, 1–8 (2016).

55. Mahrouki, S. *et al.* Nosocomial dissemination of plasmids carrying blaTEM-24, blaDHA-1, aac(6')-Ib-cr, and qnrA6 in Providencia spp. strains isolated from a Tunisian hospital. *Diagn. Microbiol. Infect. Dis.* **81**, 50–52 (2015).

56. Livermore, D. M., Maskell, J. P. & Williams, J. D. Detection of PSE-2 β-lactamase in enterobacteria. *Antimicrob. Agents Chemother.* **25**, 268–272 (1984).

57. Philippon, A. M., Paul, G. C. & Jacoby, G. A. Properties of PSE-2 β-lactamase and genetic basis for its production in Pseudomonas aeruginosa. *Antimicrob. Agents Chemother.* **24**, 362–369 (1983).

58. Zhu, X. Q. *et al.* Novel lnu(G) gene conferring resistance to lincomycin by nucleotidylation, located on Tn6260 from Enterococcus faecalis E531. *J. Antimicrob. Chemother.* **72**, 993–997 (2017).

59. Fabre, A. *et al.* Whole-genome sequence analysis of multidrug-resistant campylobacter isolates: A focus on aminoglycoside resistance determinants. *J. Clin. Microbiol.* **56**, 1–12 (2018).

60. Gu, J. *et al.* Prevalence and transmission of antimicrobial-resistant Staphylococci and Enterococci from shared bicycles in Chengdu, China. *Sci. Total Environ.* **738**, 1–9 (2020).
