# Supplementary material for: Genomic analysis of sewage from 101 countries reveals global landscape of antimicrobial resistance
Source: Nat Commun. 2022 Dec 1;13:7251. doi: 10.1038/s41467-022-34312-7 (PMC9715550; doi:10.1038/s41467-022-34312-7)
Supplement: Supplementary file 2 — Description of Additional Supplementary Files [file 41467_2022_34312_MOESM2_ESM.pdf]

## Description of Additional Supplementary Files:

**Supplementary Data 1:** Sample and sequencing overview. Table of sequencing datasets with relevant data on time, location and accession numbers. Notice that "unique_sample_id" refers to the actual biological sample that might have multiple associated sequencing runs.

**Supplementary Data 2:** Number of flanked ARGs. Table with counts of how often each ARG was found with flanking sequence and how frequently the contigs were assigned to a plasmid source.

**Supplementary Data 3:** ARG synteny trees based on UPGMA clustering of up- and down-stream 1Kb flanking sequence kmers. Prokka-identified features near the centered ARG are drawn in for each tree.

**Supplementary Data 4:** ARG synteny trees based on UPGMA clustering of ARG kmers when requiring 1Kb up- and down-stream flanking sequence. Prokkaidentified features near the centered ARG are drawn in for each gene.

**Supplementary Data 5:** ARG synteny trees based on UPGMA clustering of up- and down-stream 5Kb flanking sequence kmers. Prokka-identified features near the centered ARG are drawn in for each tree.

**Supplementary Data 6:** ARG synteny trees based on UPGMA clustering of ARG kmers when requiring 5Kb up- and down-stream flanking sequence. Prokkaidentified features near the centered ARG are drawn in for each gene.
