# Supplementary figures and images for: Genomic analysis of sewage from 101 countries reveals global landscape of antimicrobial resistance
Source: Nat Commun. 2022 Dec 1;13:7251. doi: 10.1038/s41467-022-34312-7 (PMC9715550; doi:10.1038/s41467-022-34312-7)

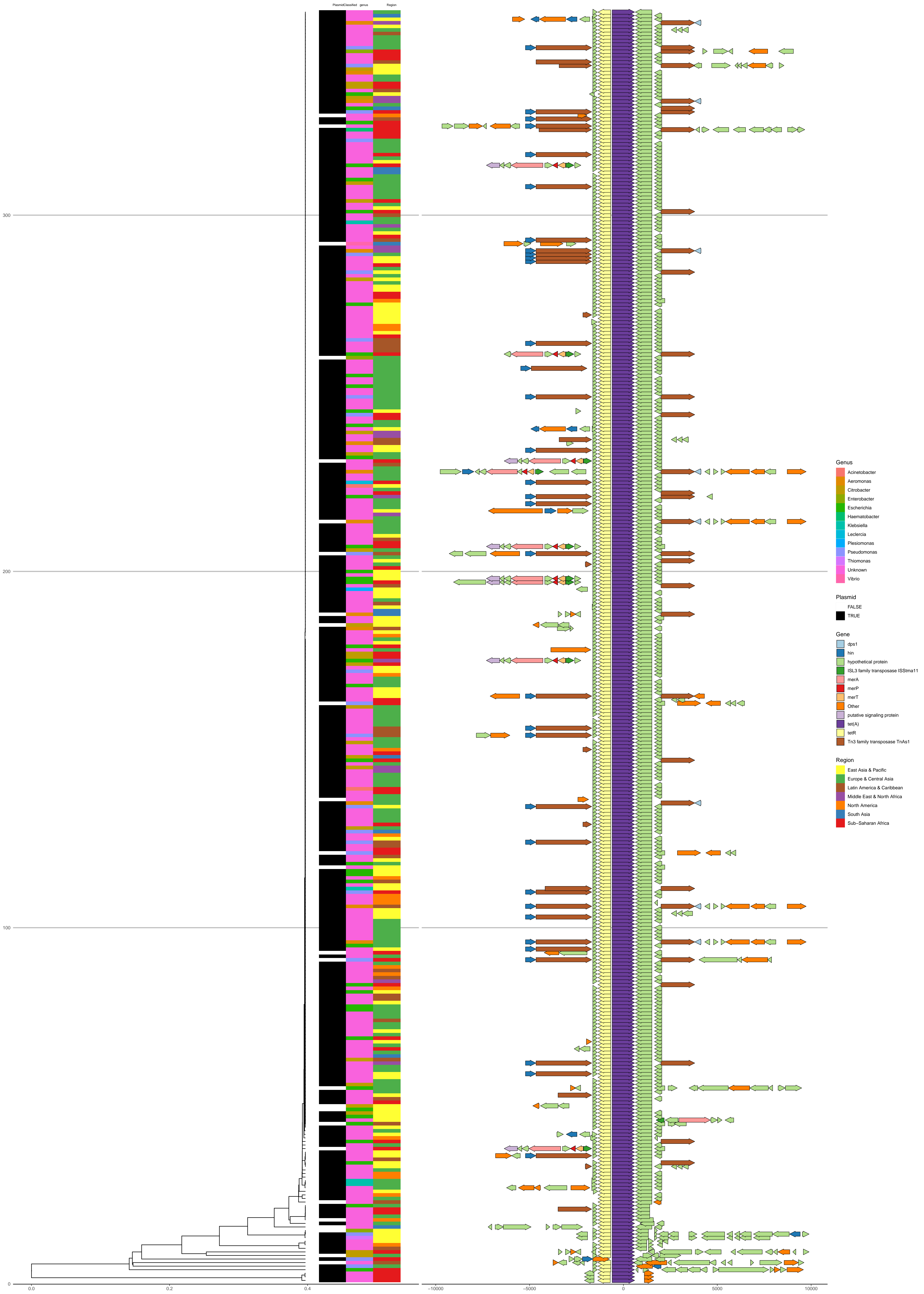

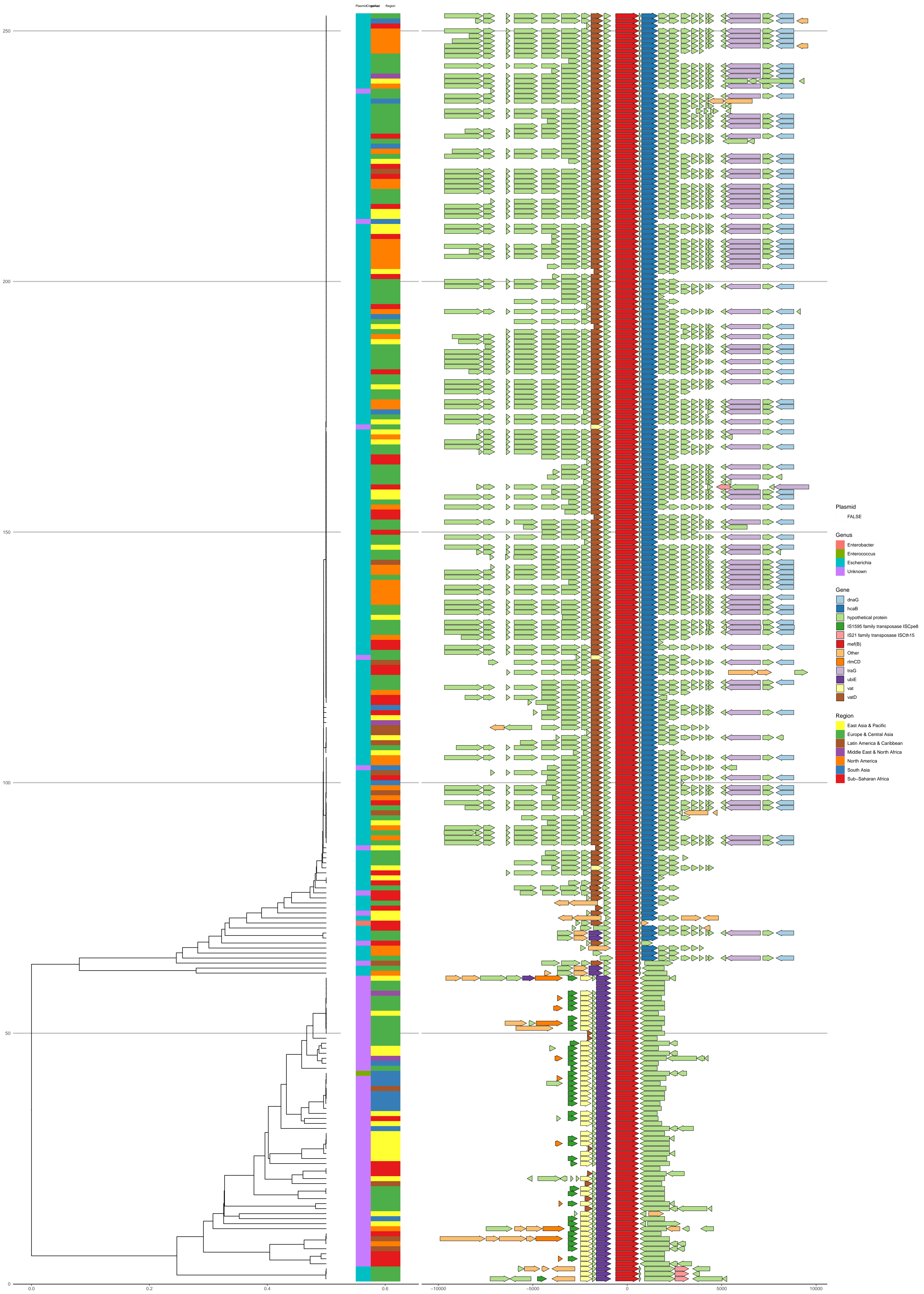

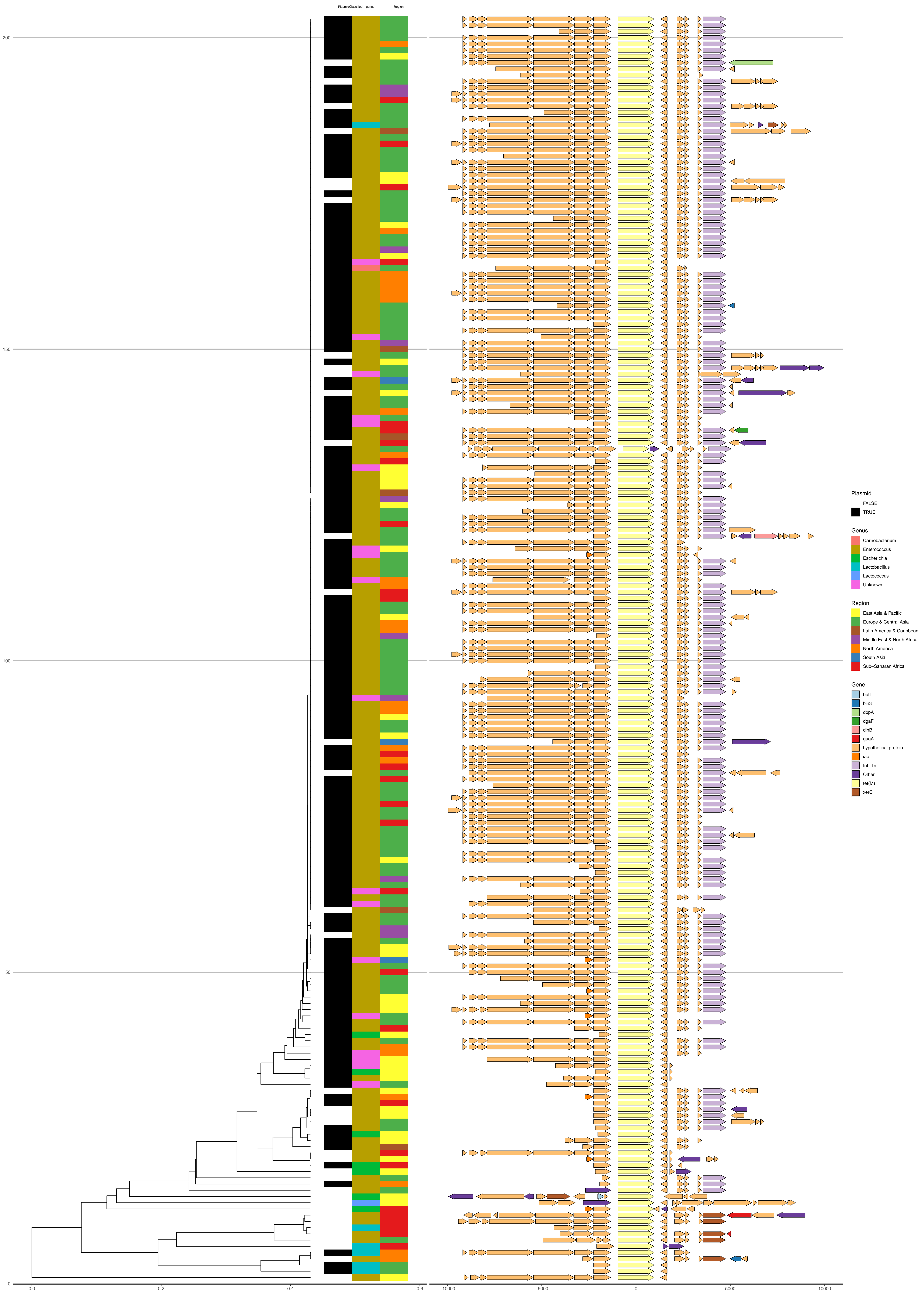

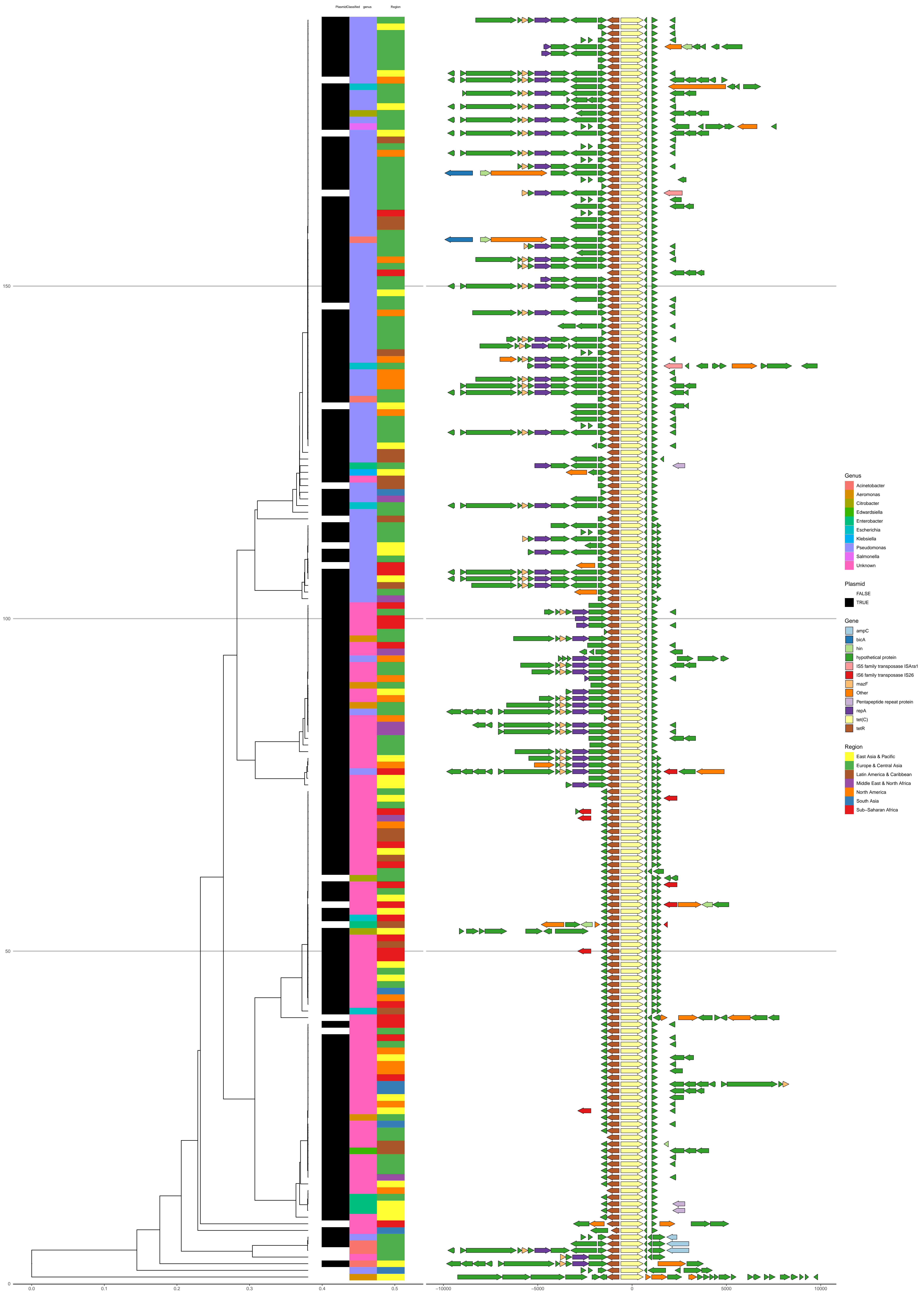

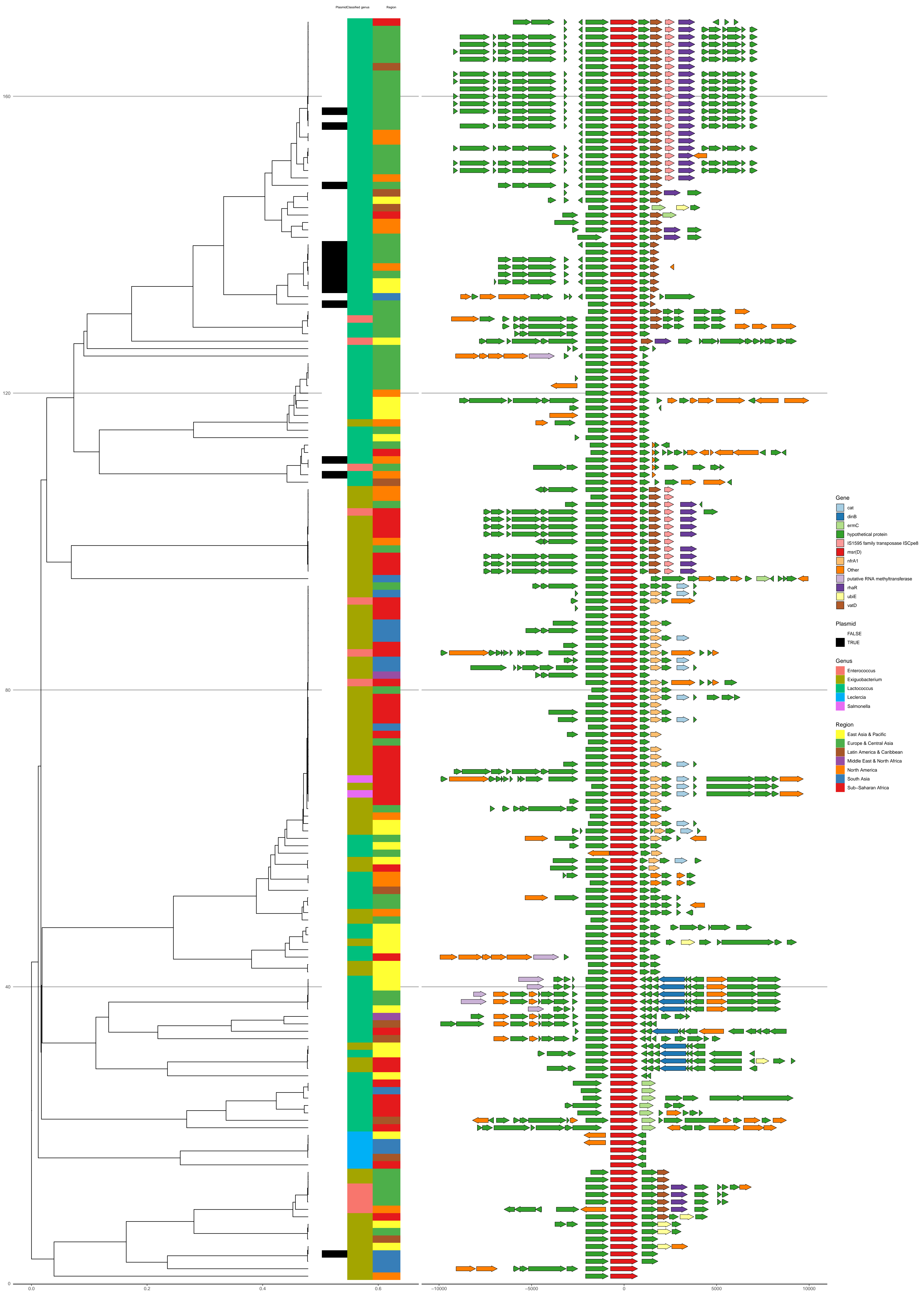

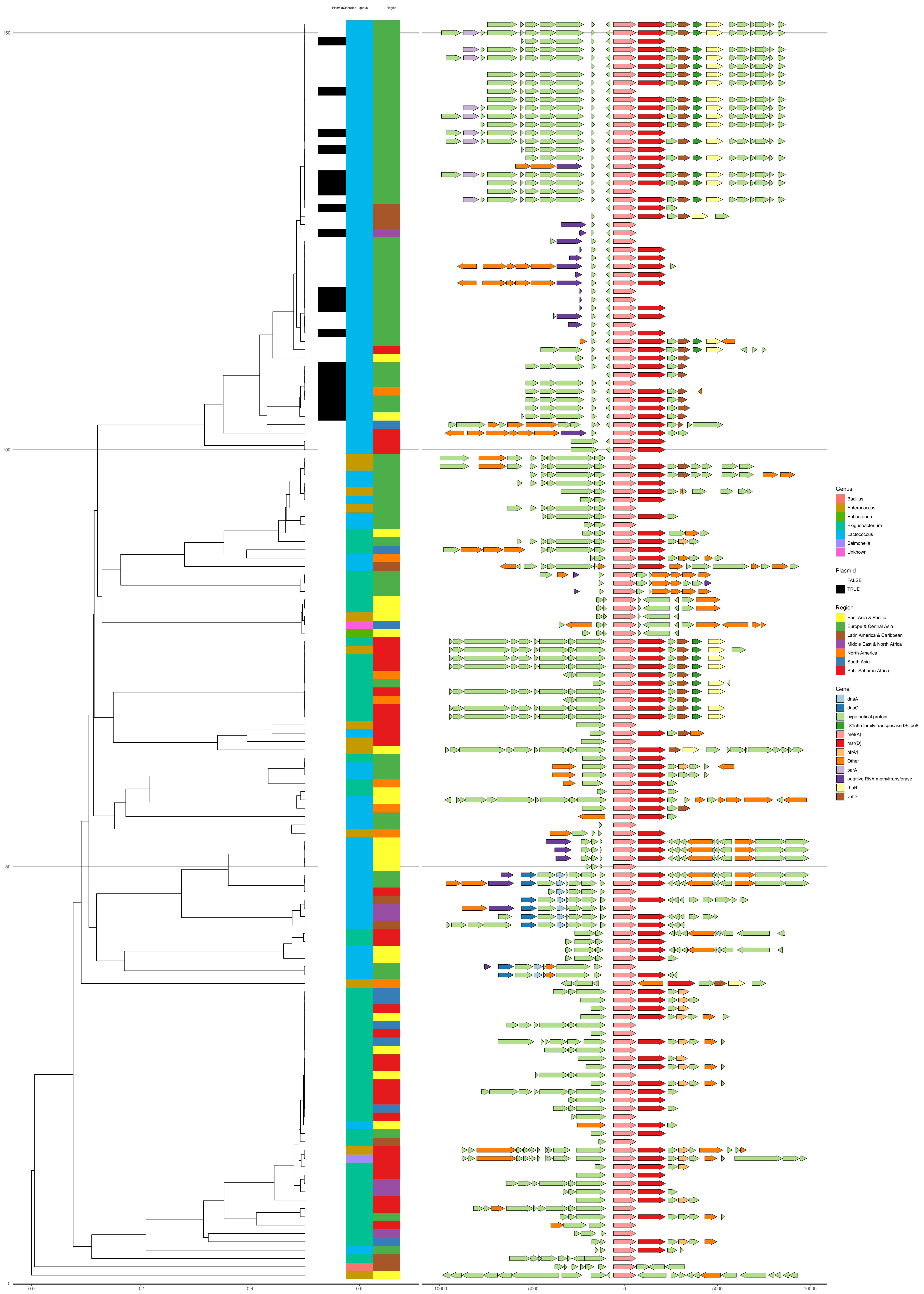

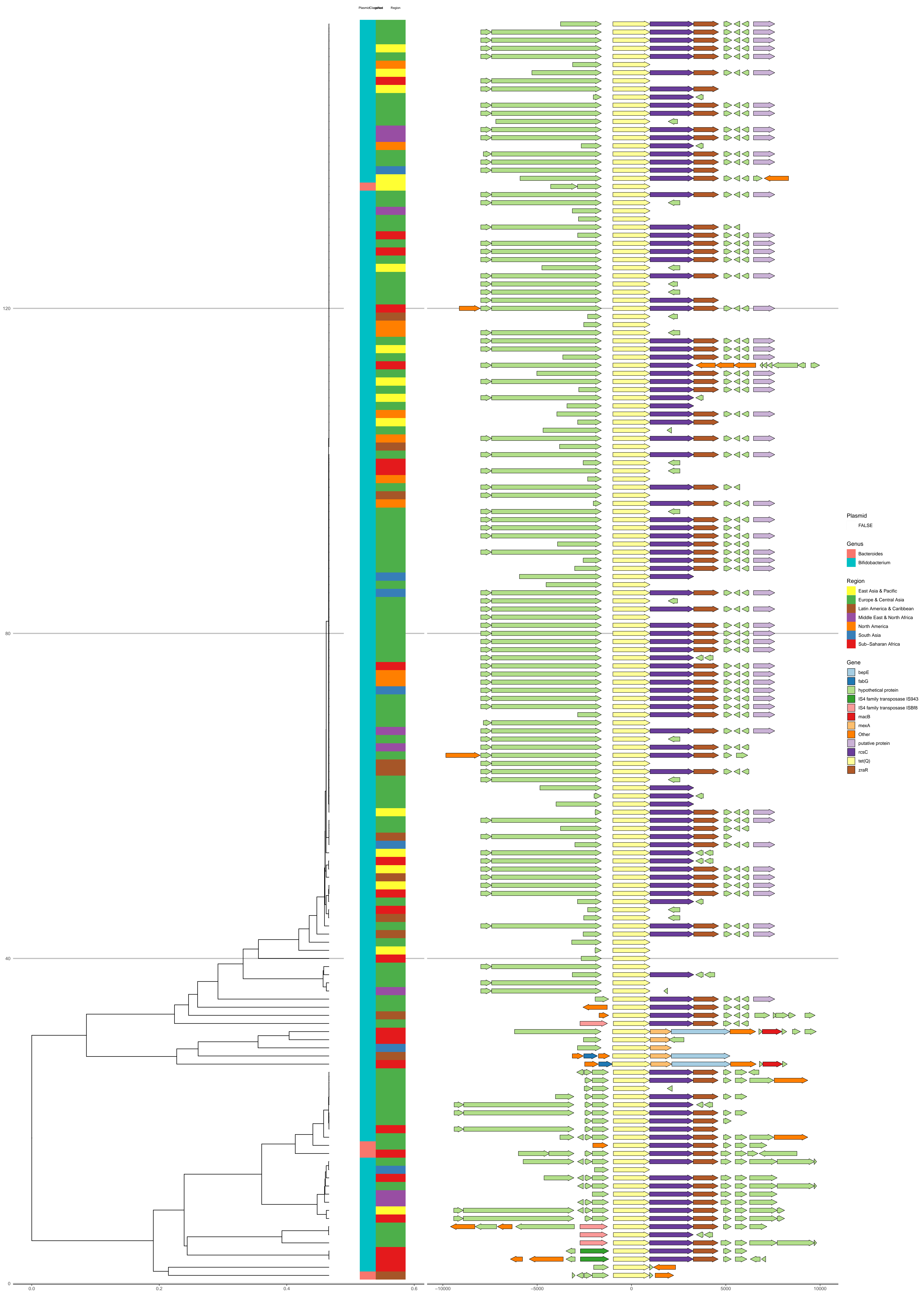

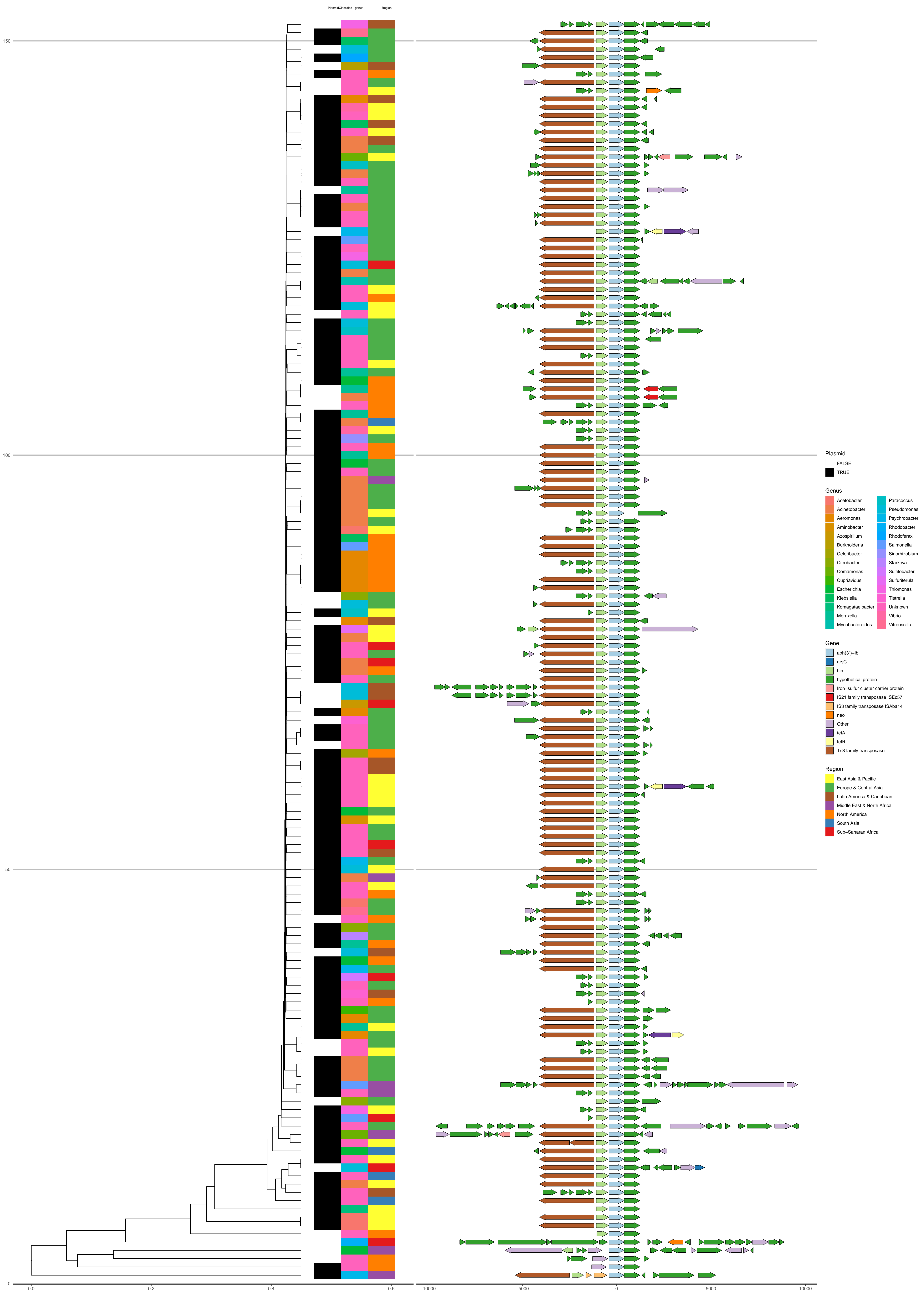

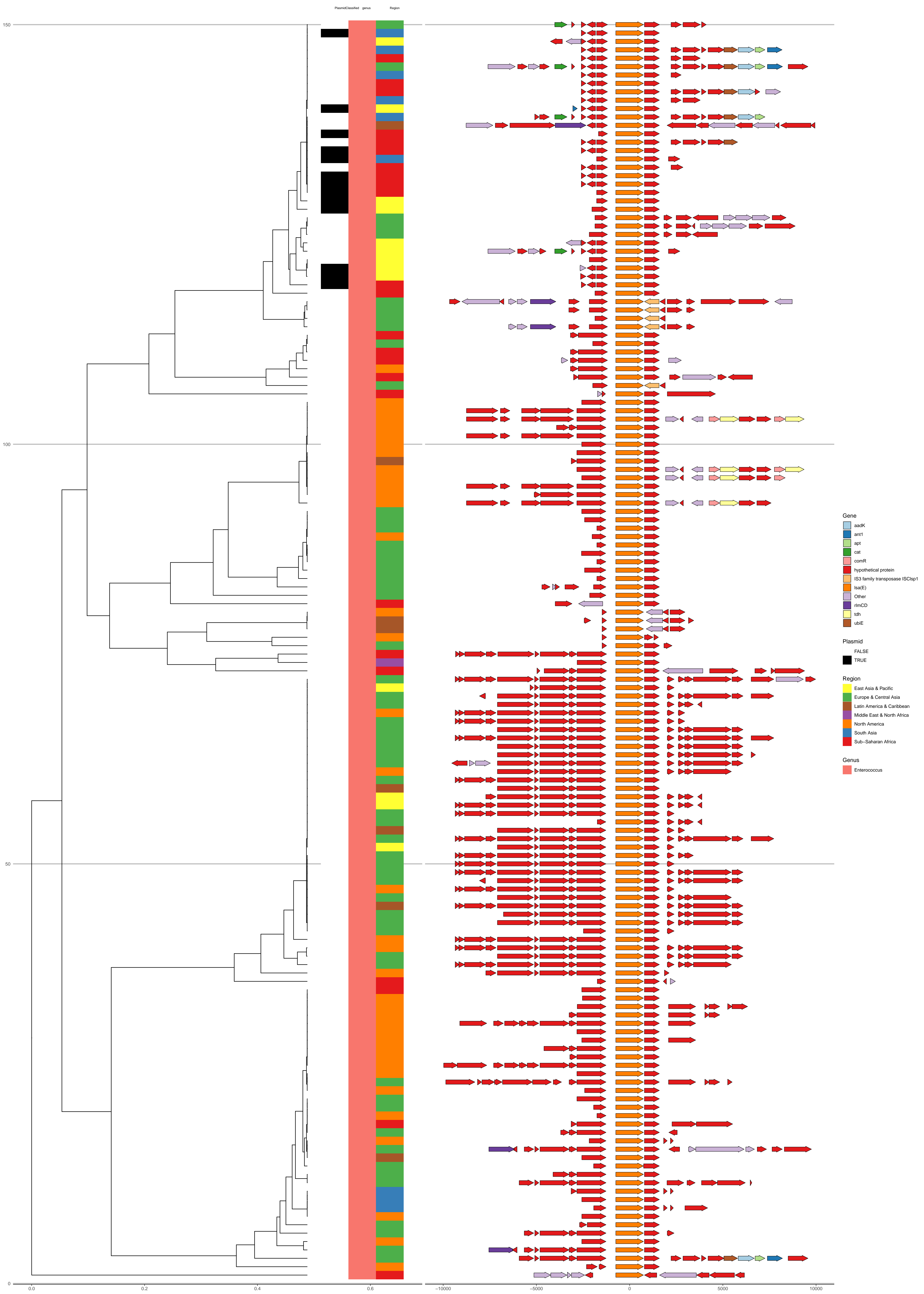

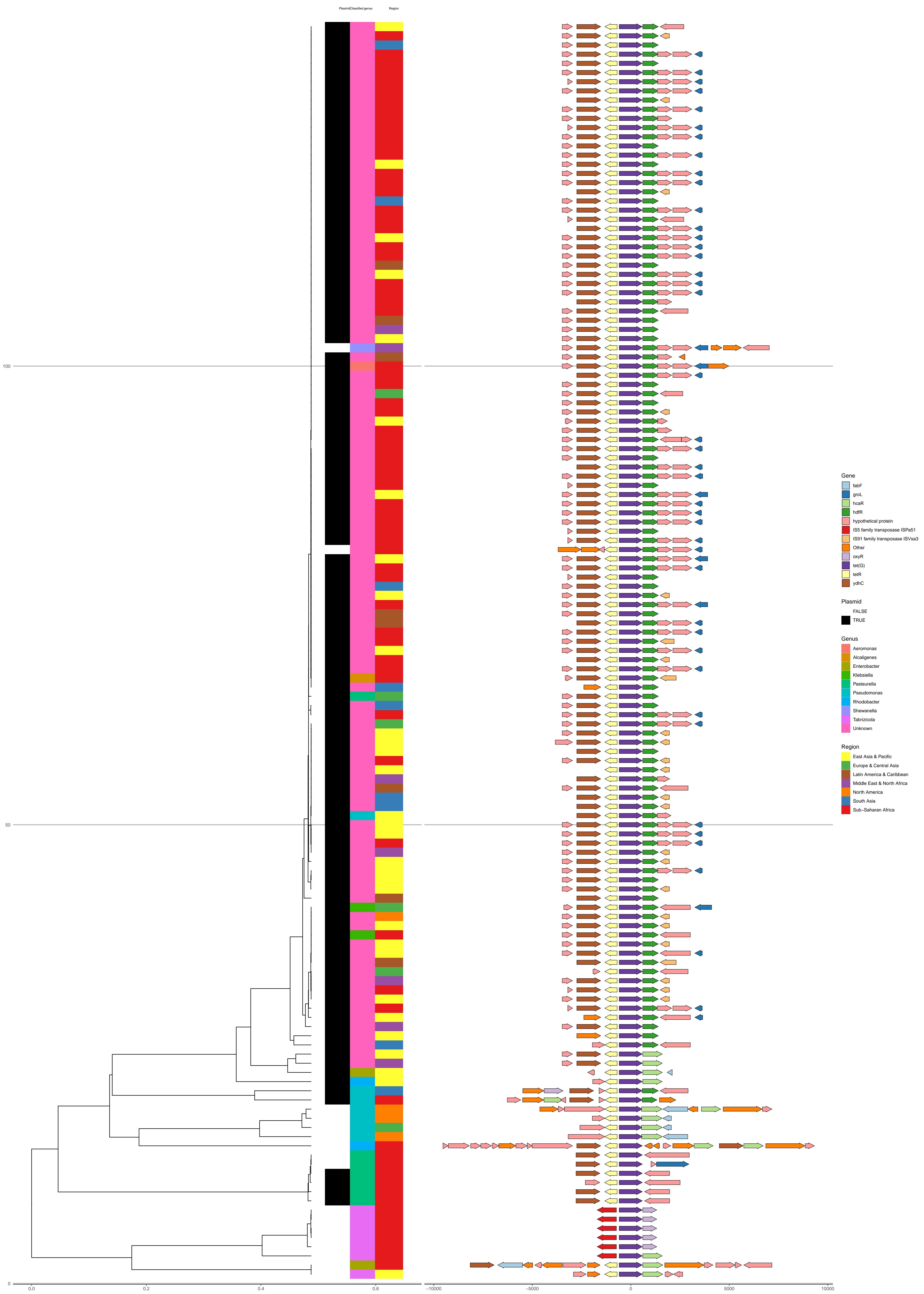

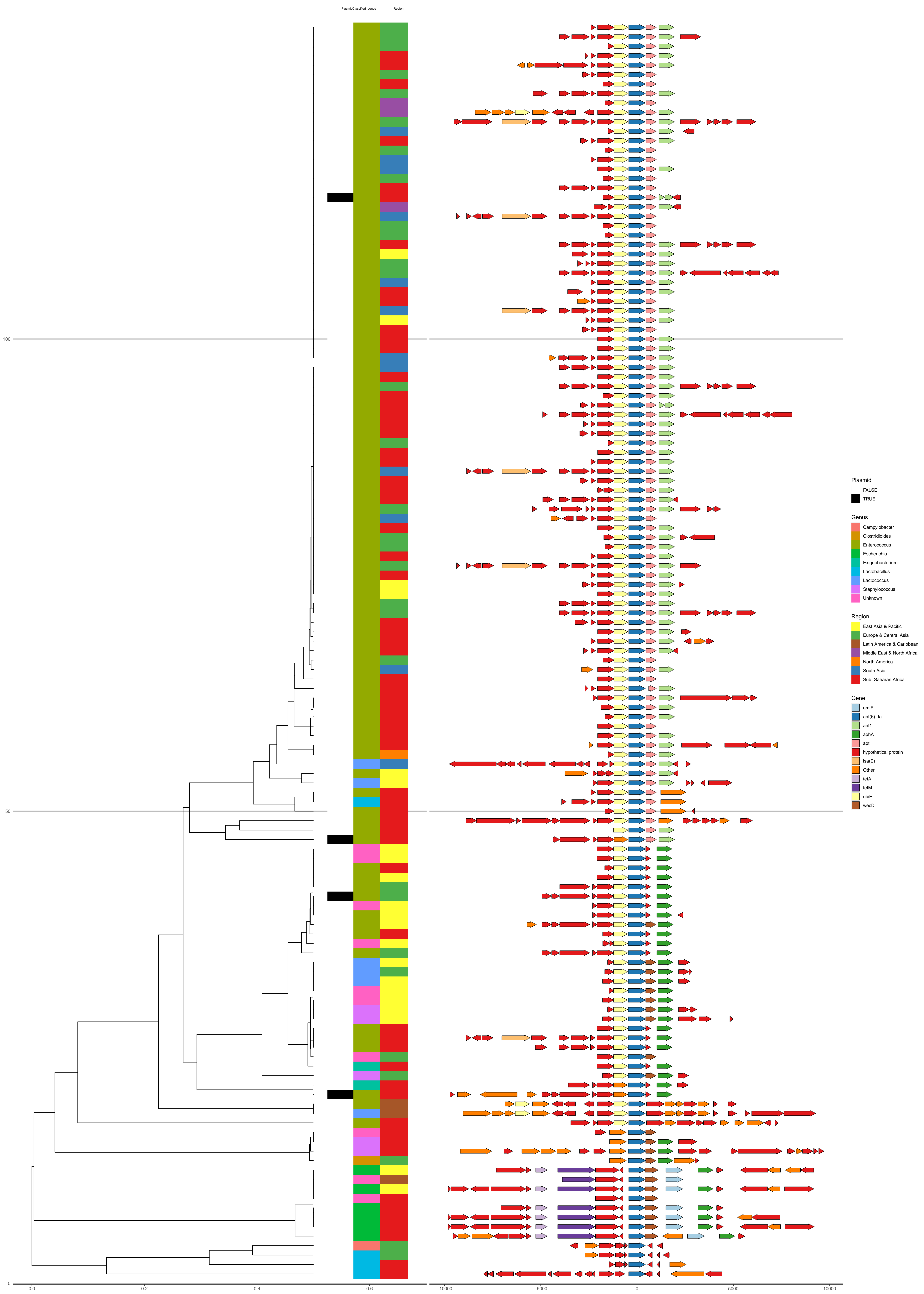

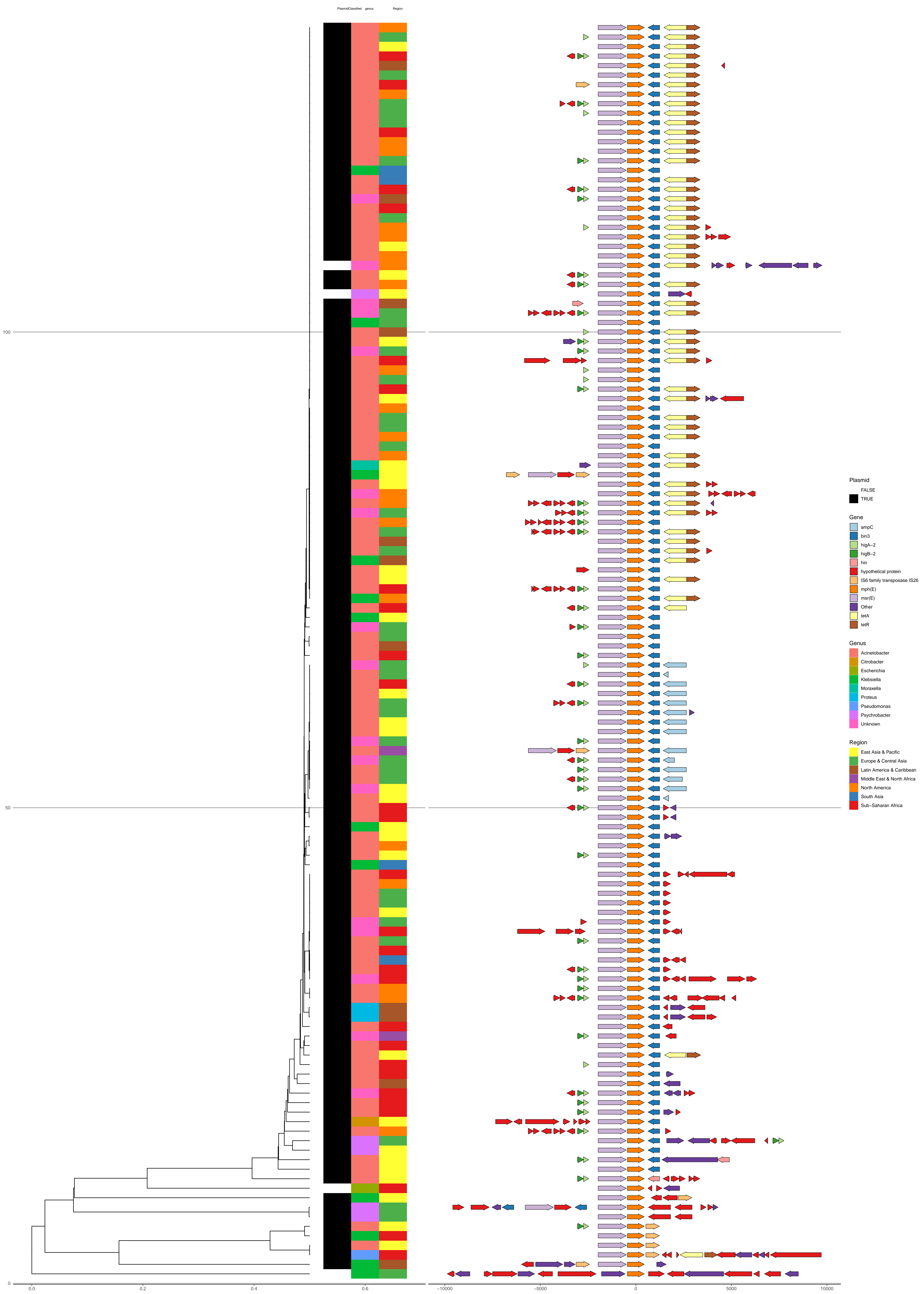

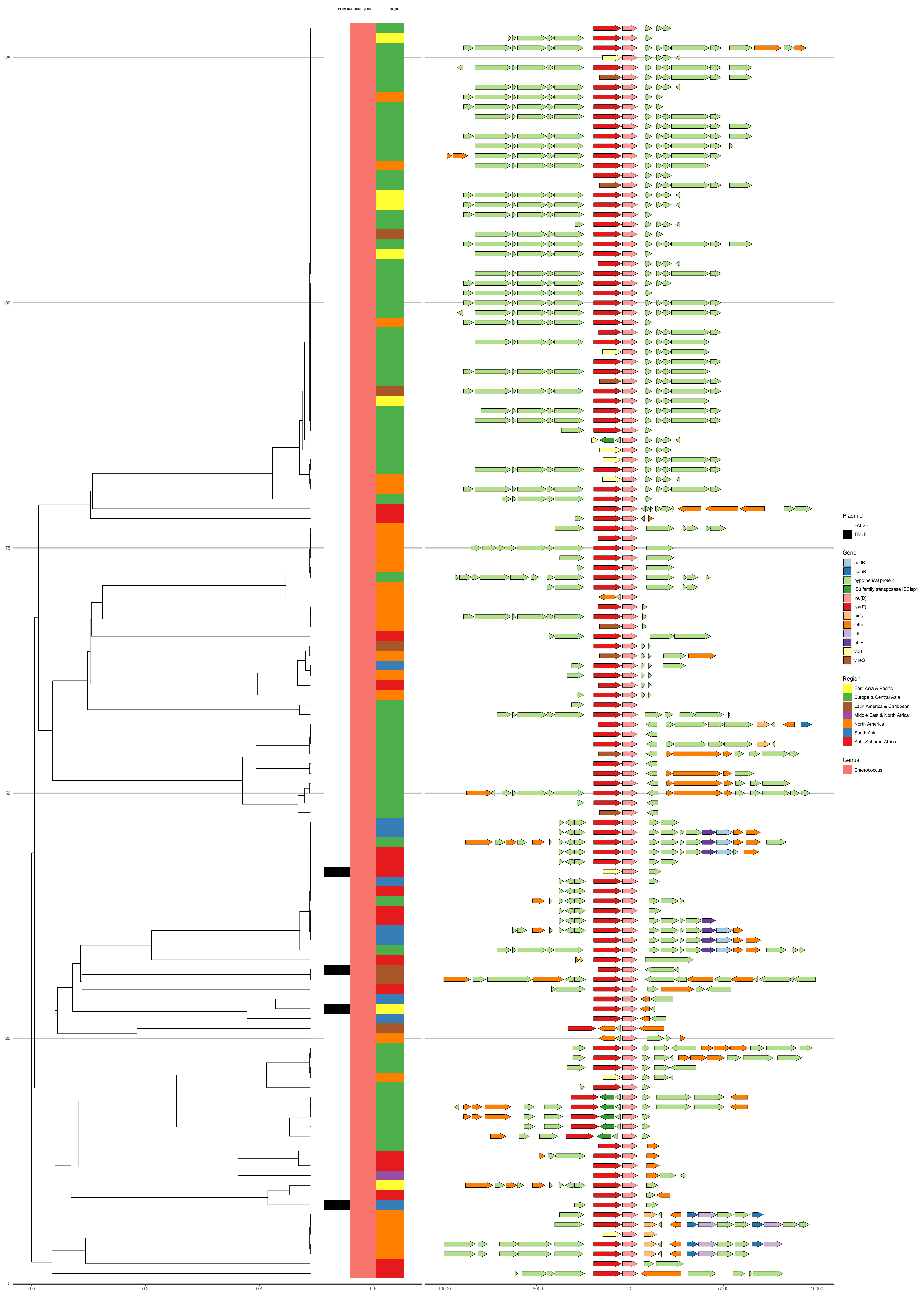

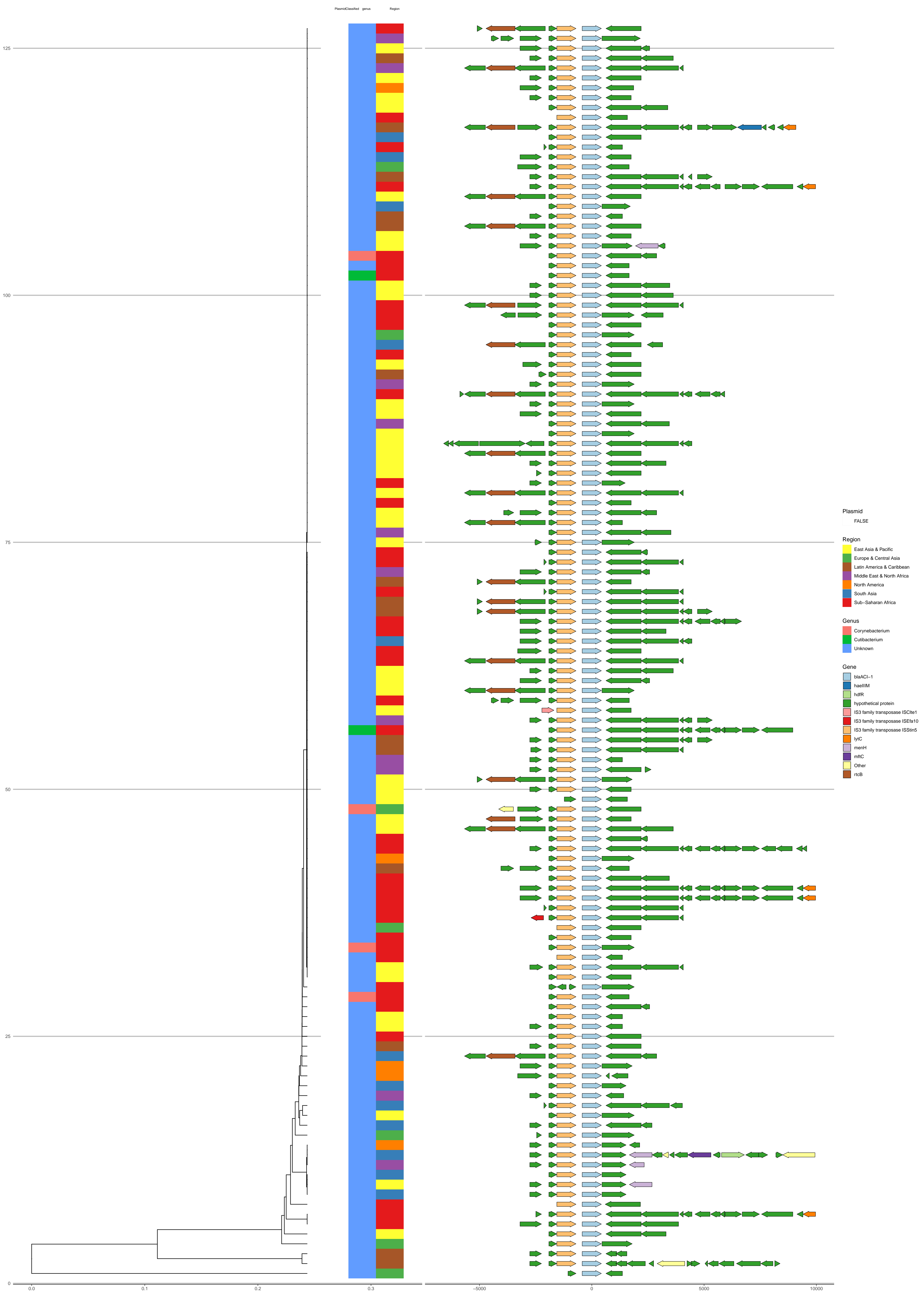

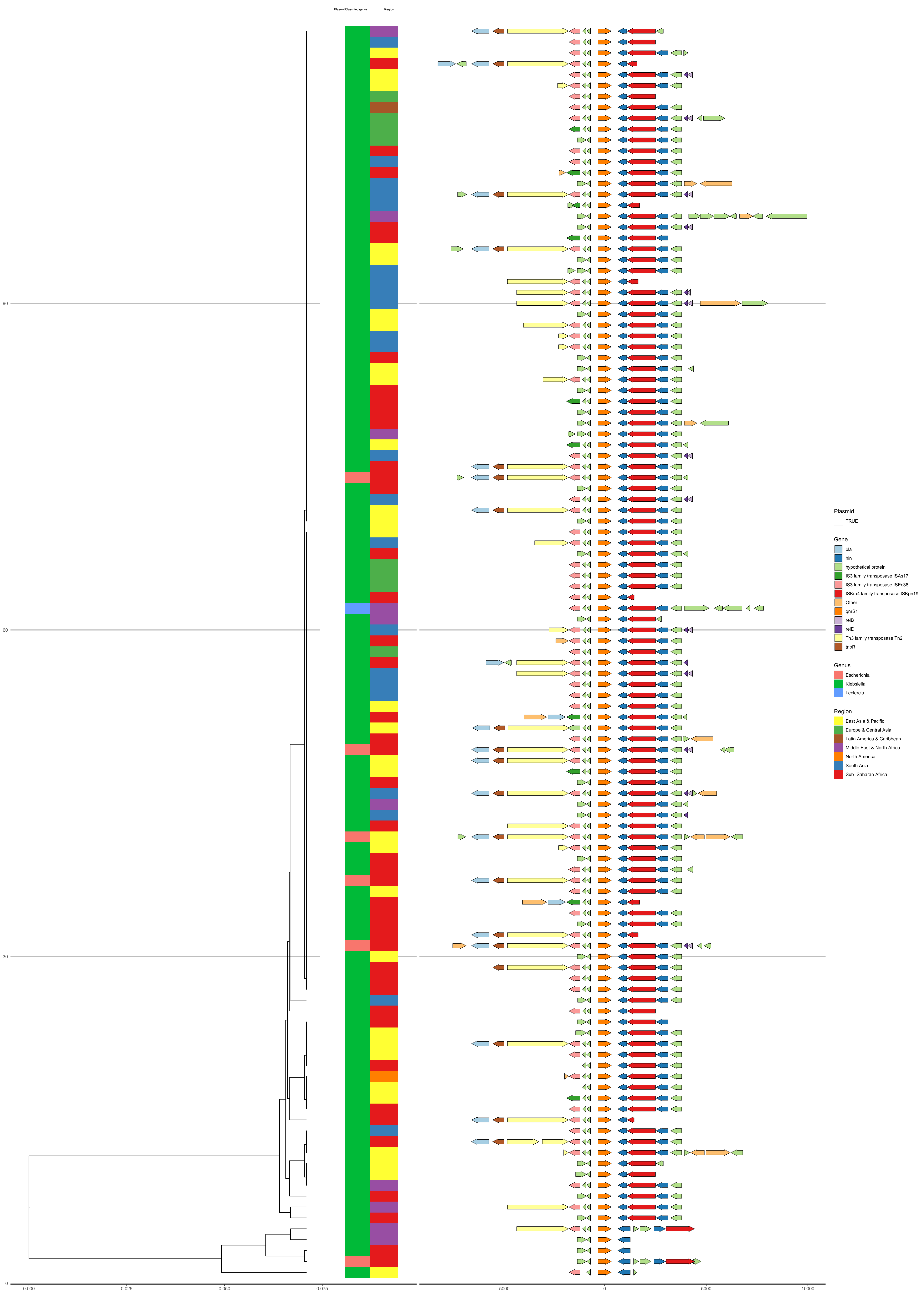

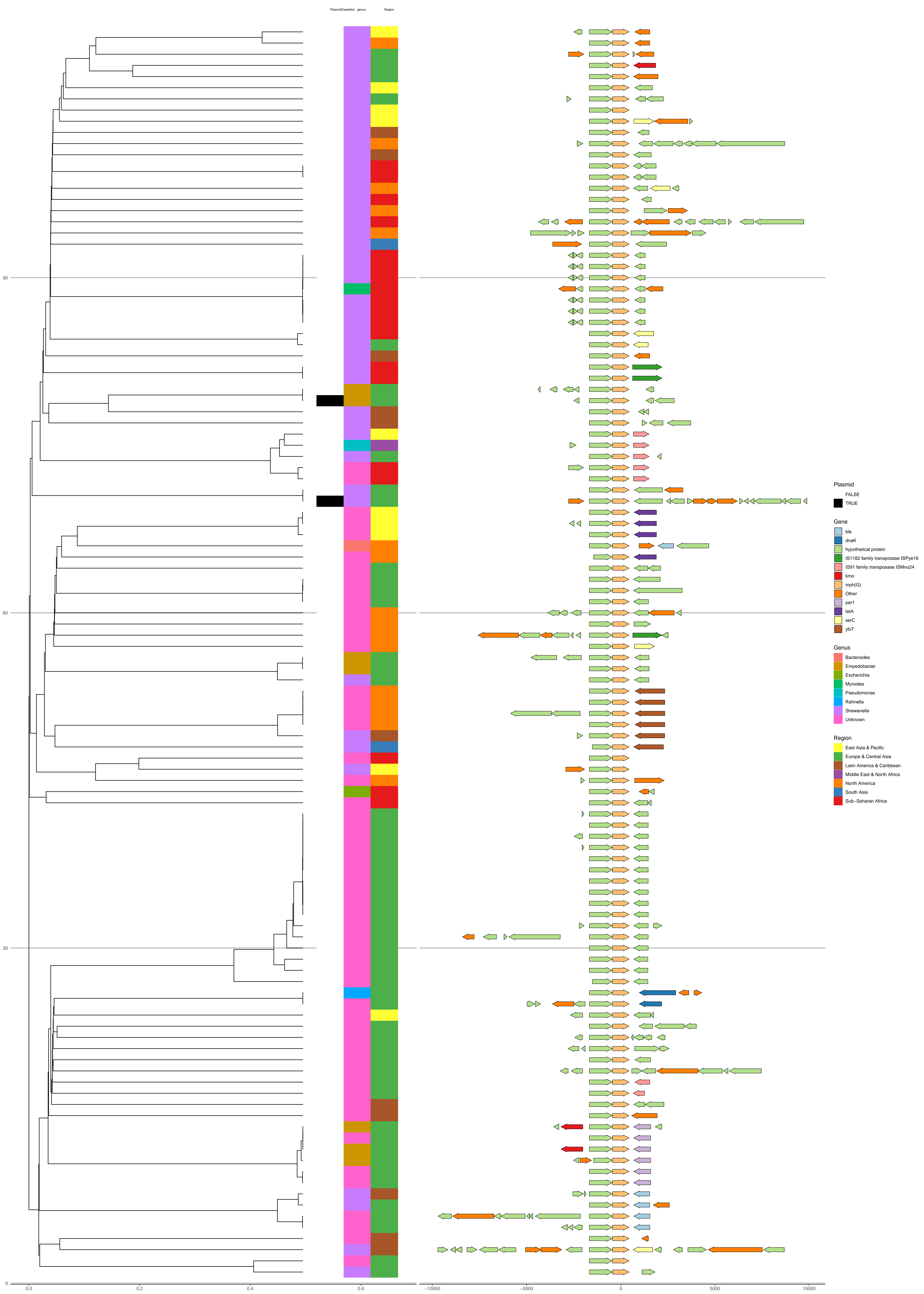

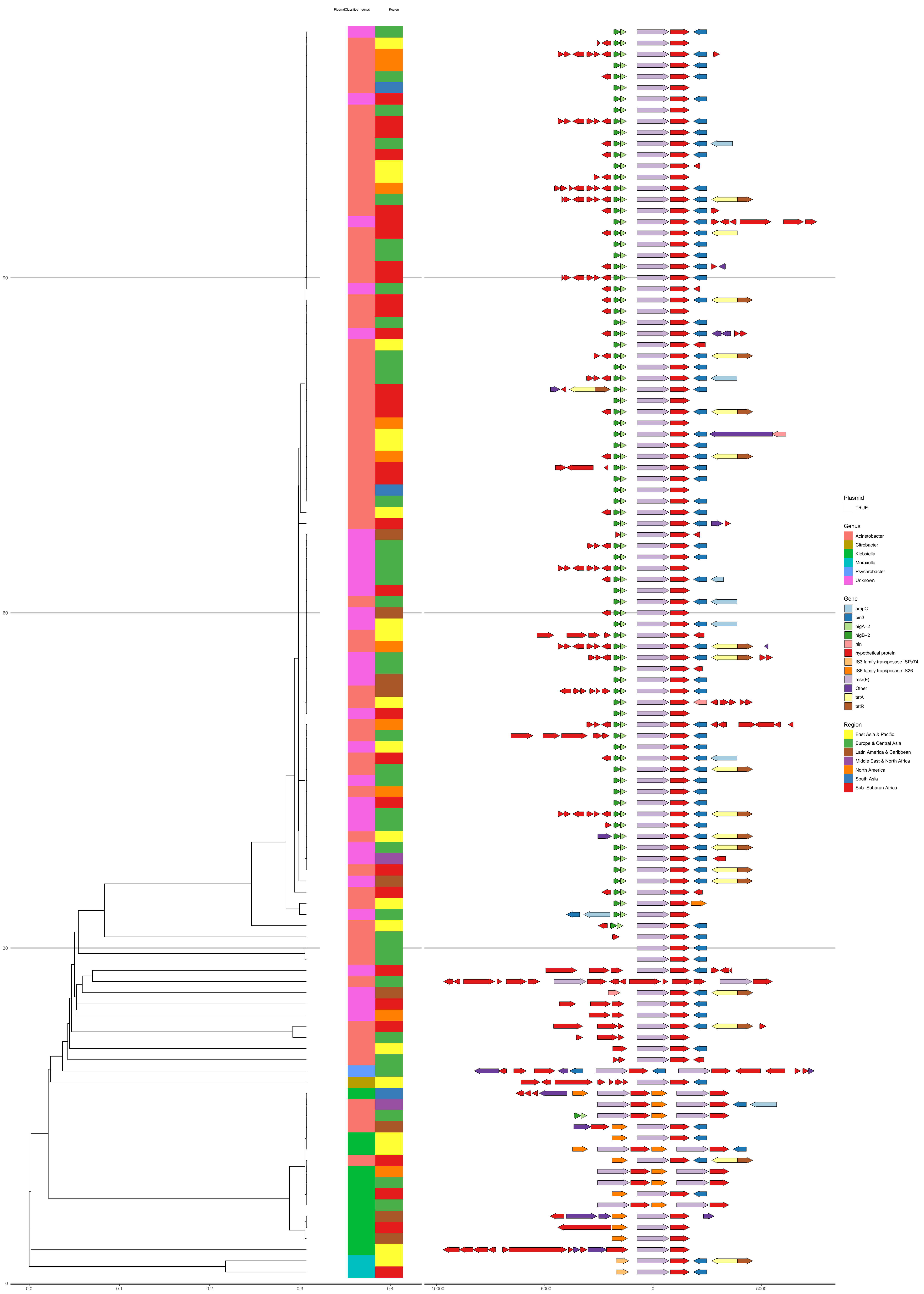

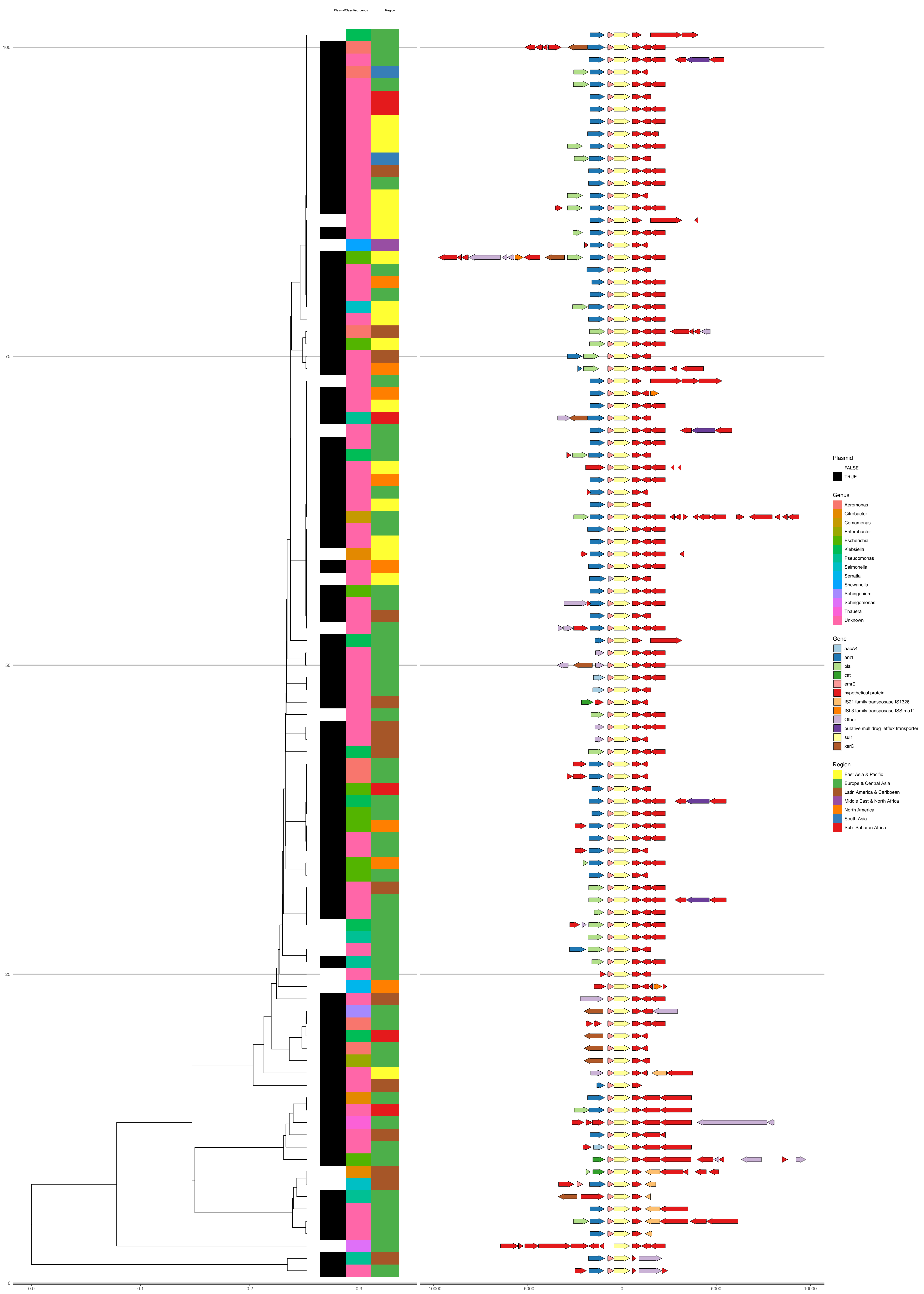

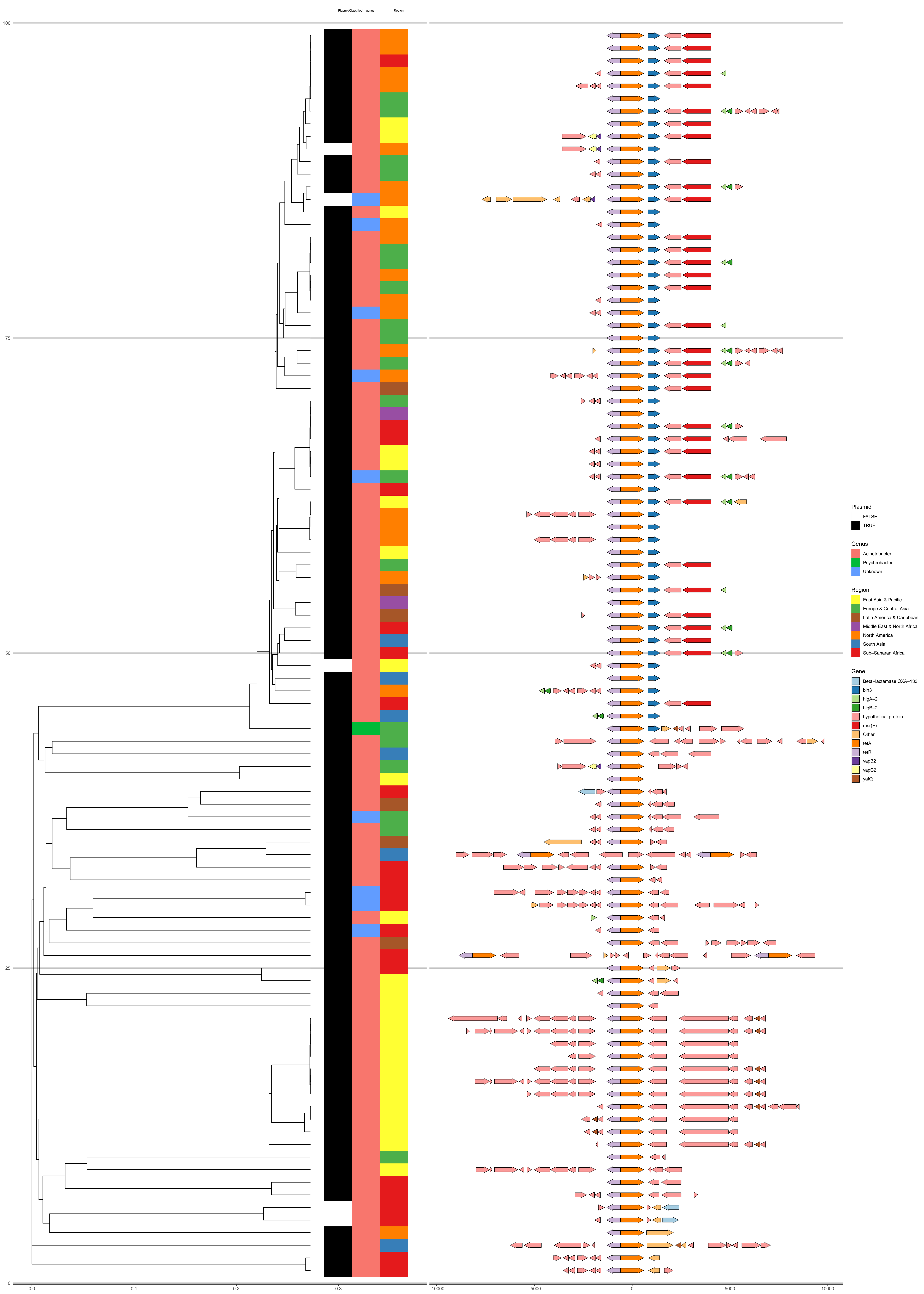

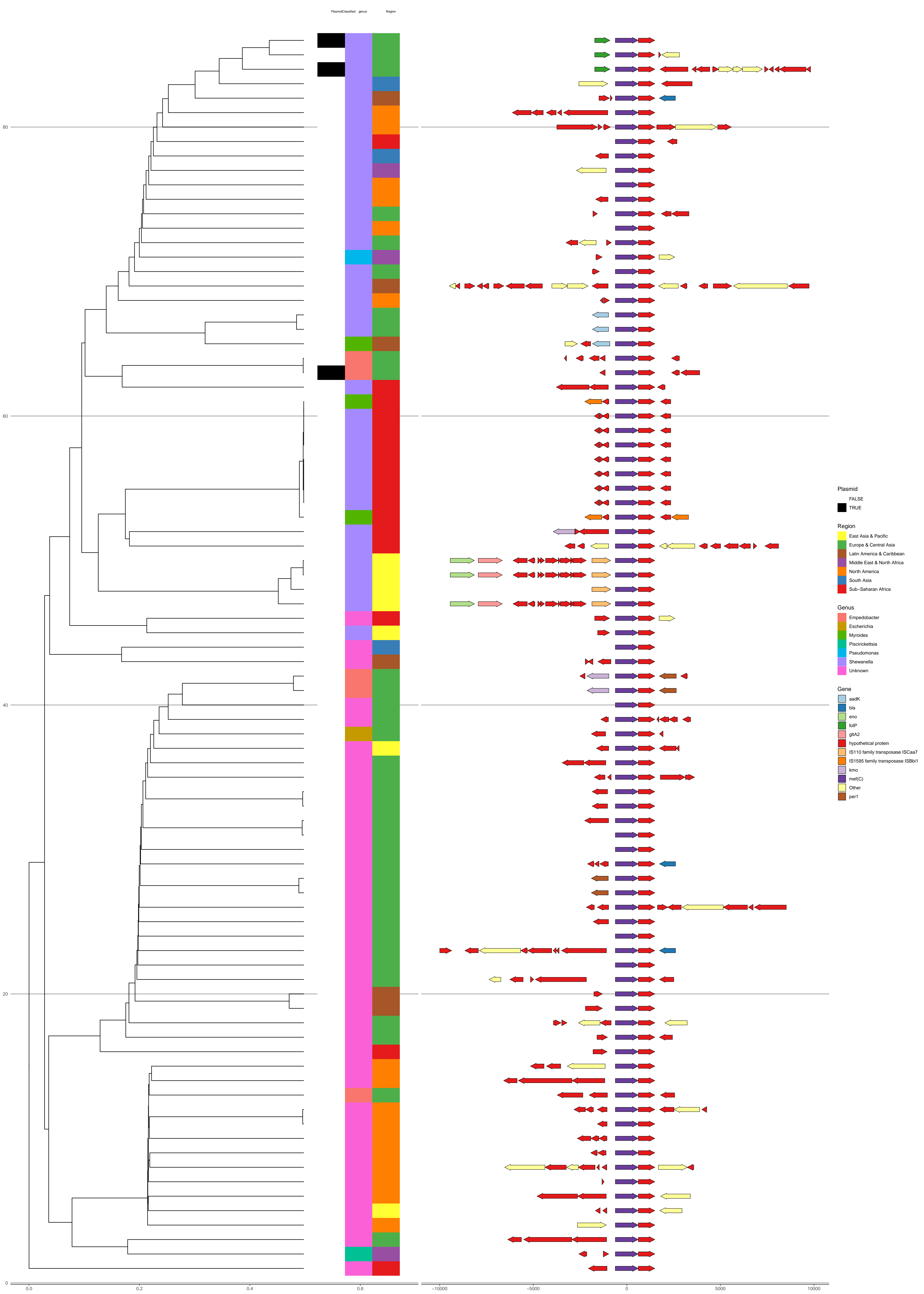

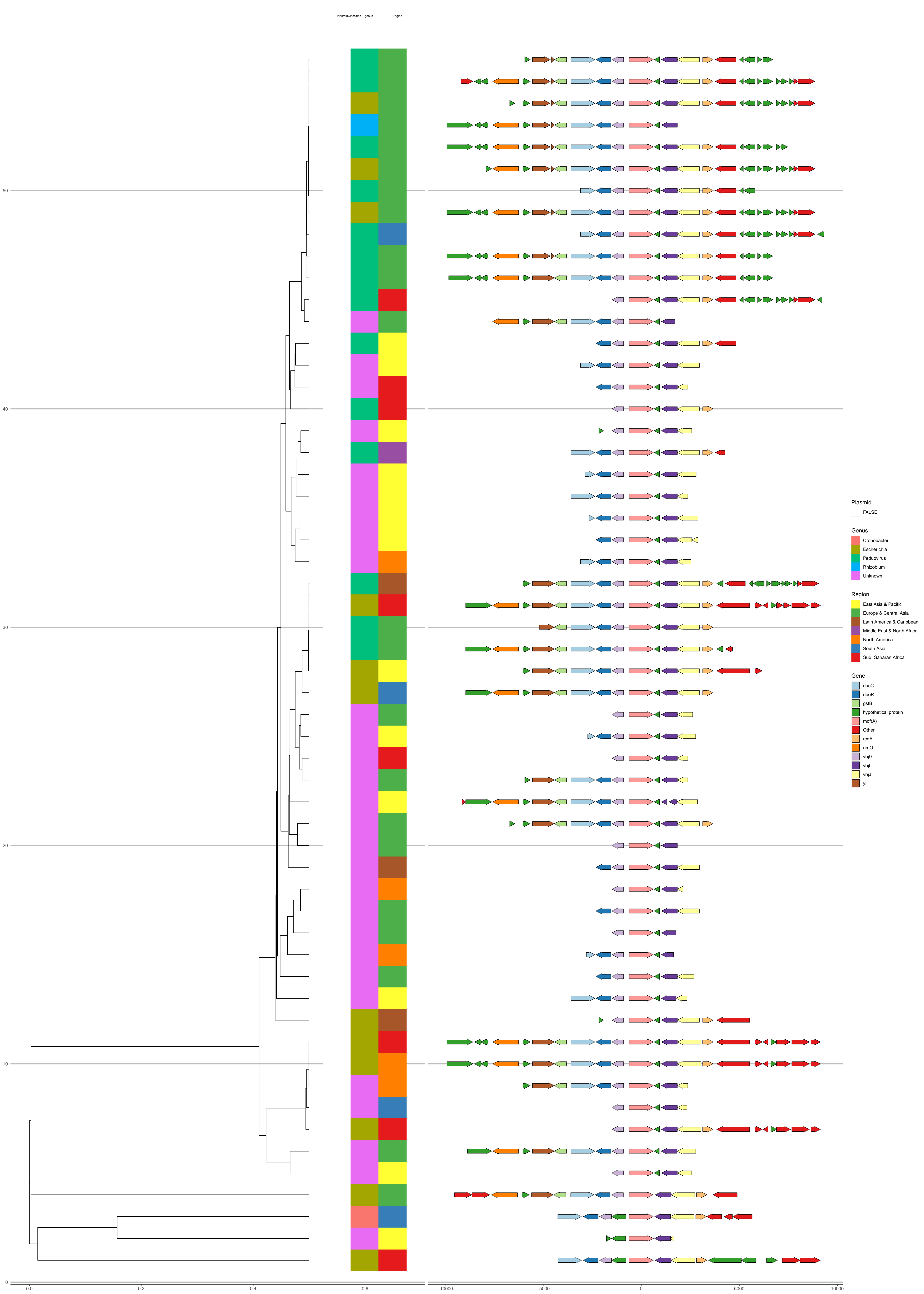

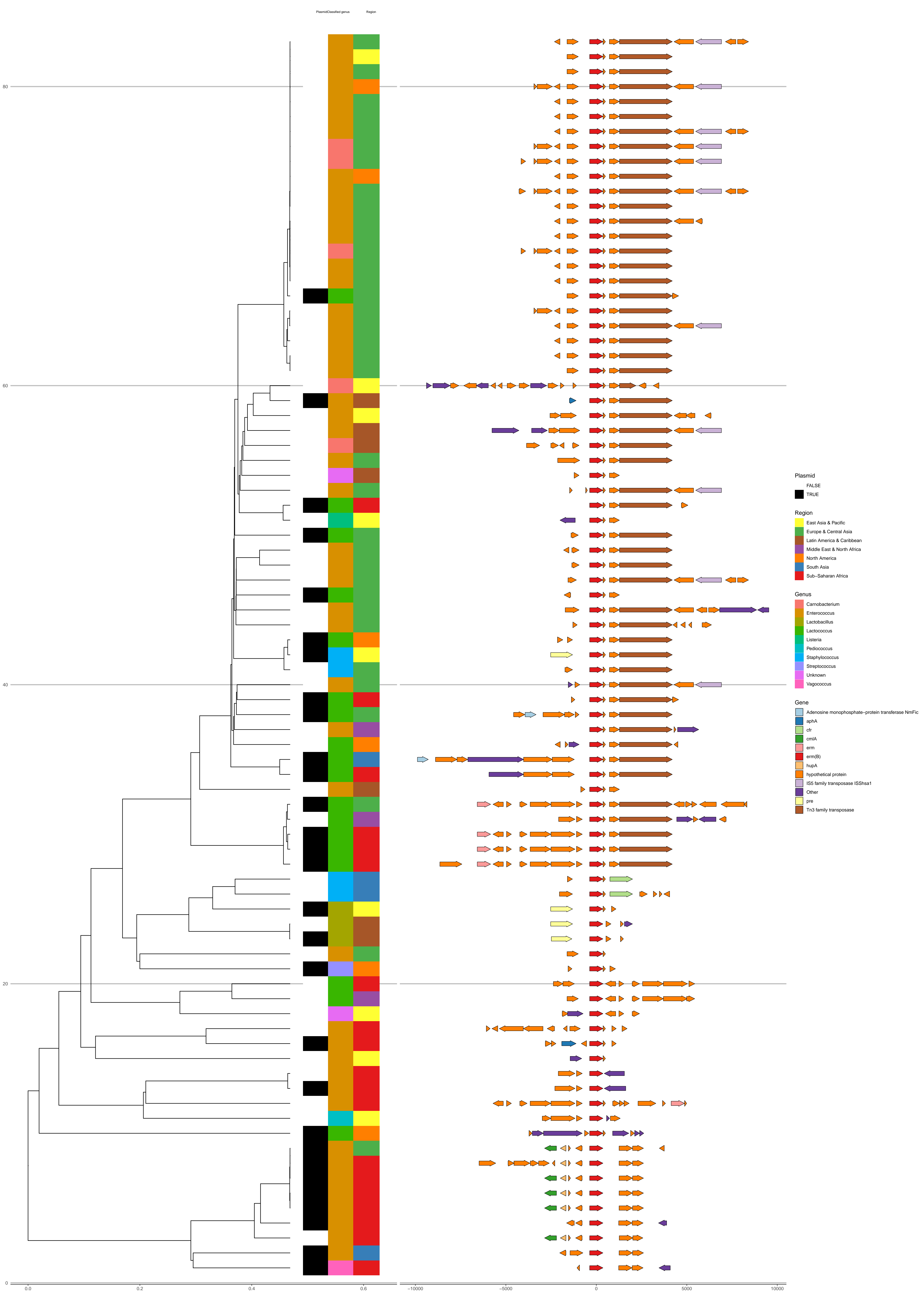

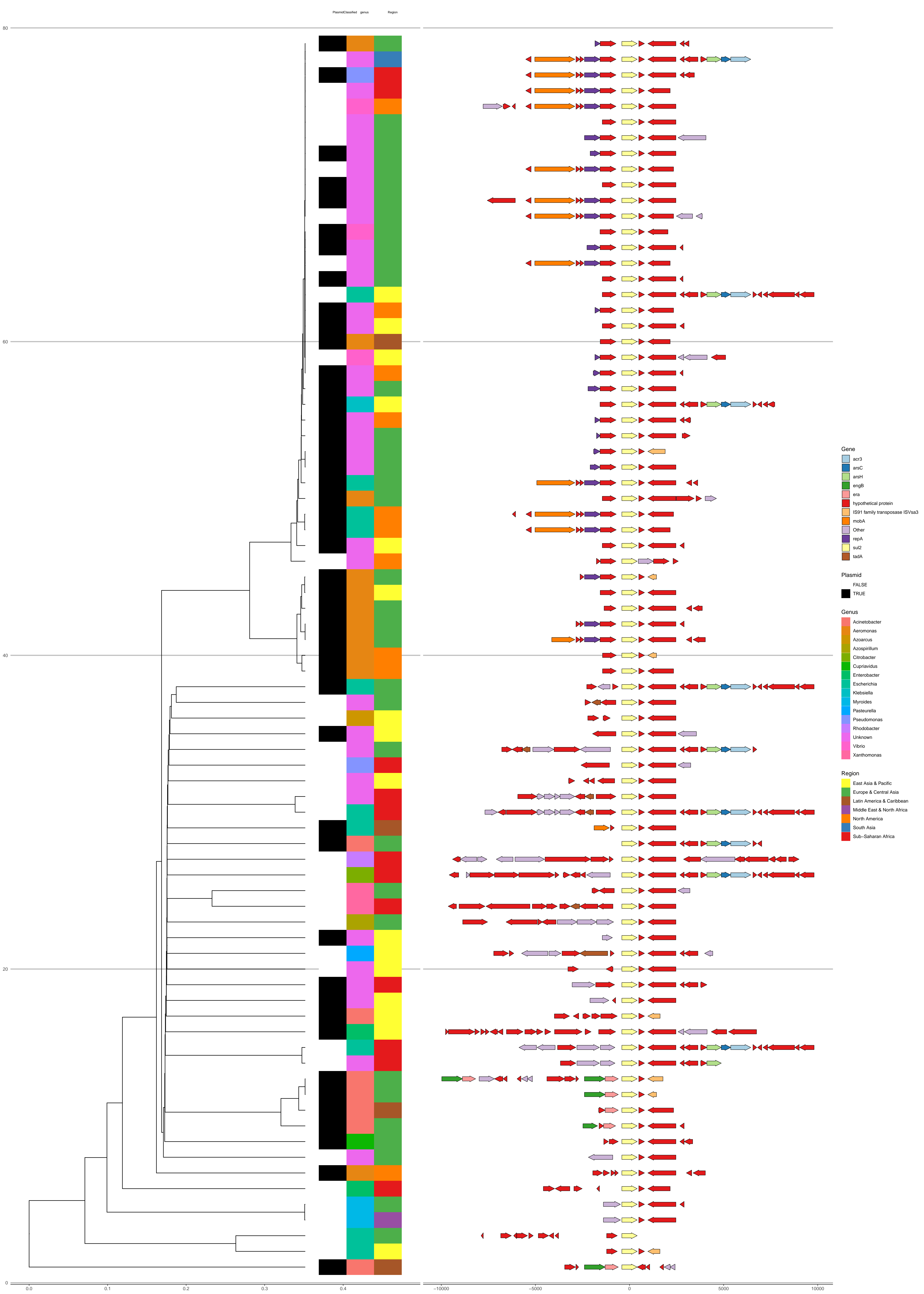

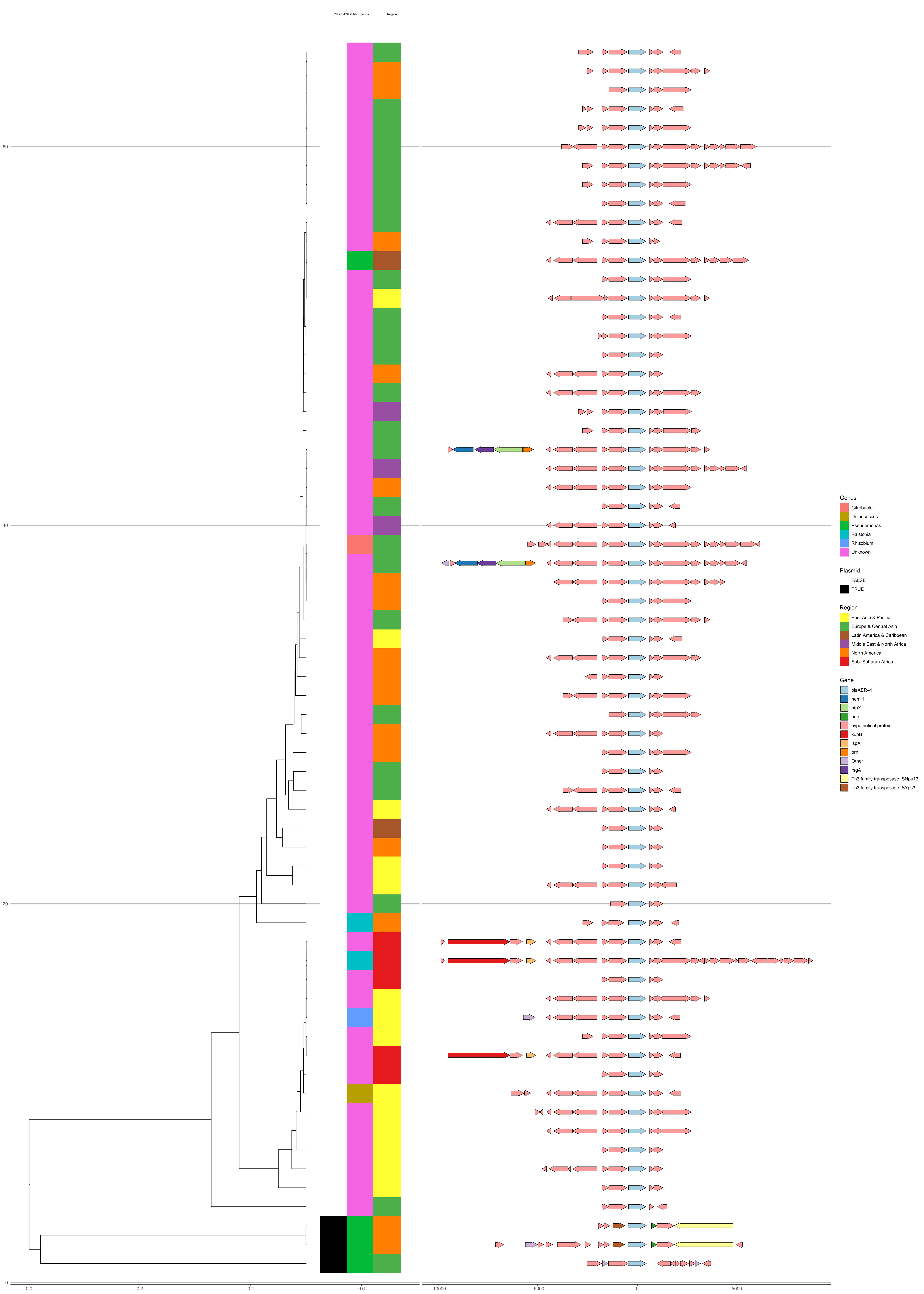

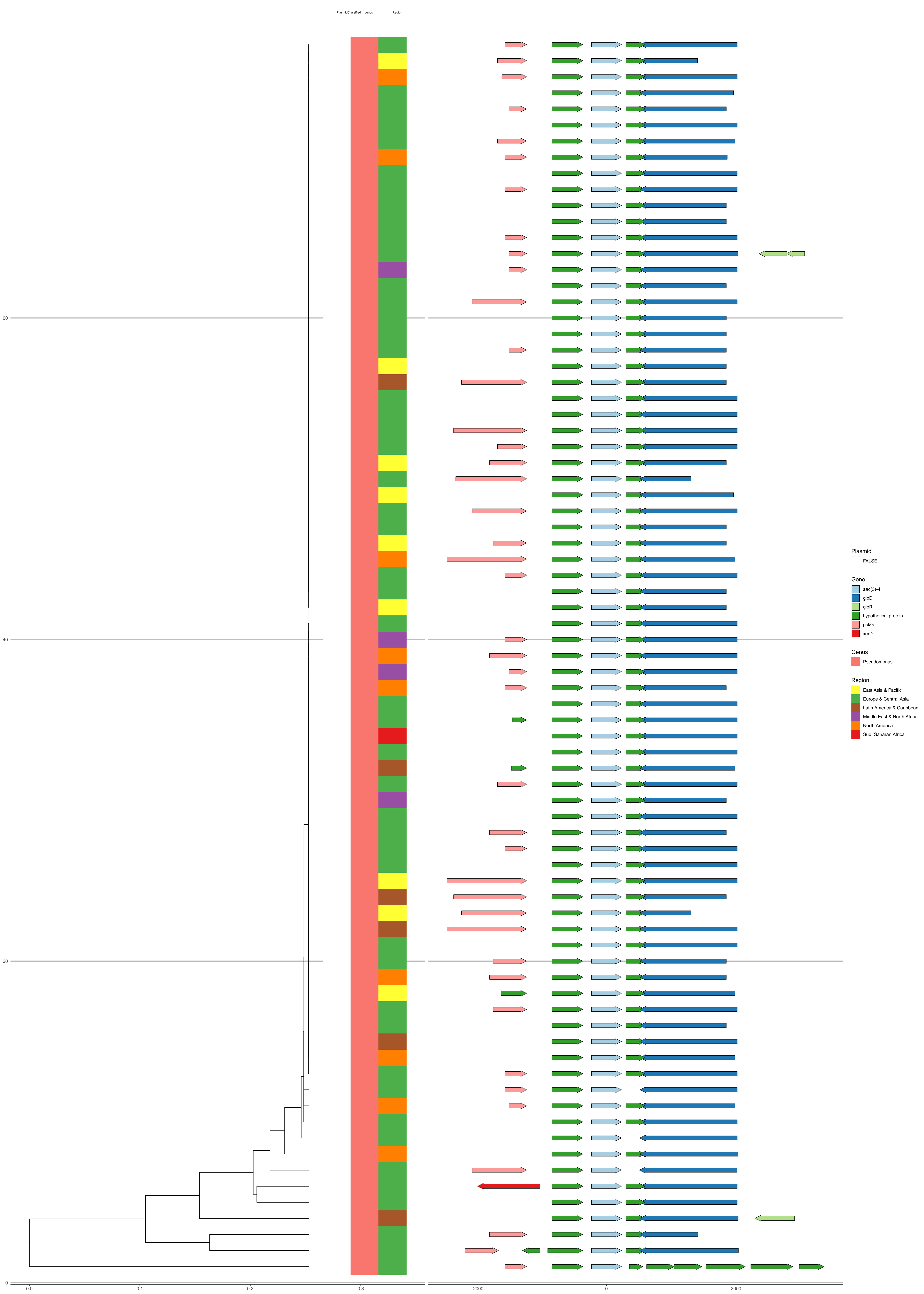

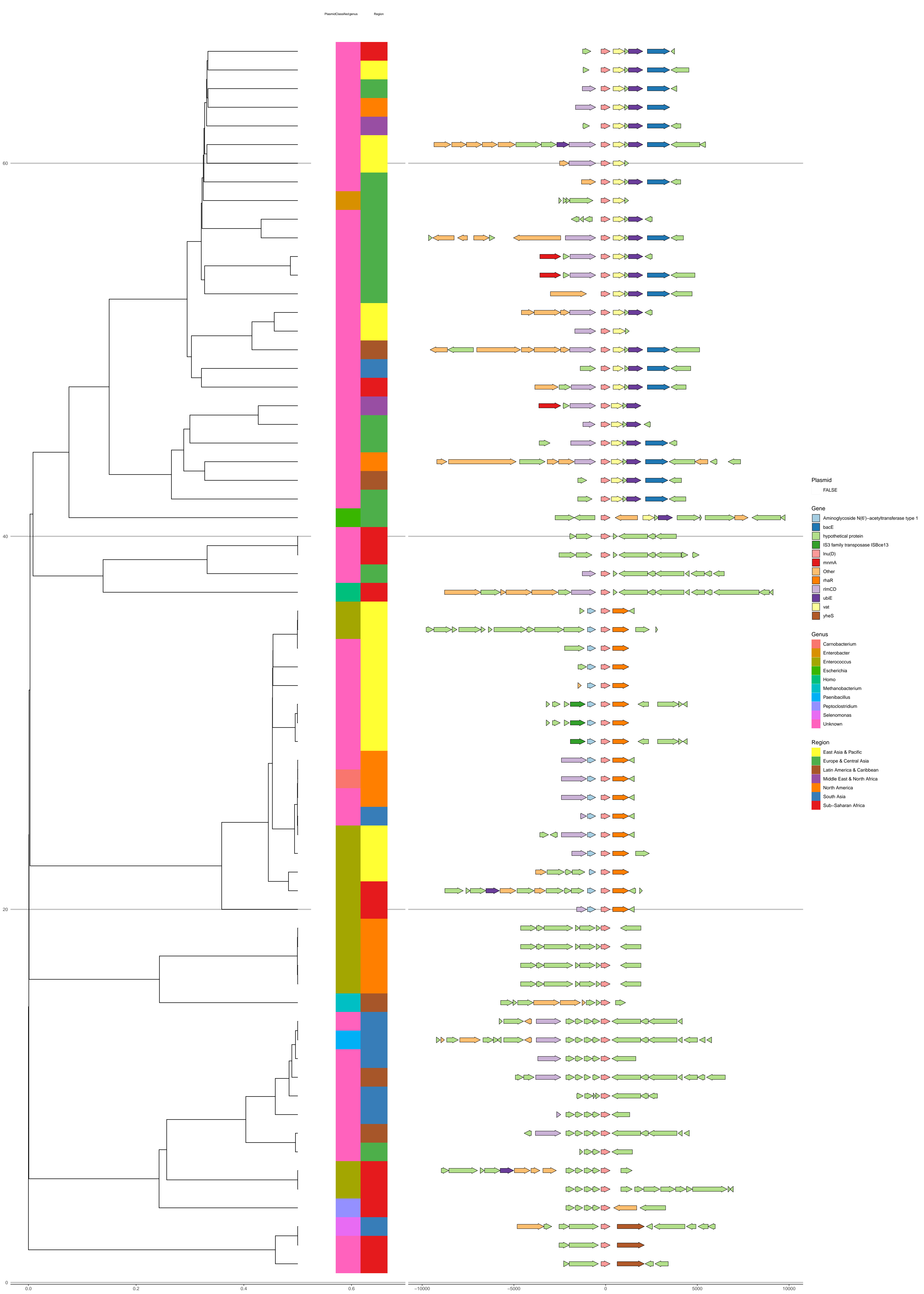

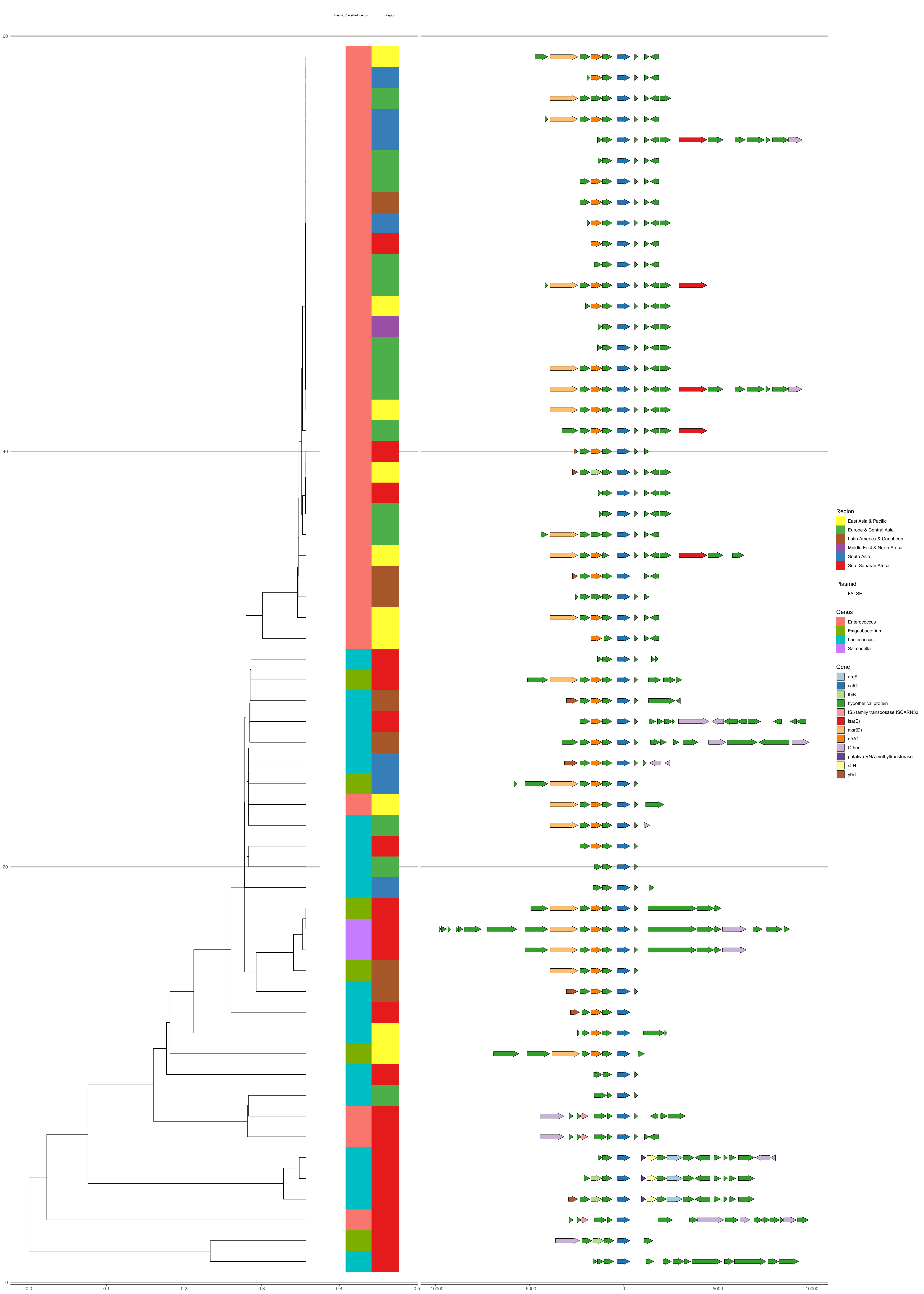

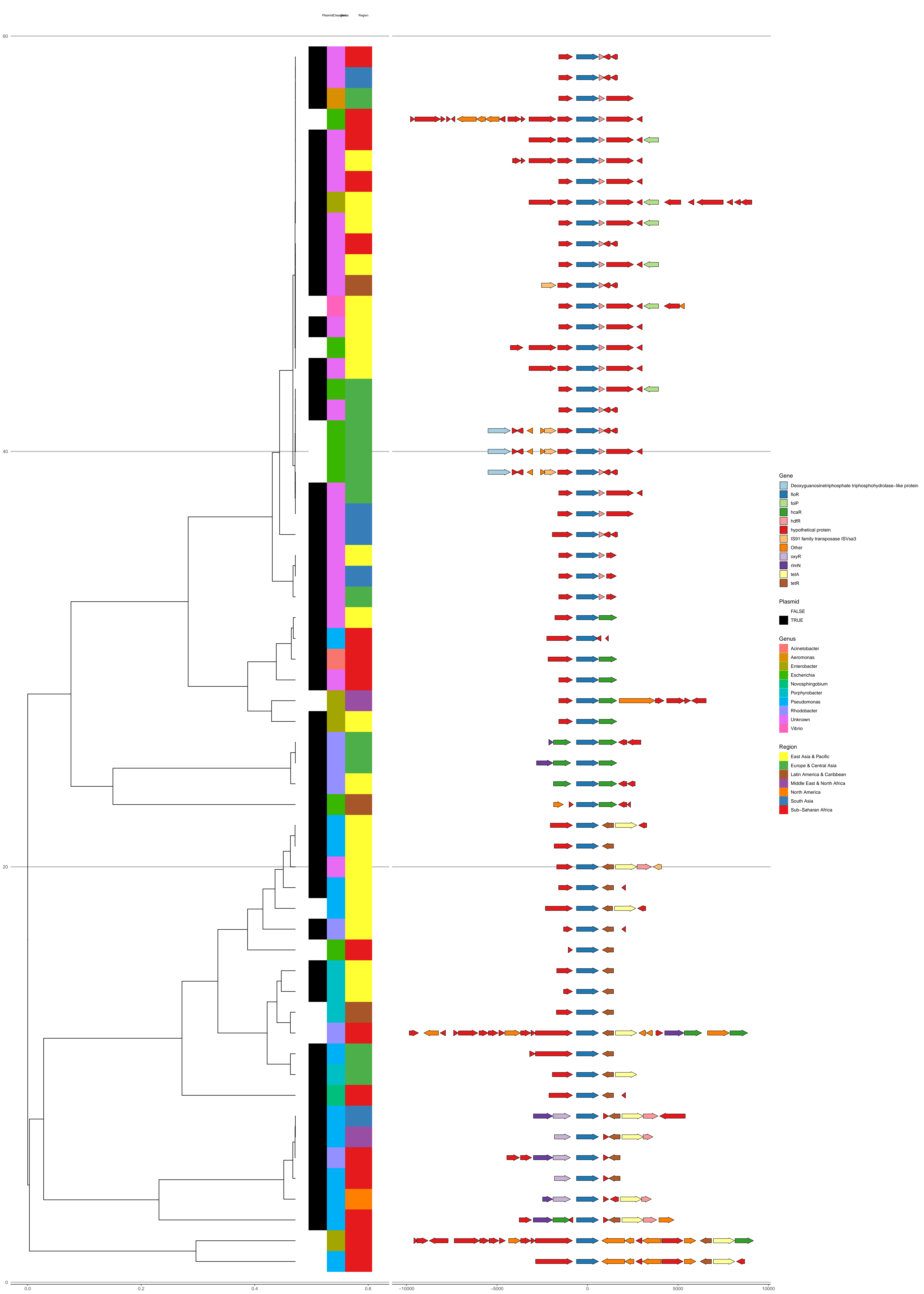

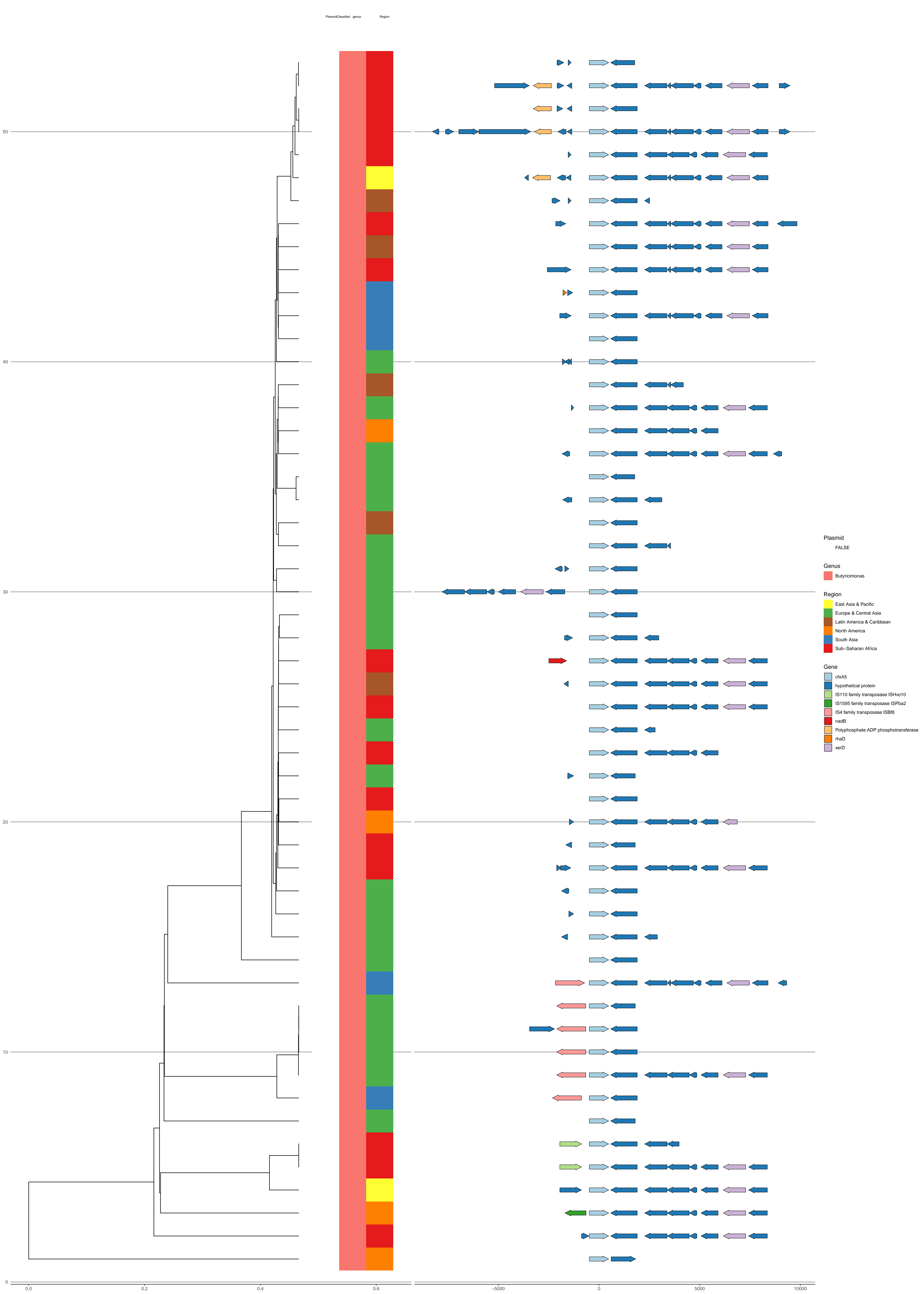

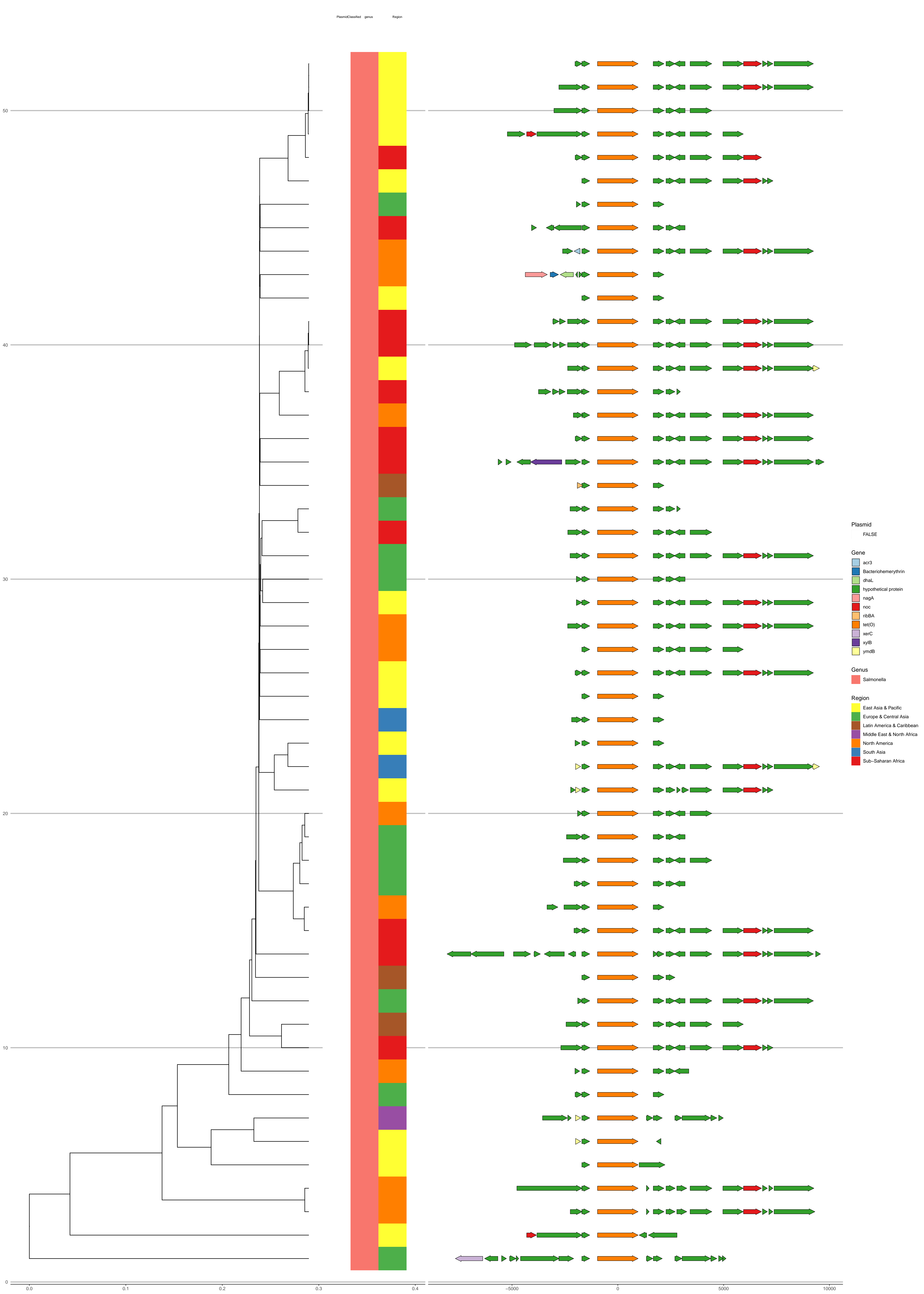

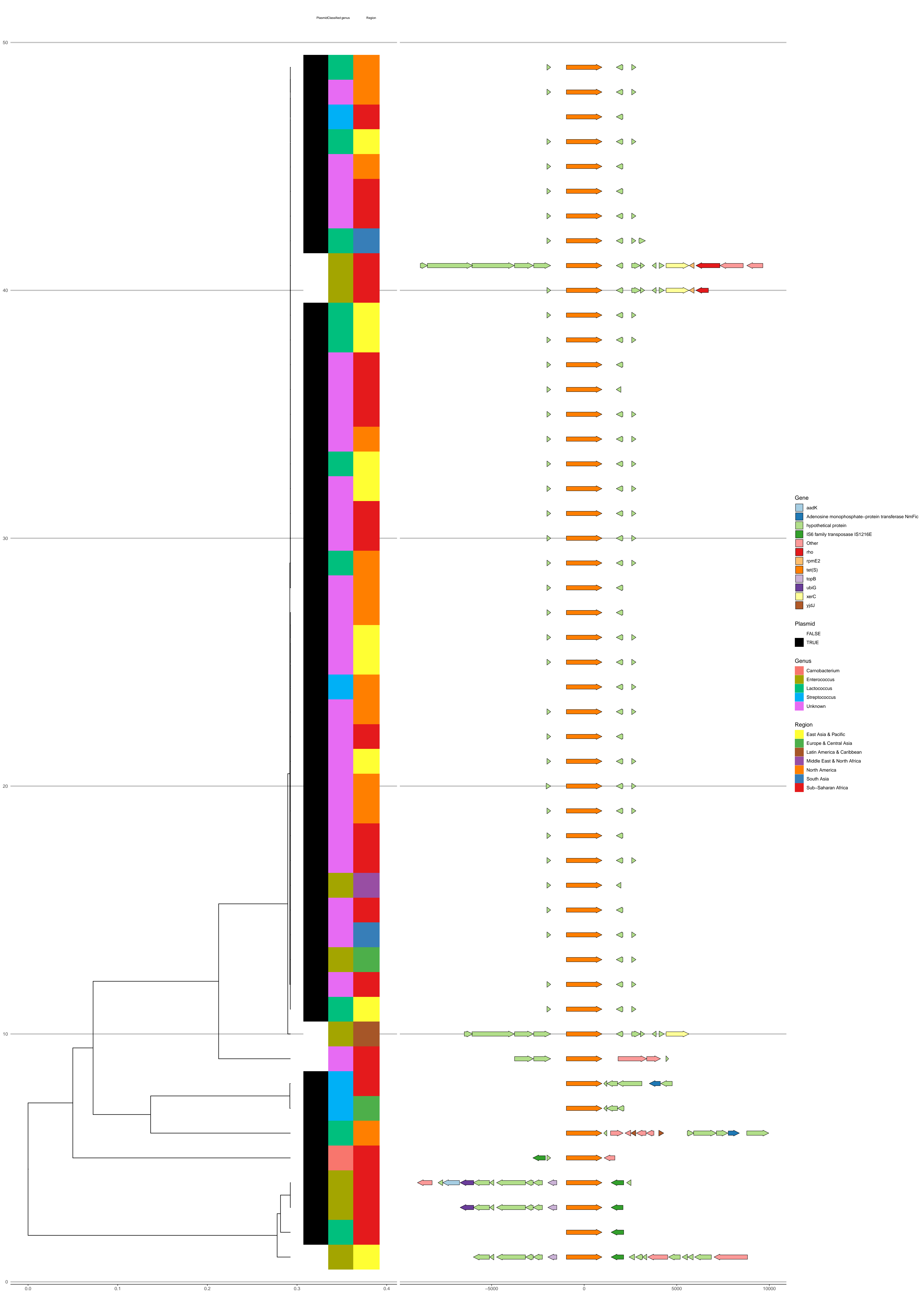

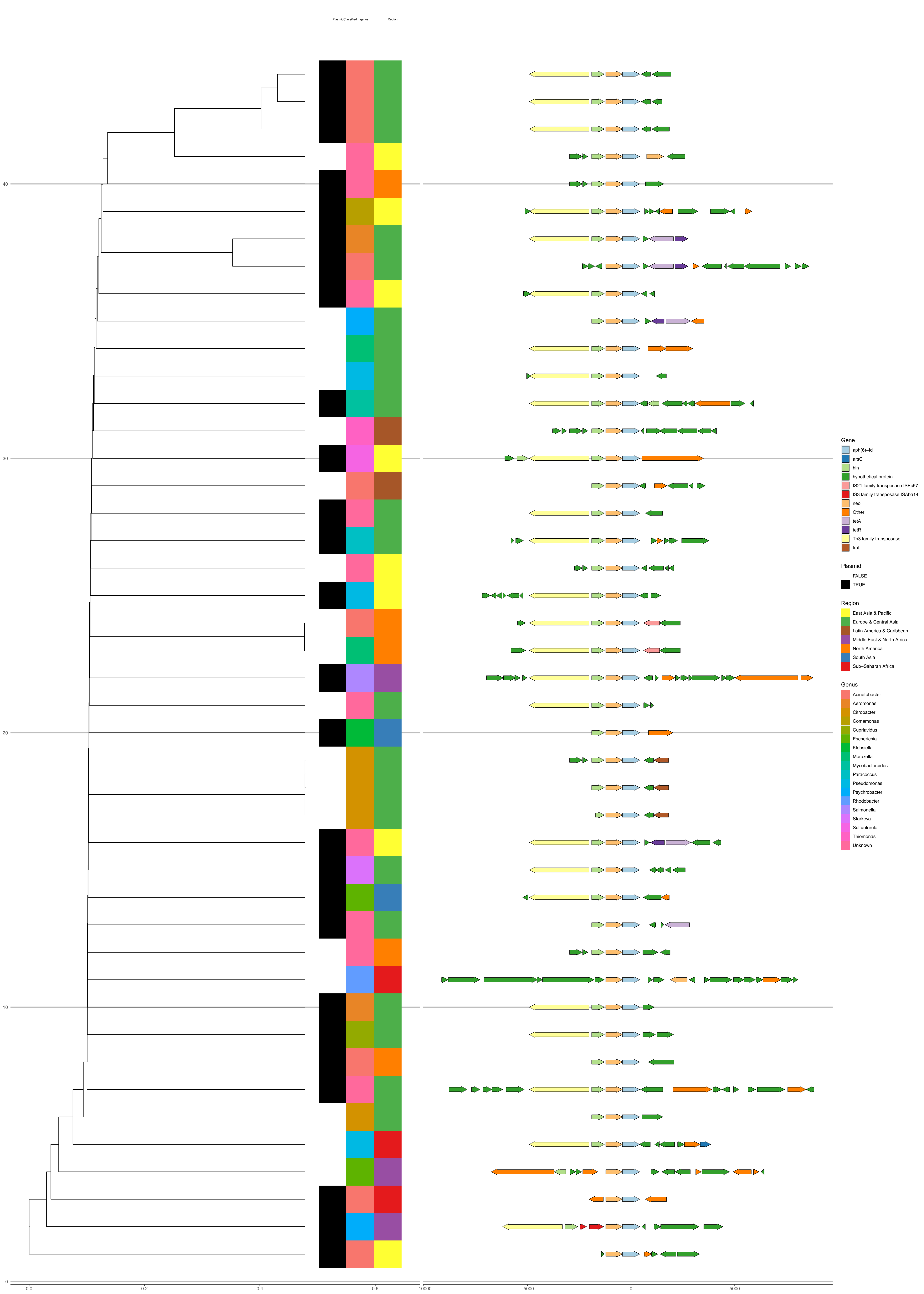

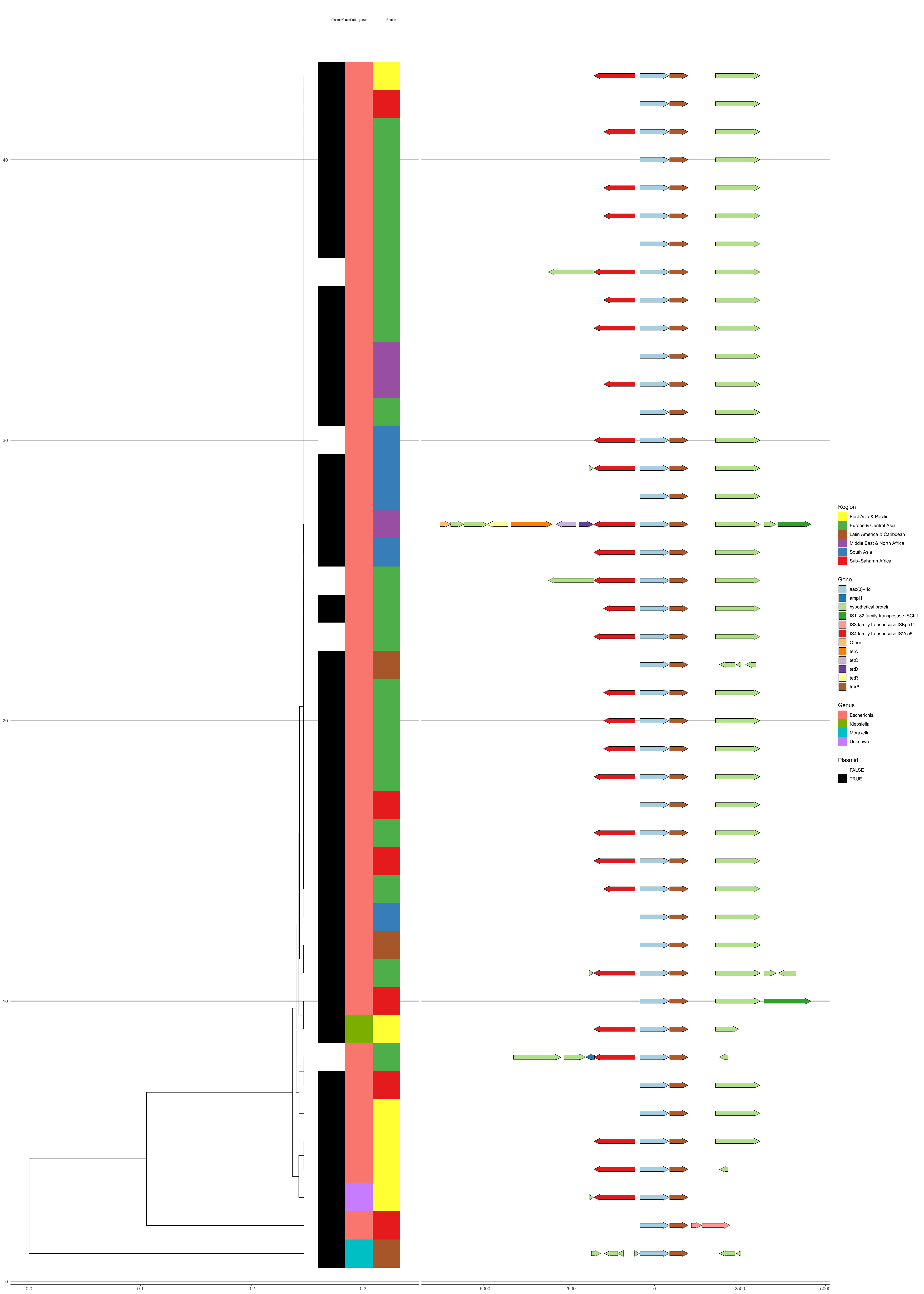

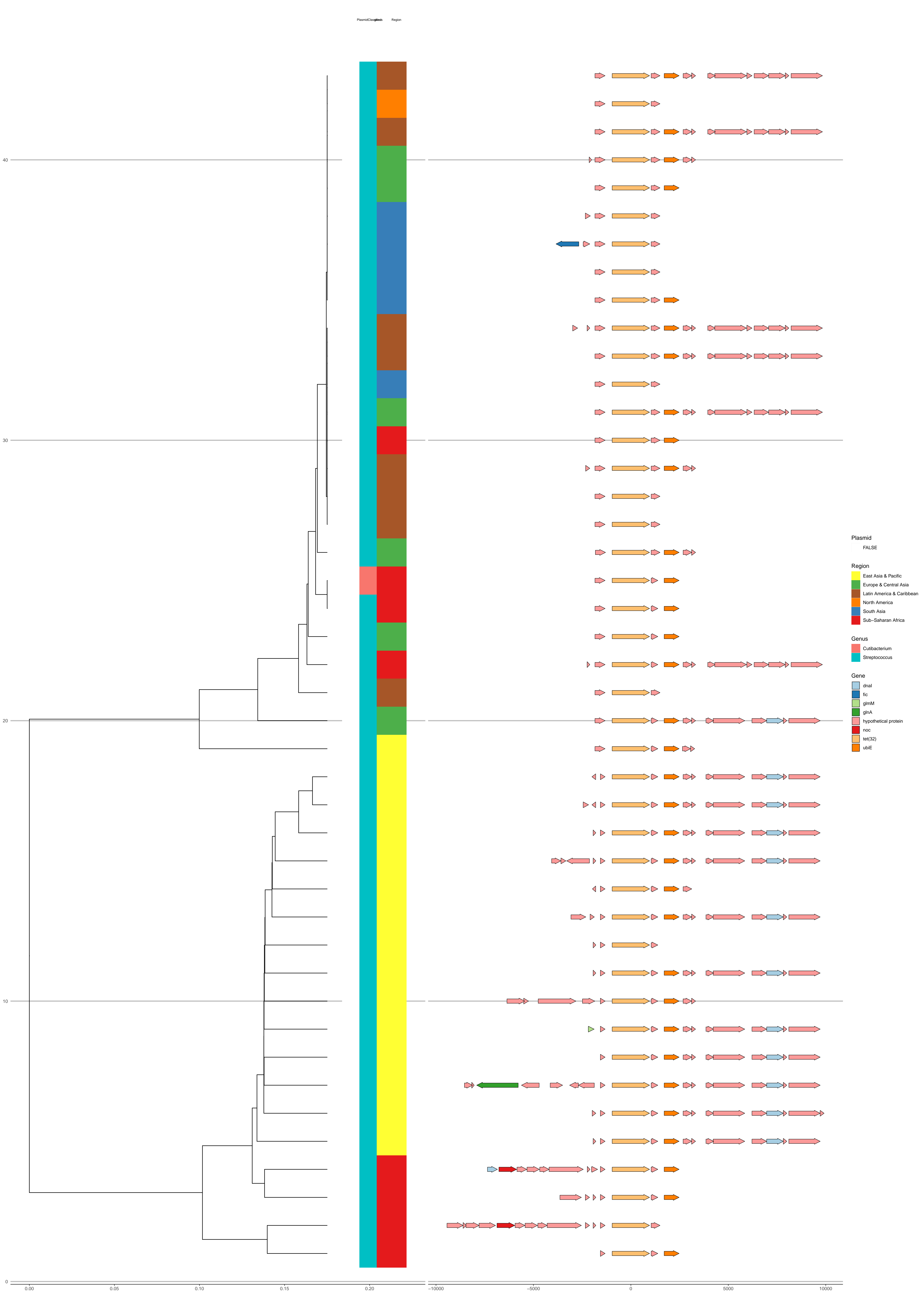

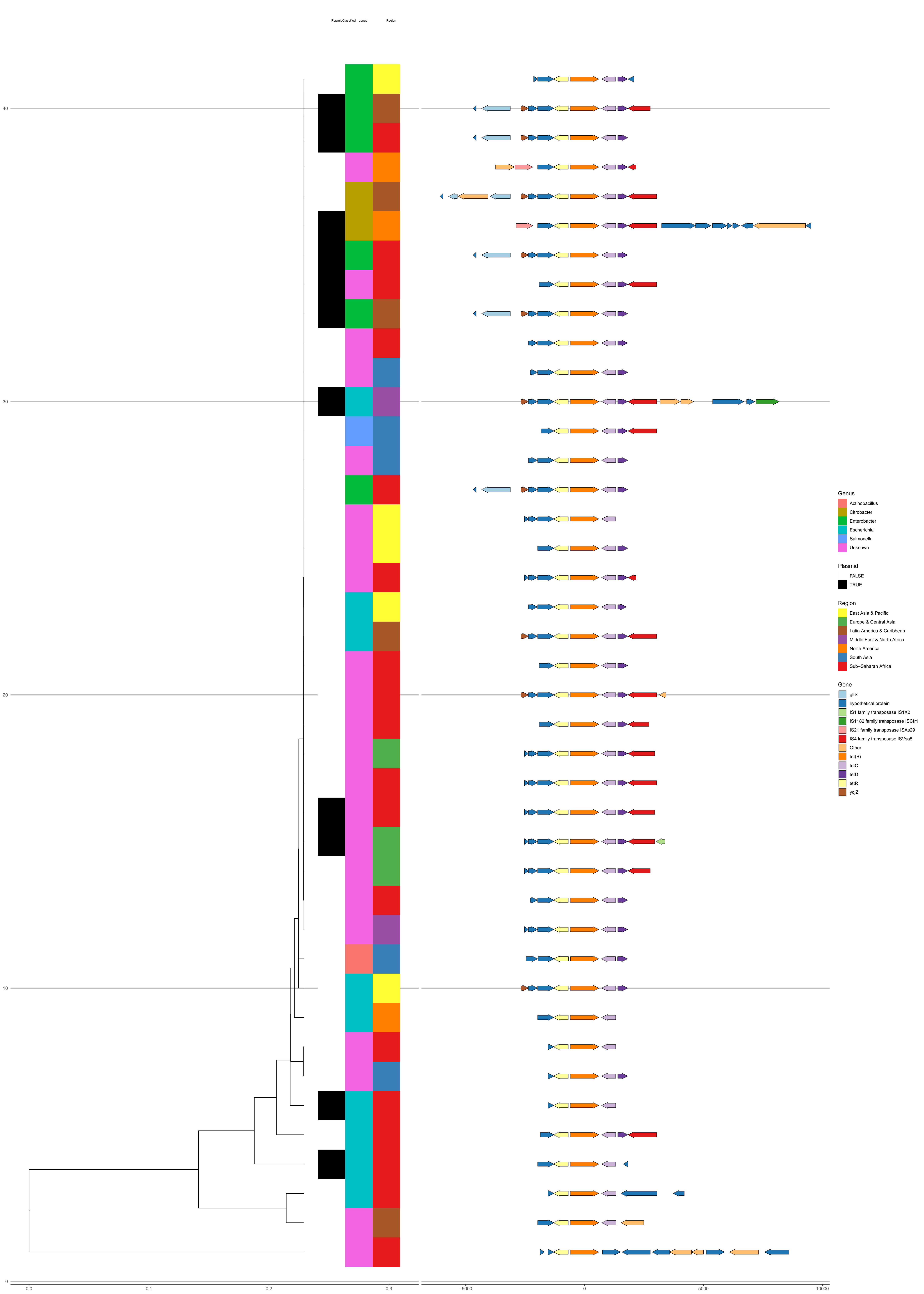

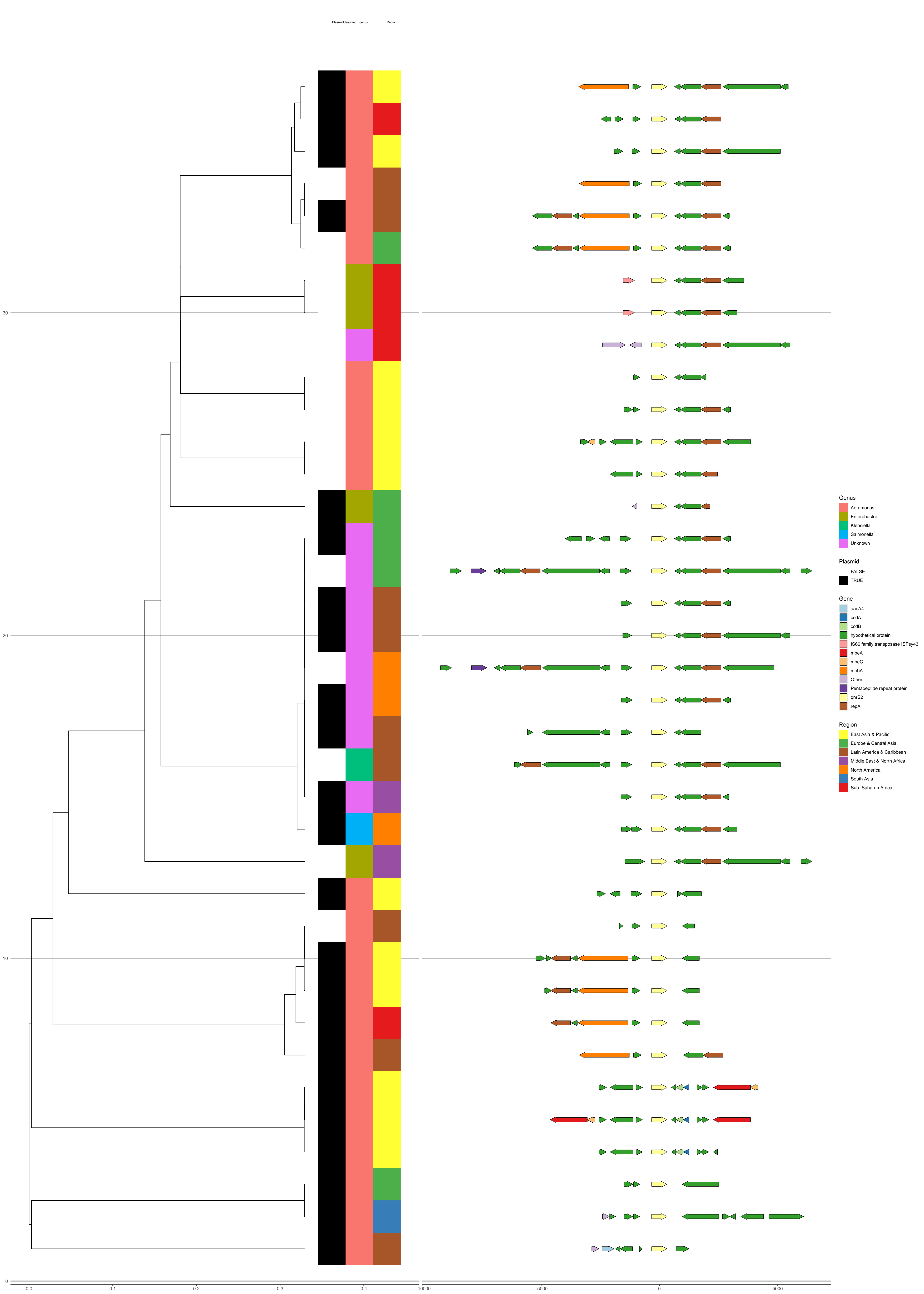

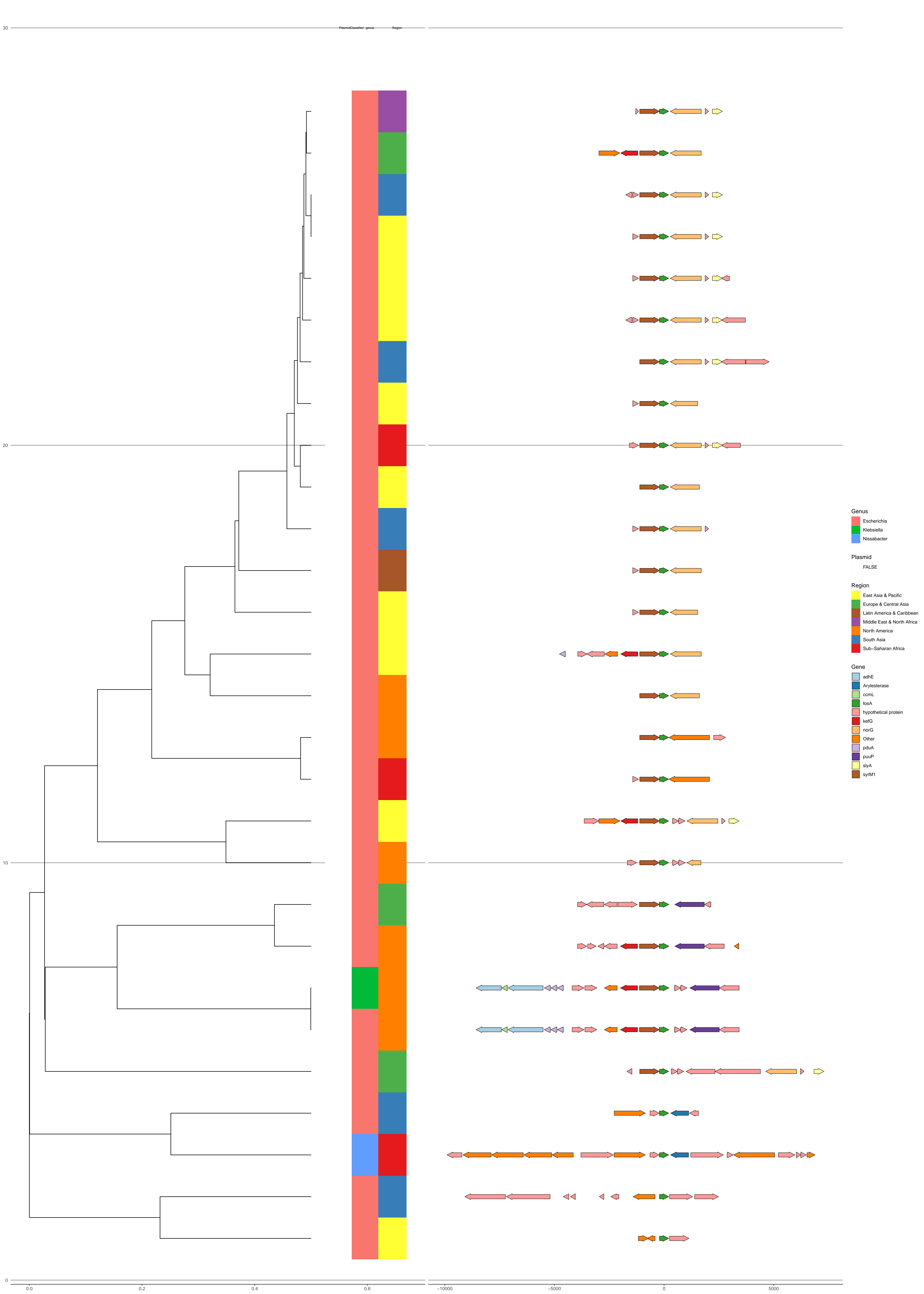

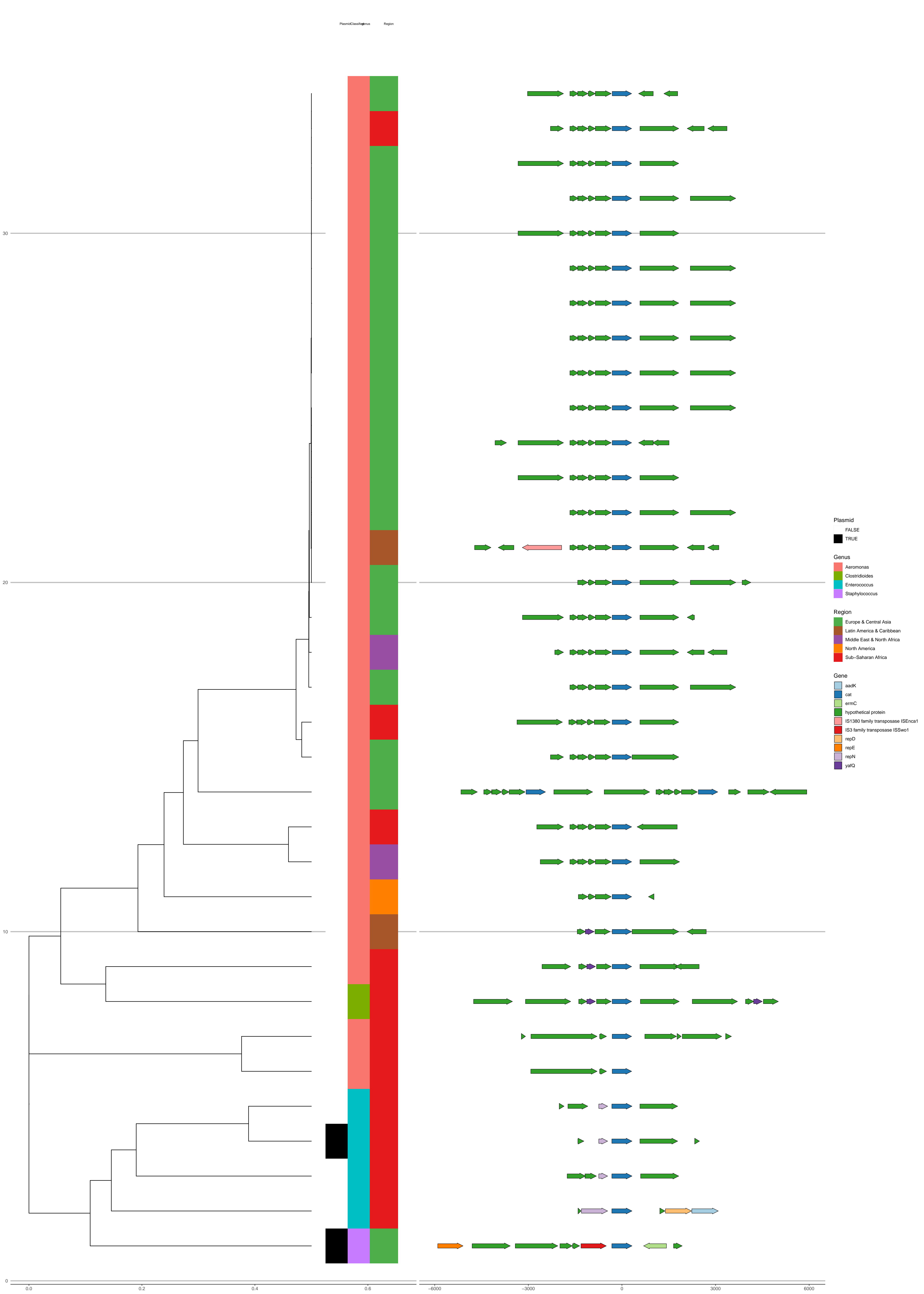

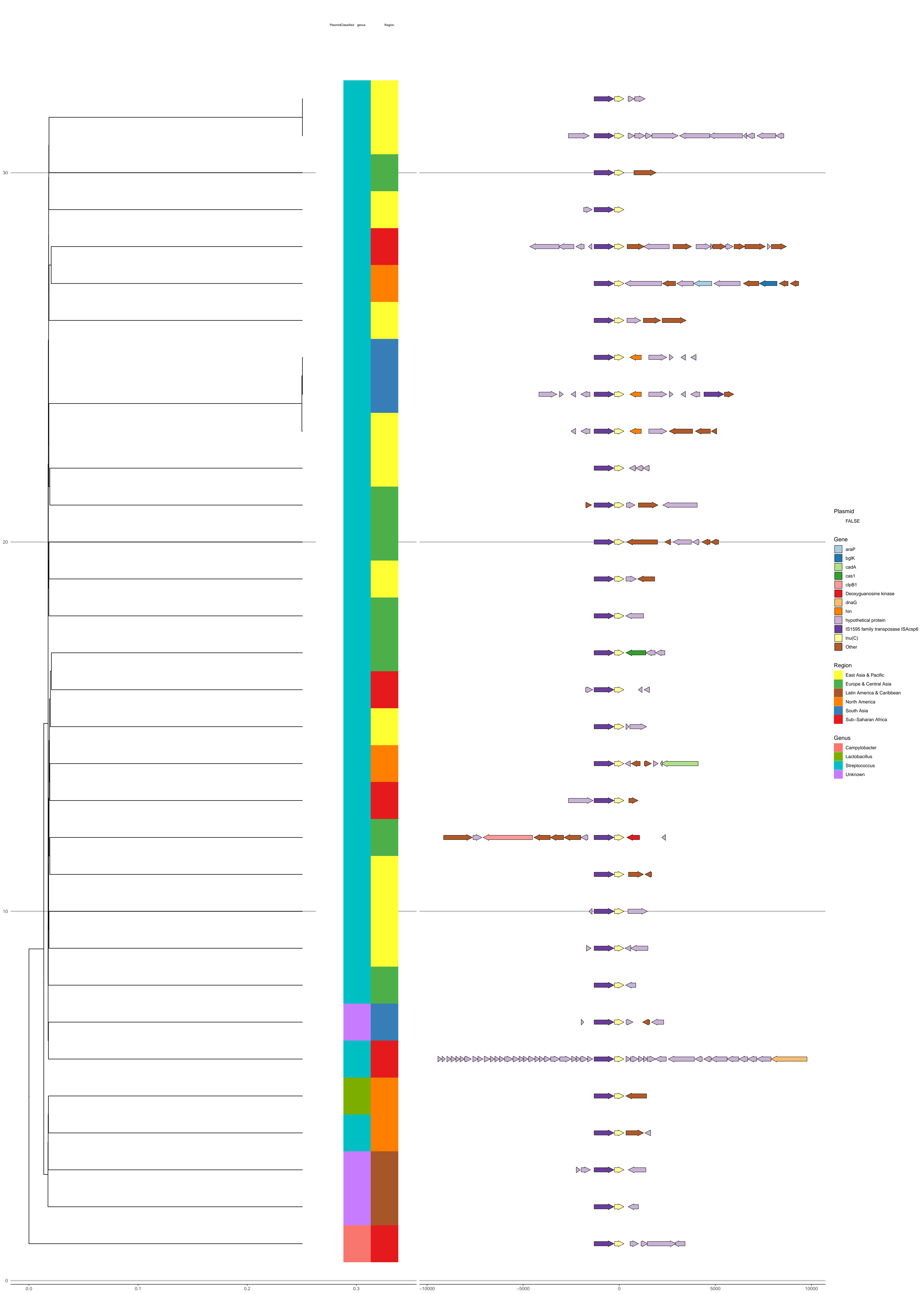

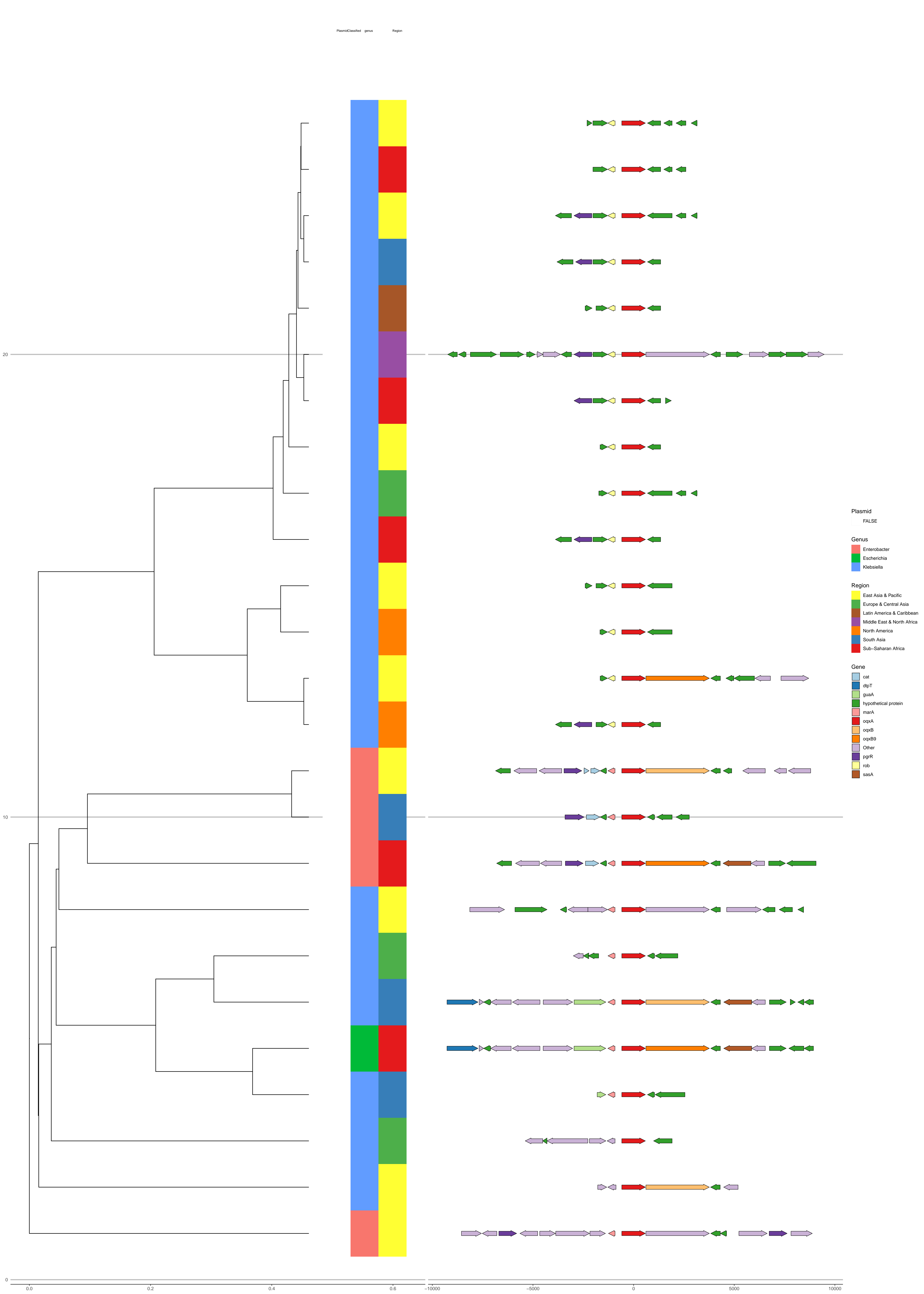

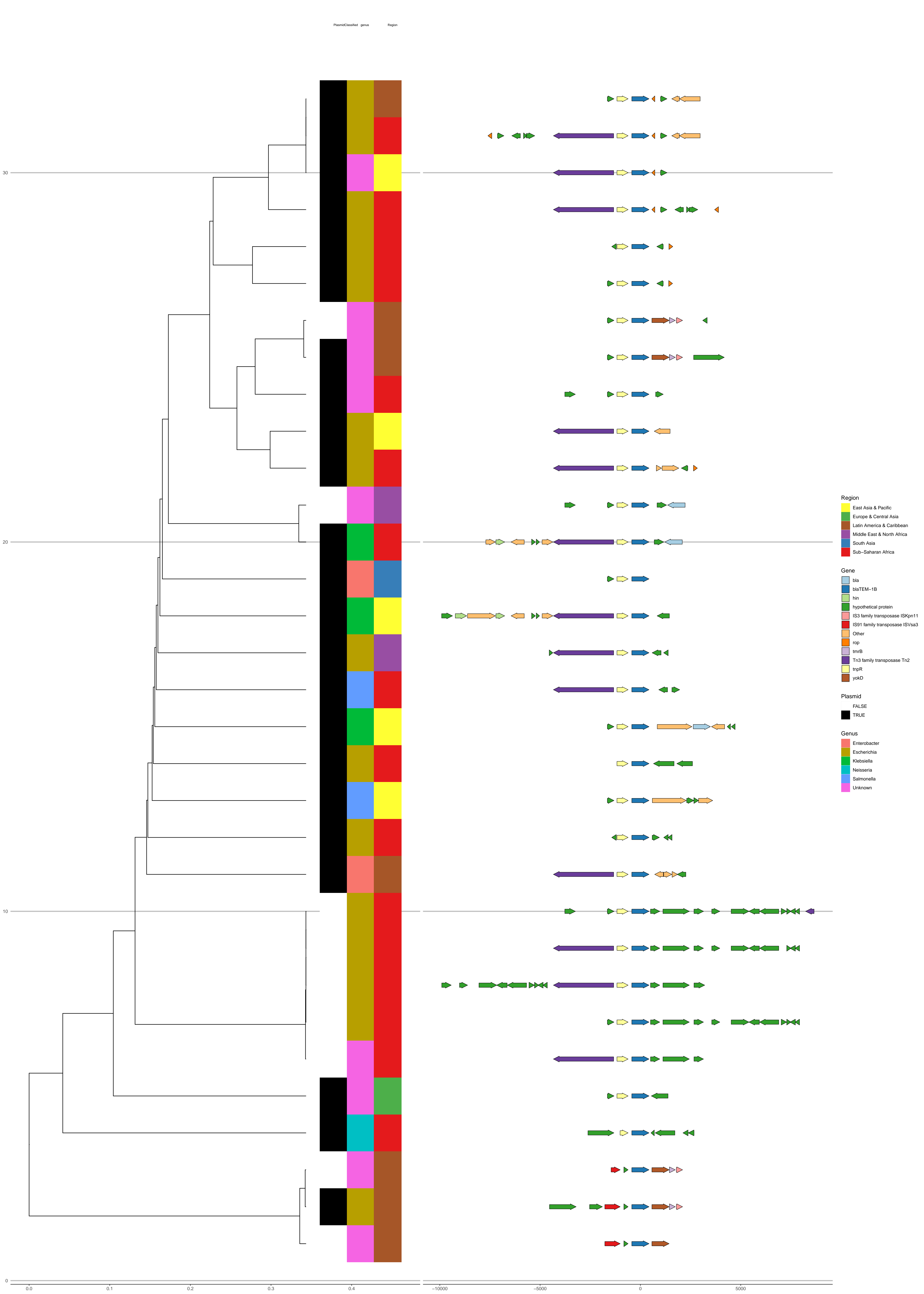

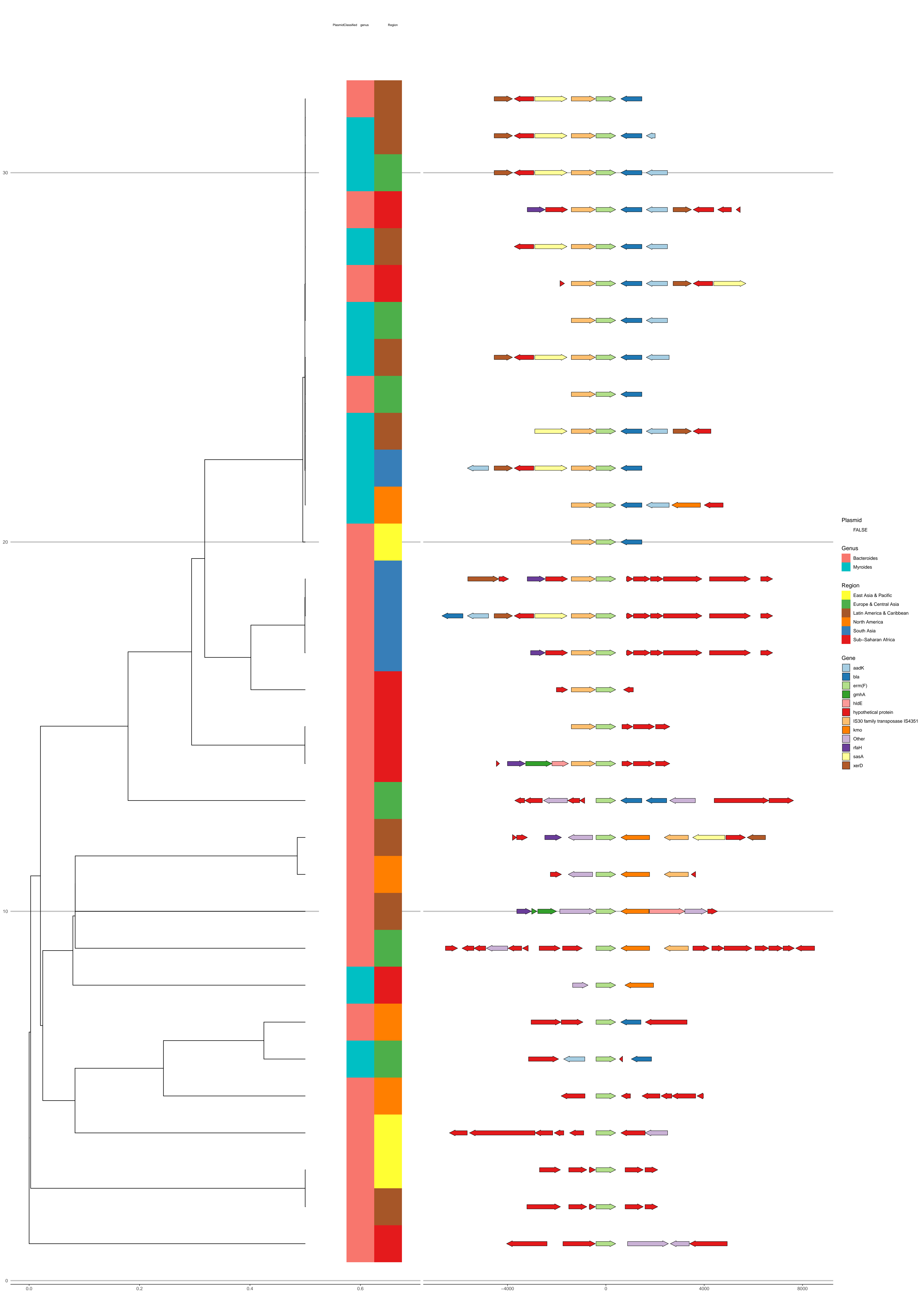

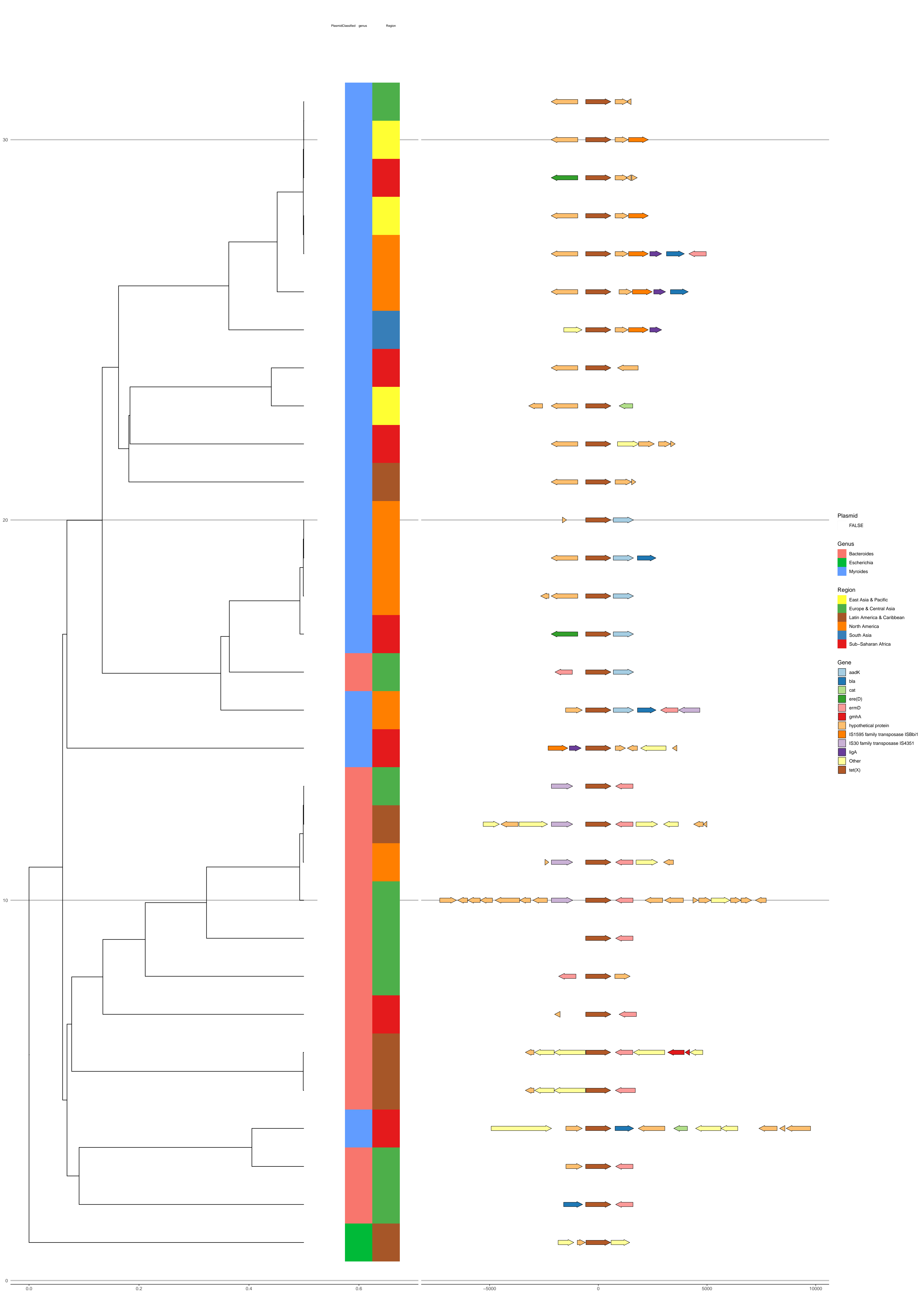

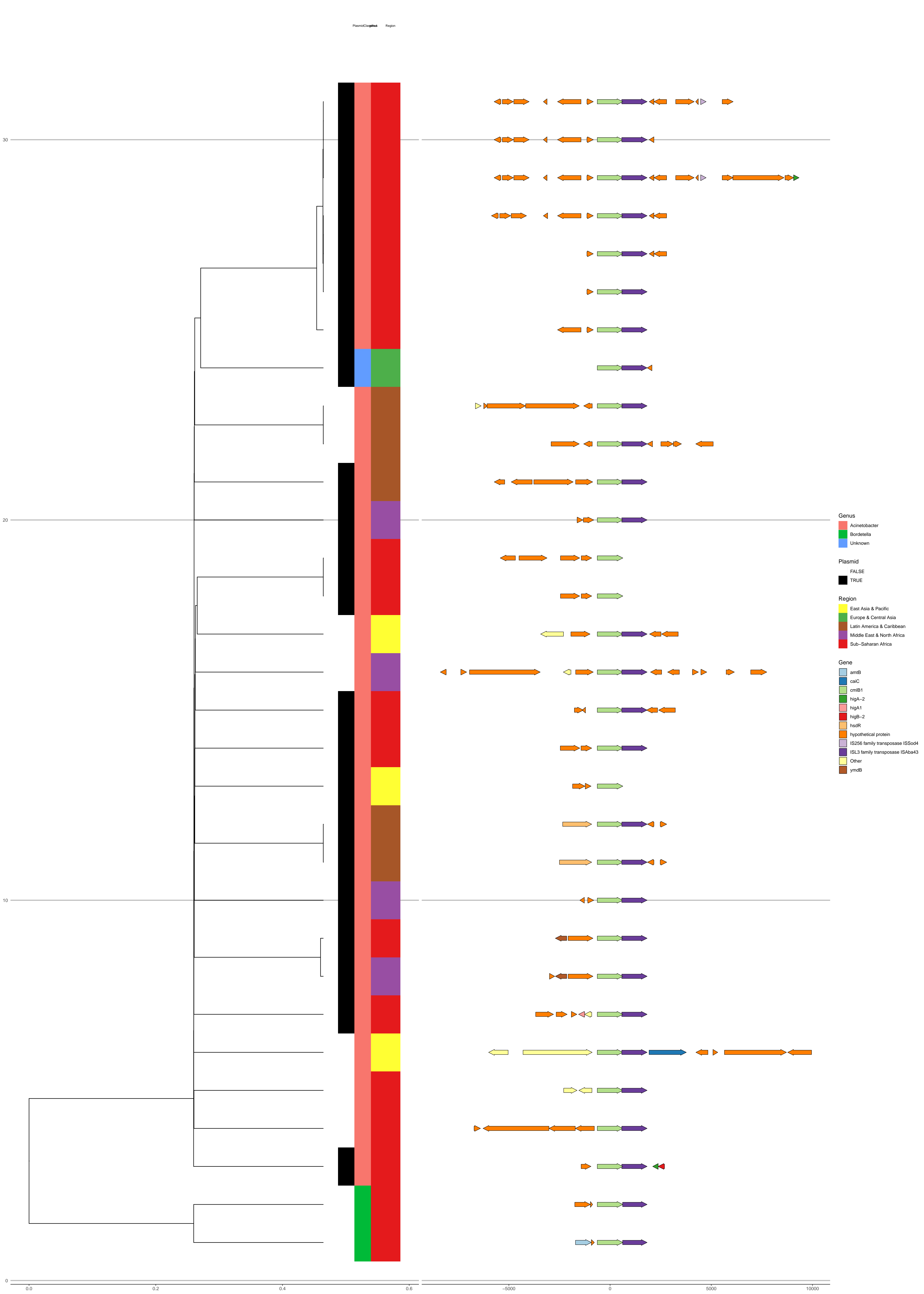

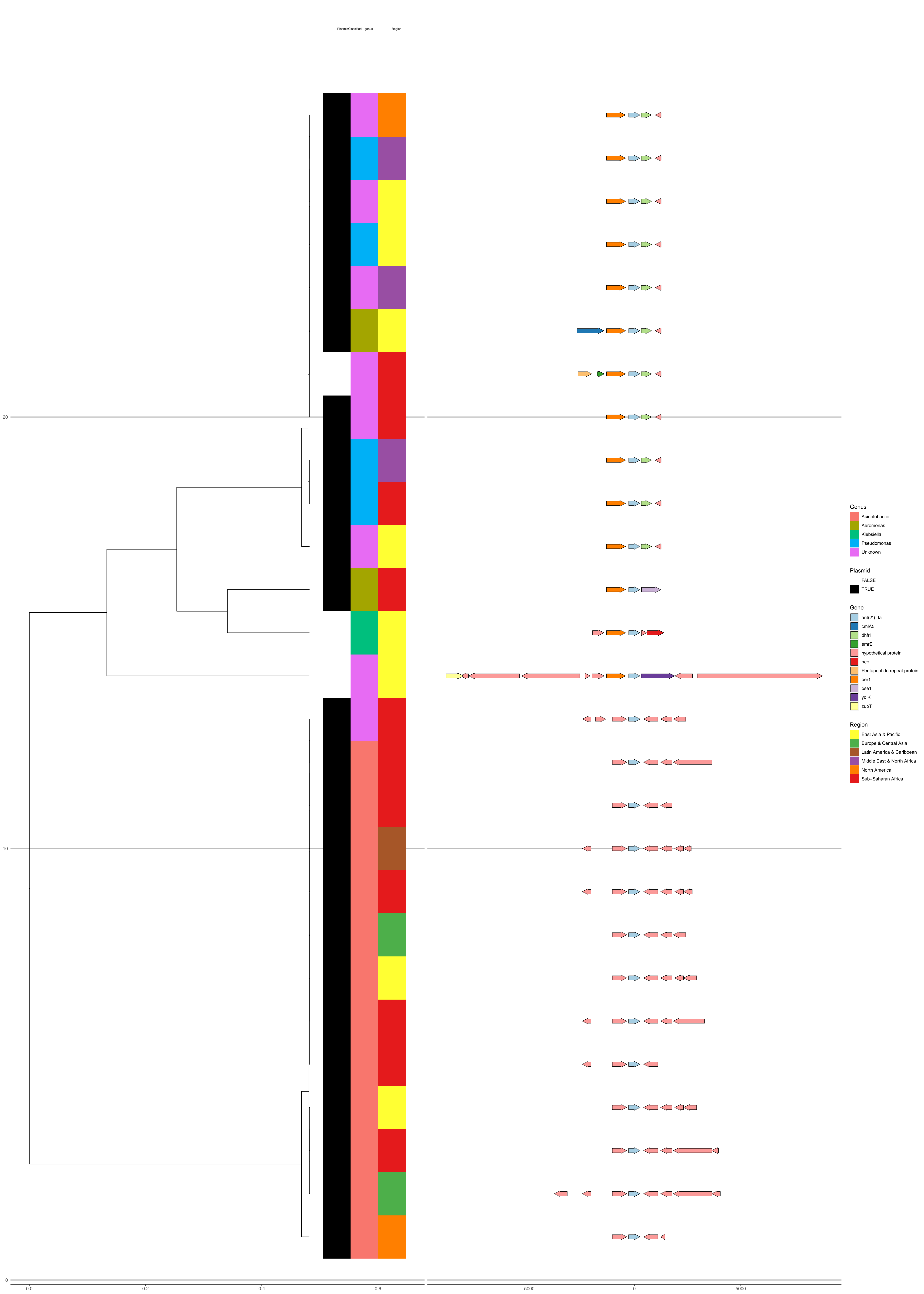

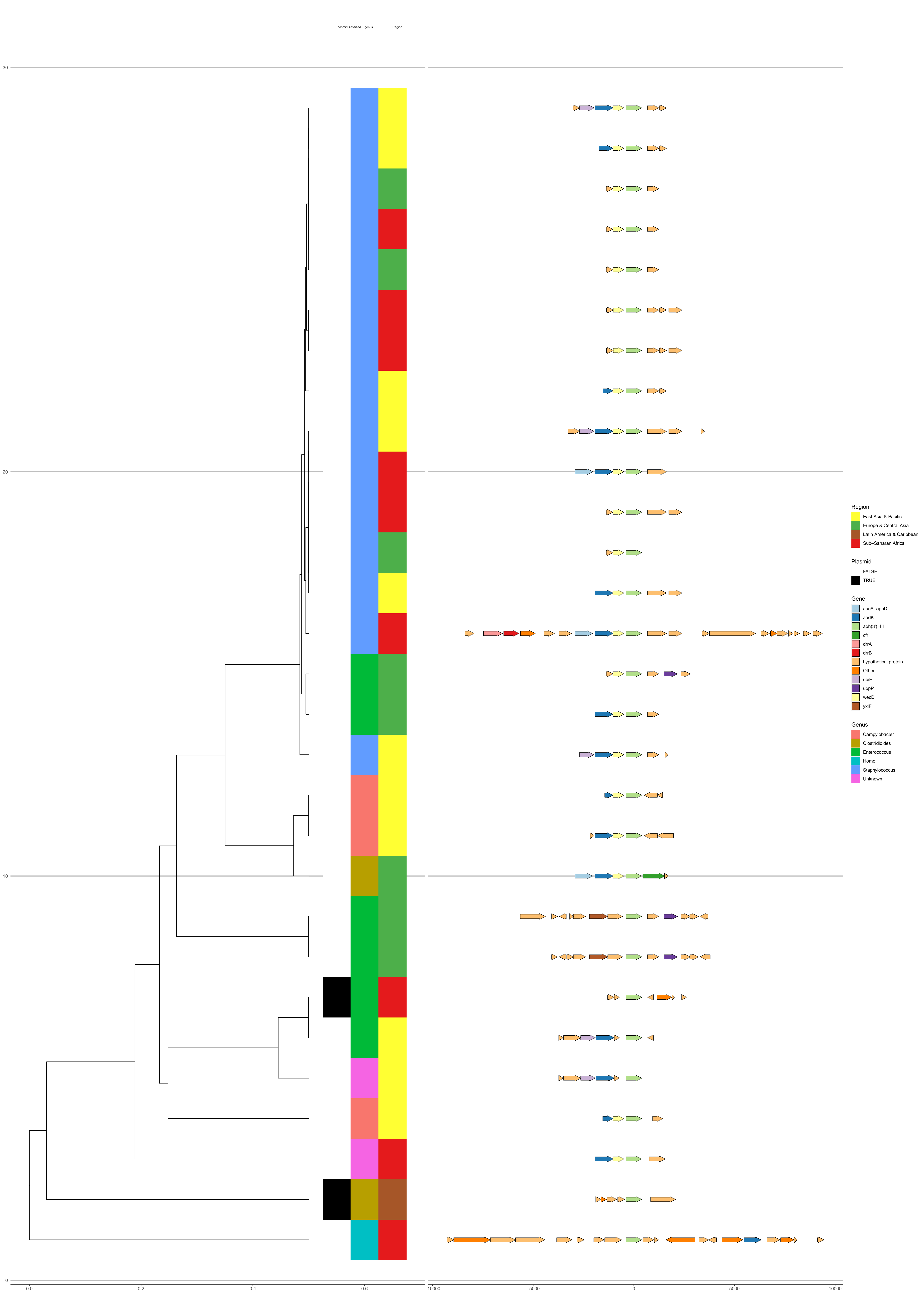

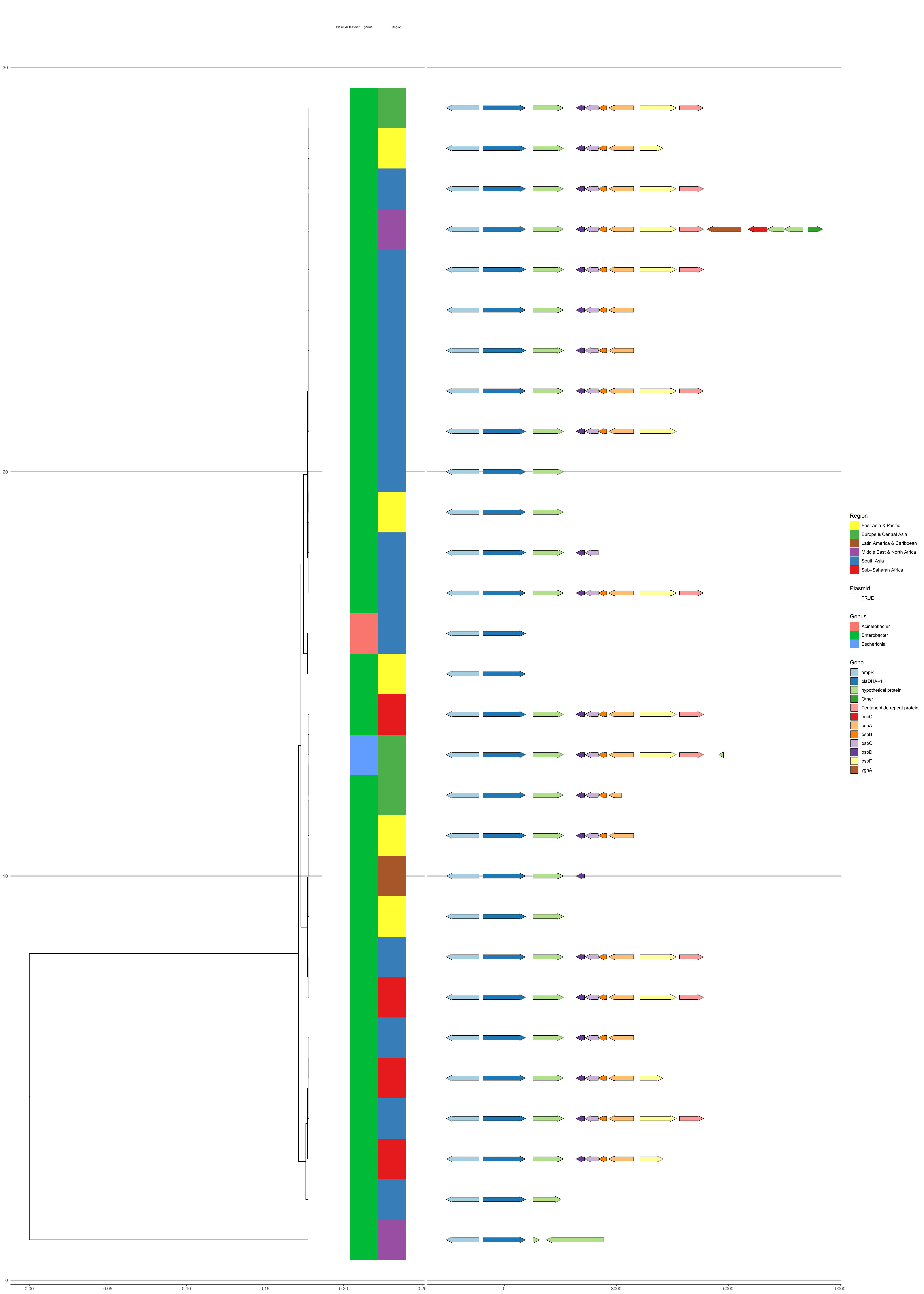

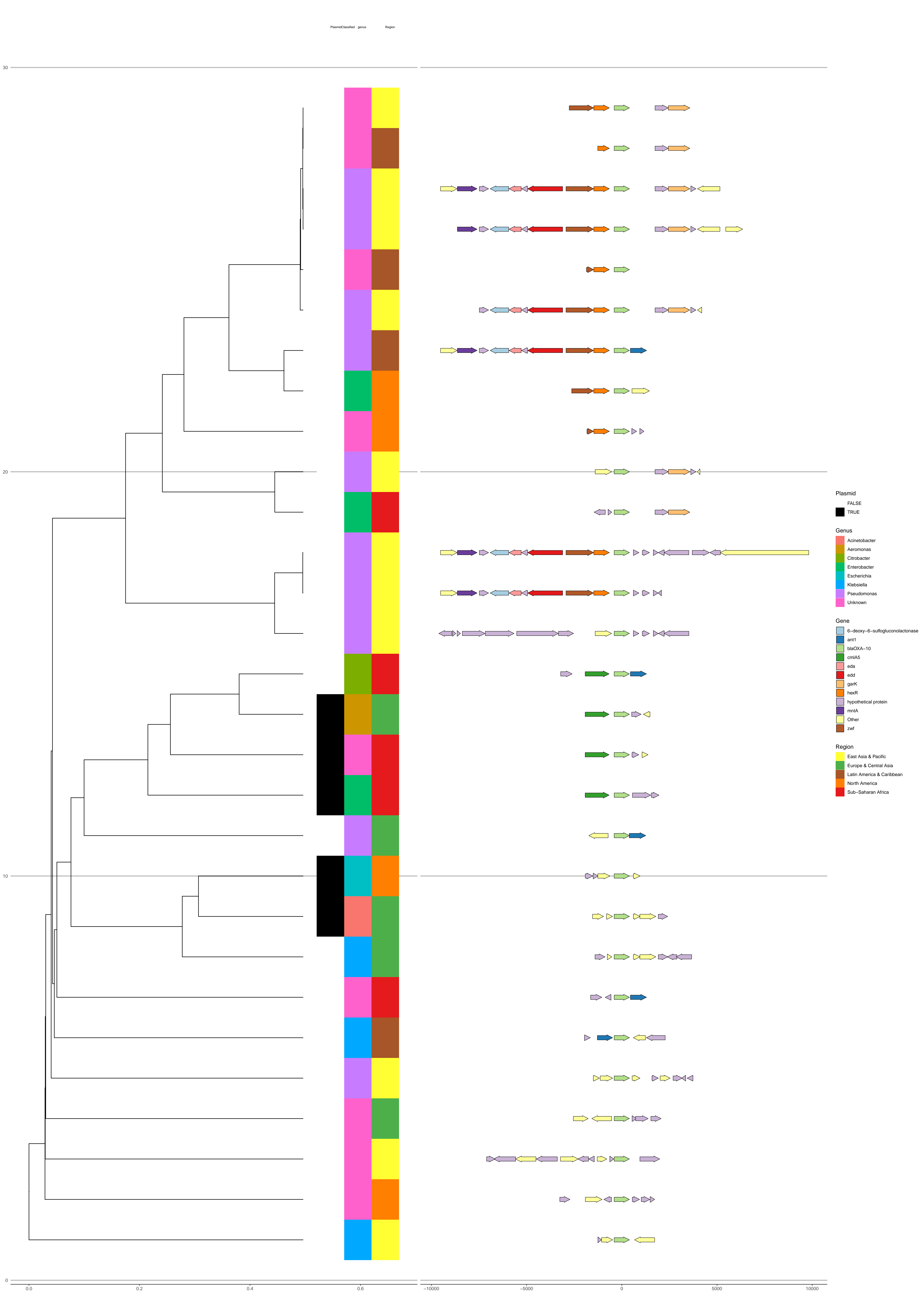

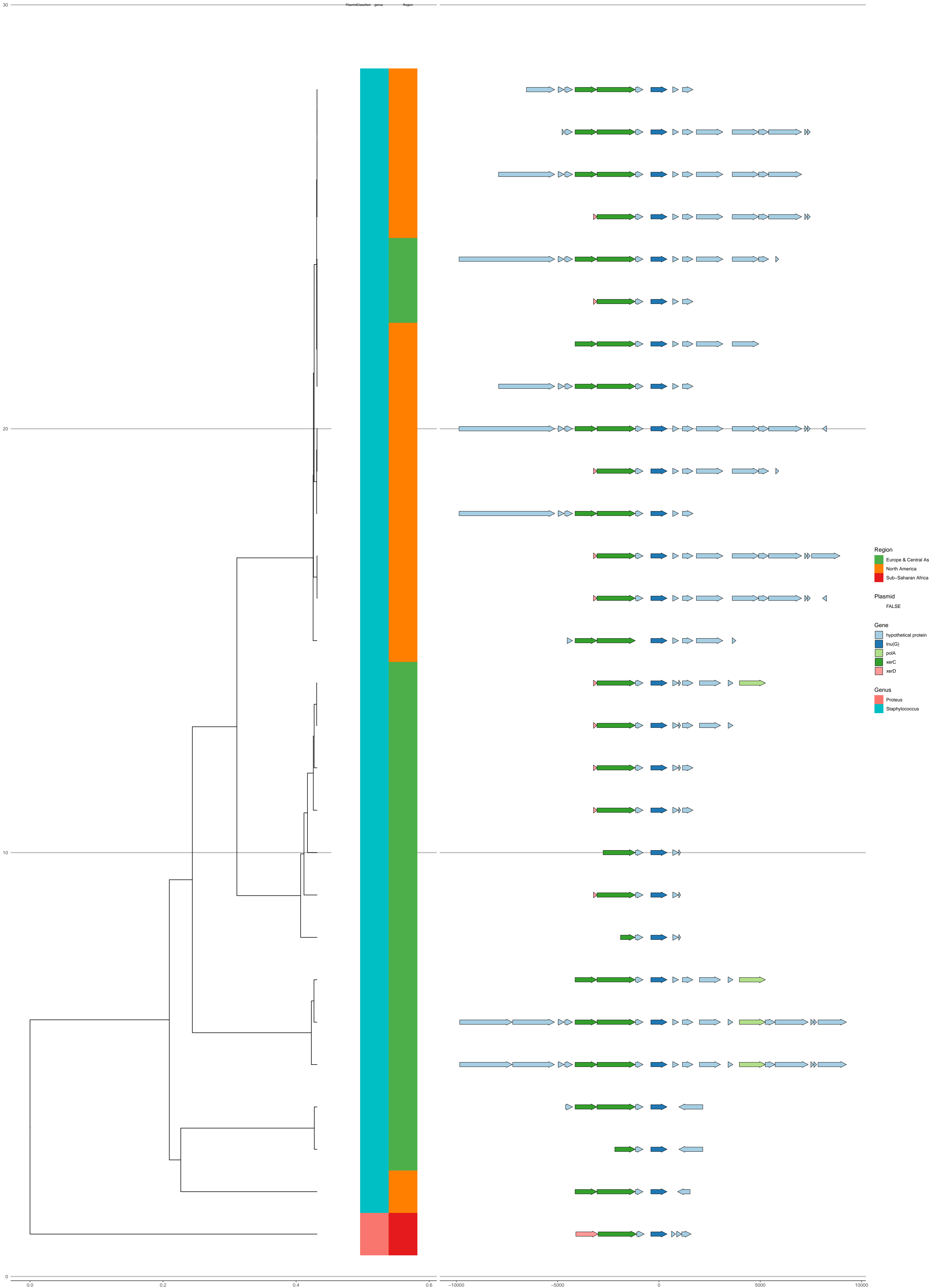

Supplement: Supplementary file 4 — Supplementary Dataset 3, Supplementary Dataset 4, Supplementary Dataset 5, Supplementary Dataset 6 [file 41467_2022_34312_MOESM4_ESM.zip › Supplementary Data 3 - ARG Flank Cluster Synteny.pdf]

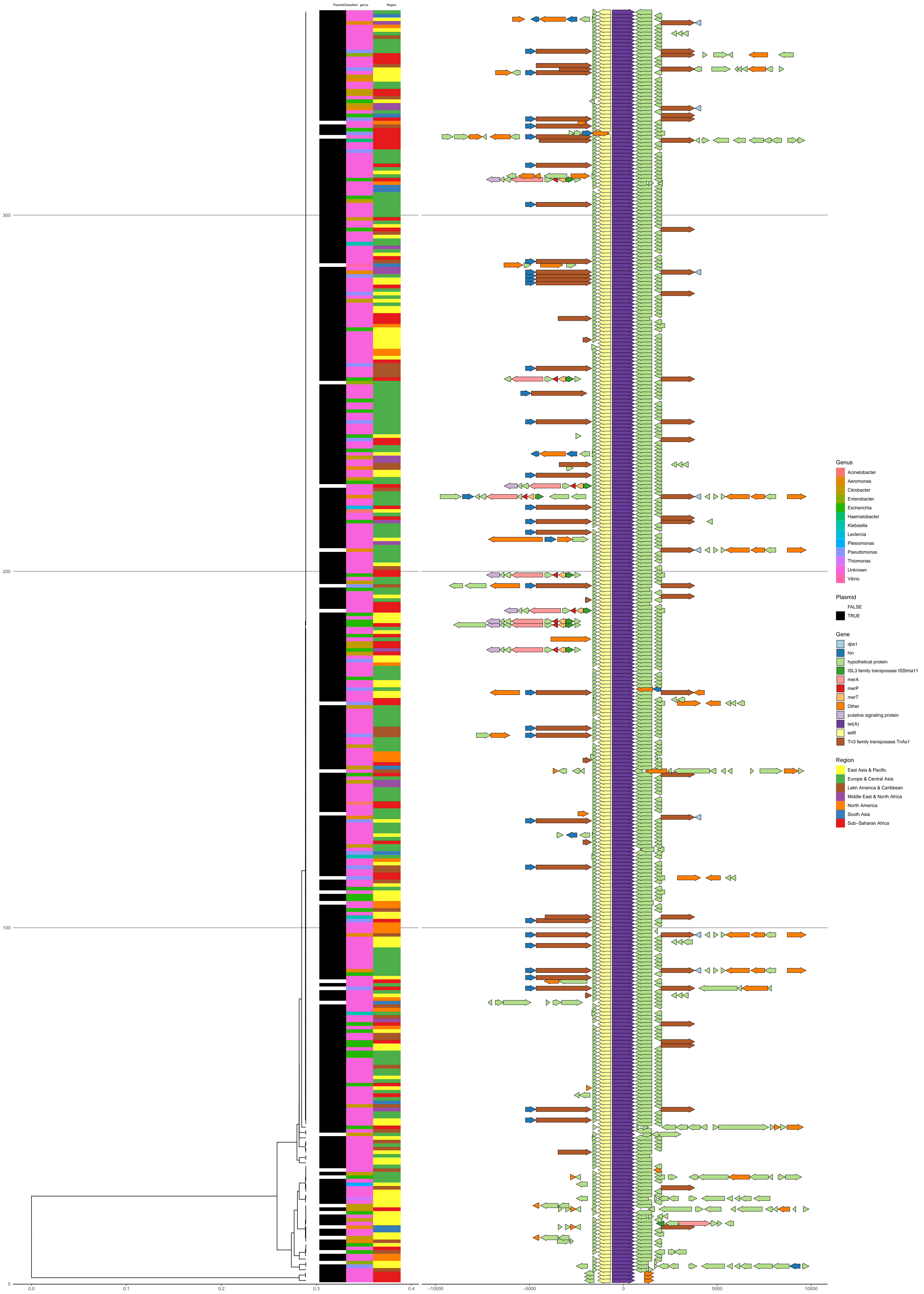

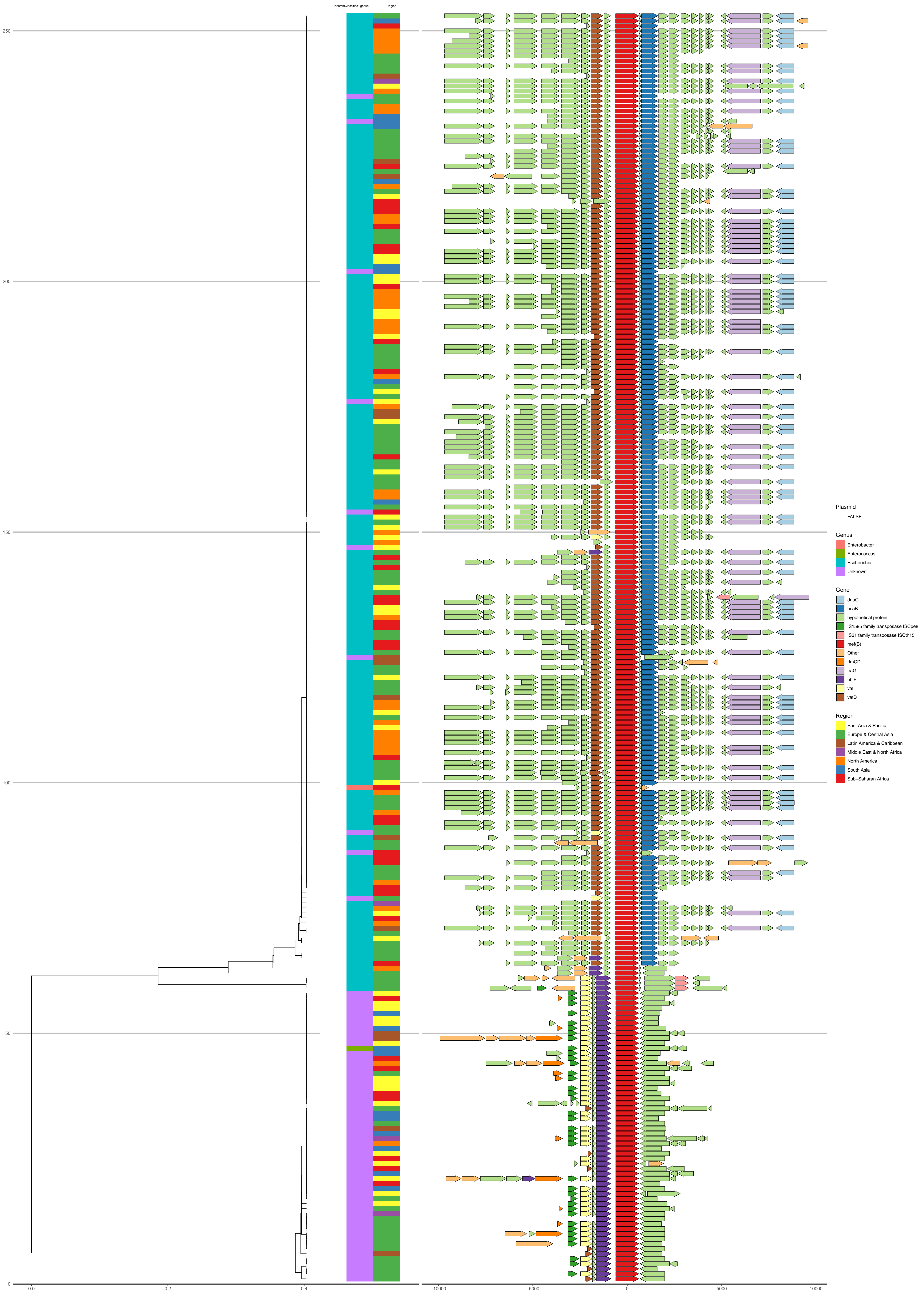

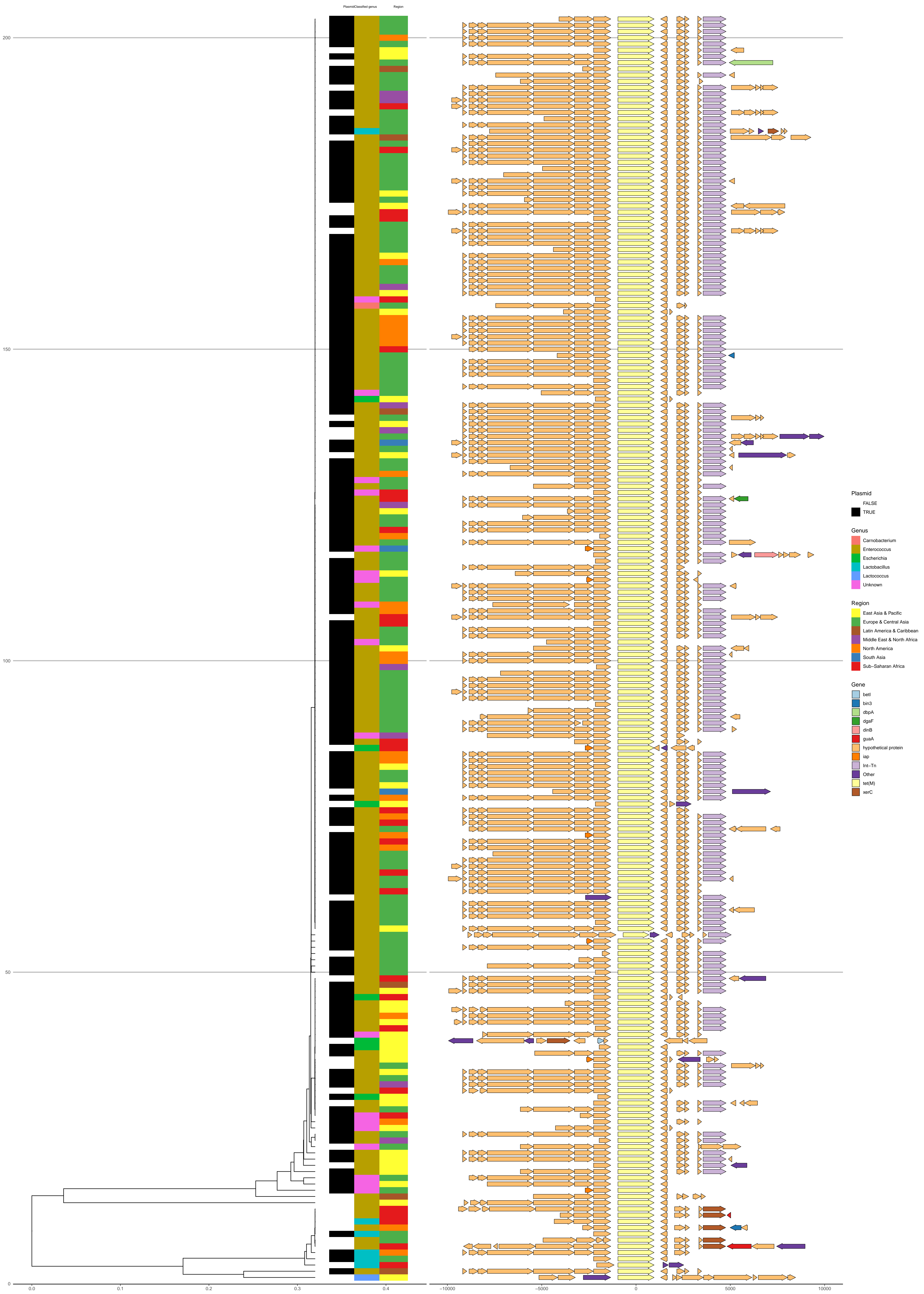

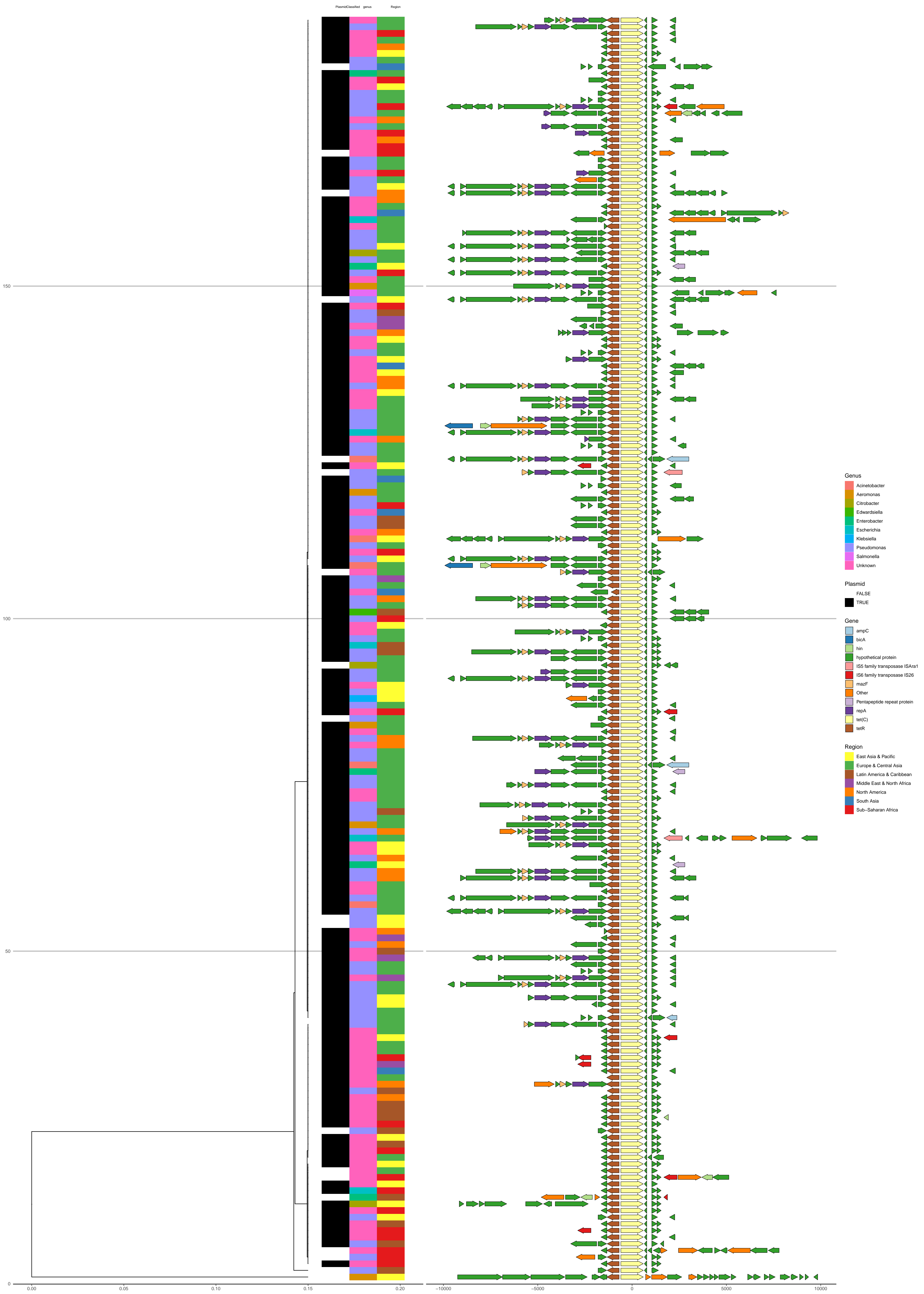

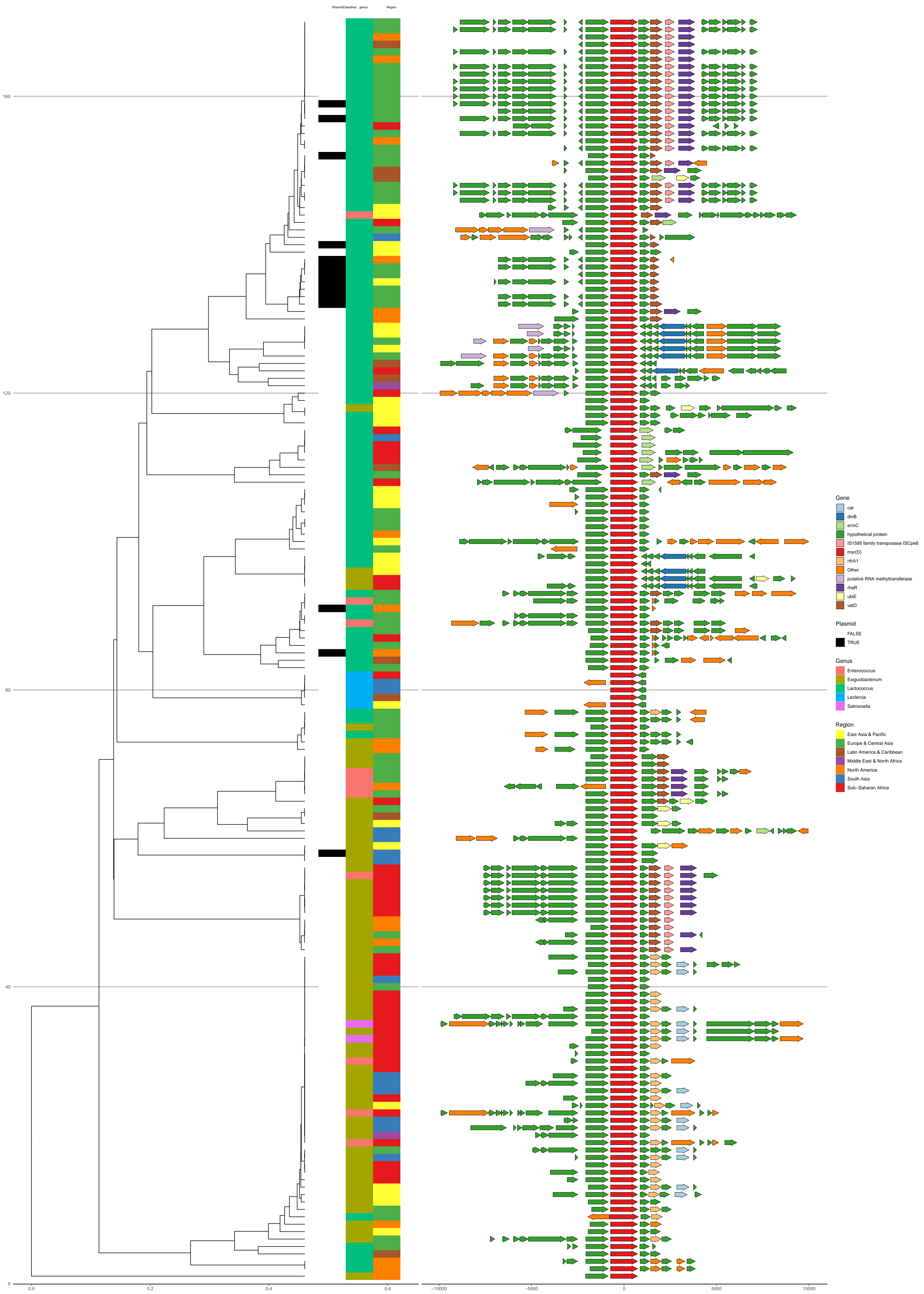

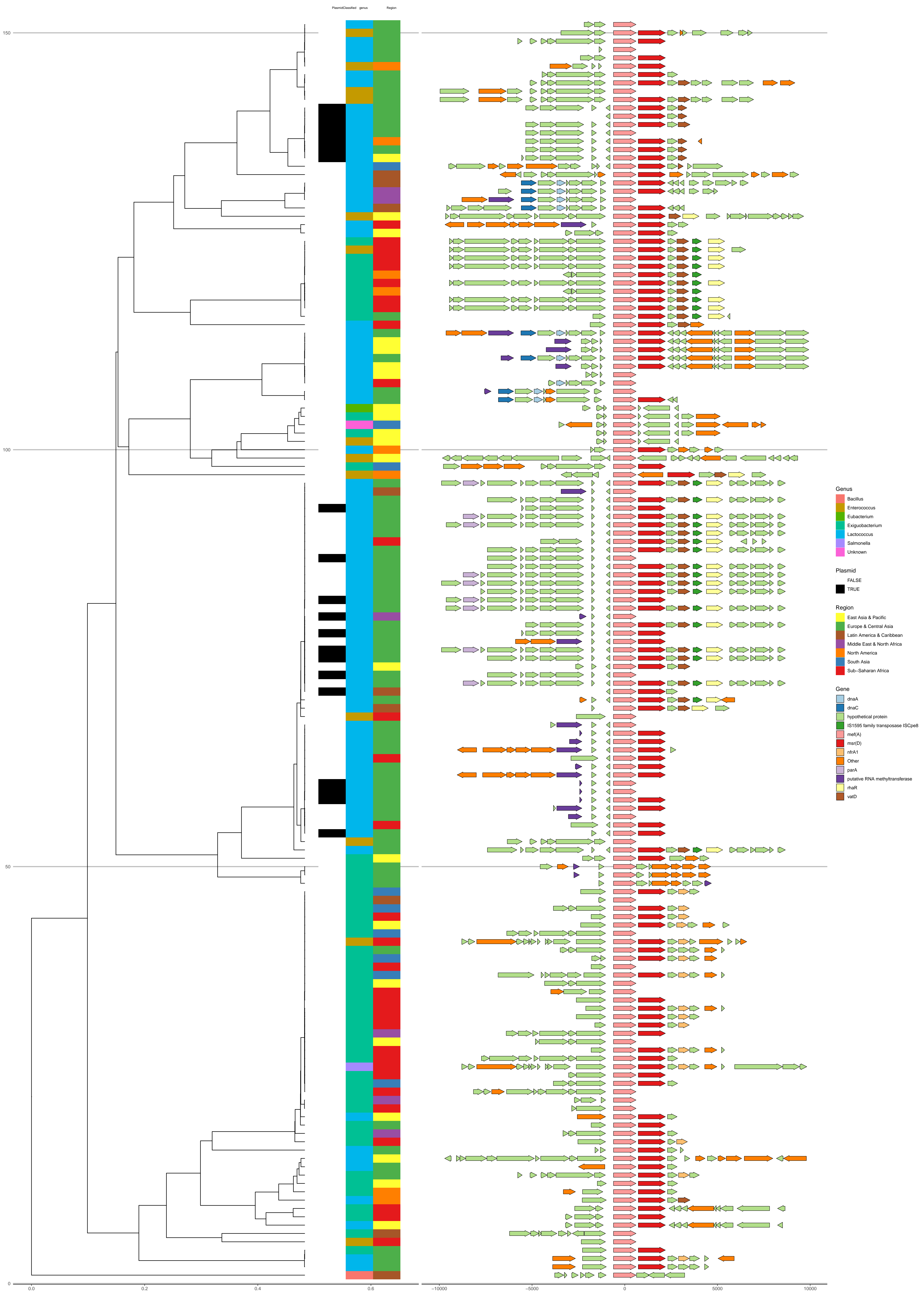

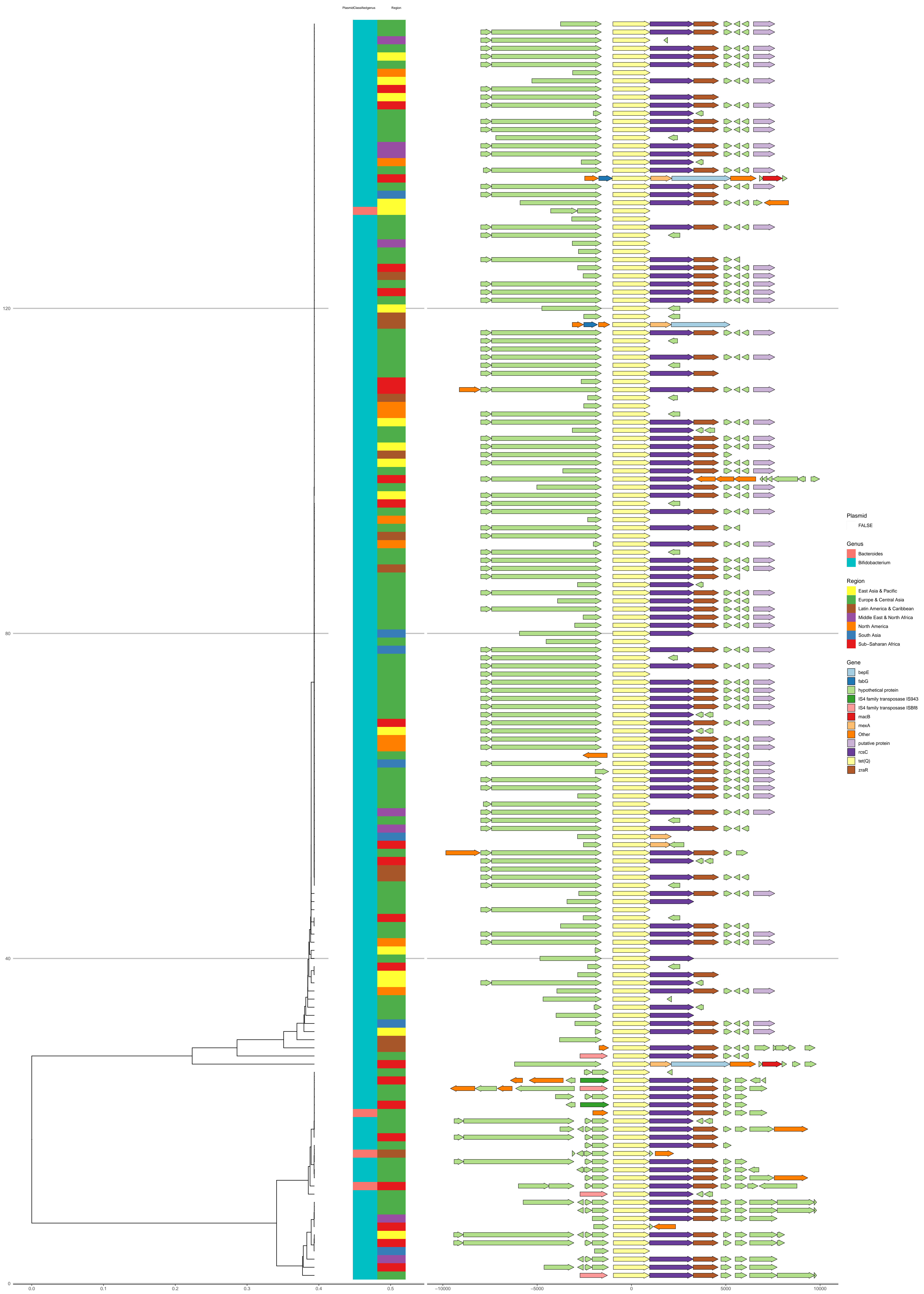

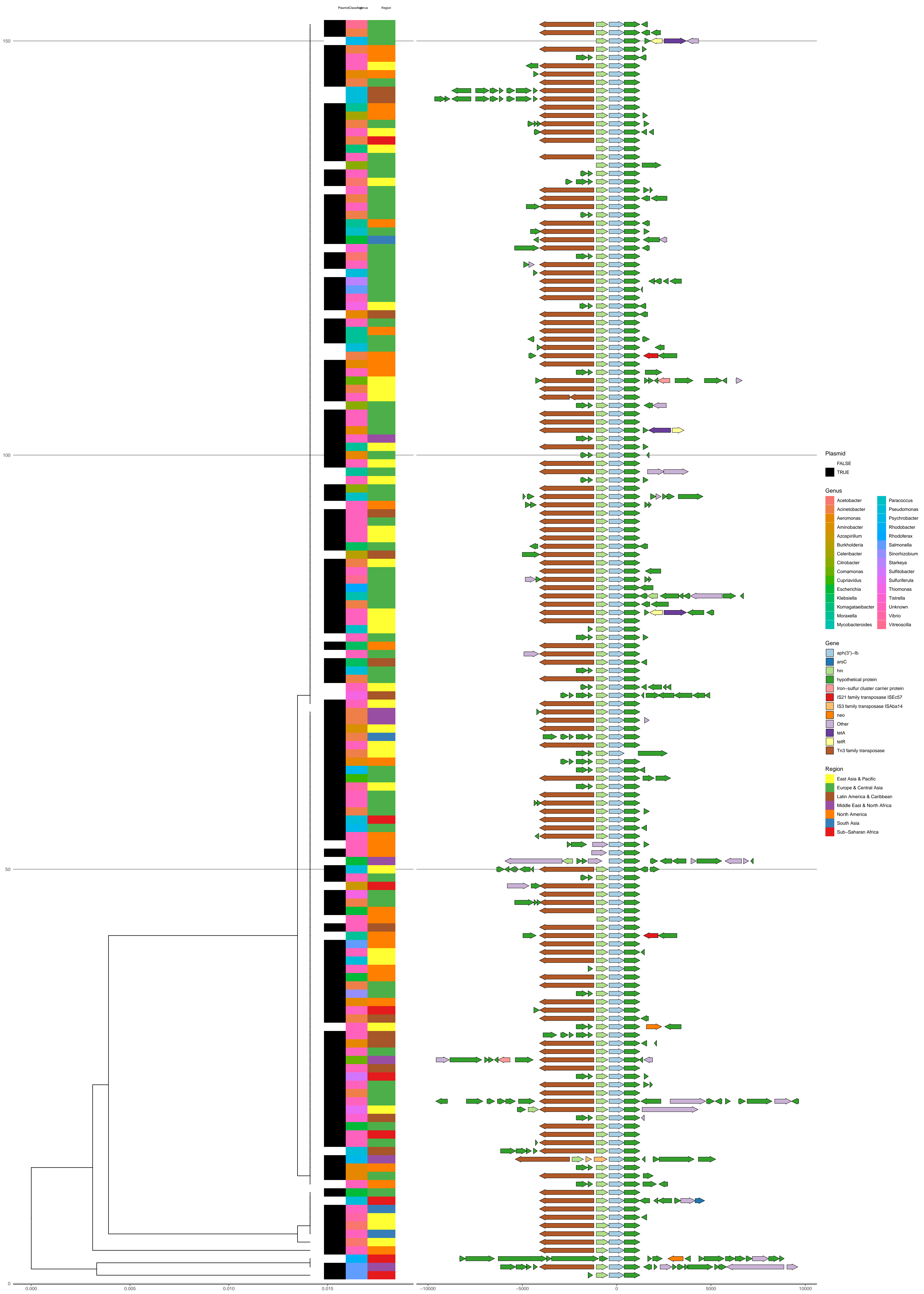

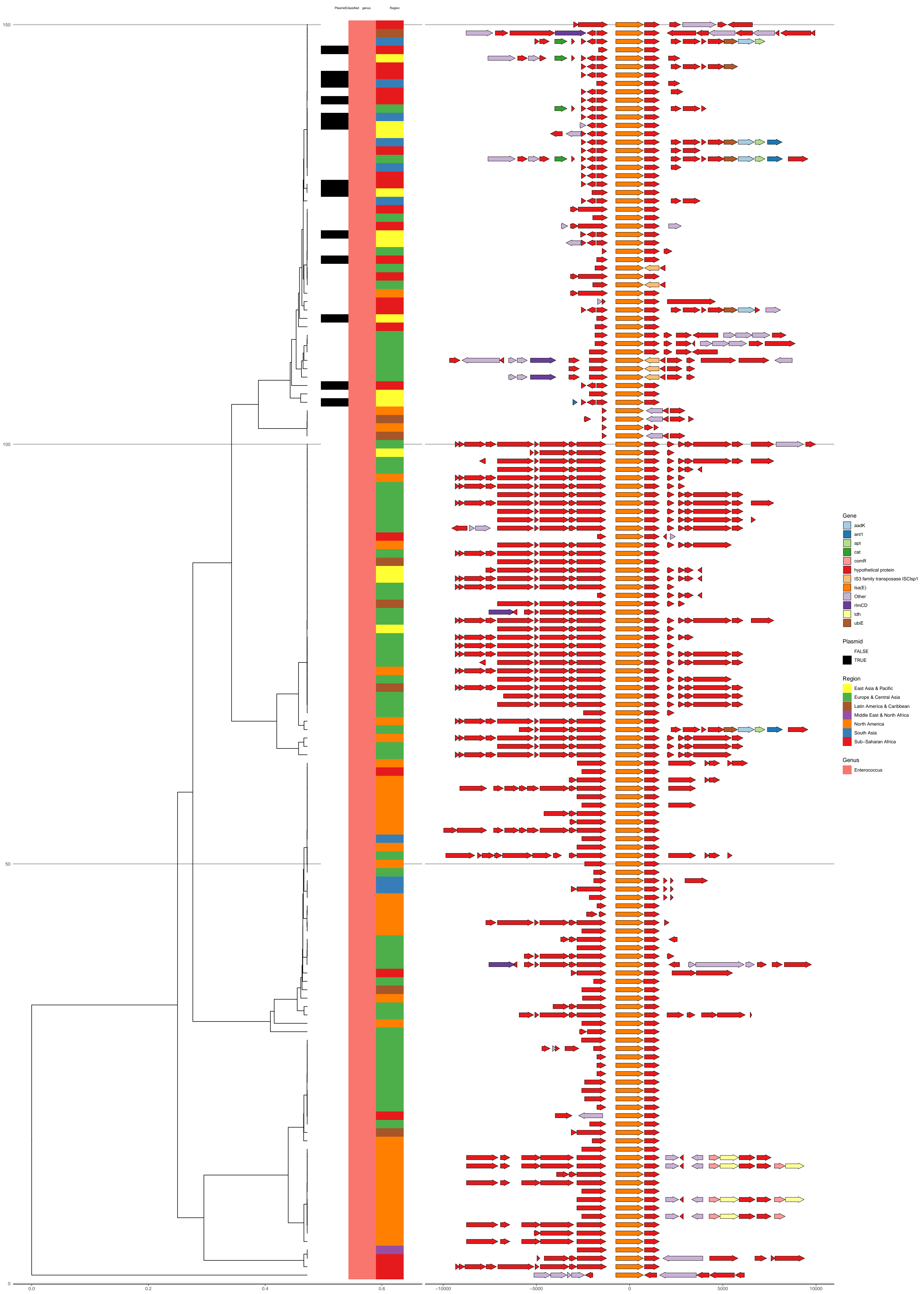

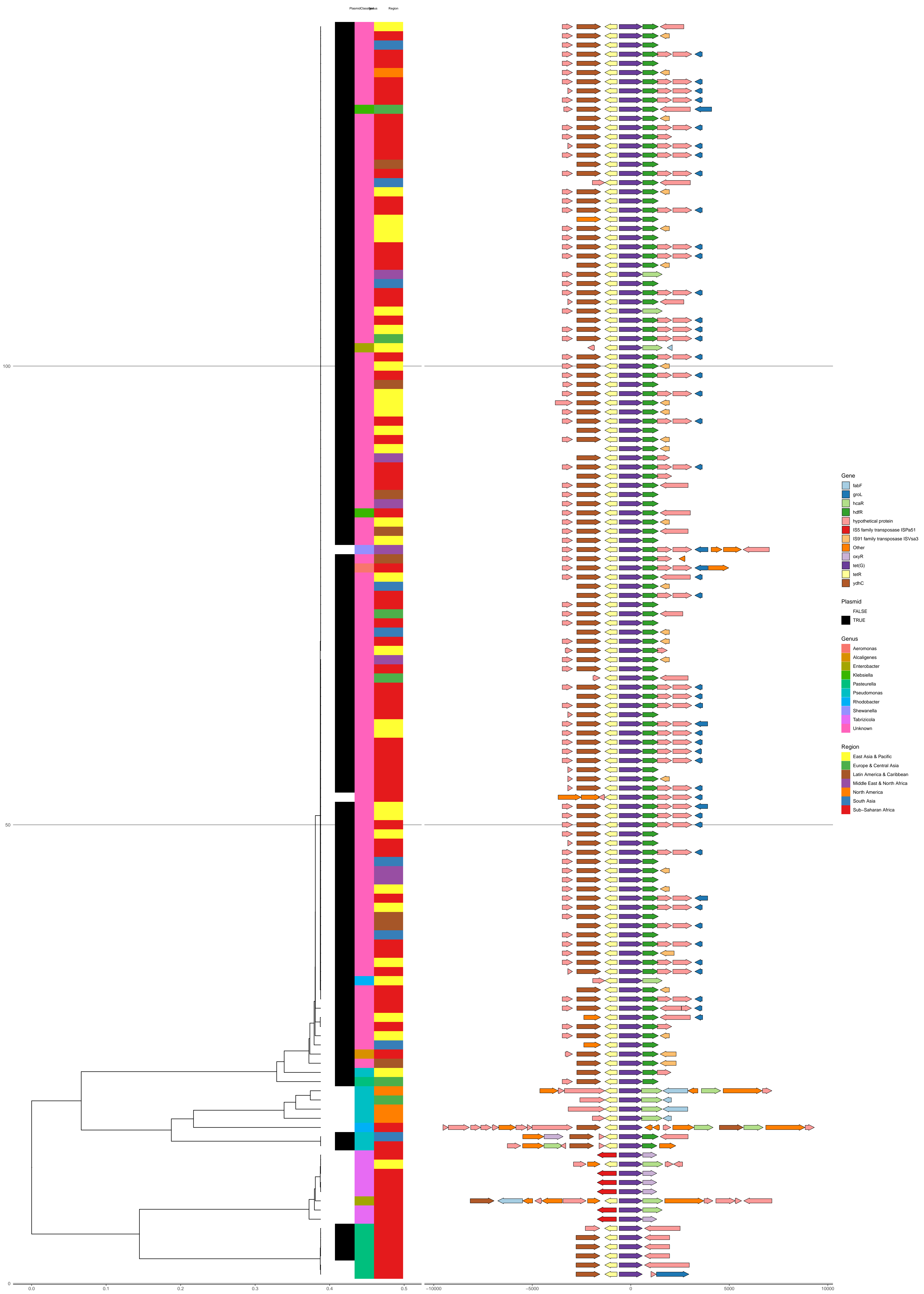

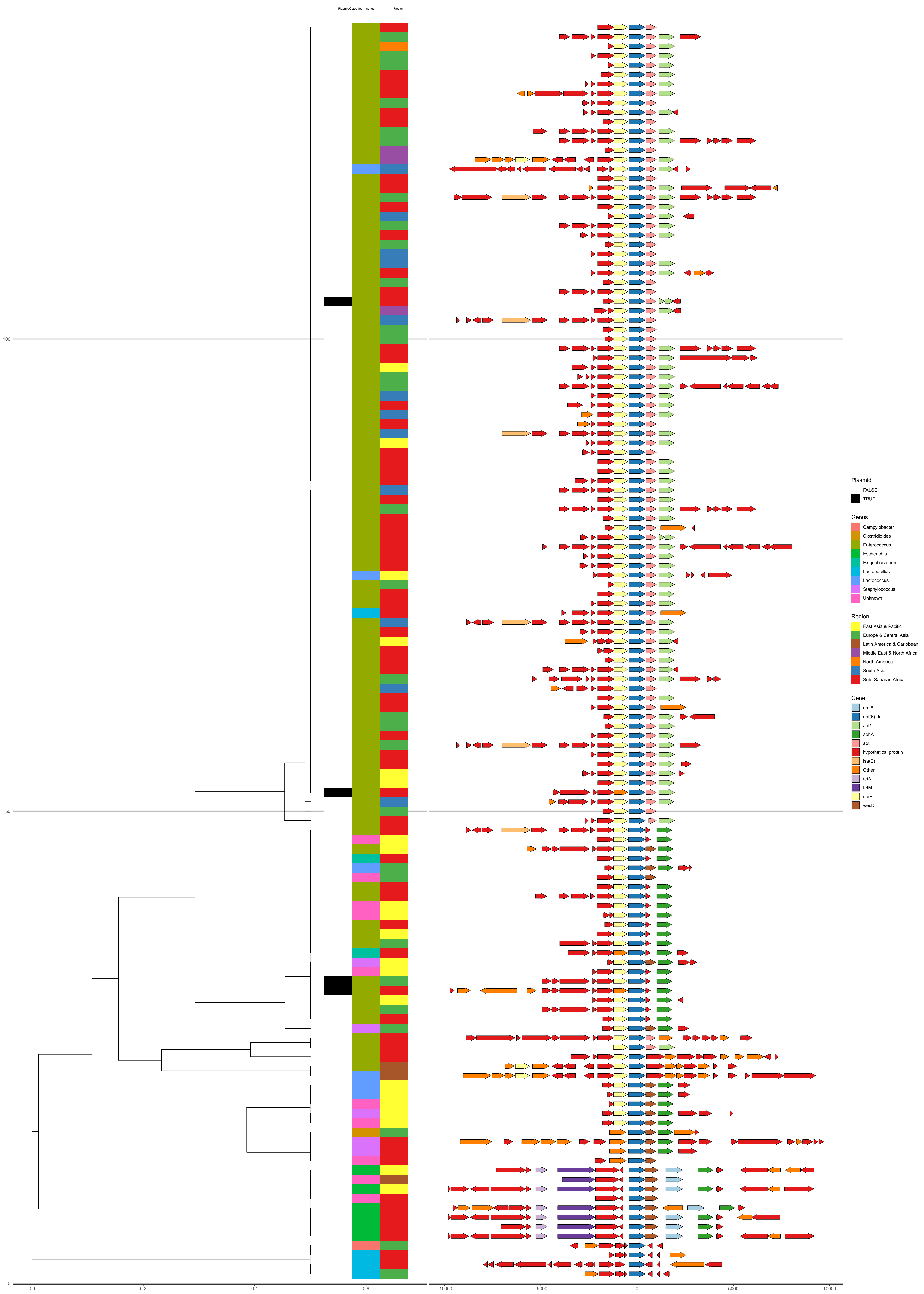

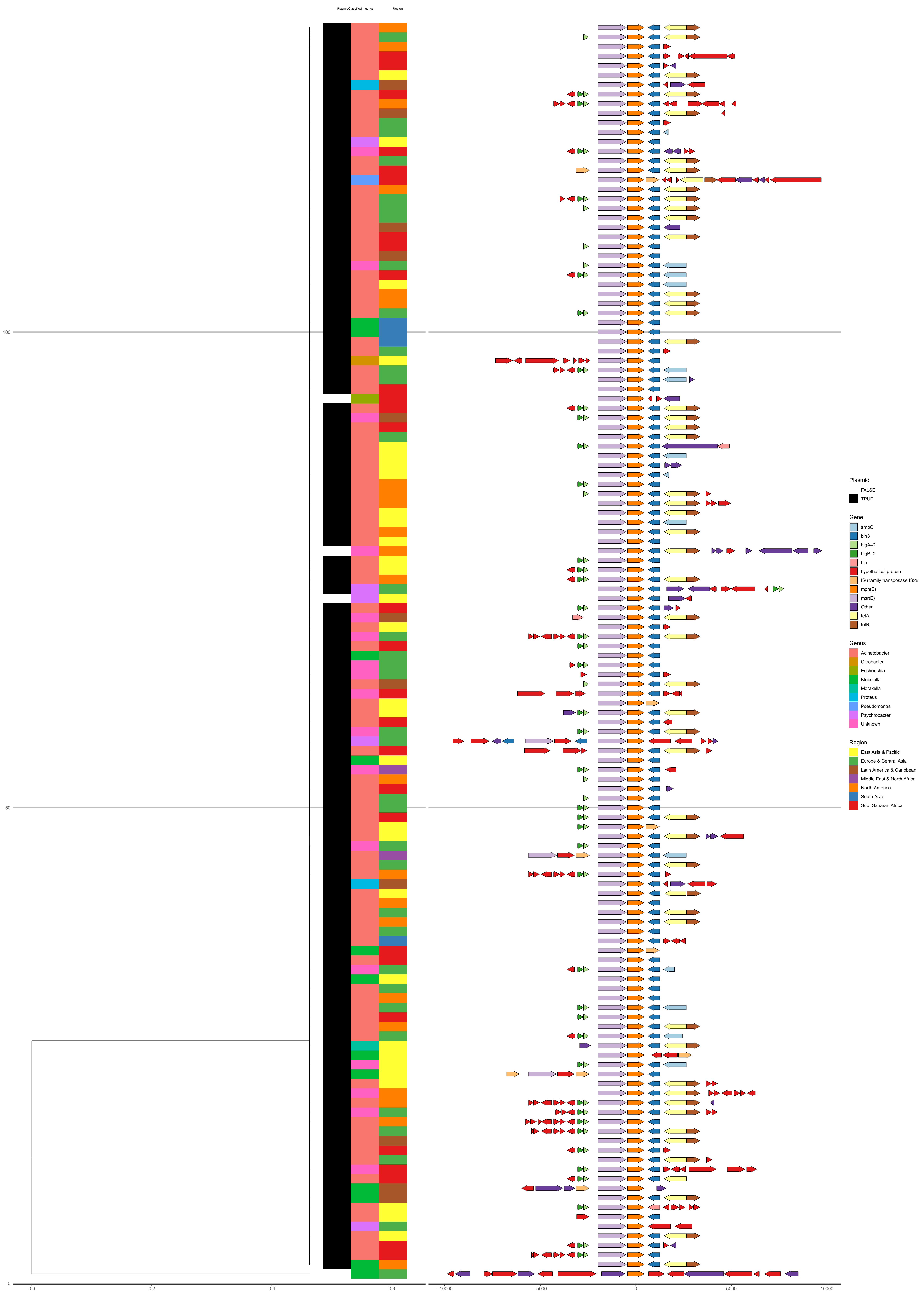

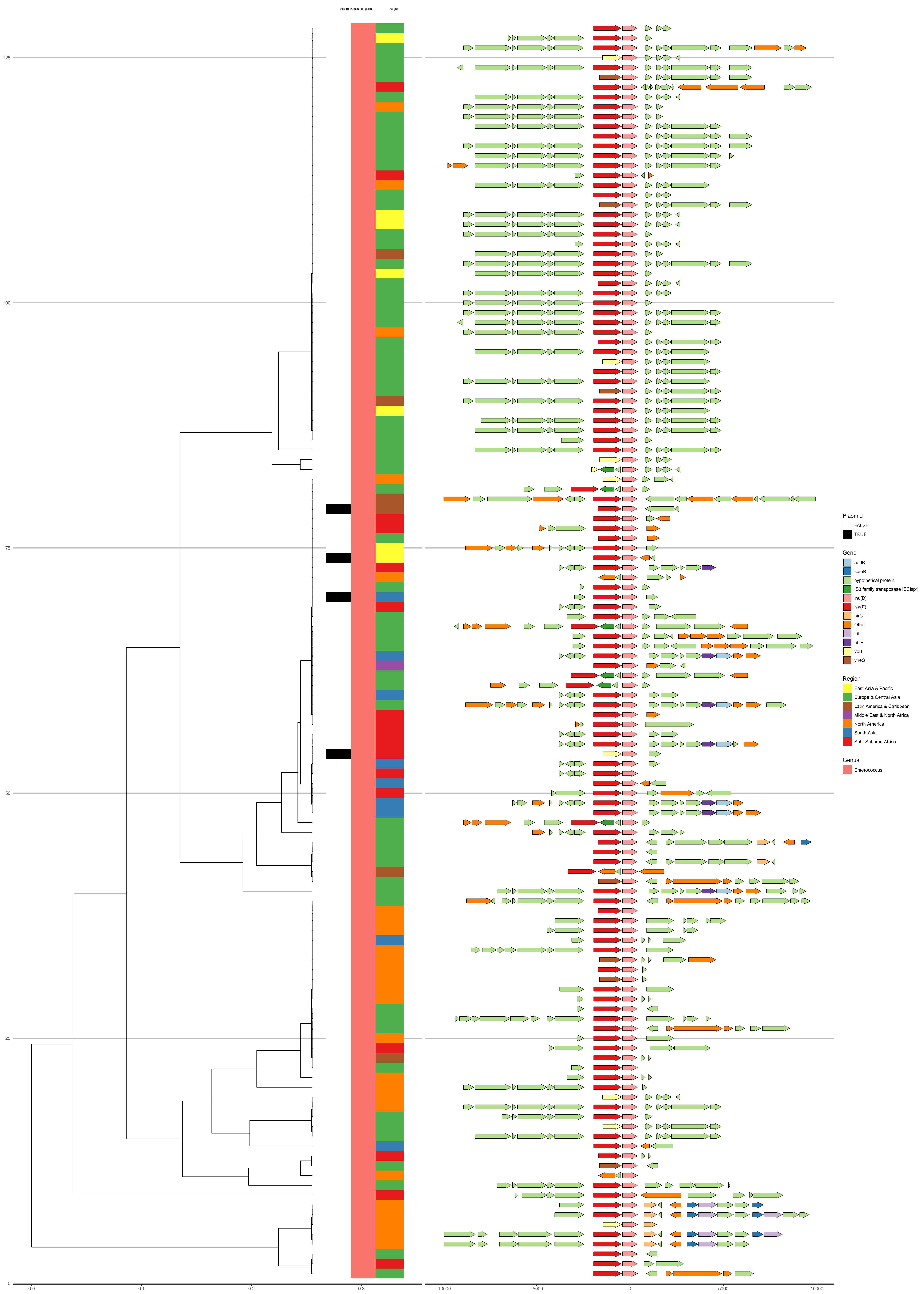

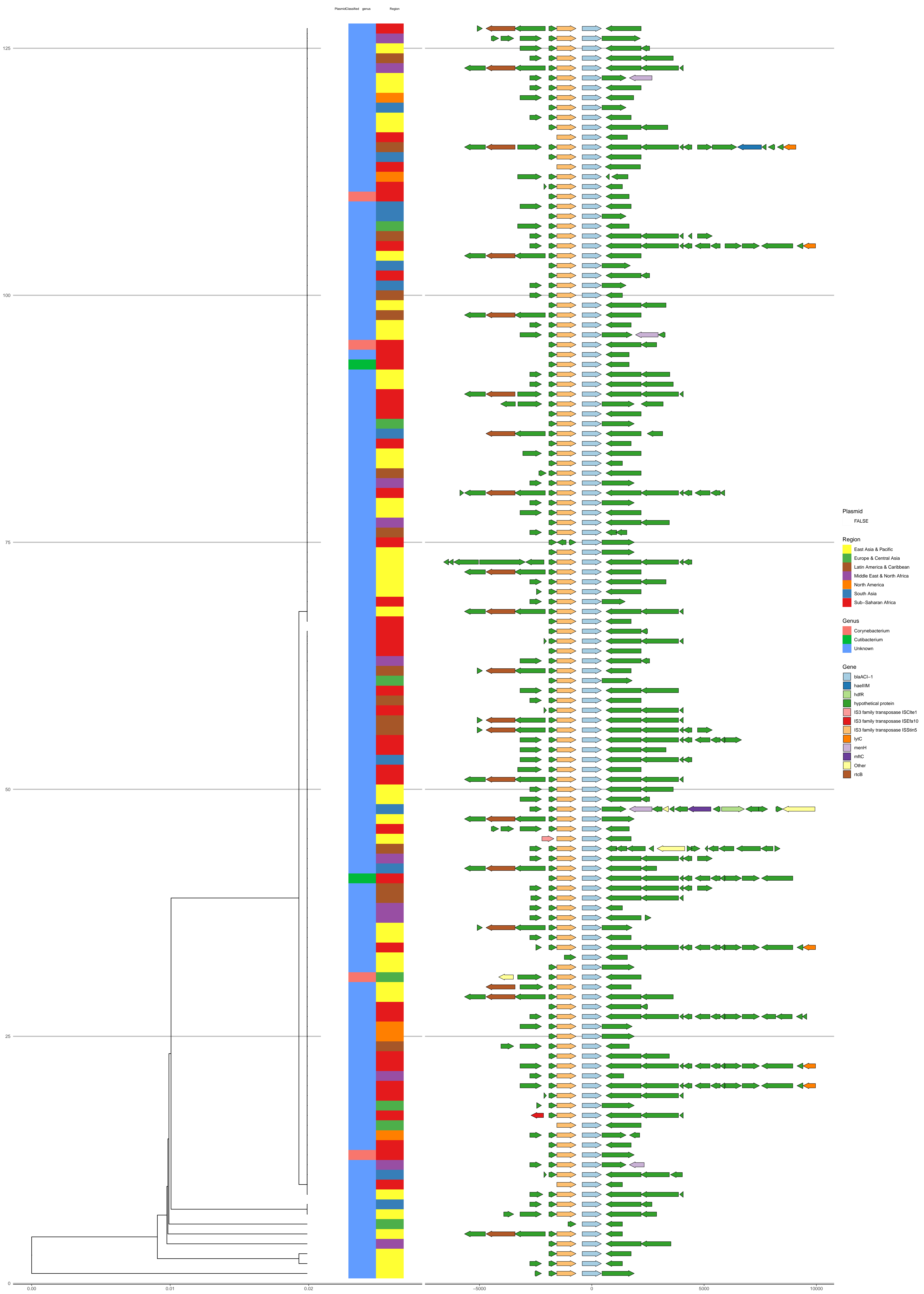

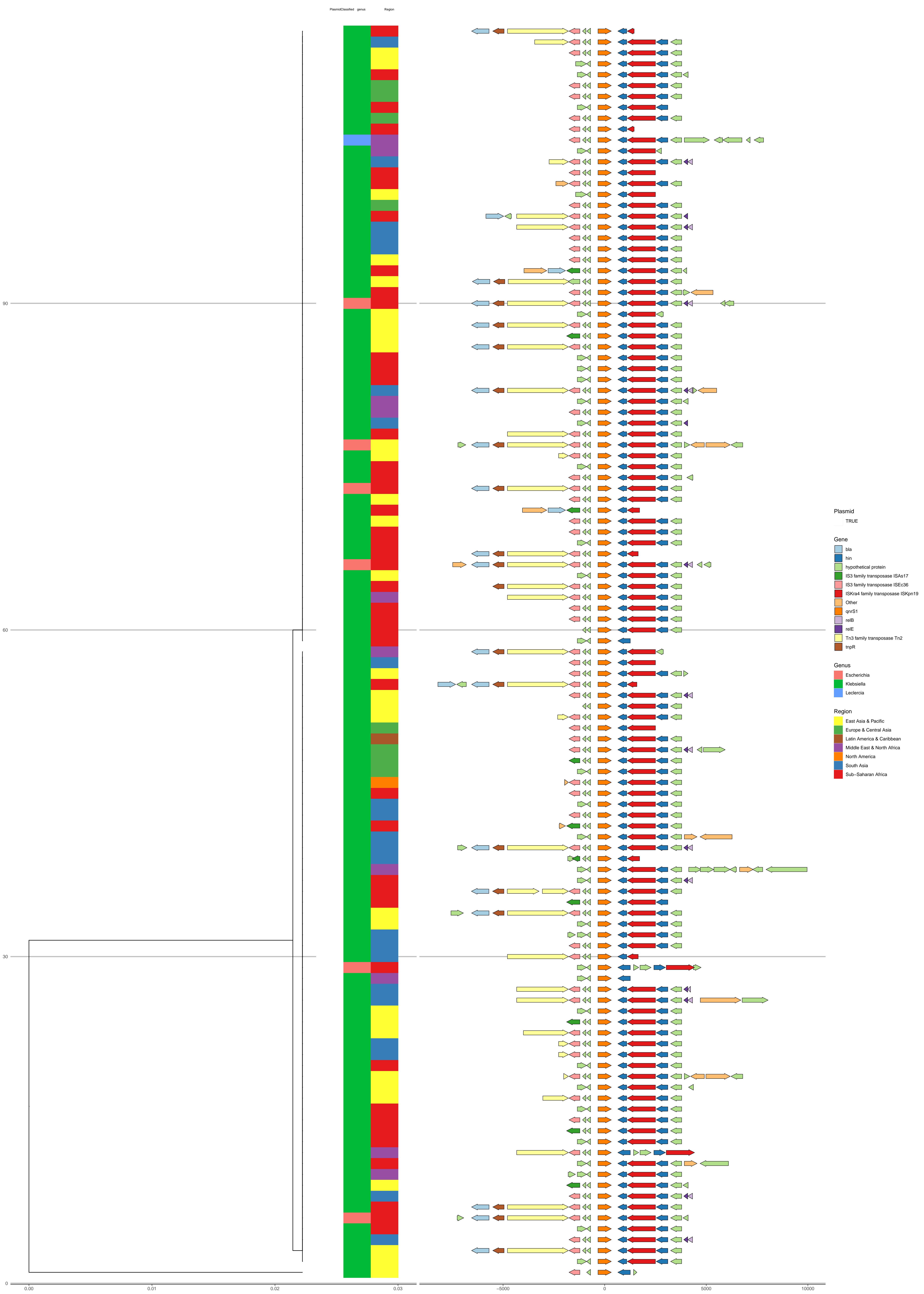

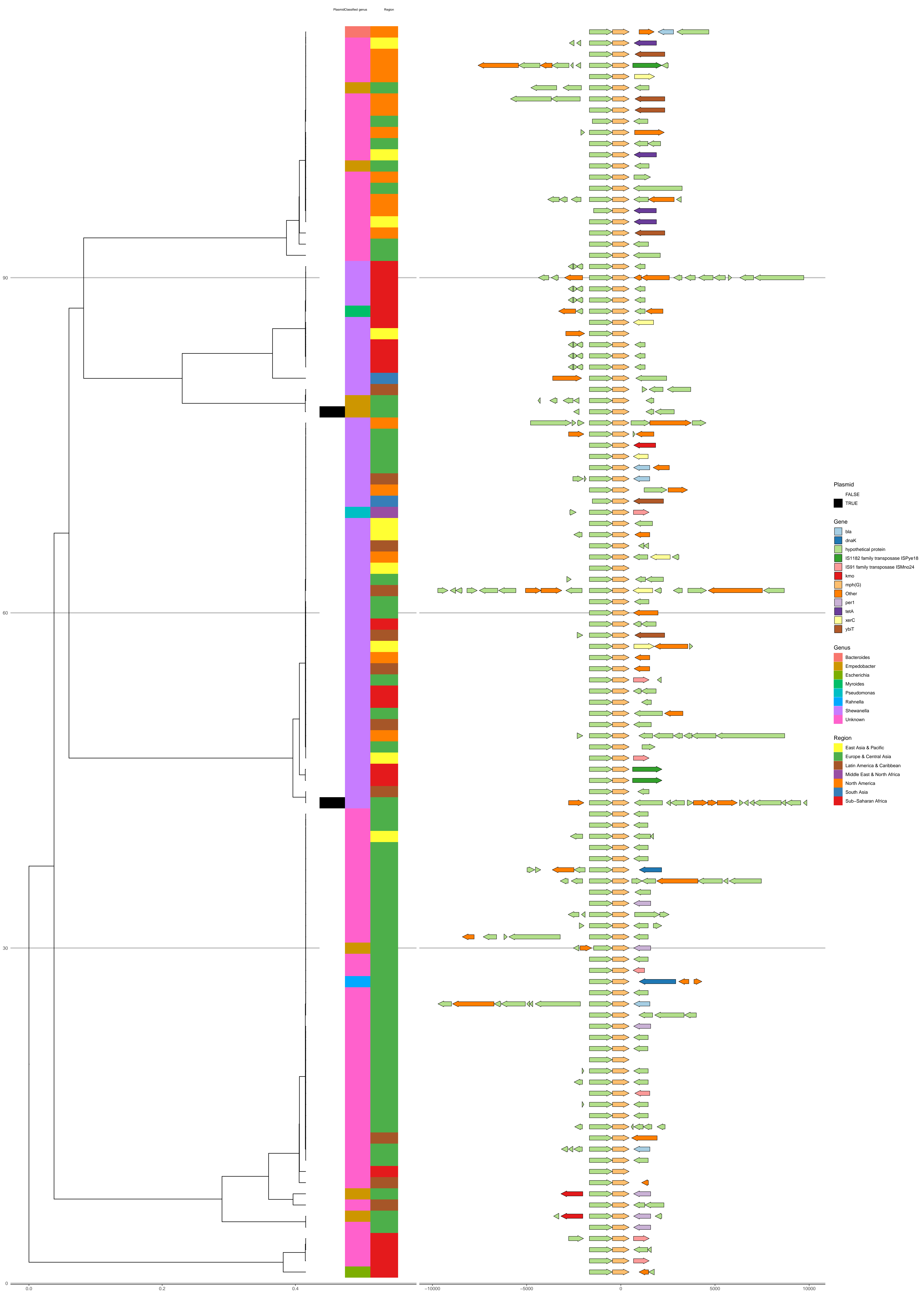

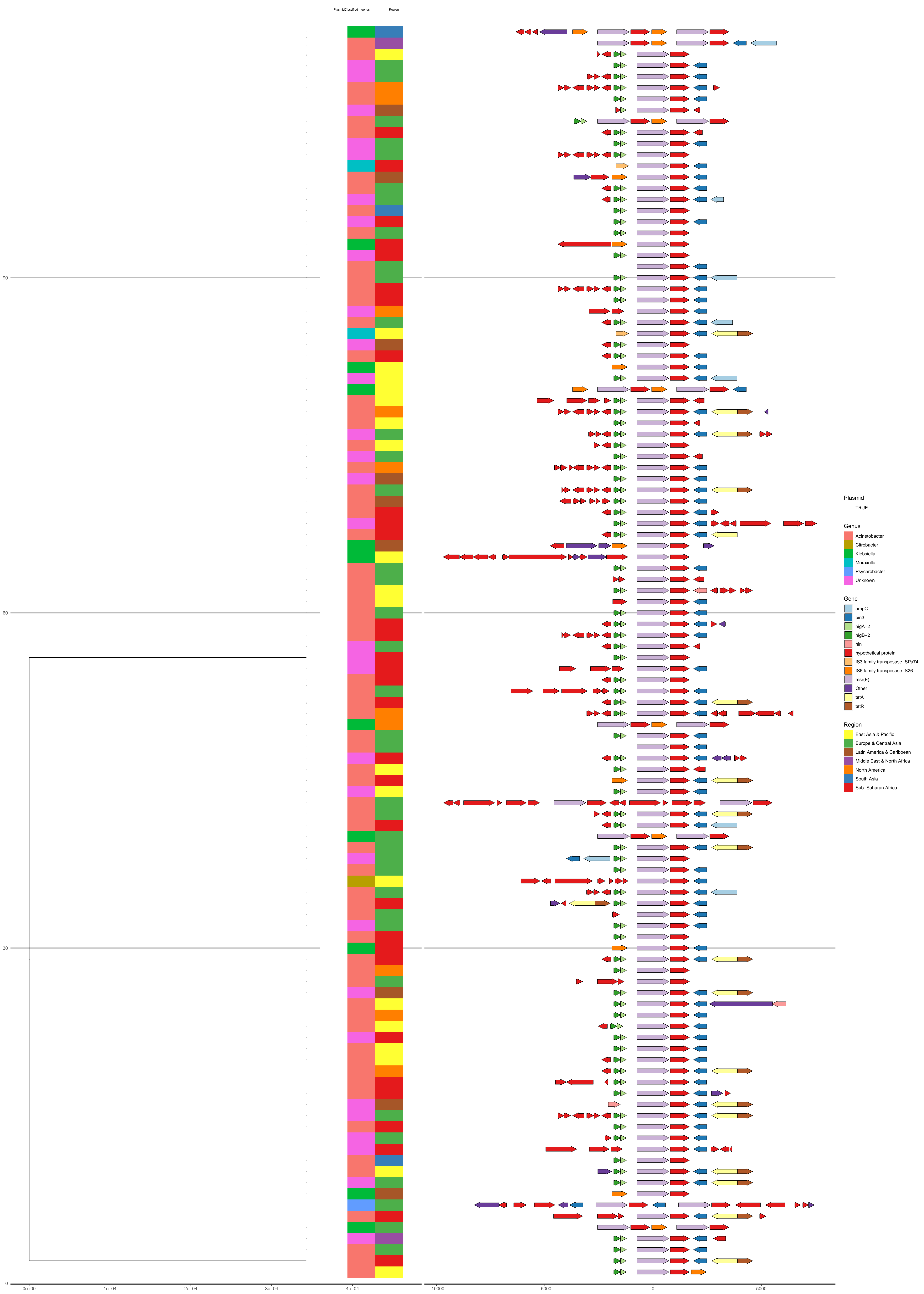

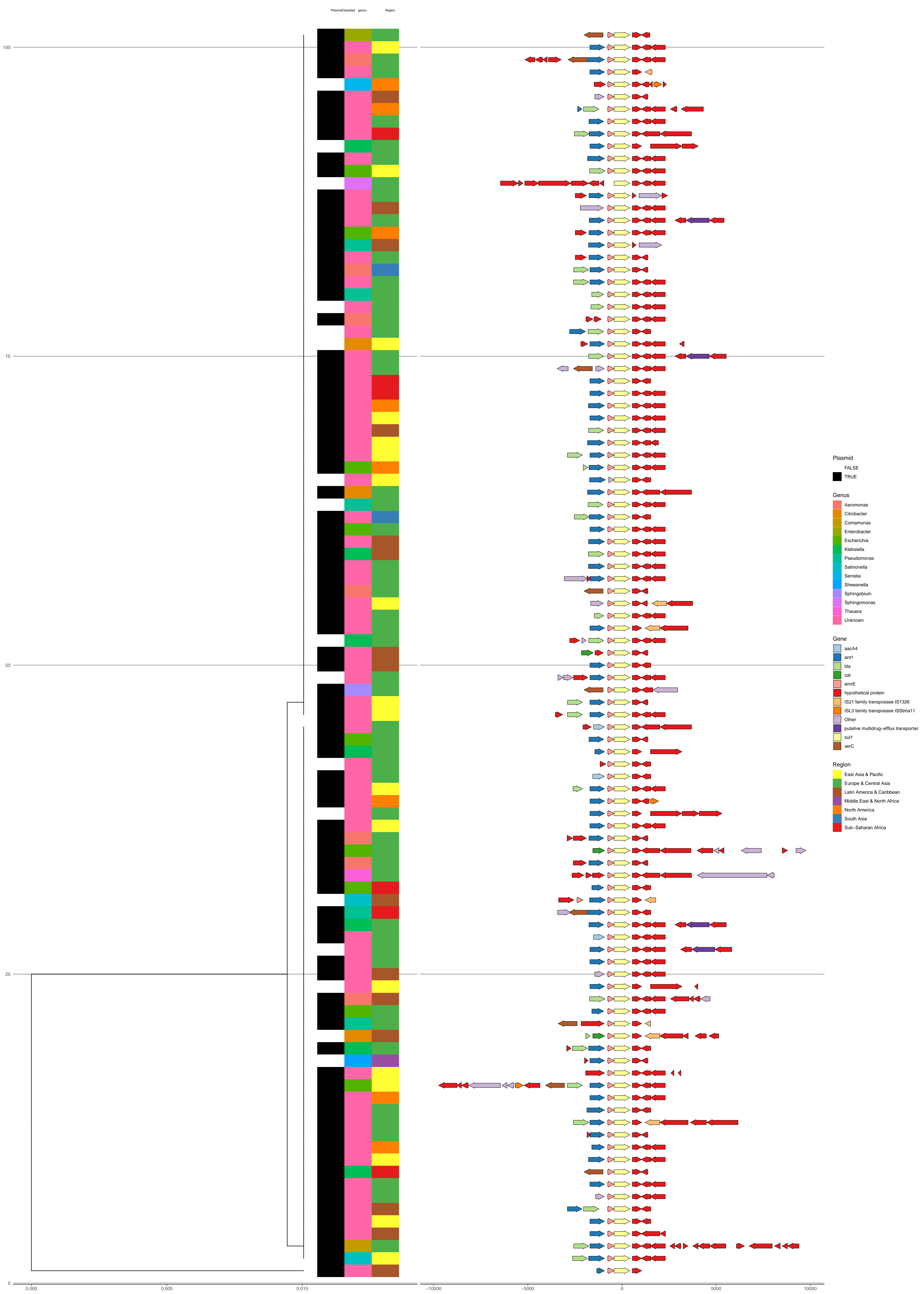

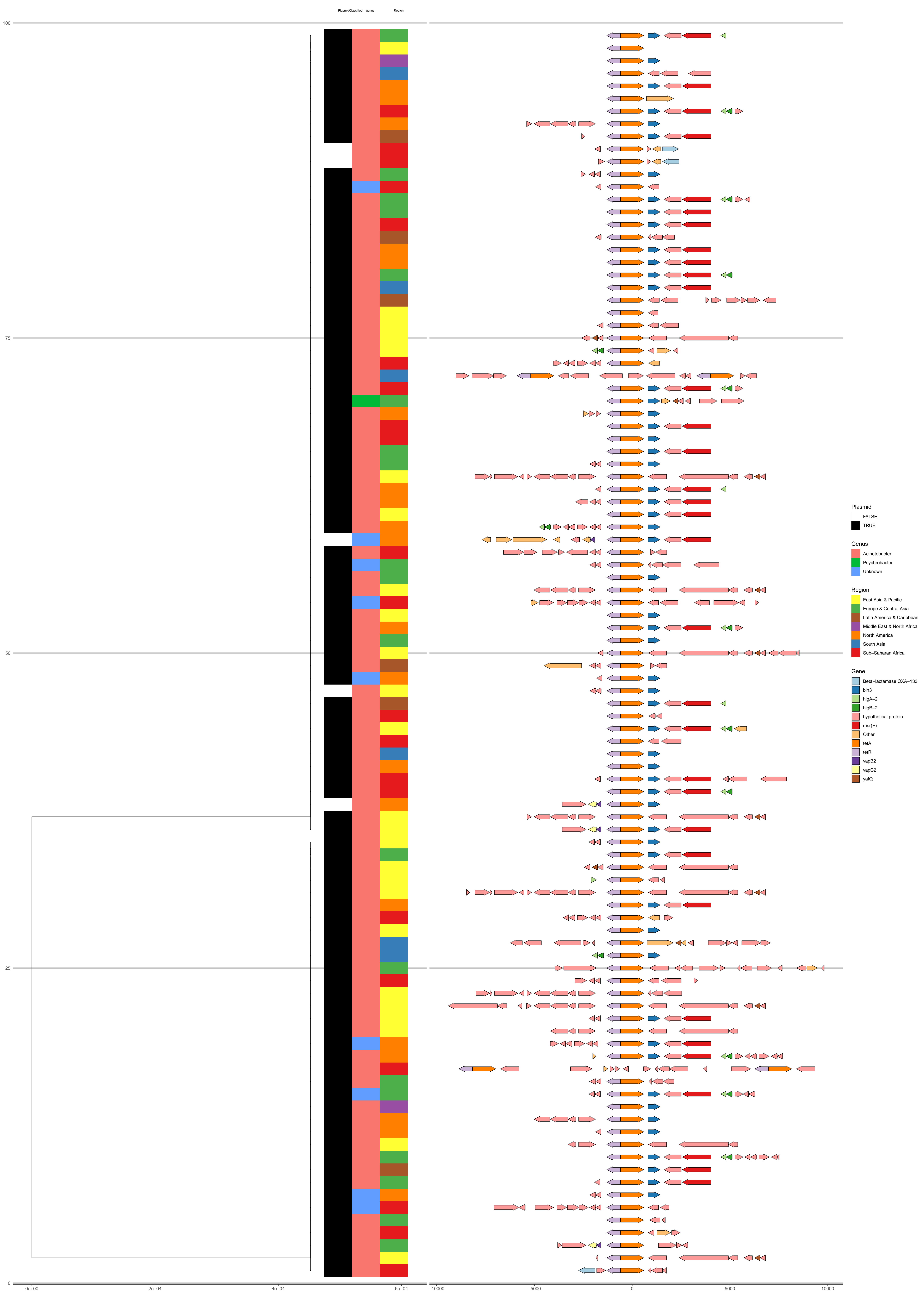

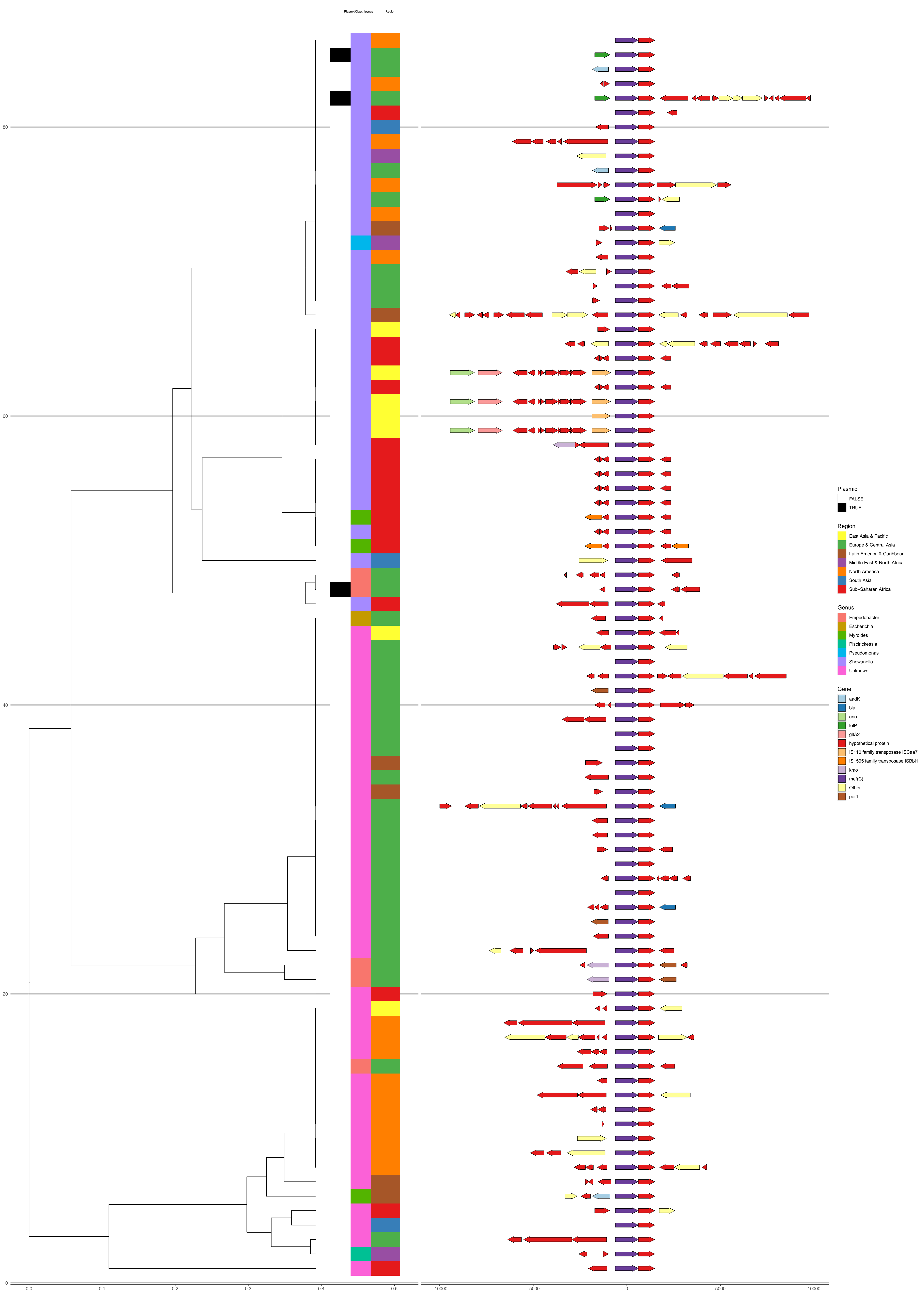

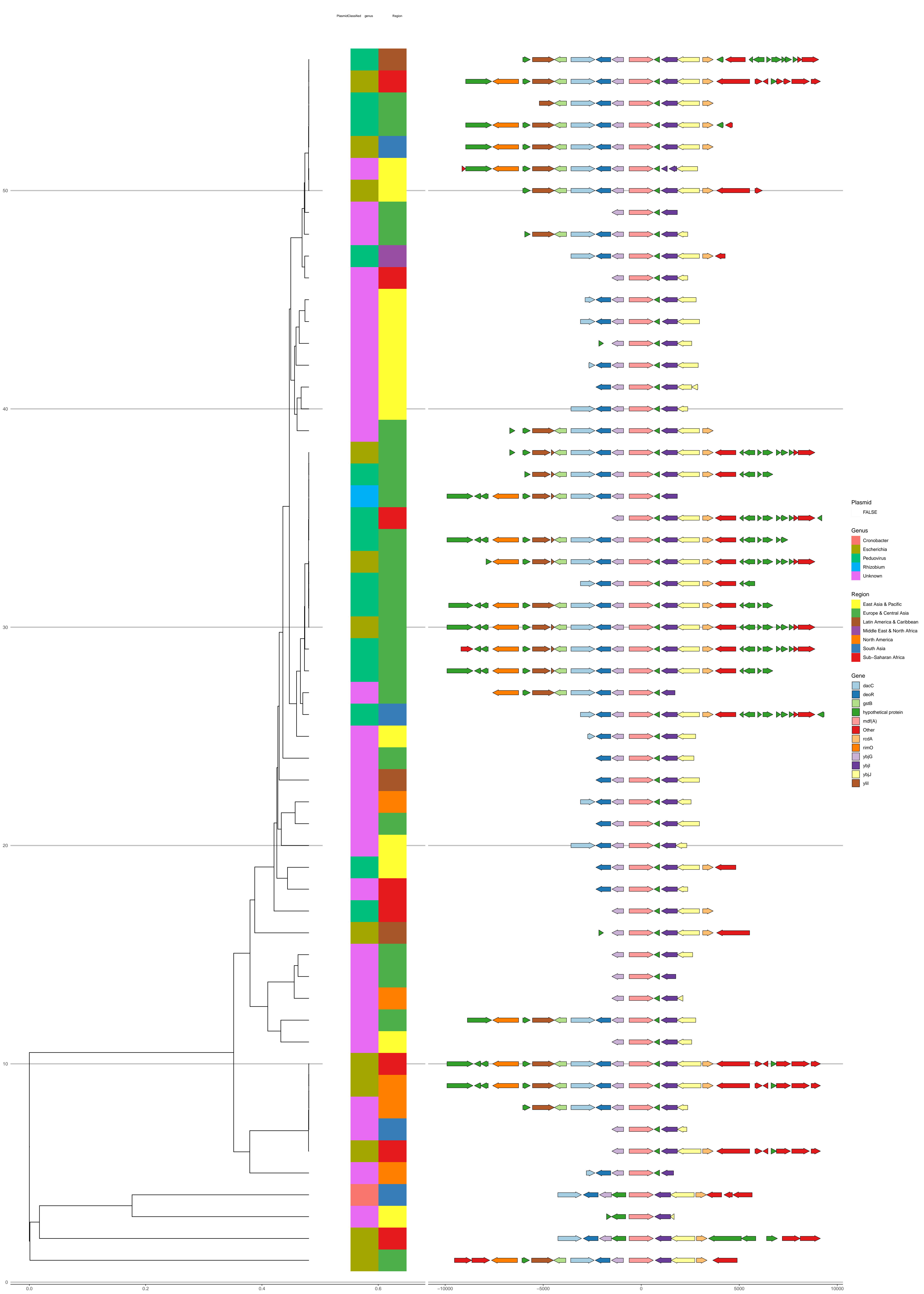

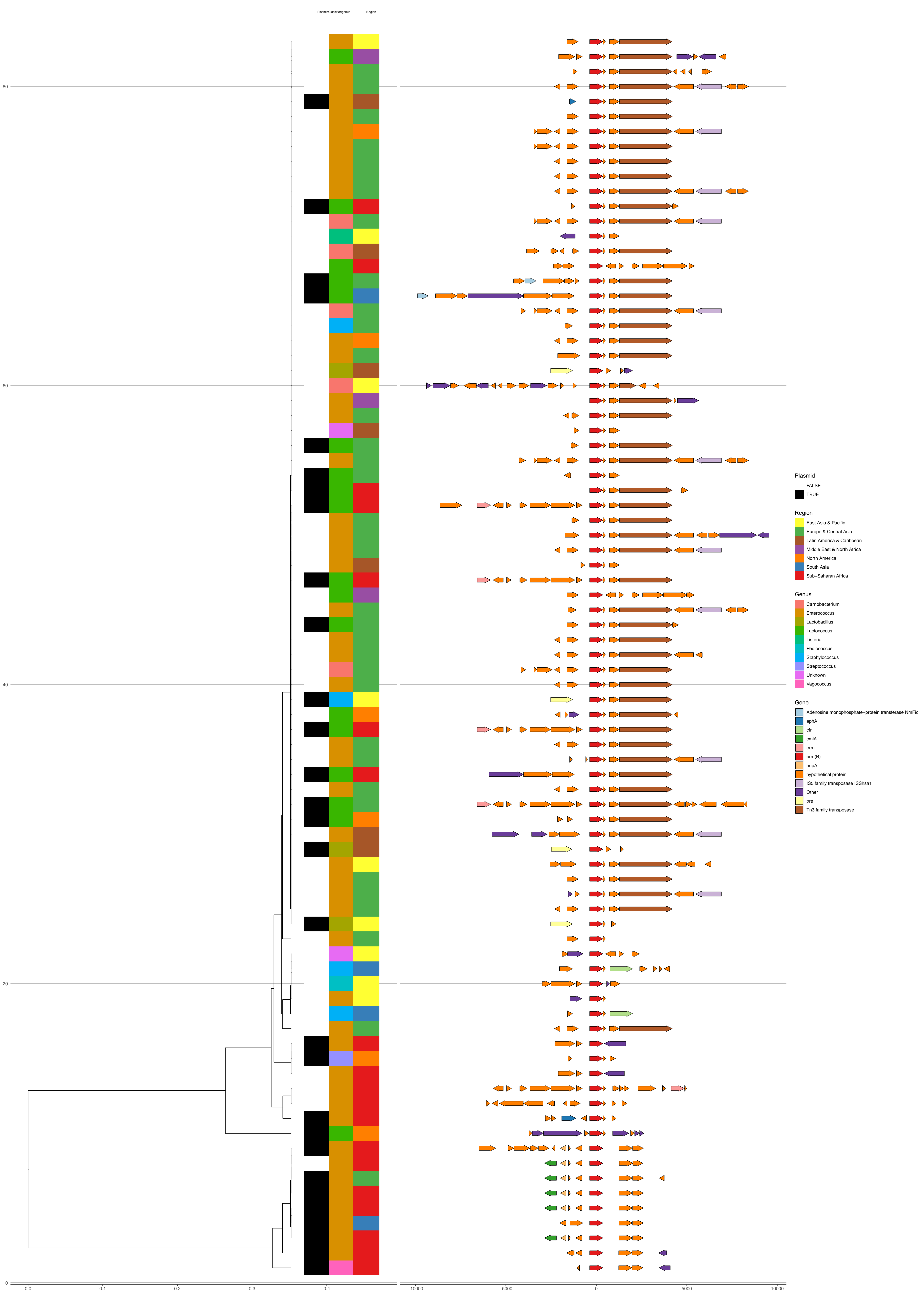

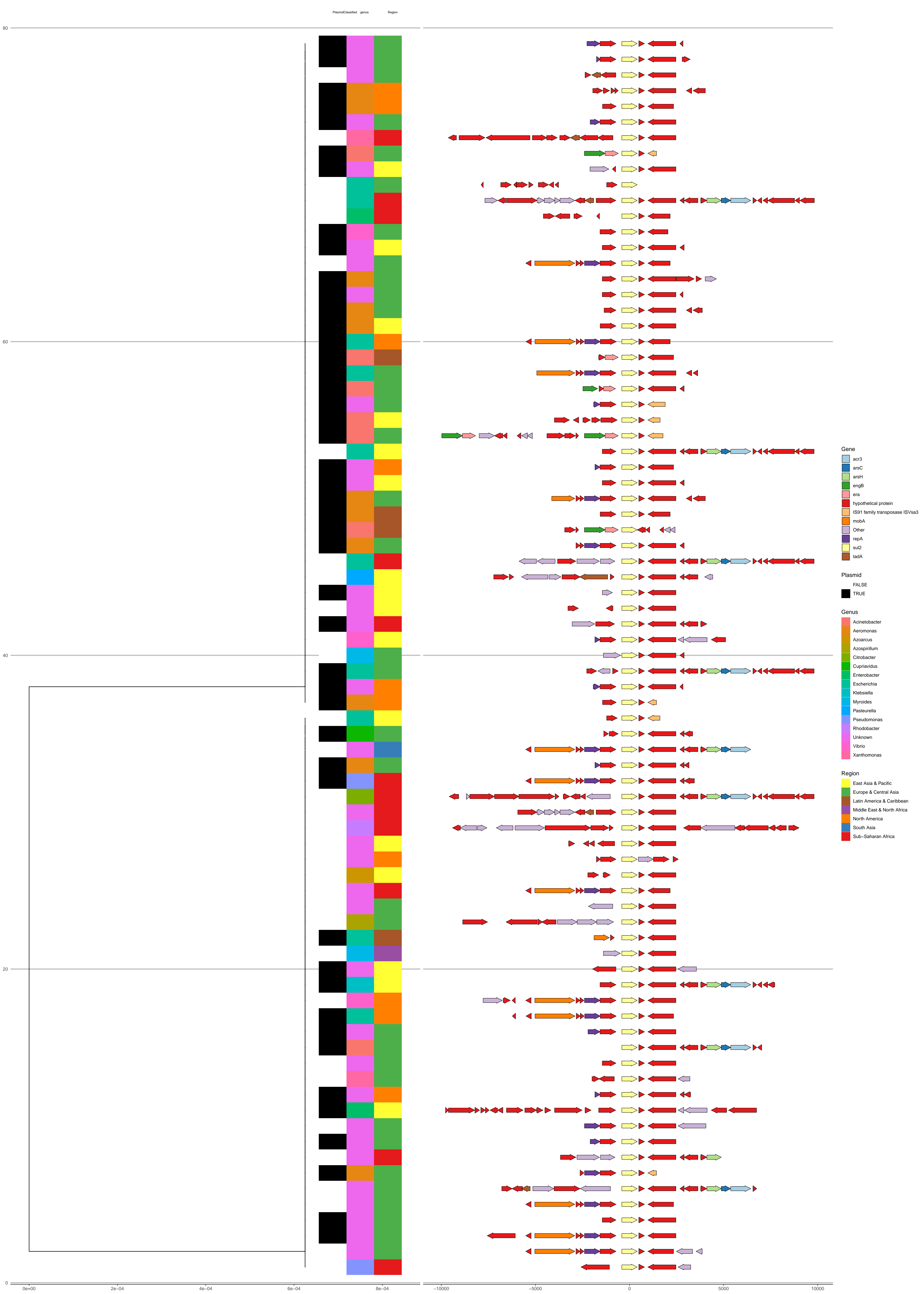

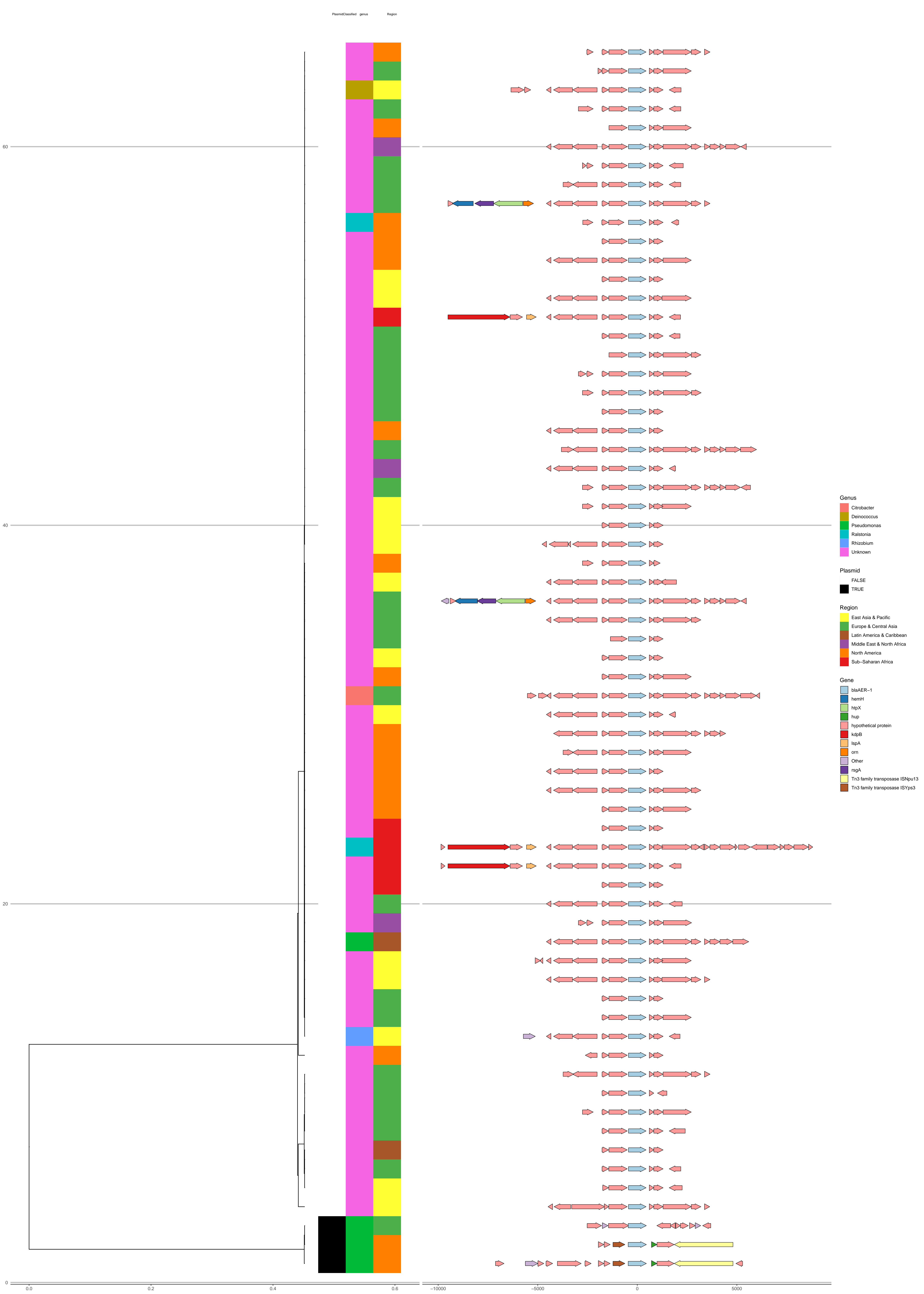

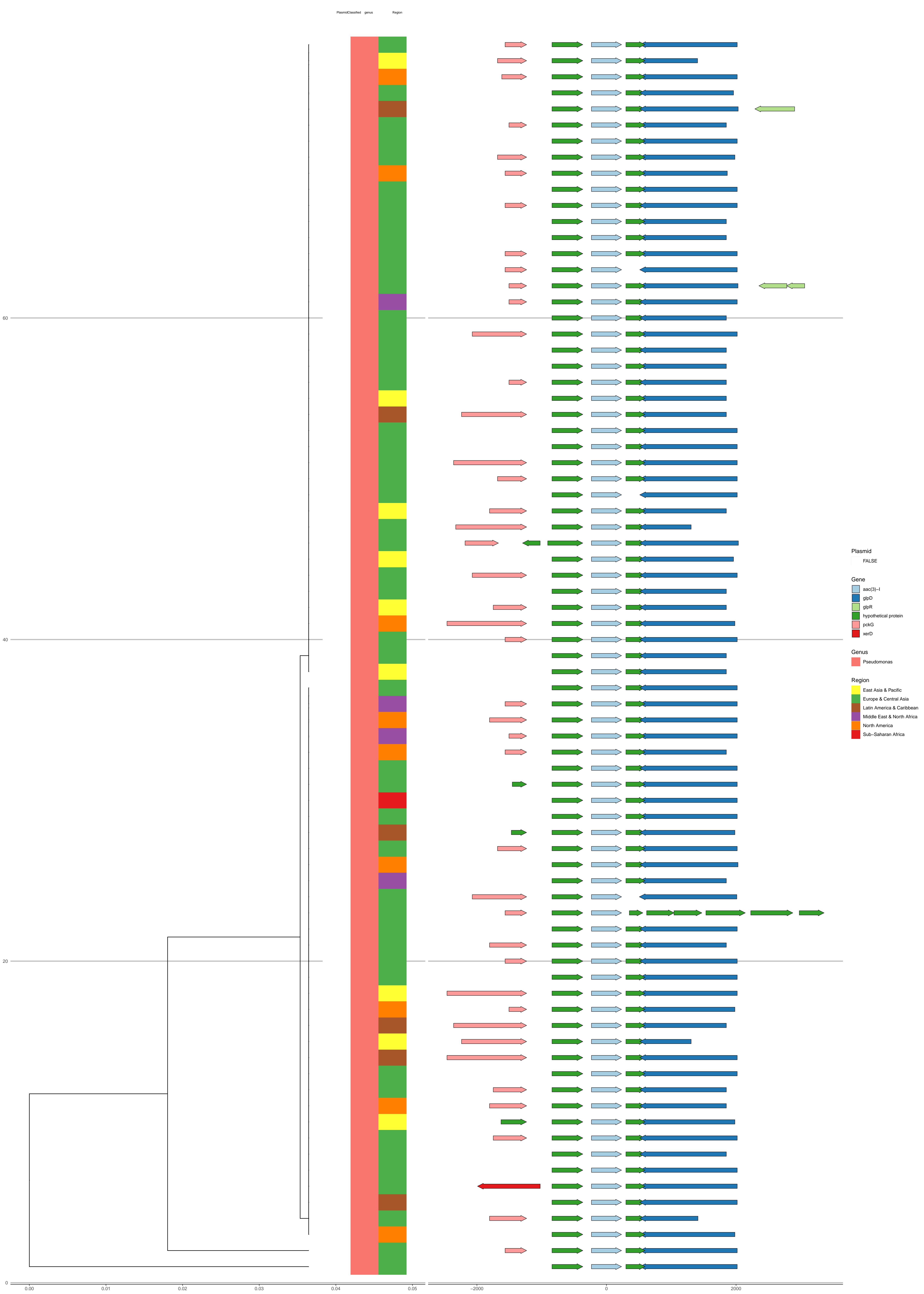

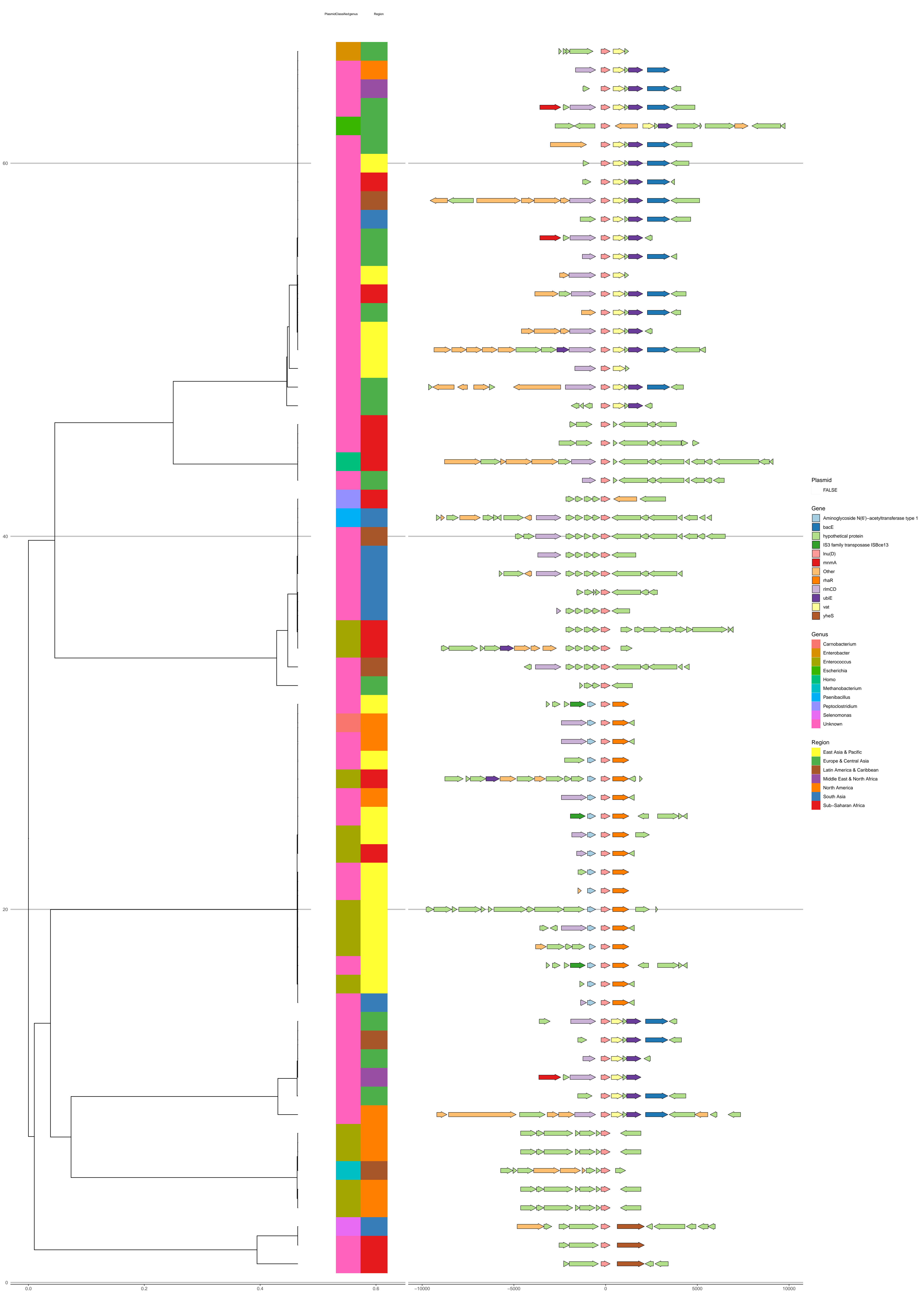

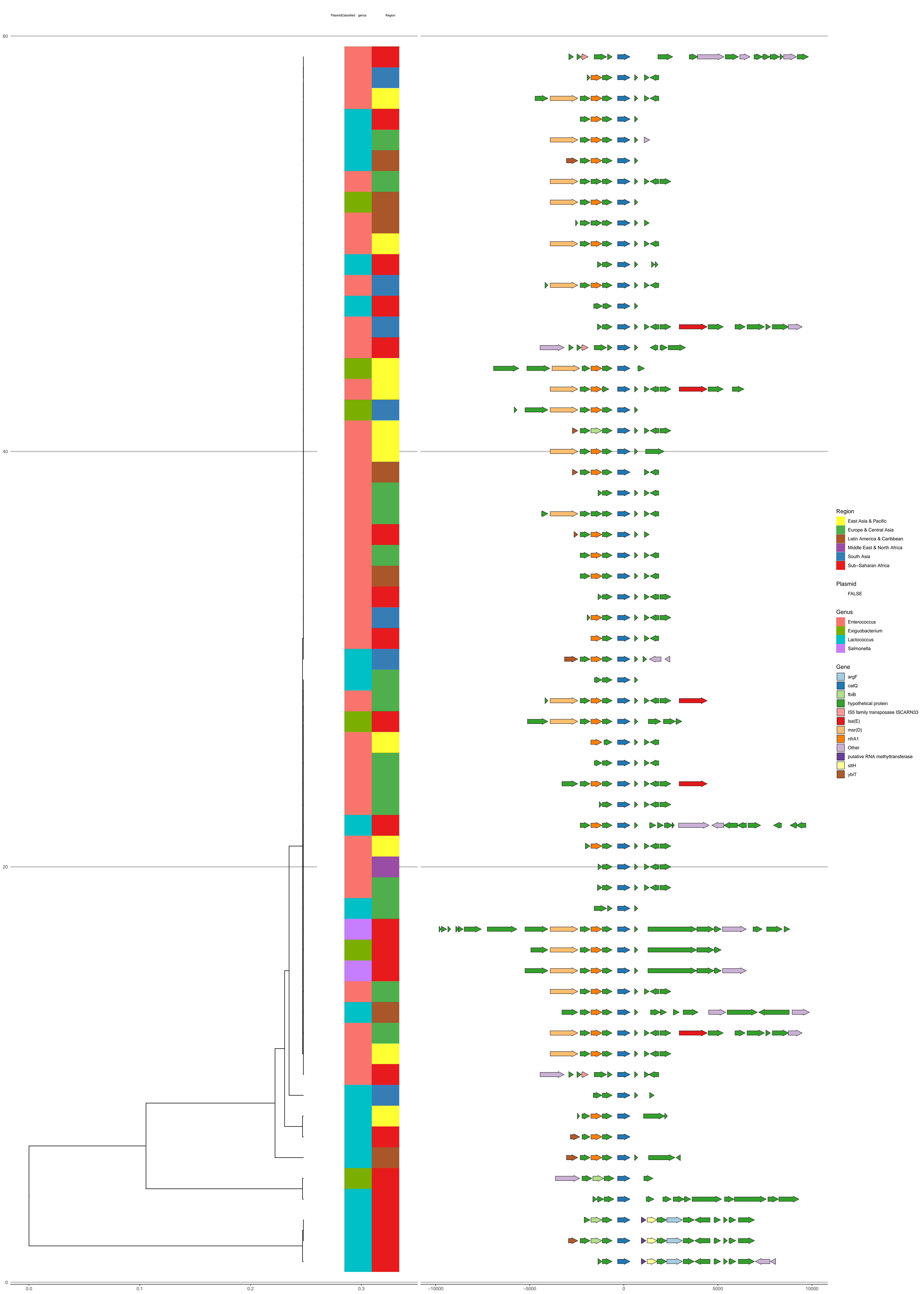

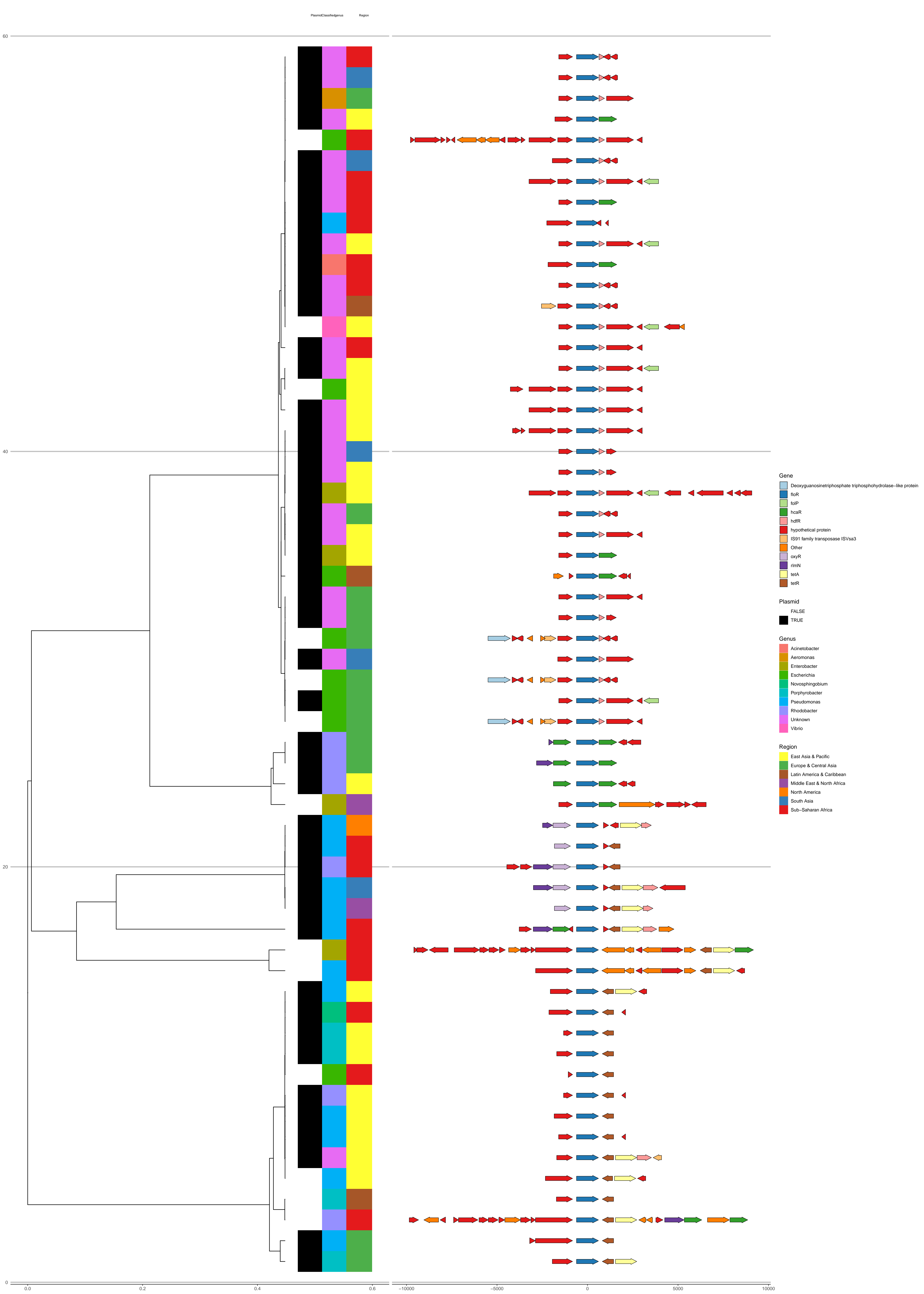

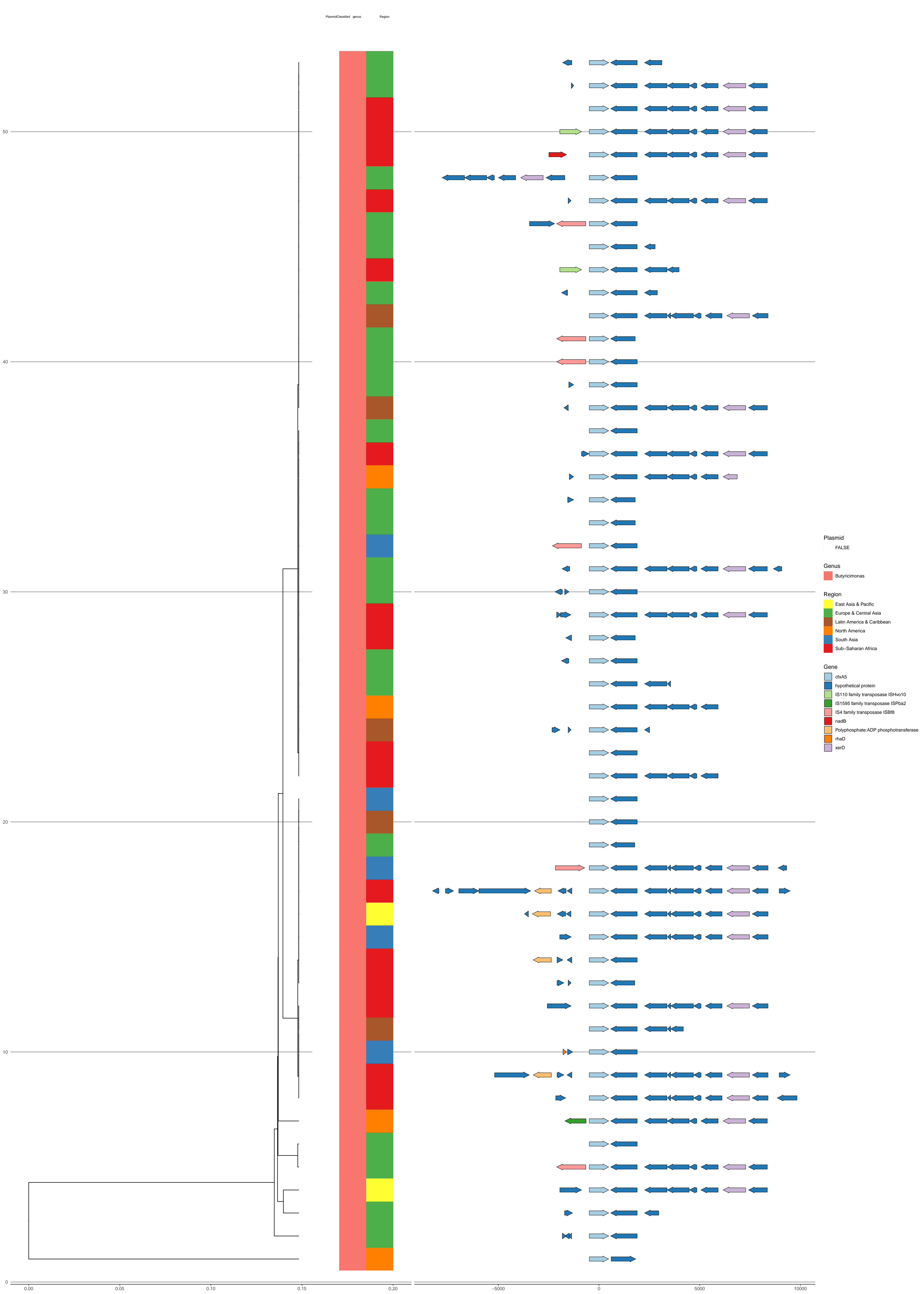

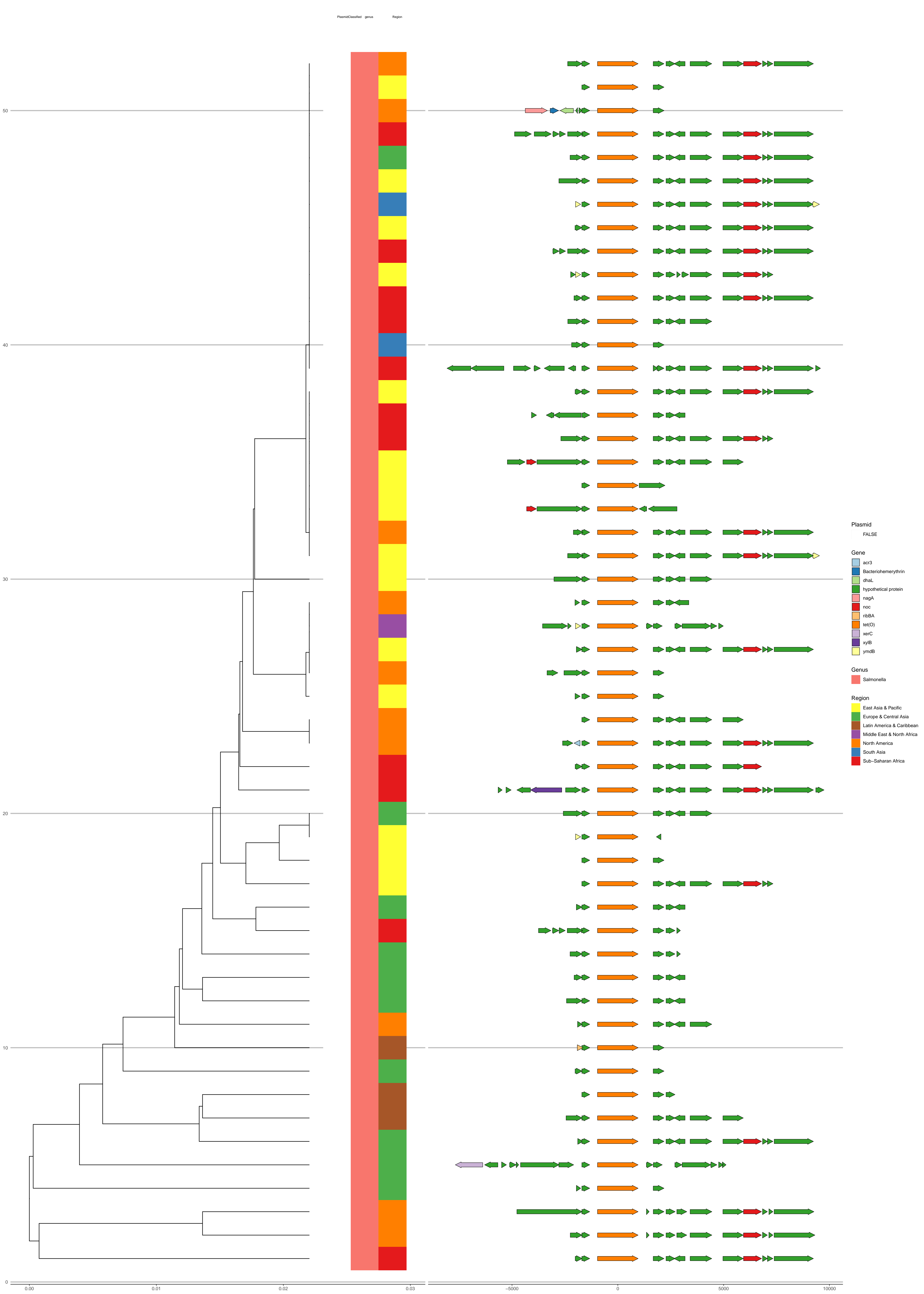

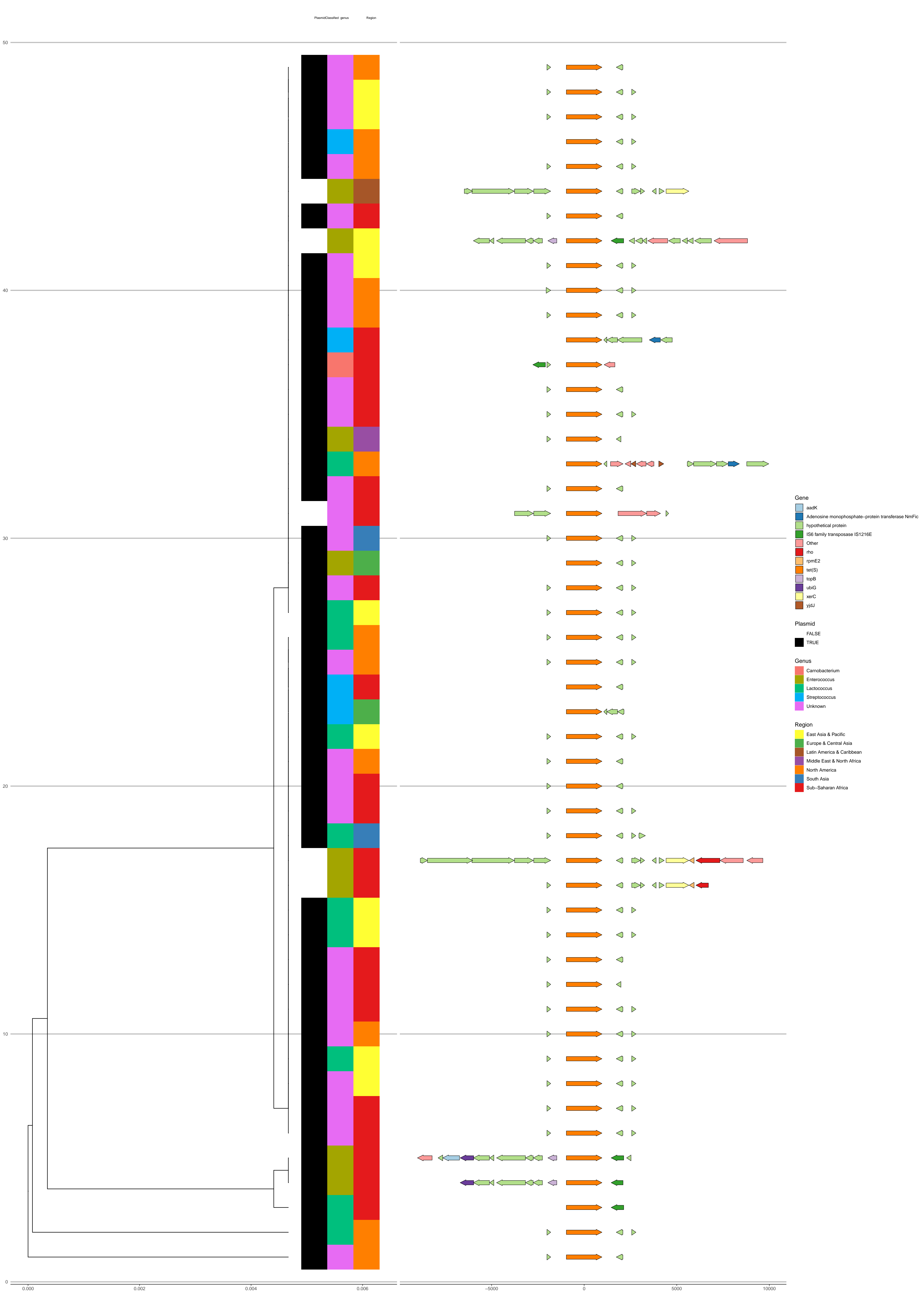

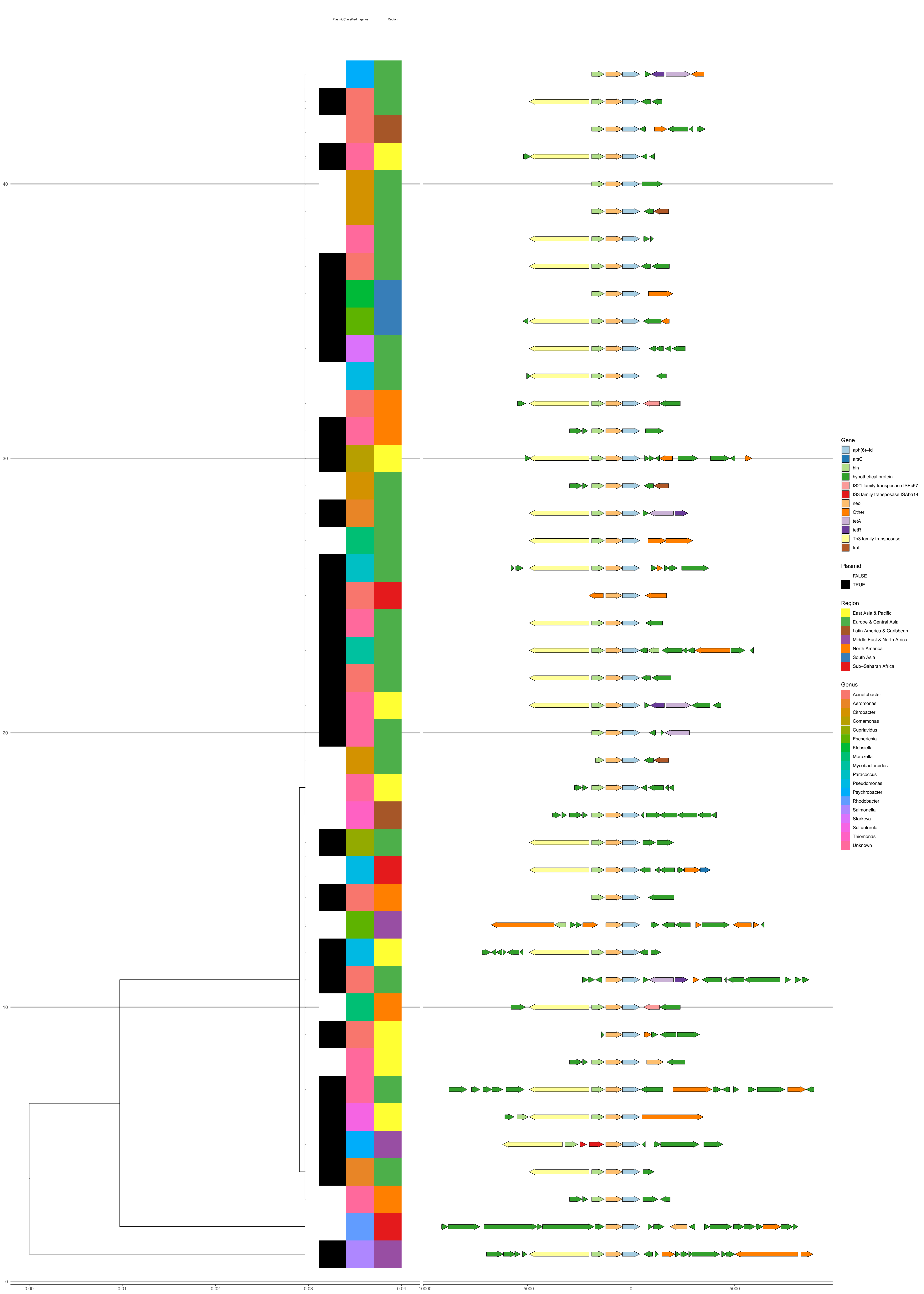

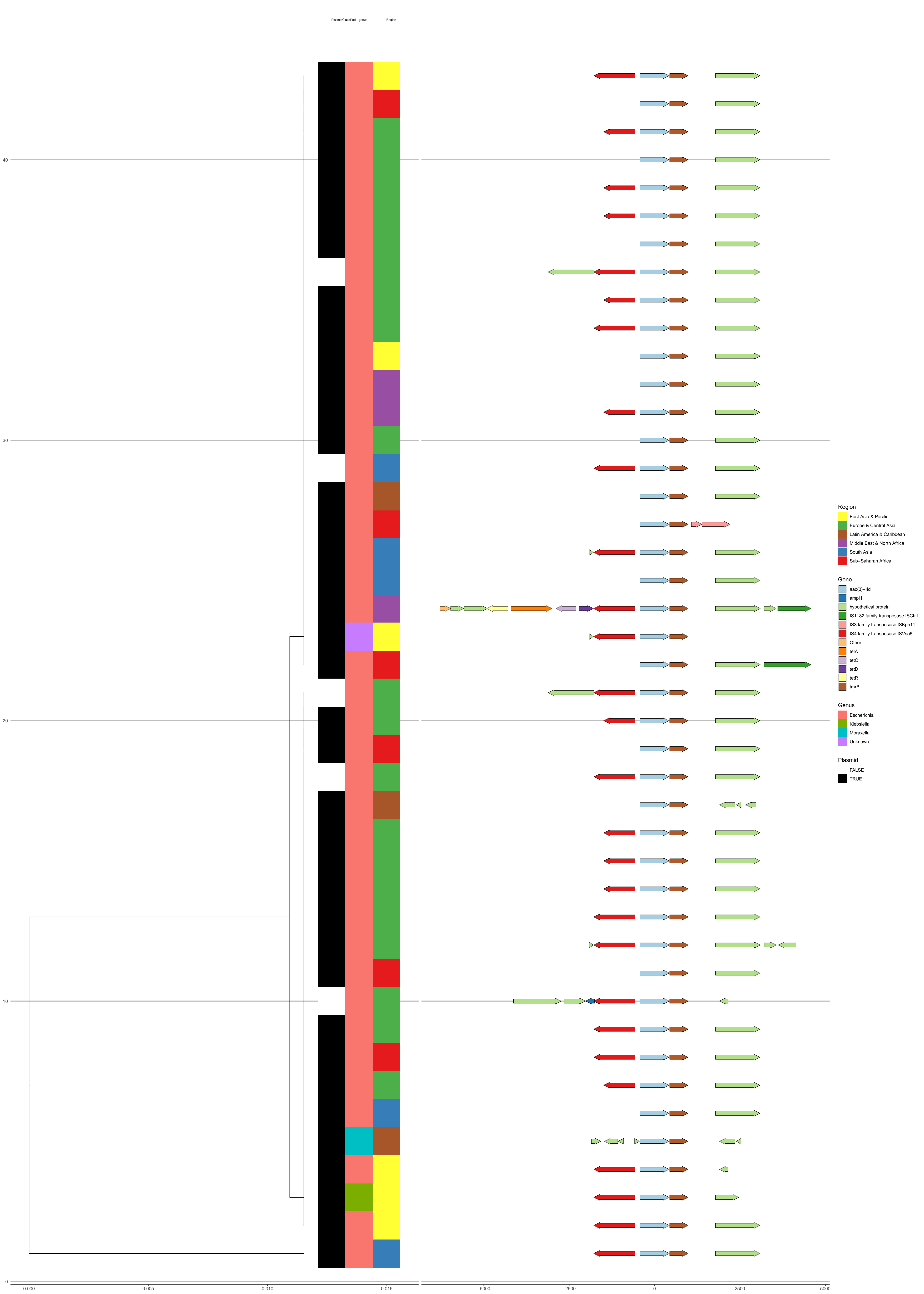

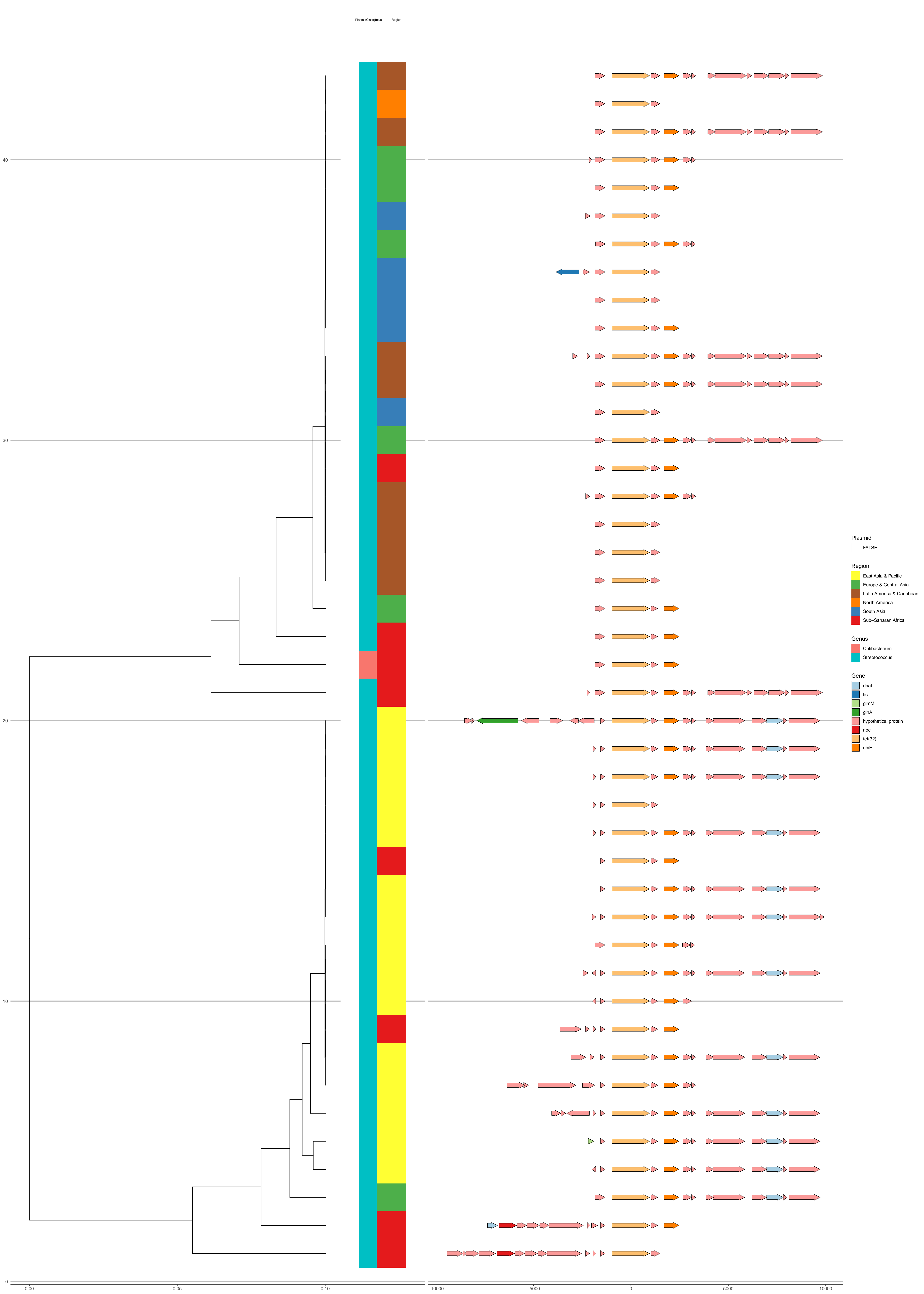

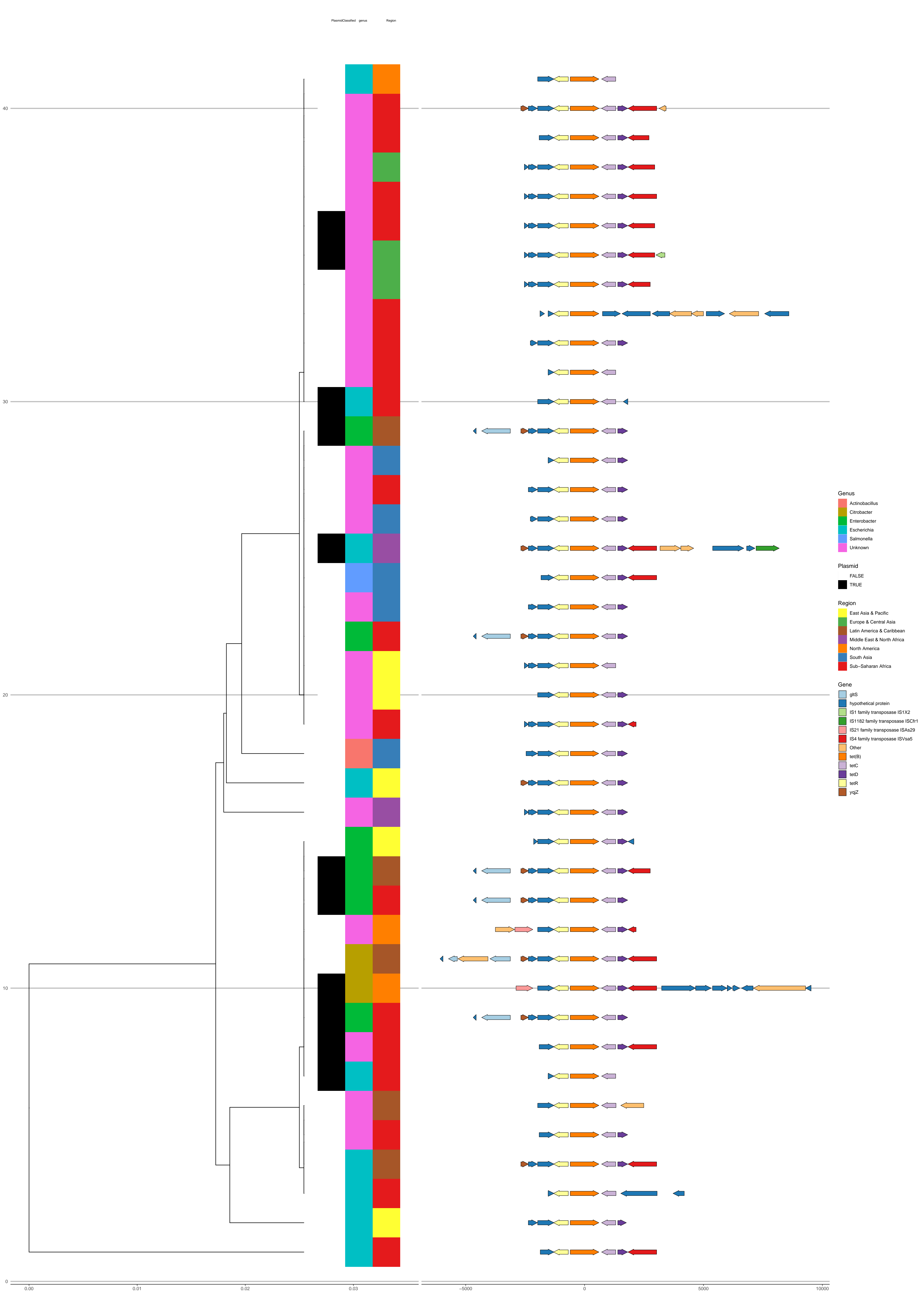

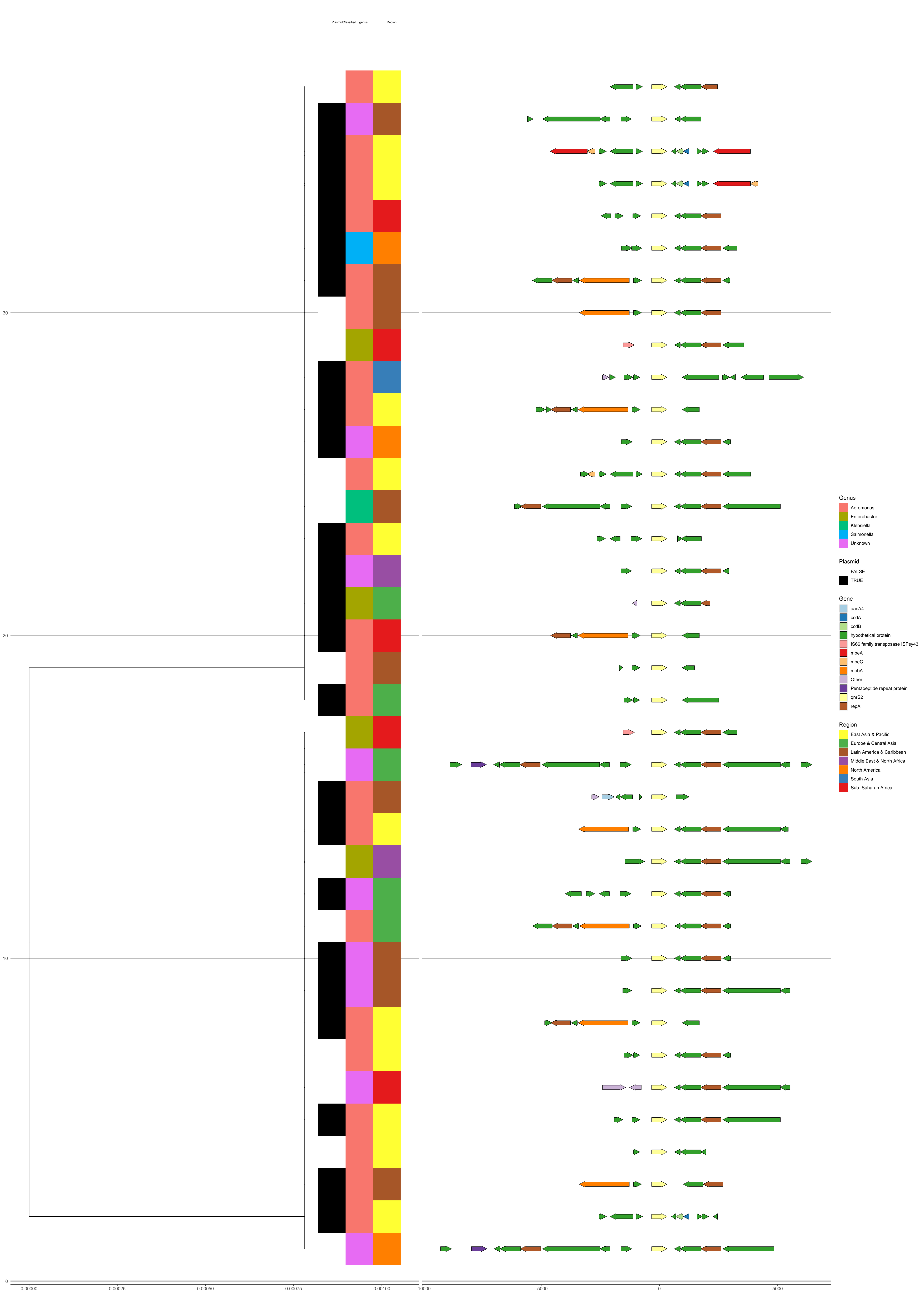

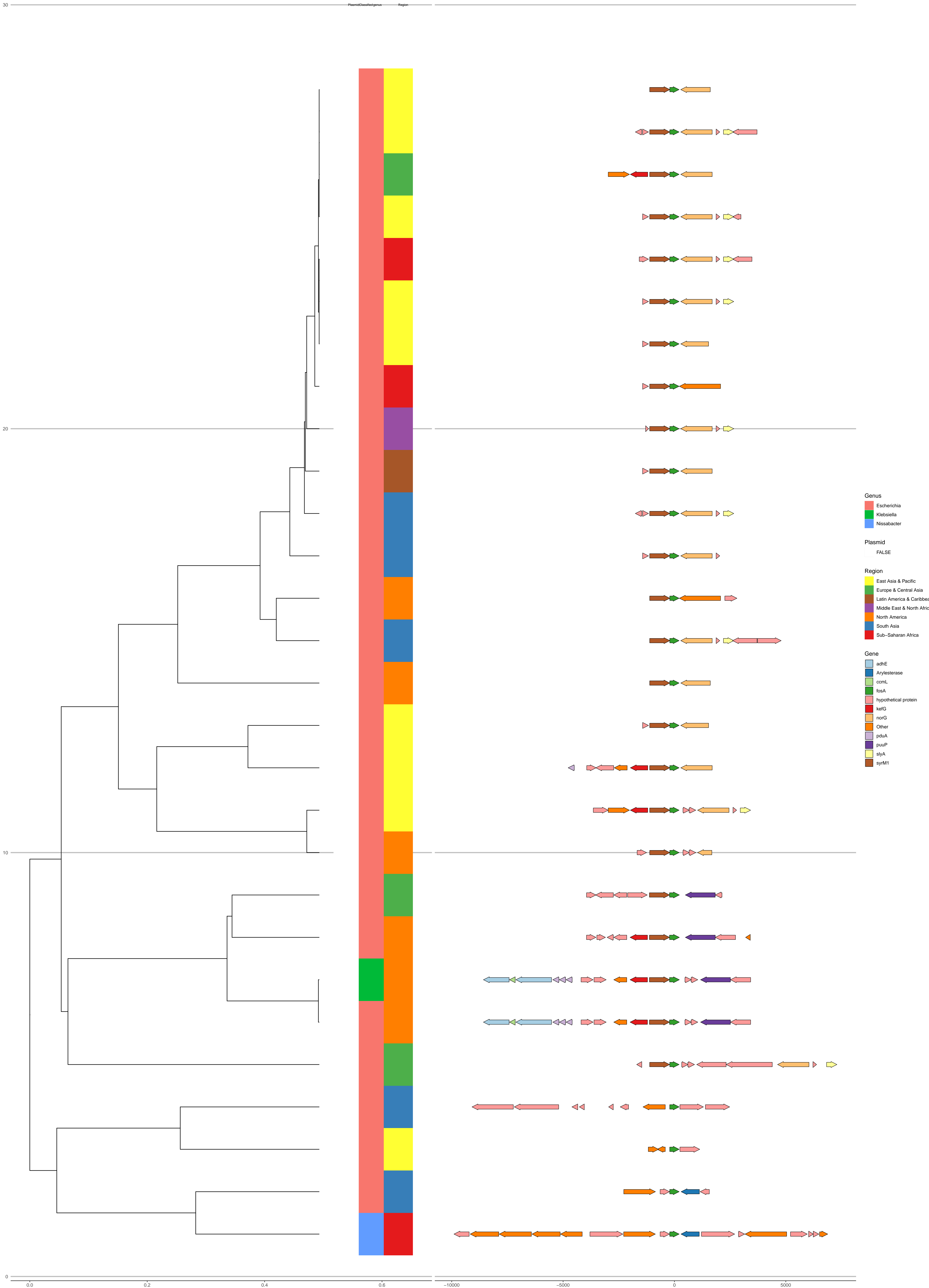

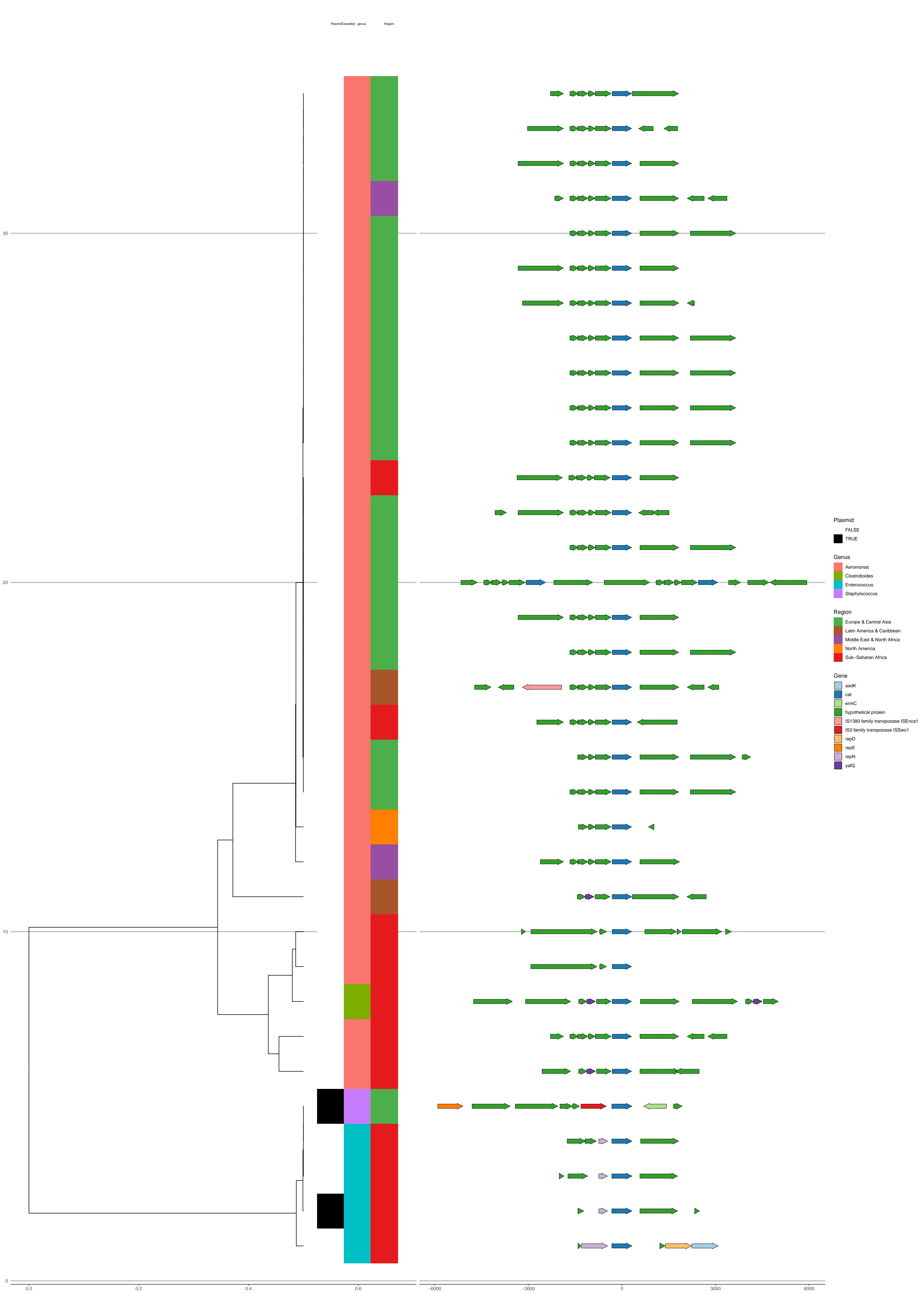

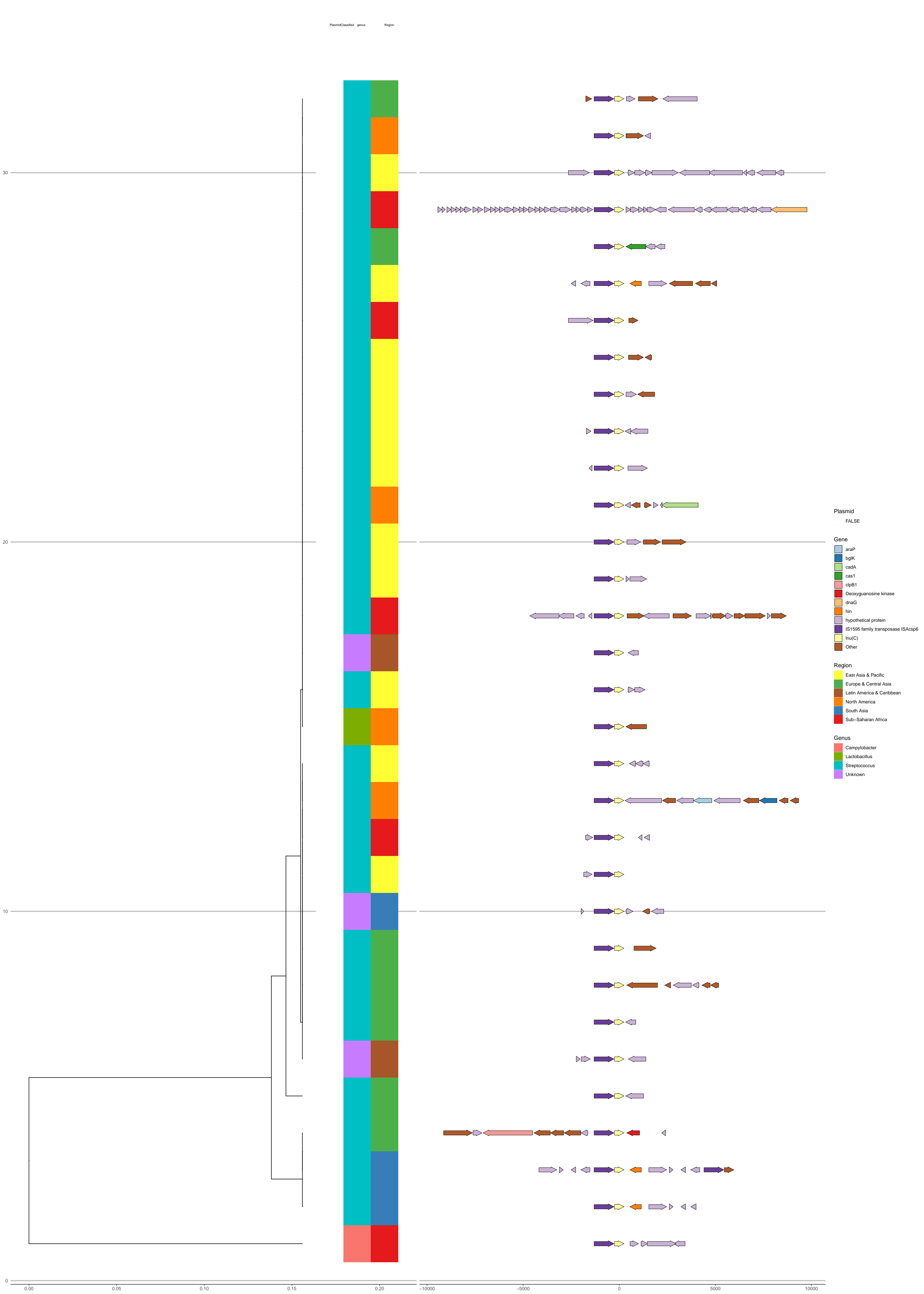

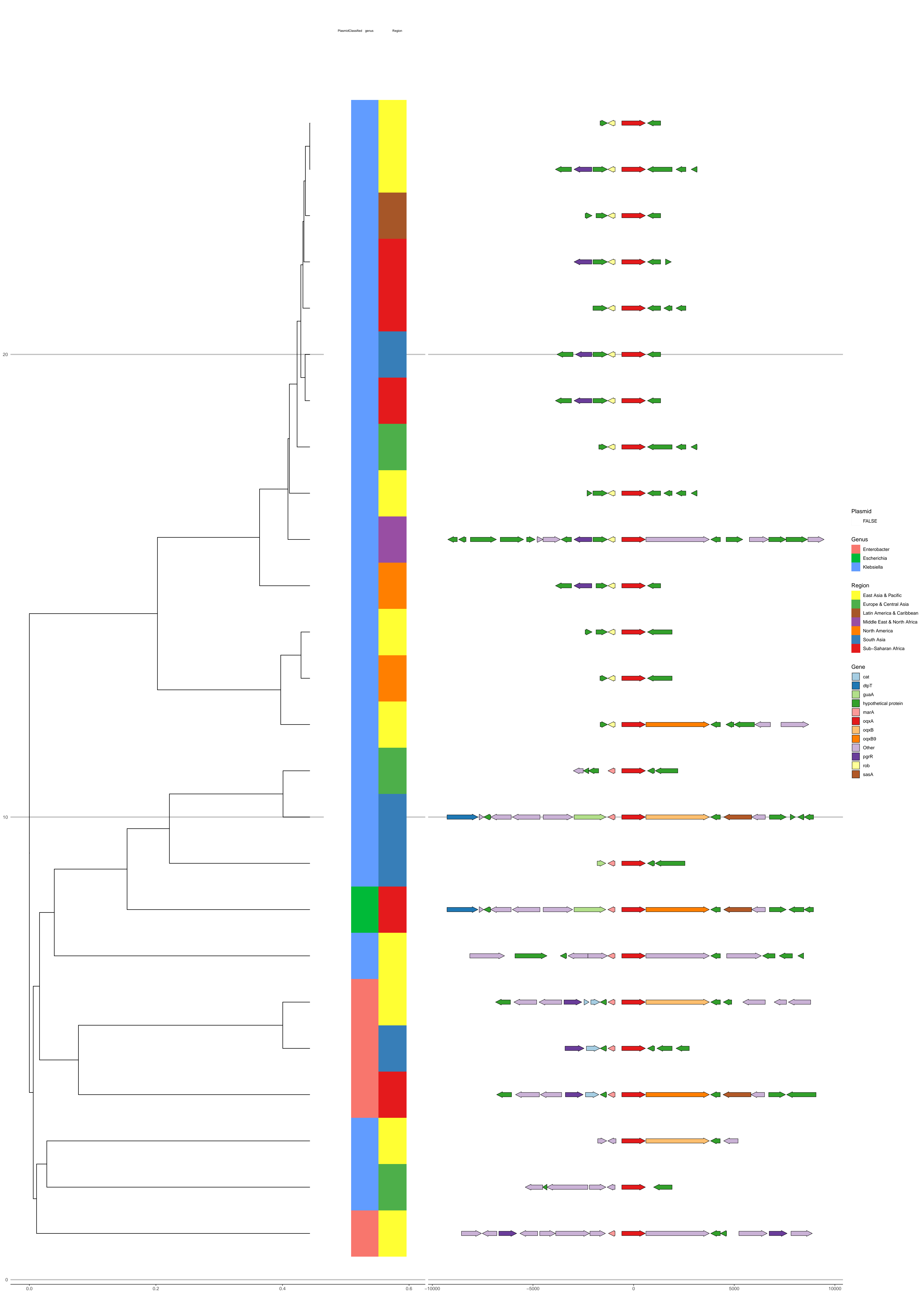

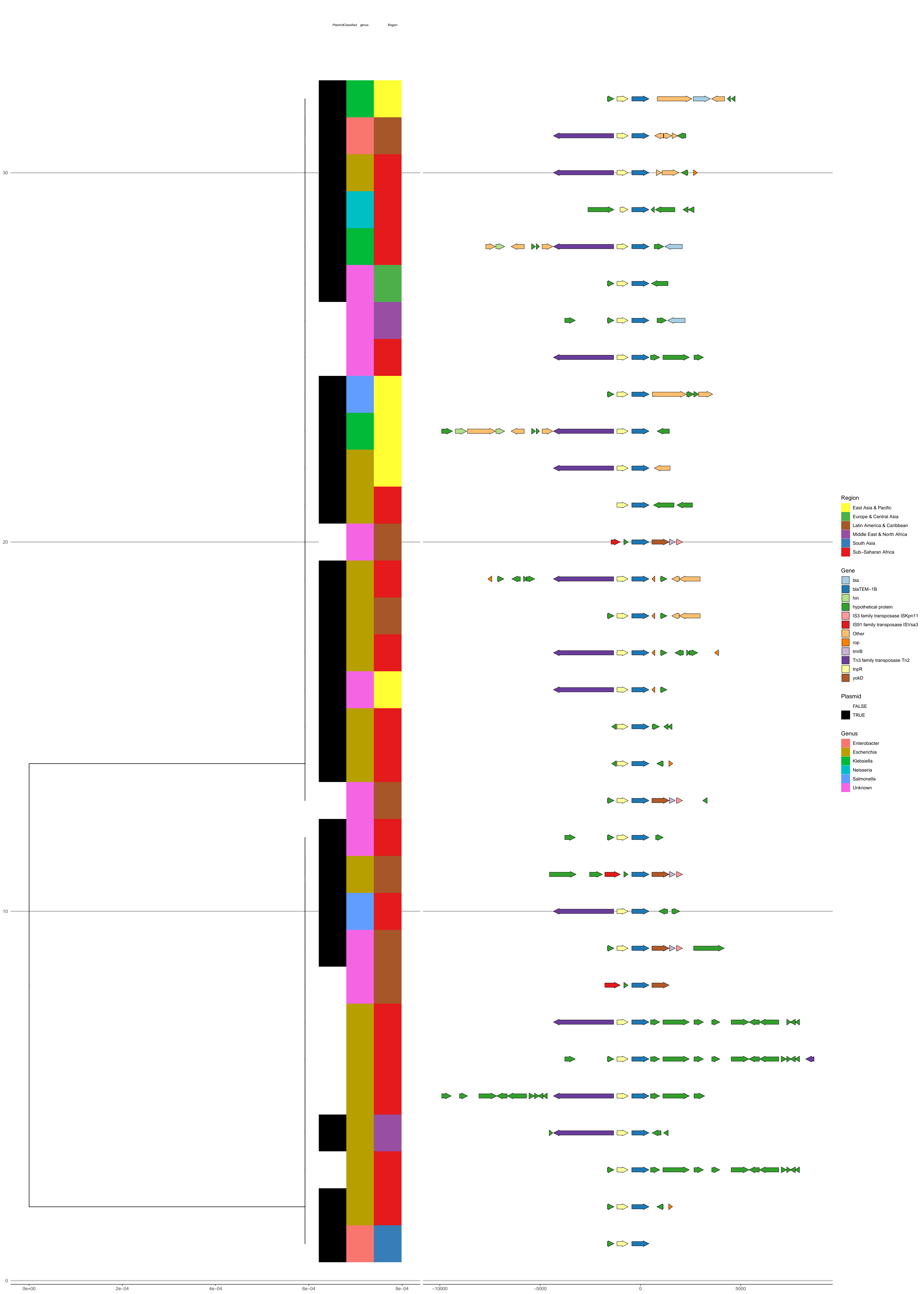

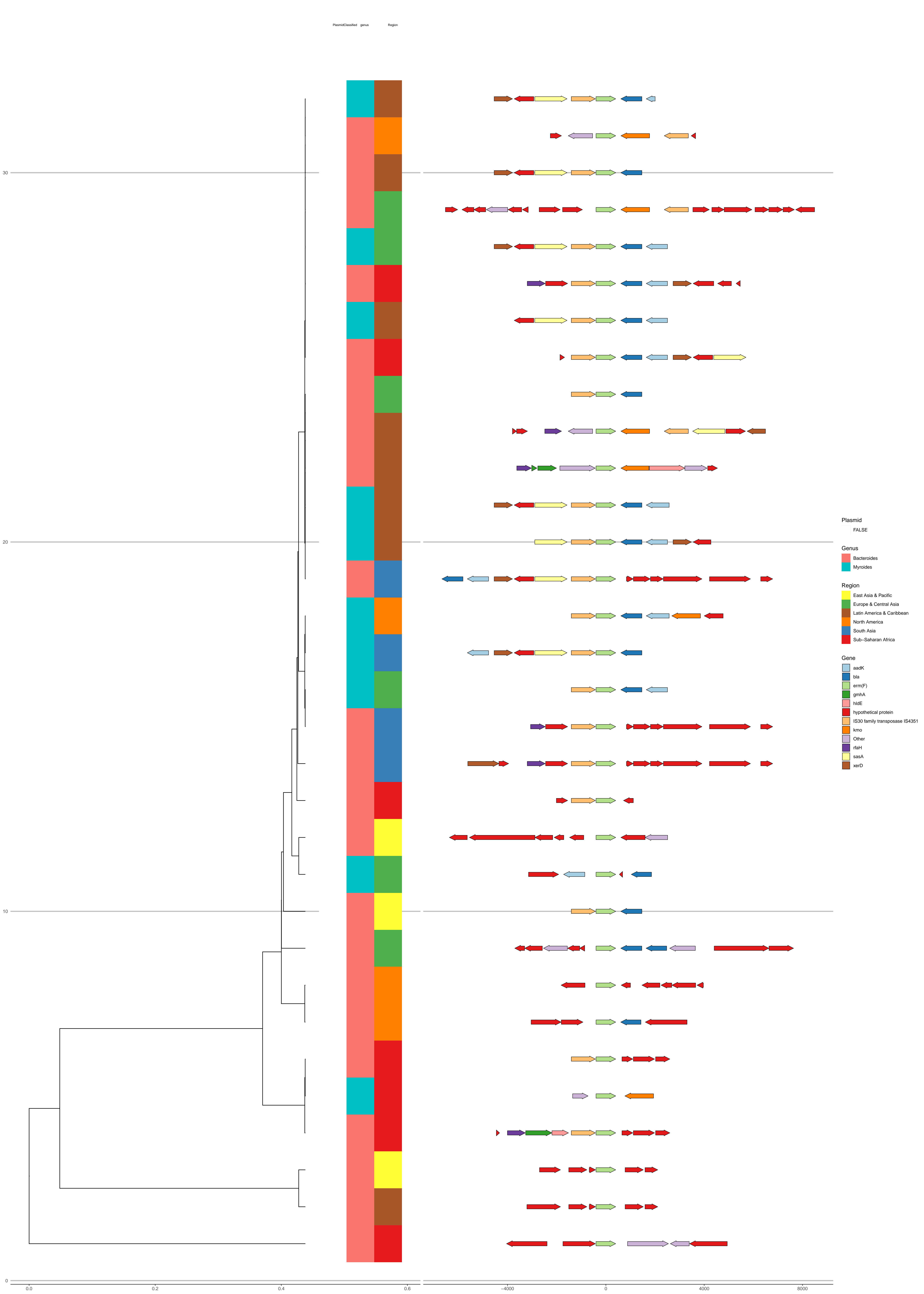

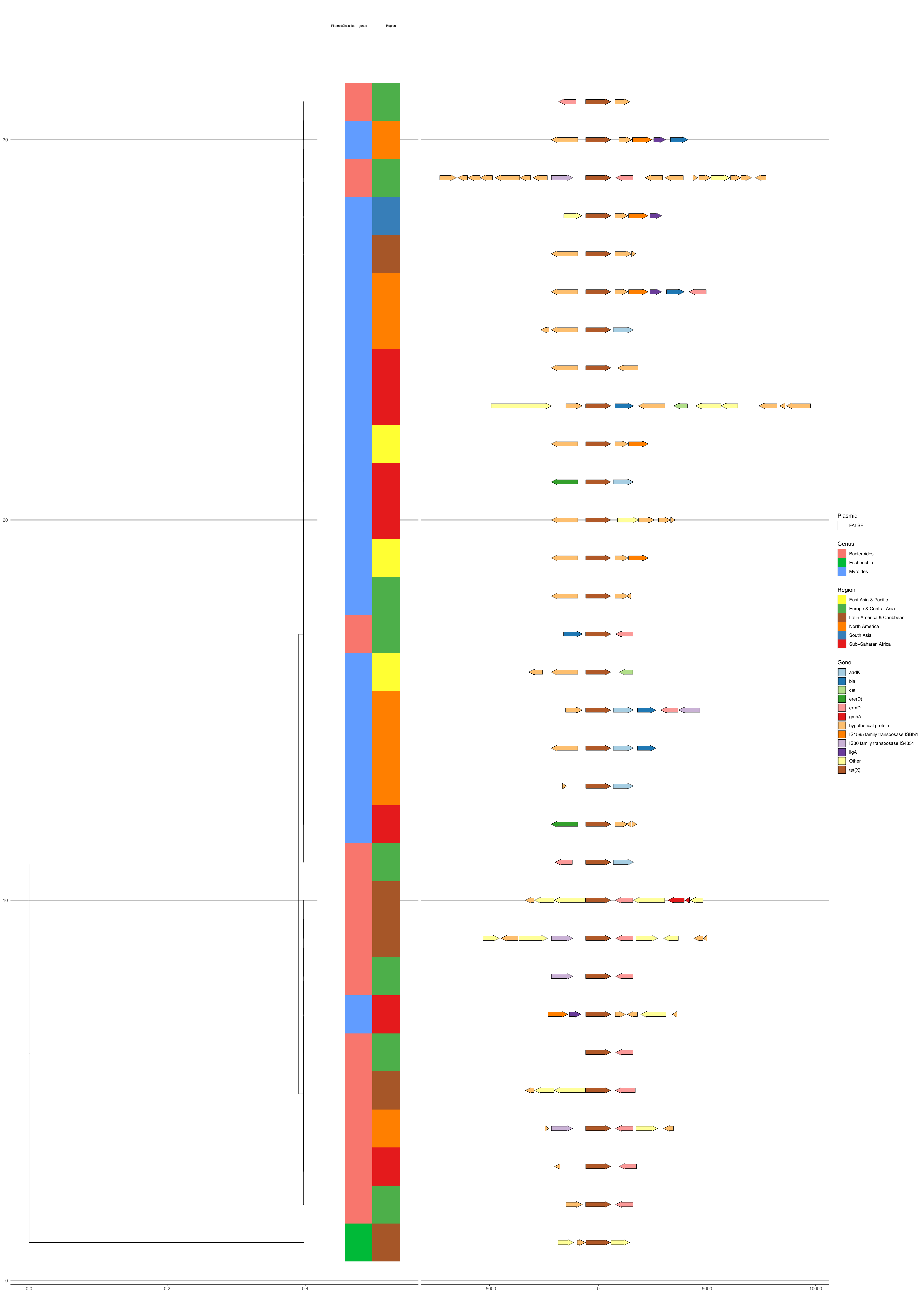

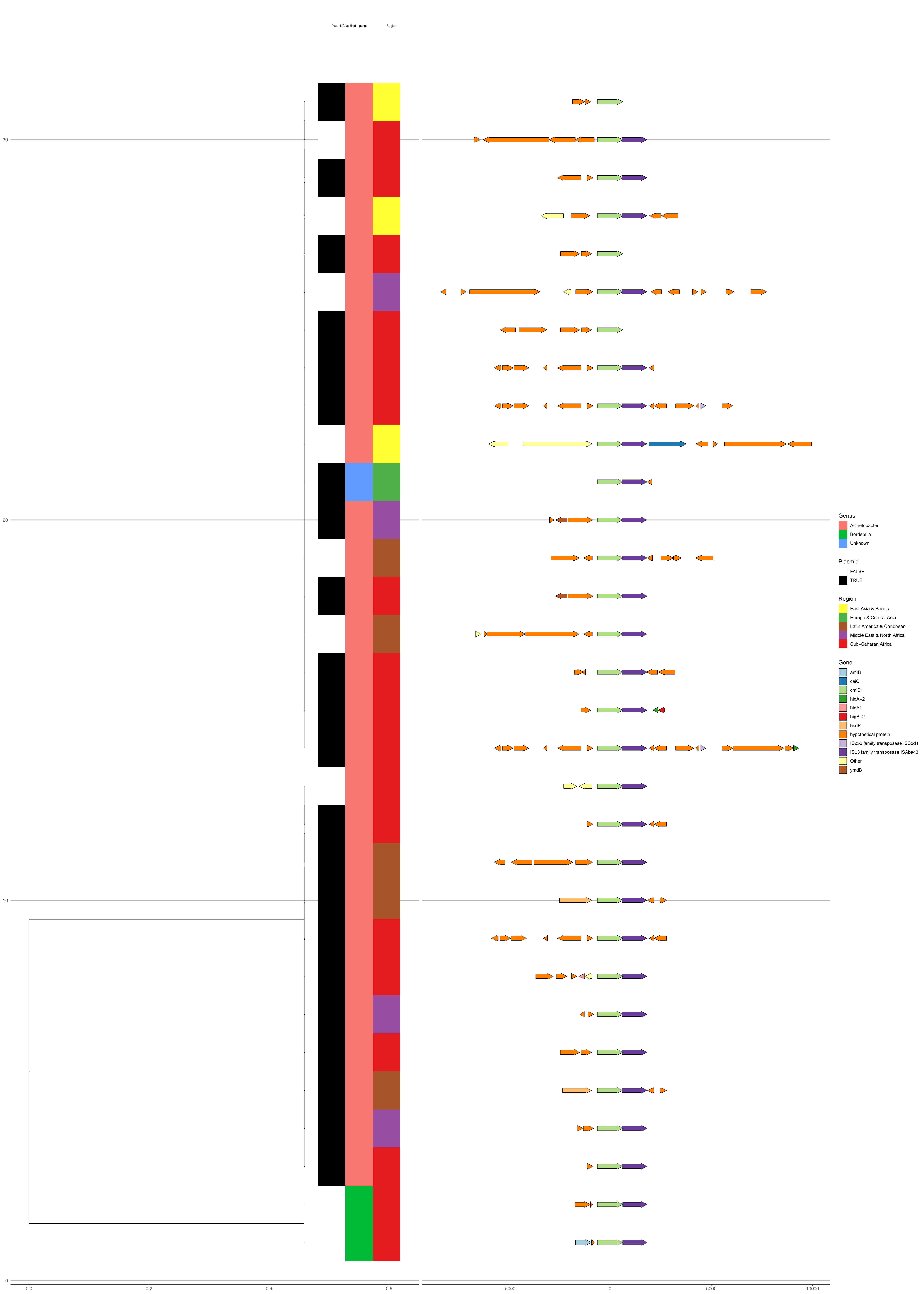

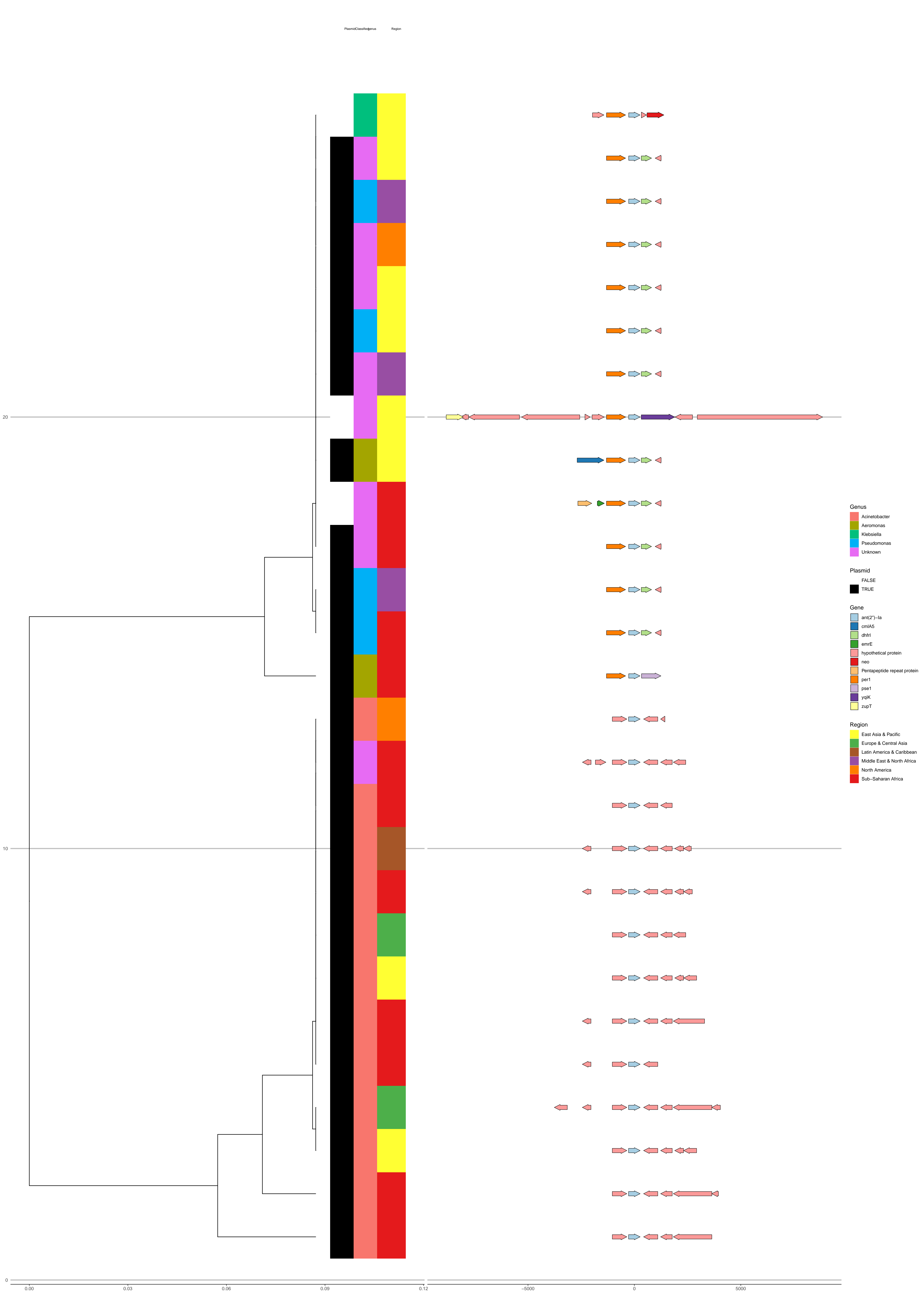

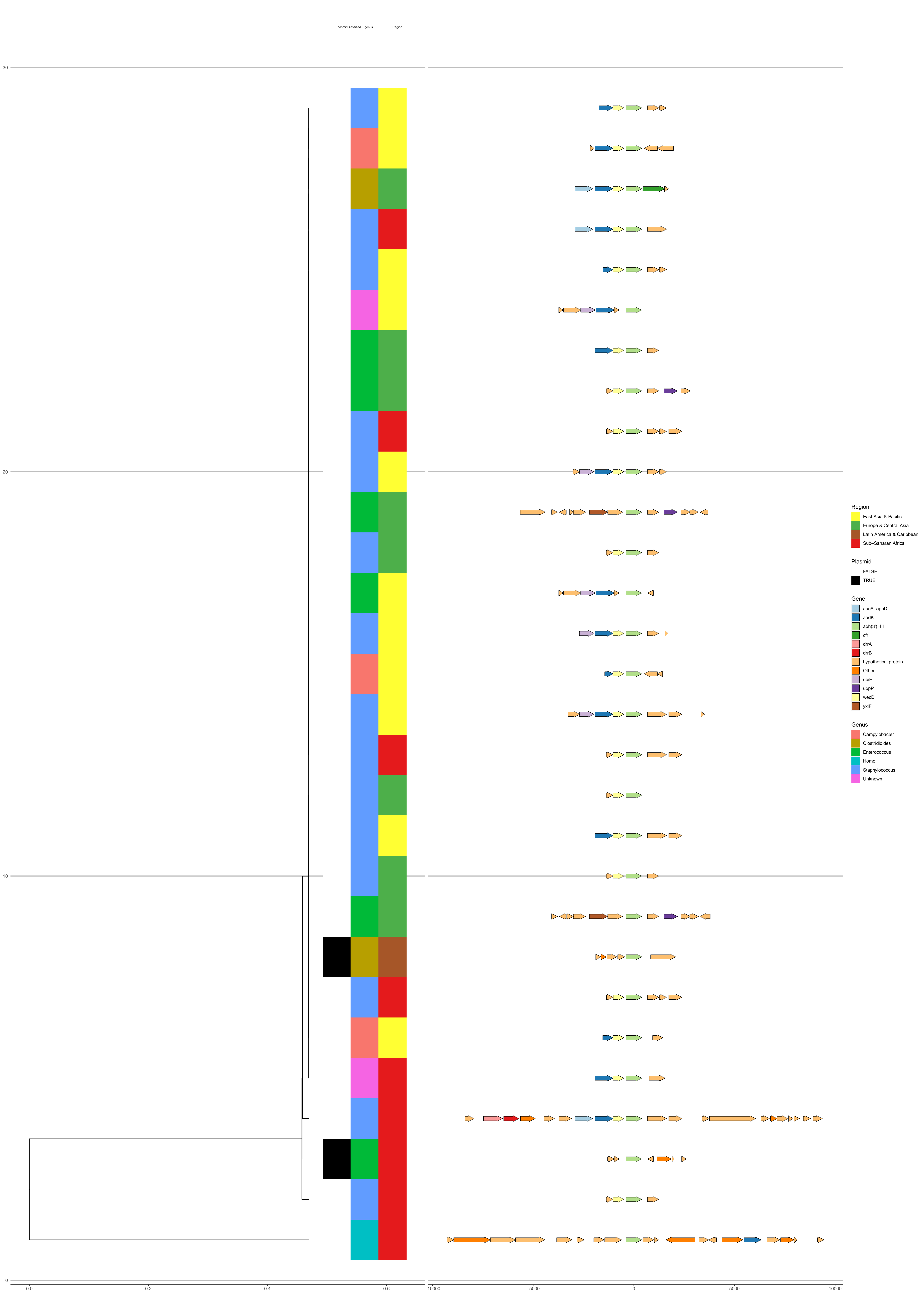

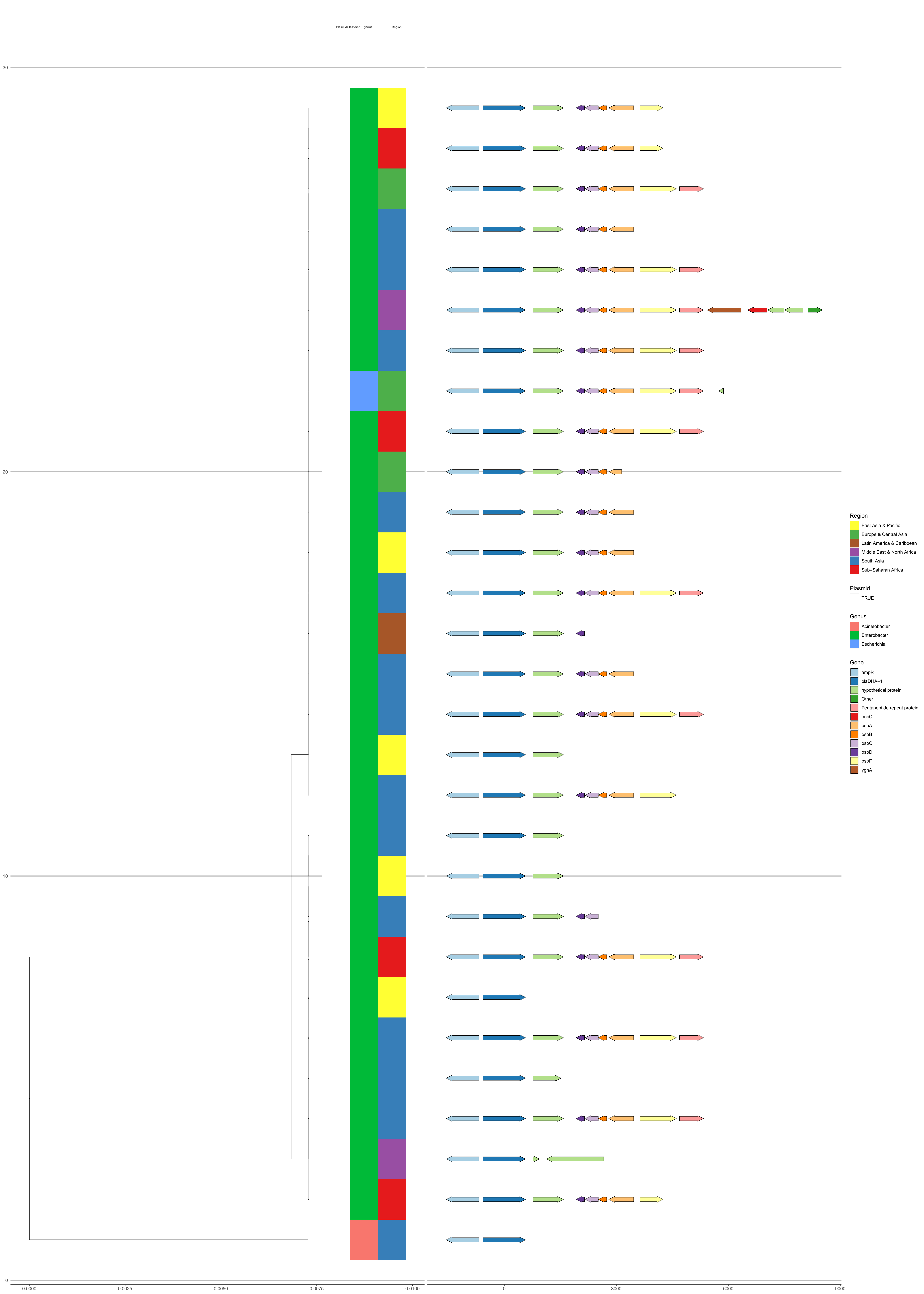

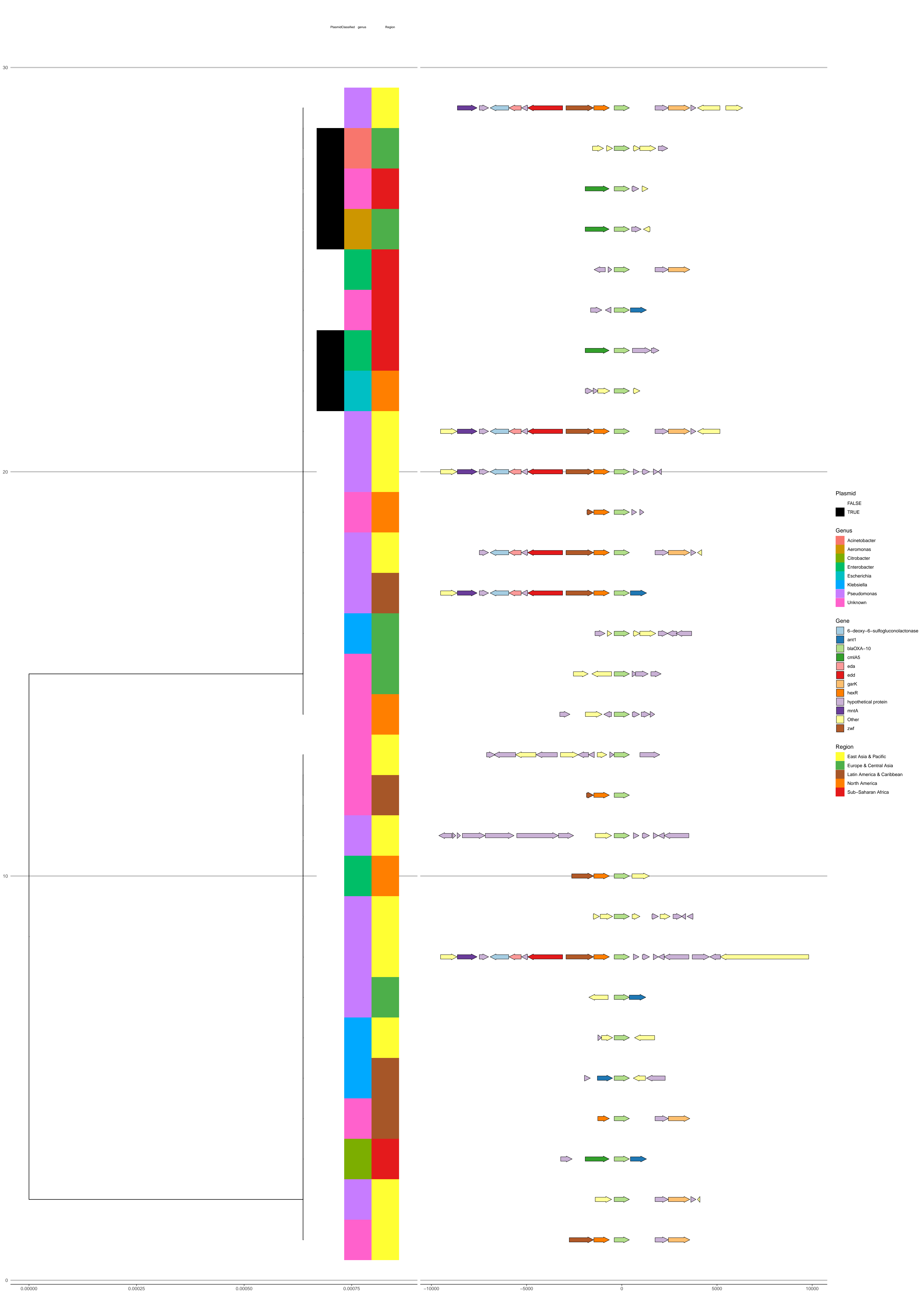

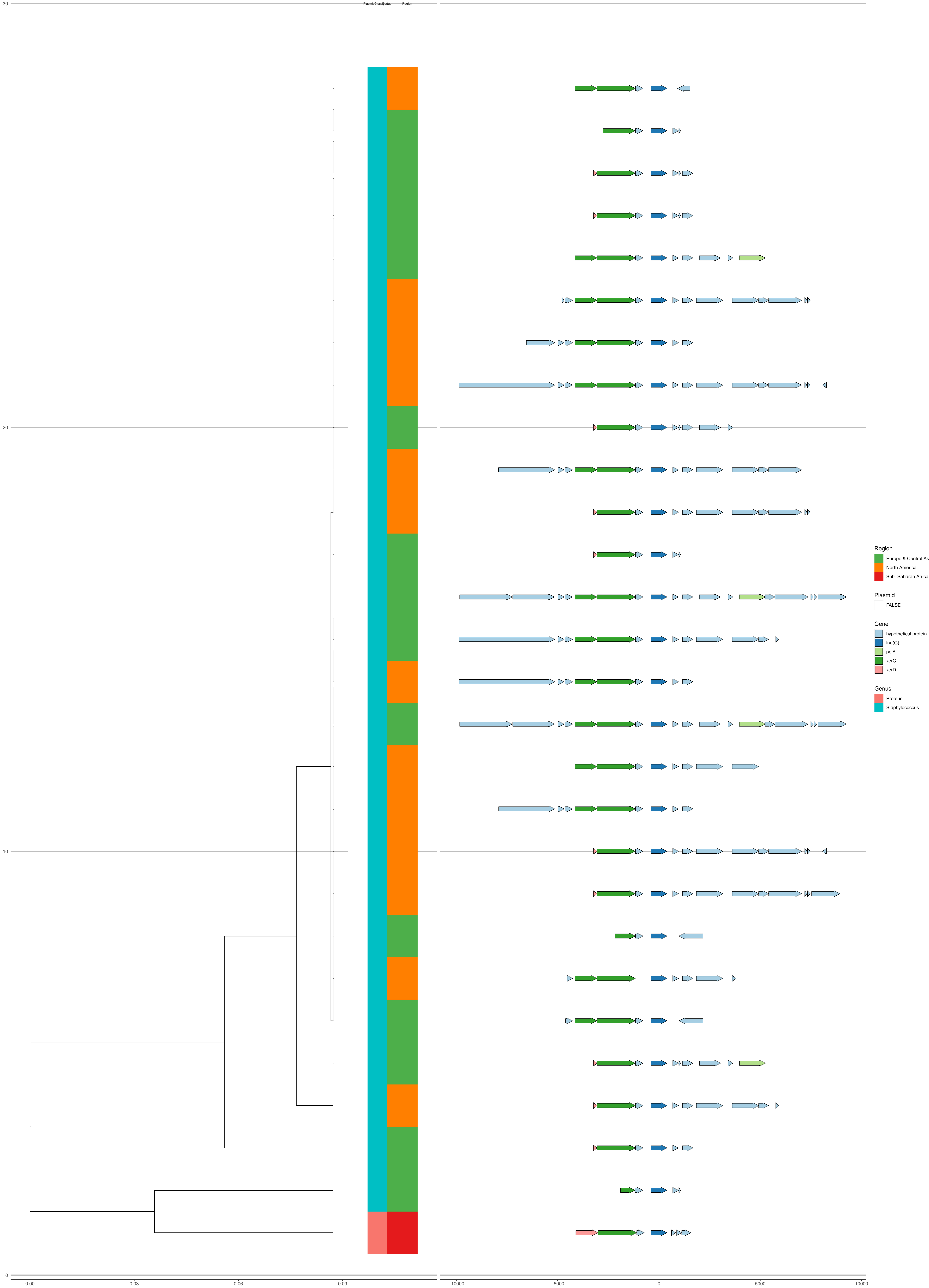

Supplement: Supplementary file 4 — Supplementary Dataset 3, Supplementary Dataset 4, Supplementary Dataset 5, Supplementary Dataset 6 [file 41467_2022_34312_MOESM4_ESM.zip › Supplementary Data 4 - ARG Gene Cluster Synteny.pdf]

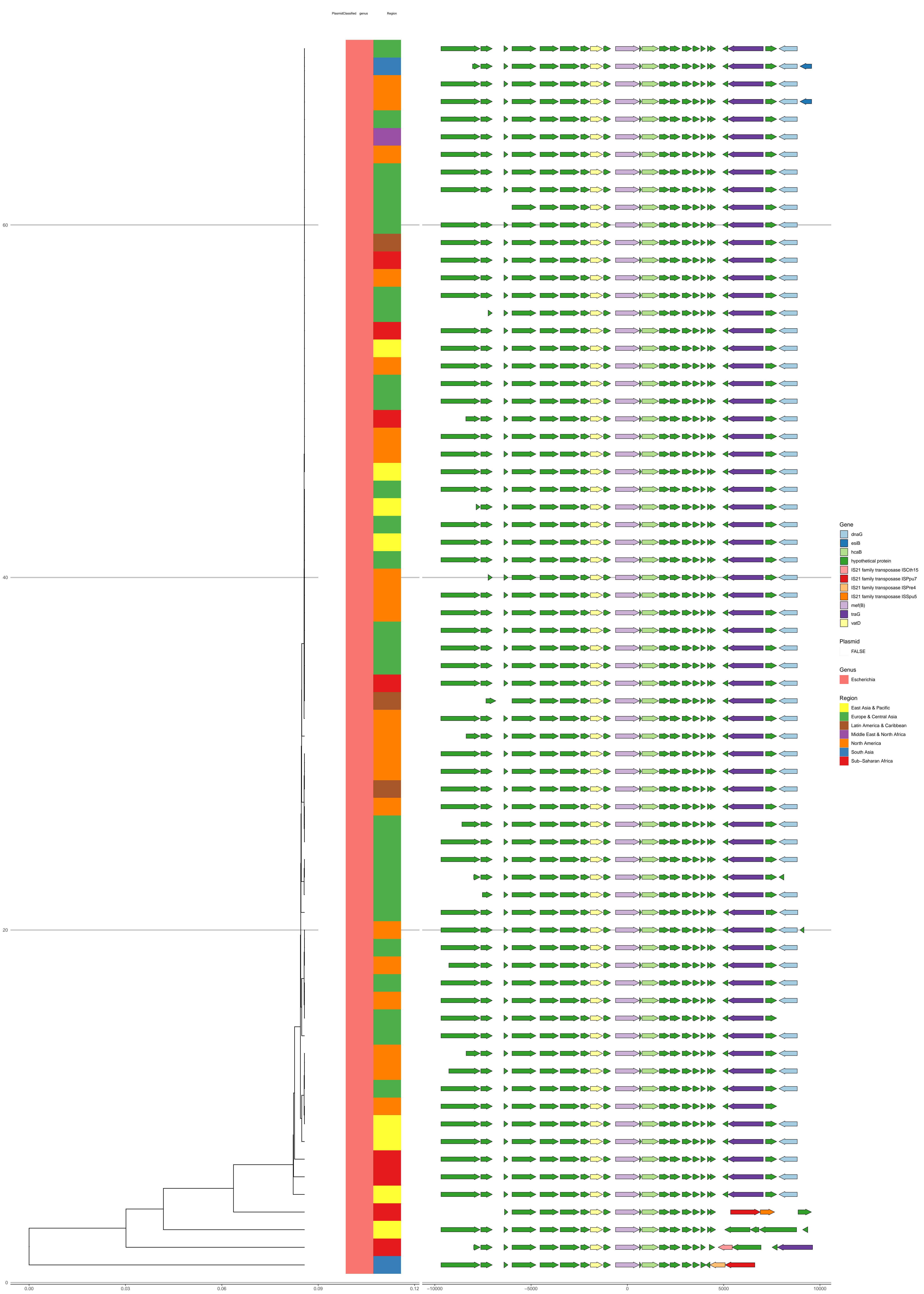

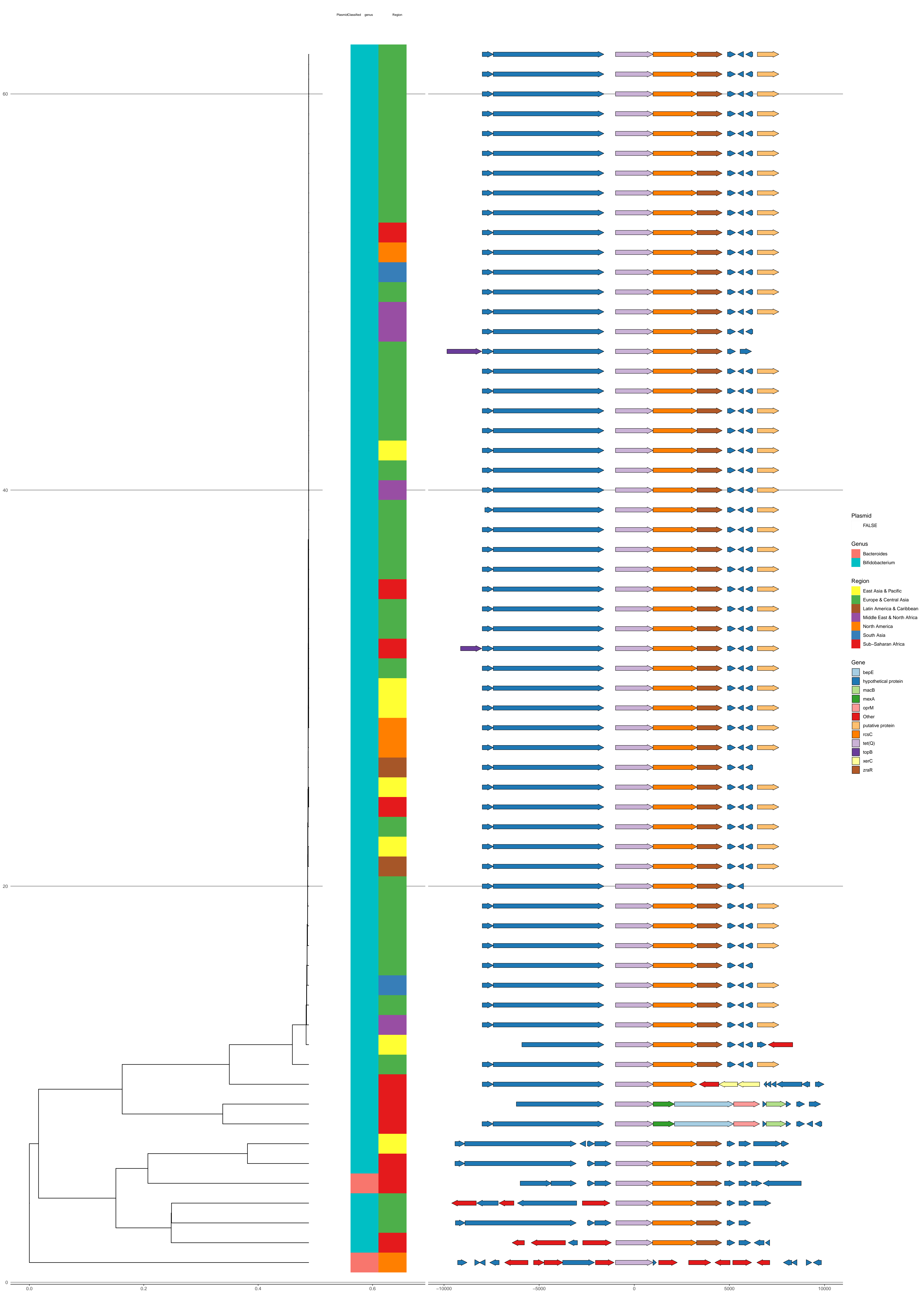

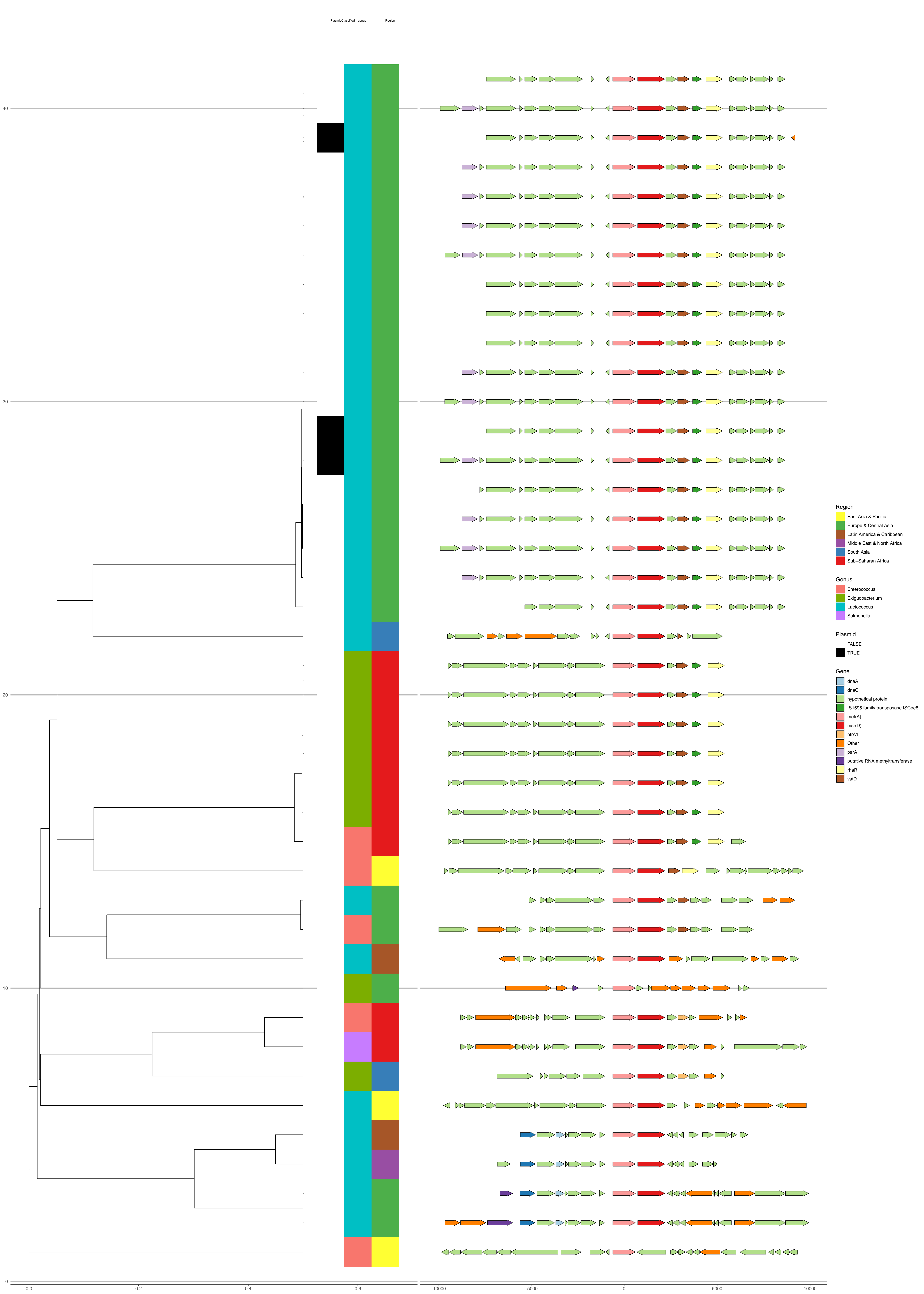

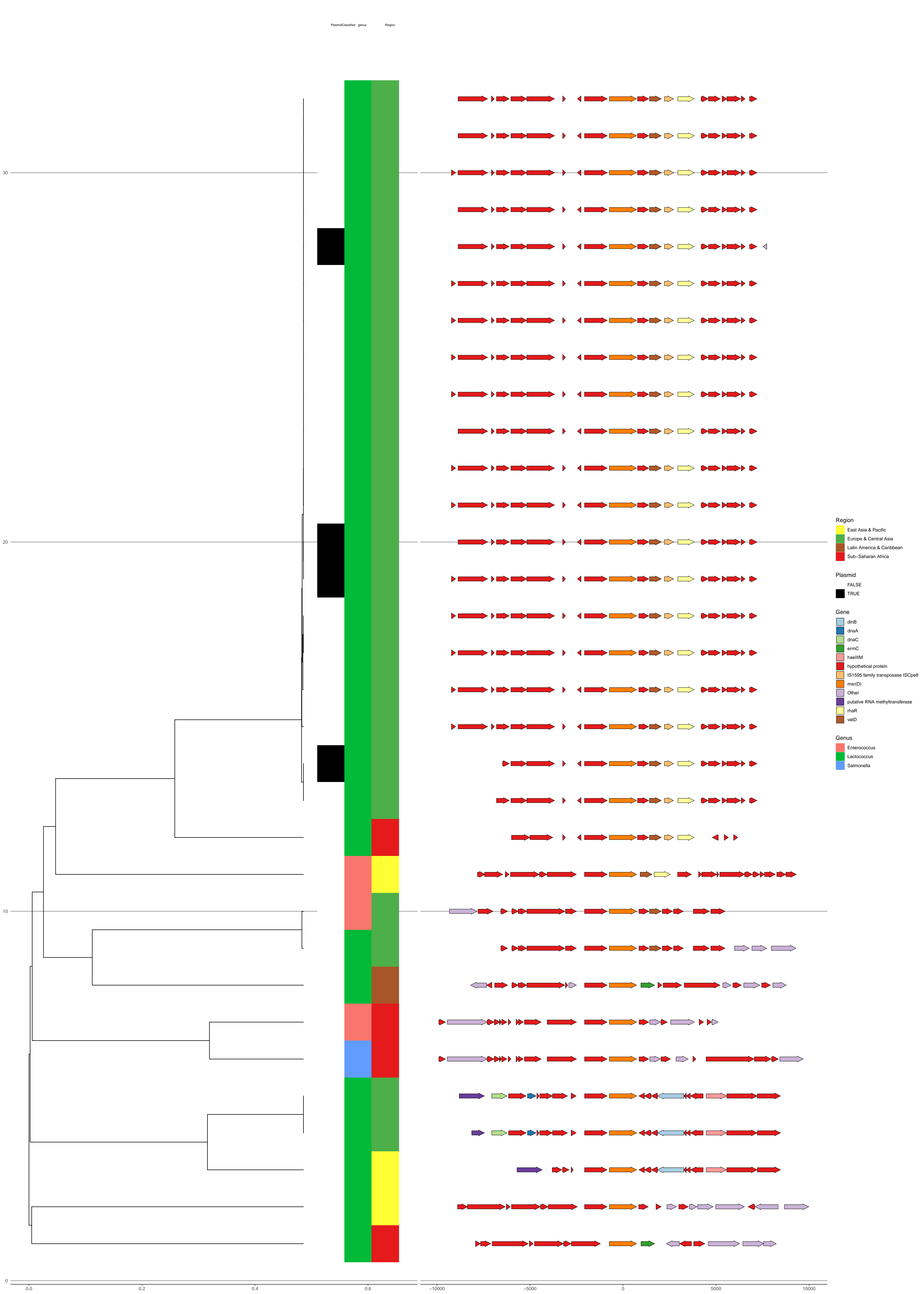

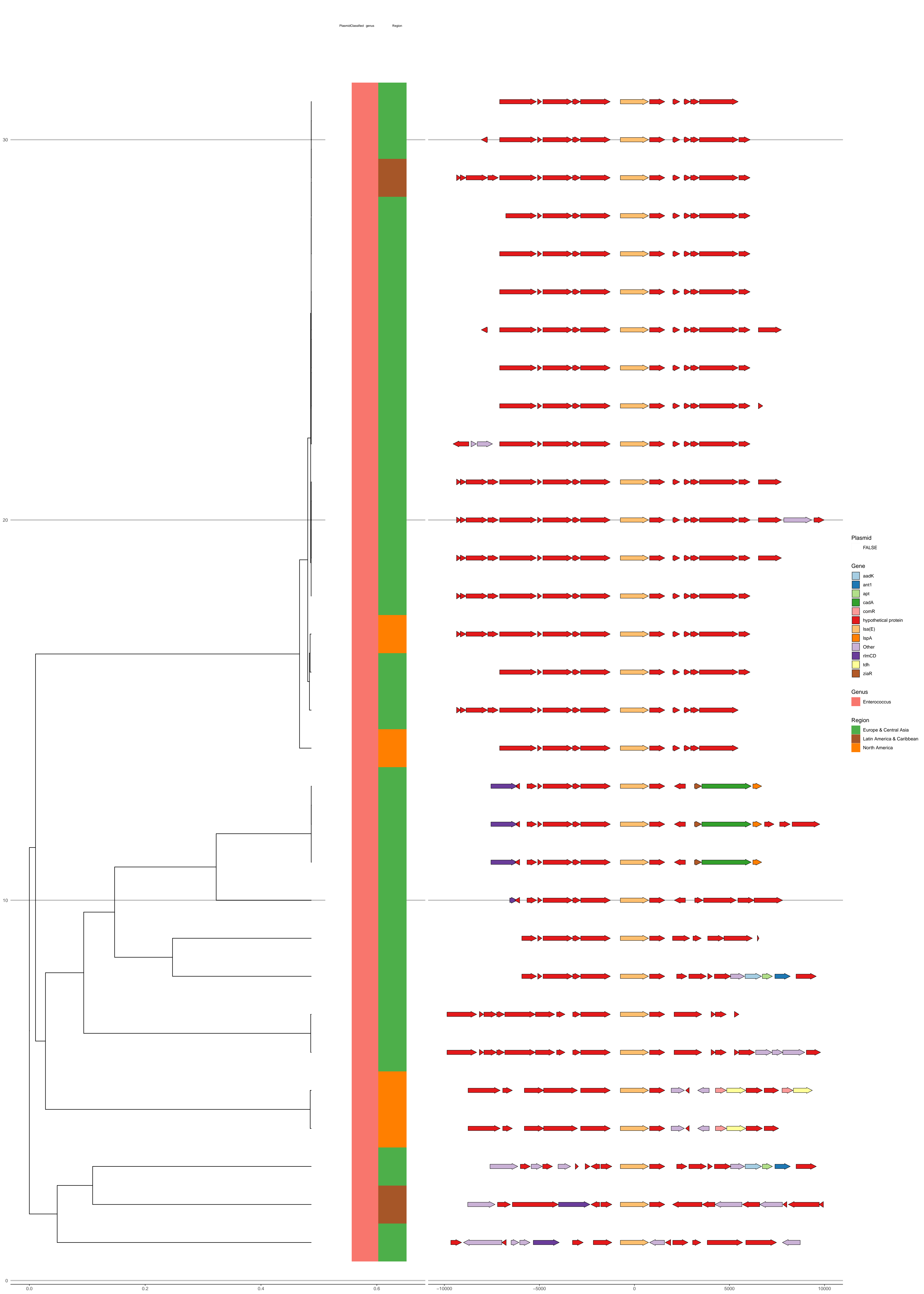

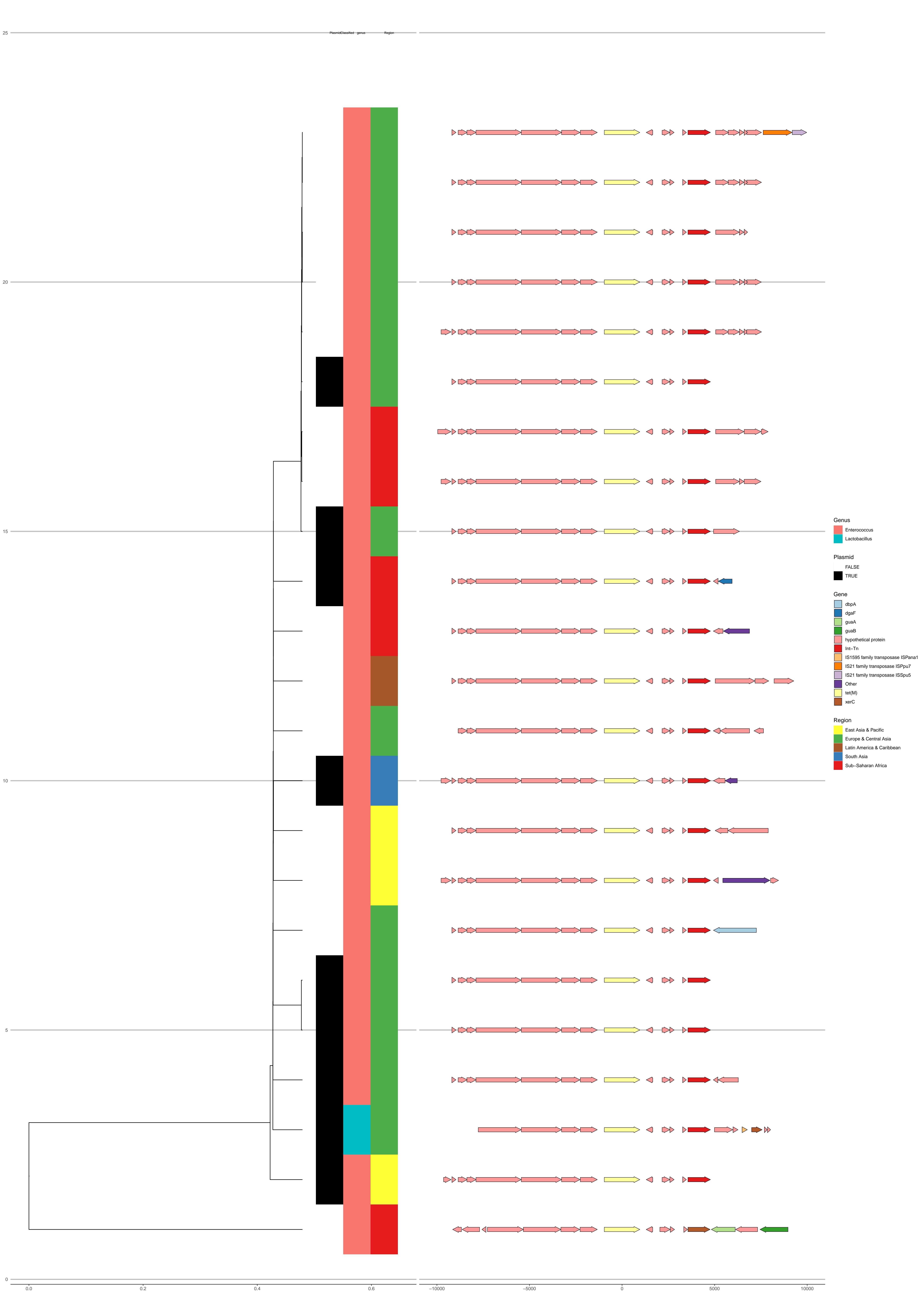

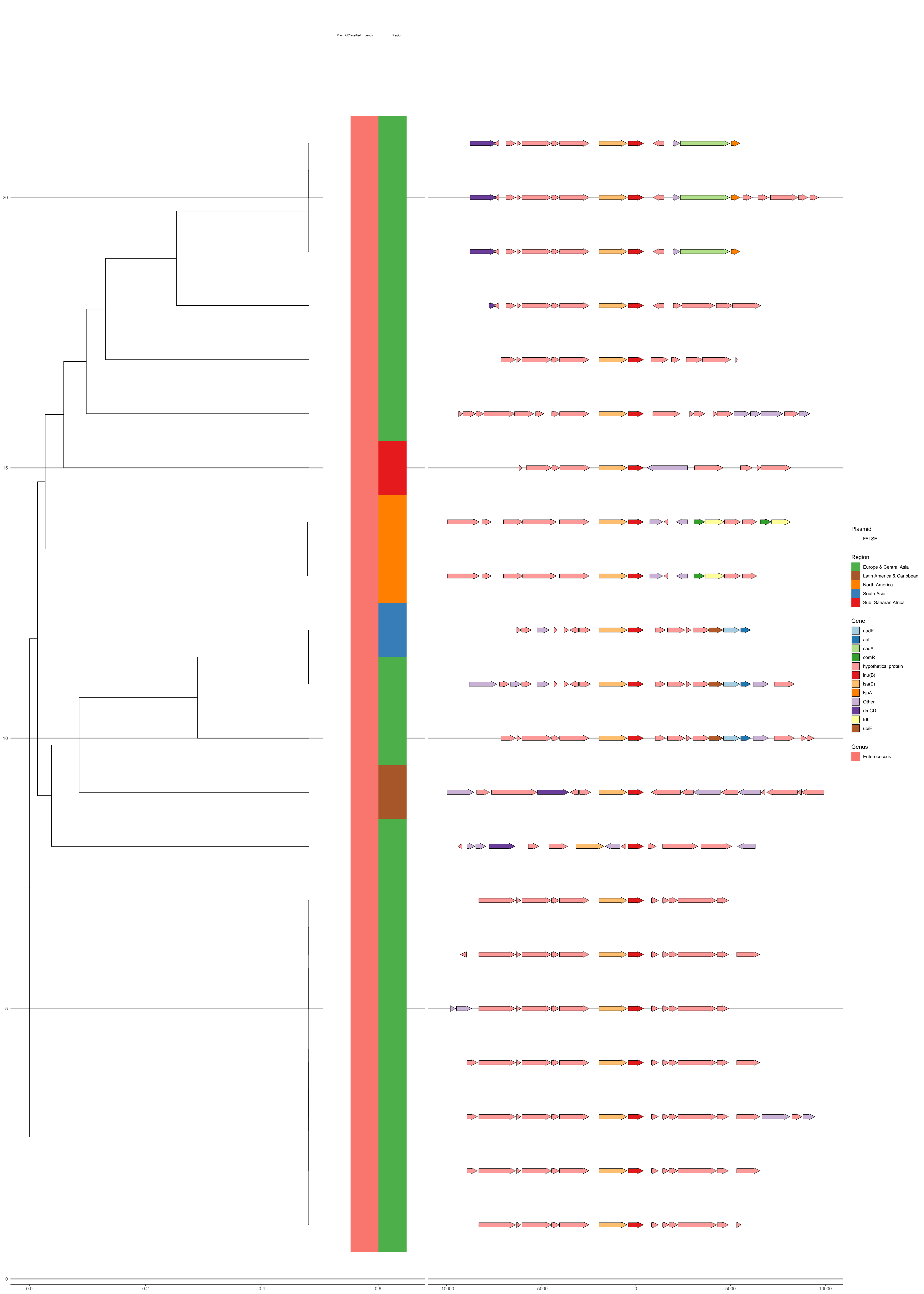

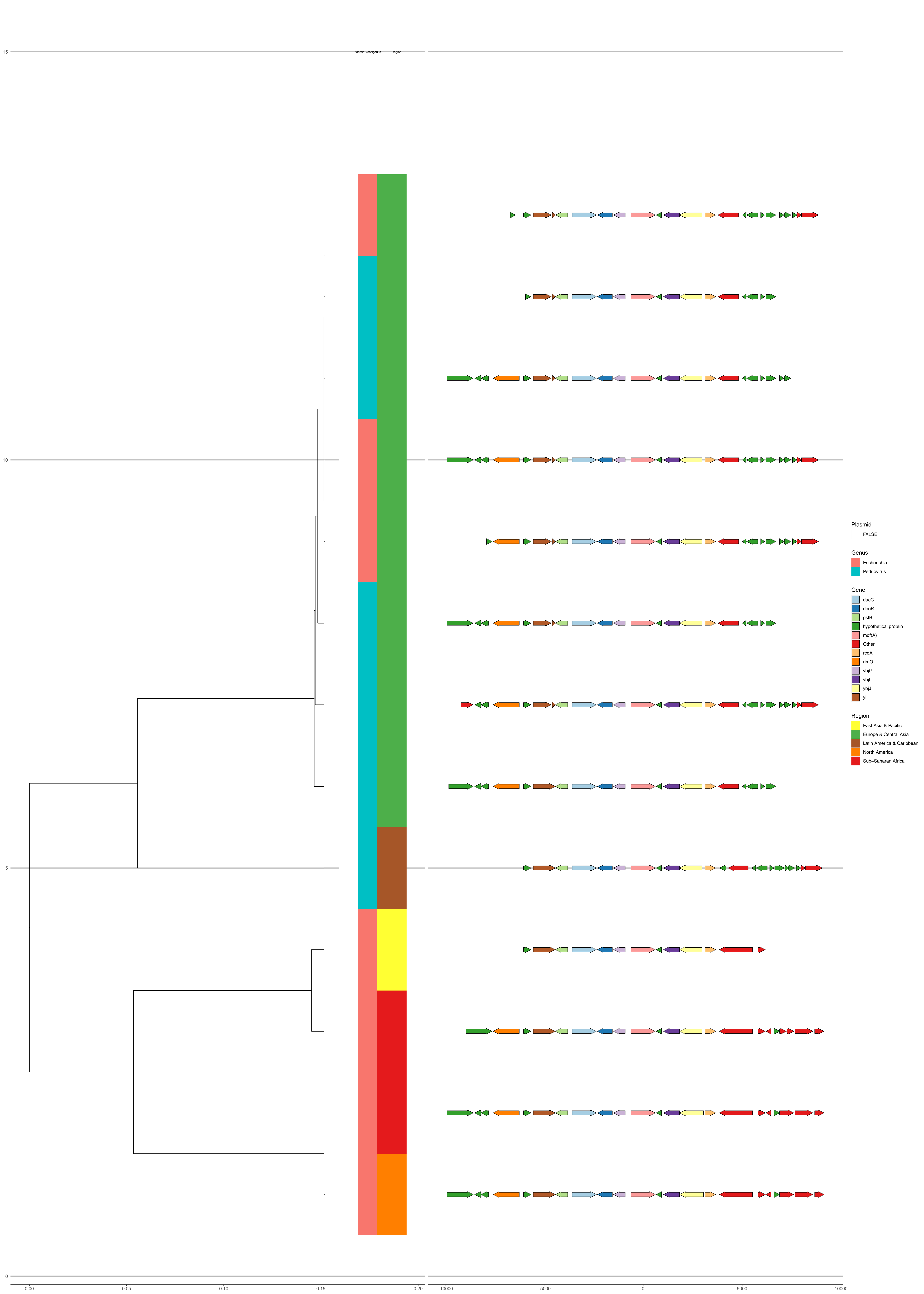

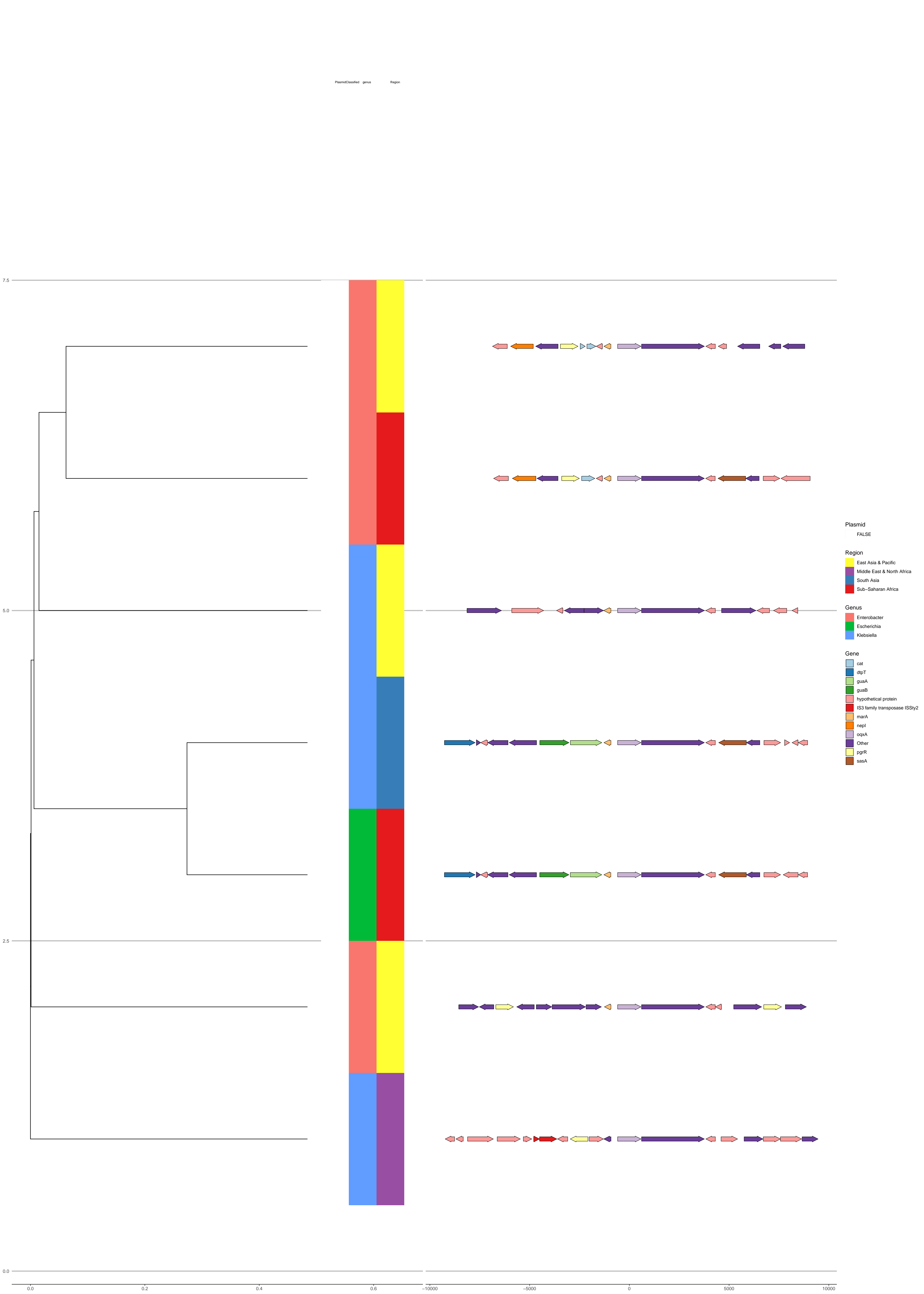

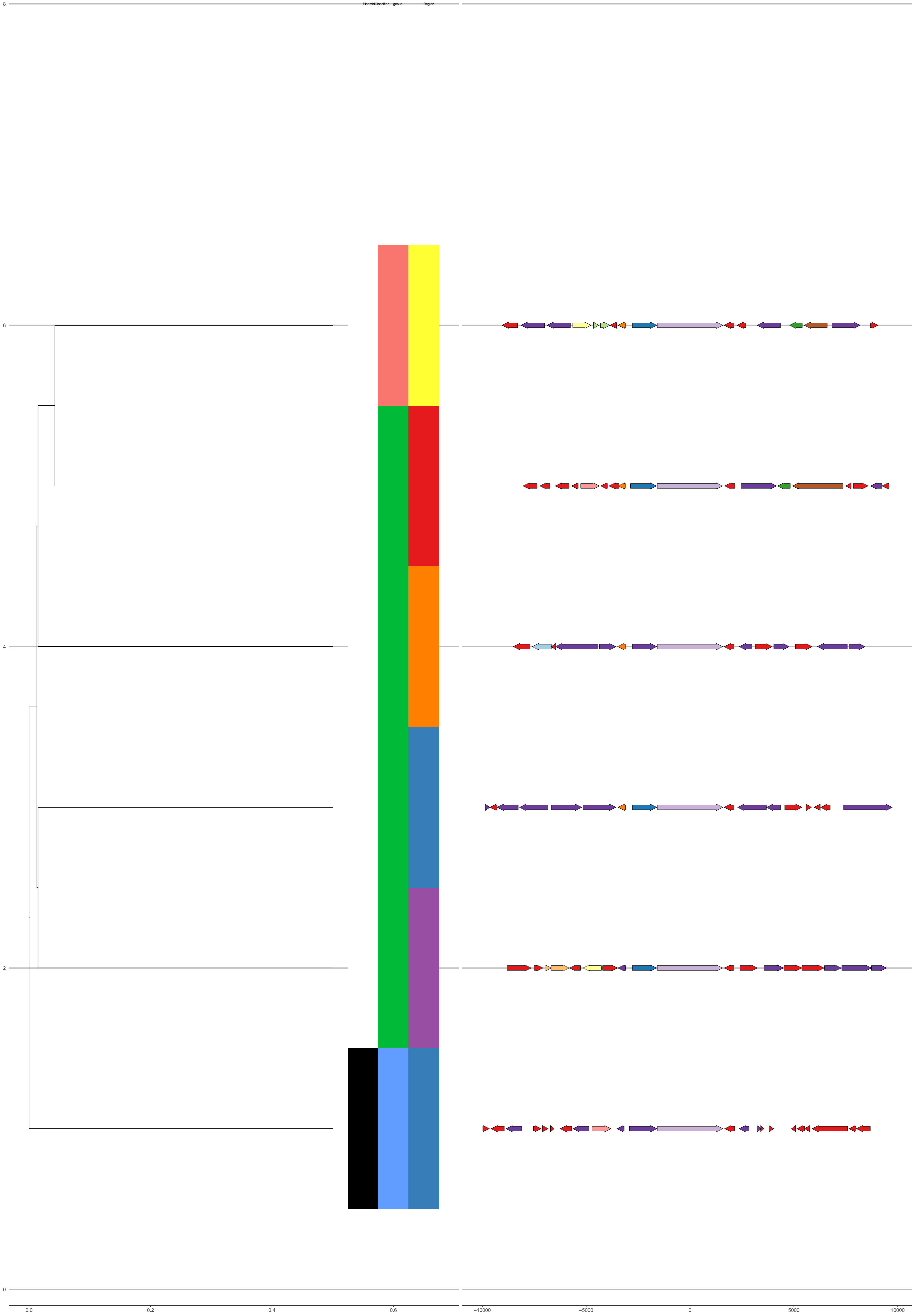

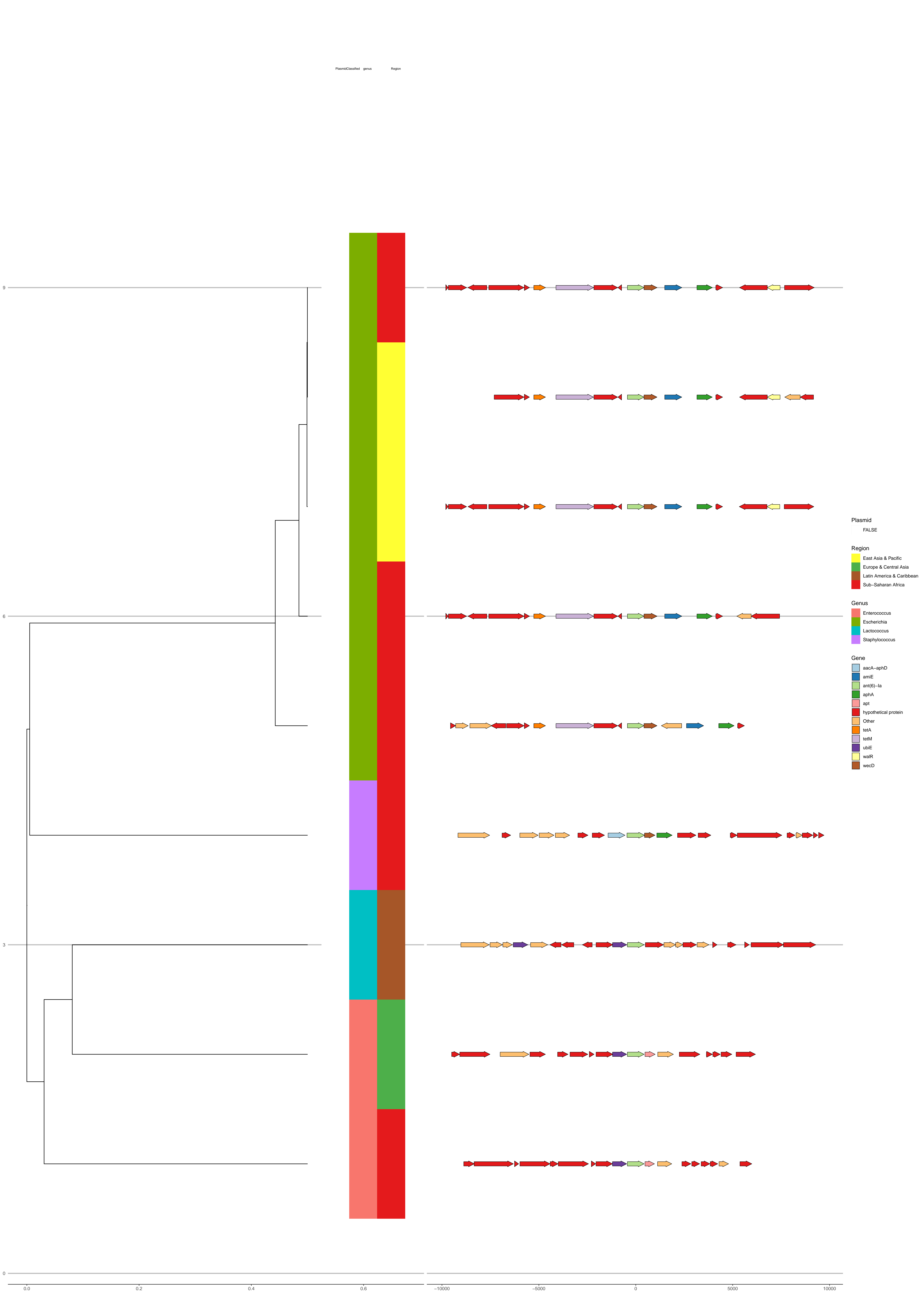

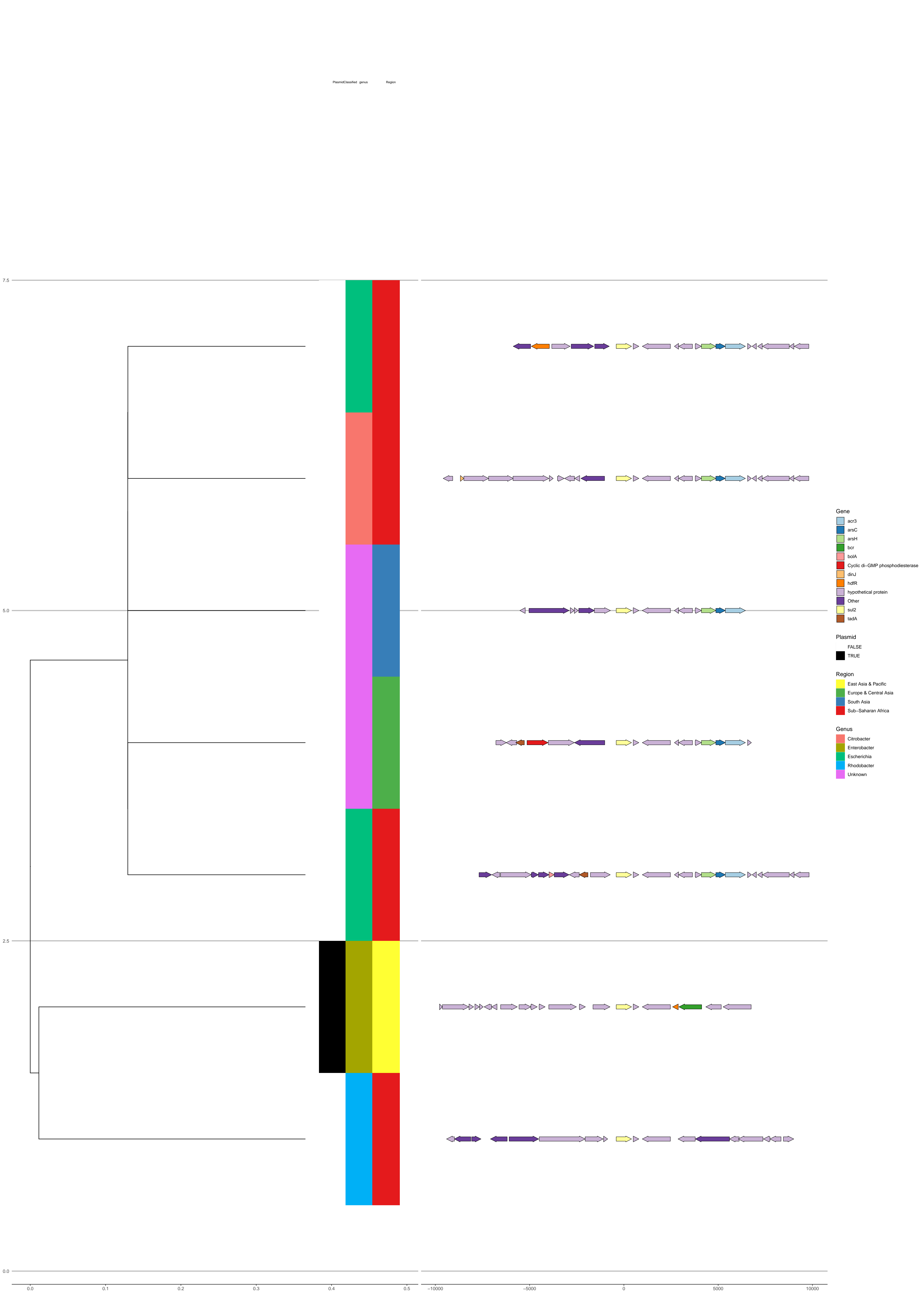

Supplement: Supplementary file 4 — Supplementary Dataset 3, Supplementary Dataset 4, Supplementary Dataset 5, Supplementary Dataset 6 [file 41467_2022_34312_MOESM4_ESM.zip › Supplementary Data 5 - ARG Flank 5Kb Cluster Synteny.pdf]

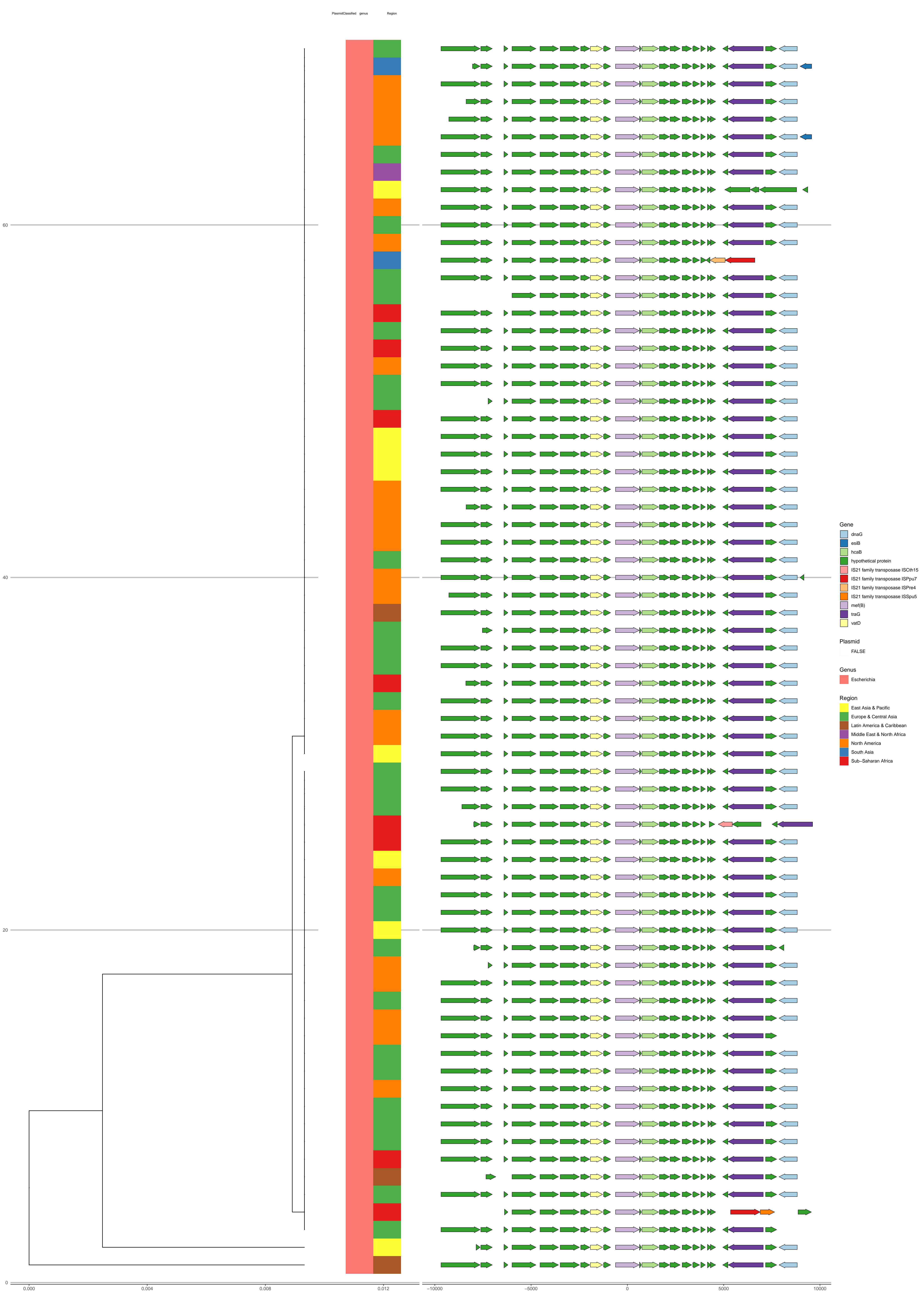

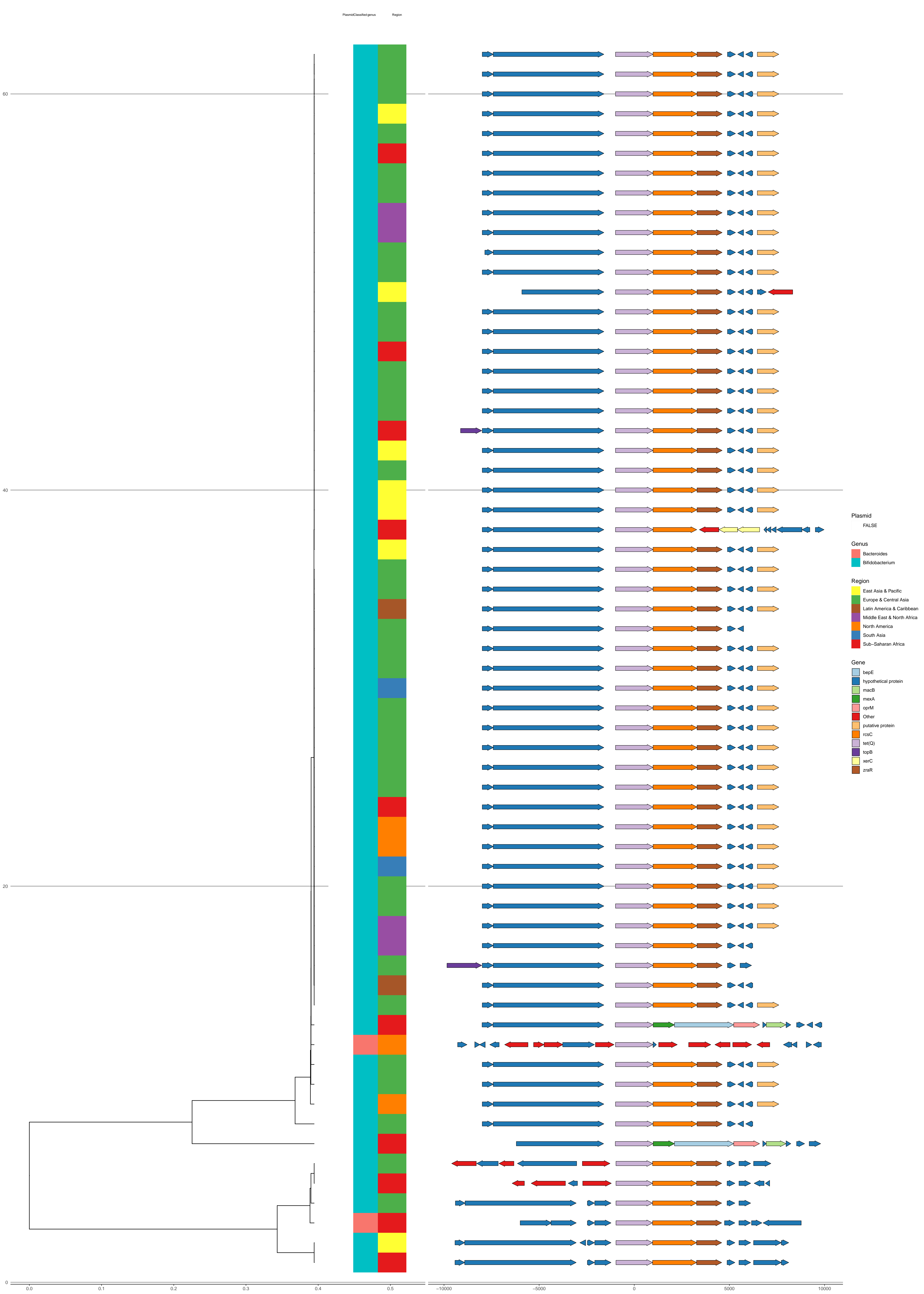

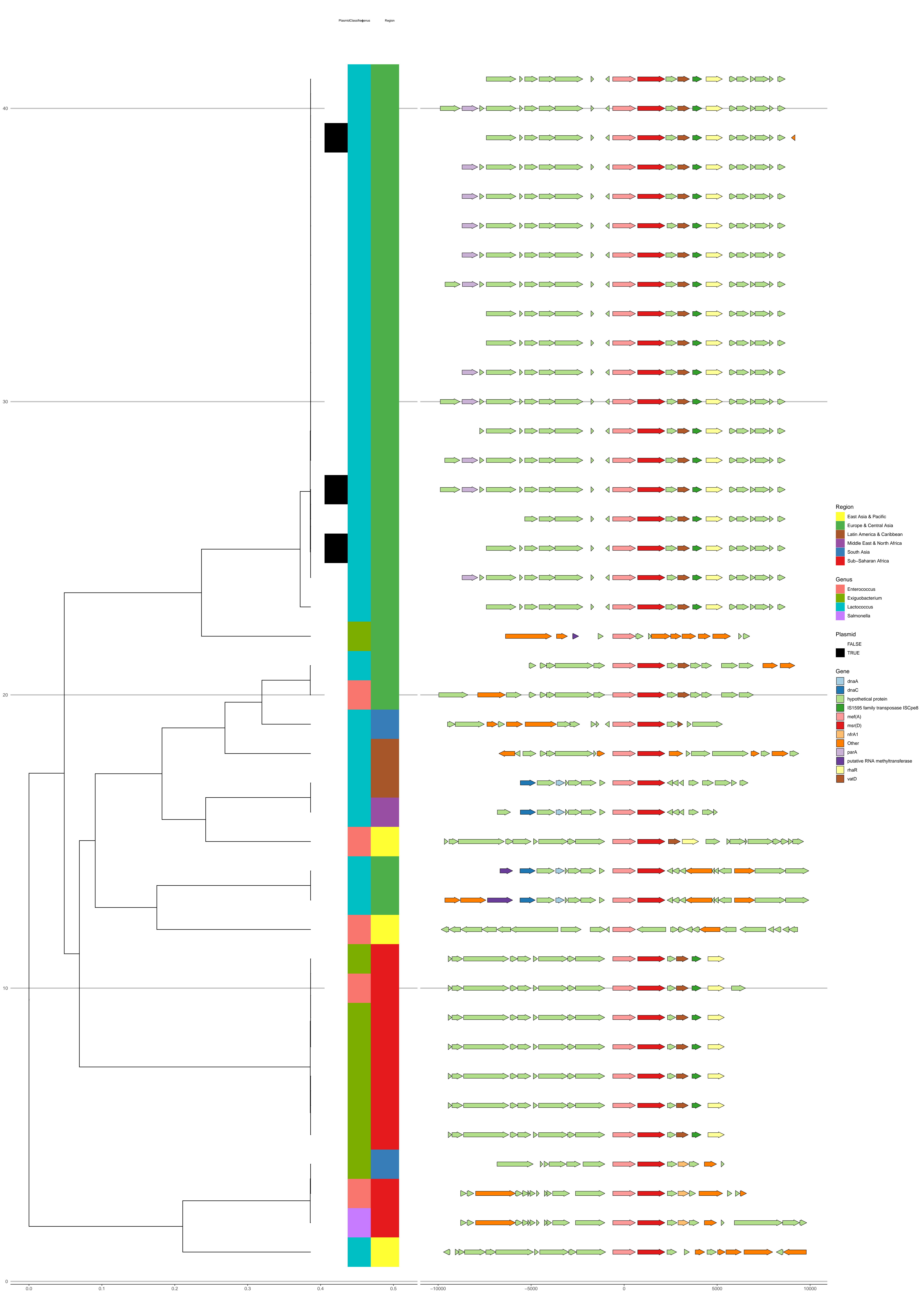

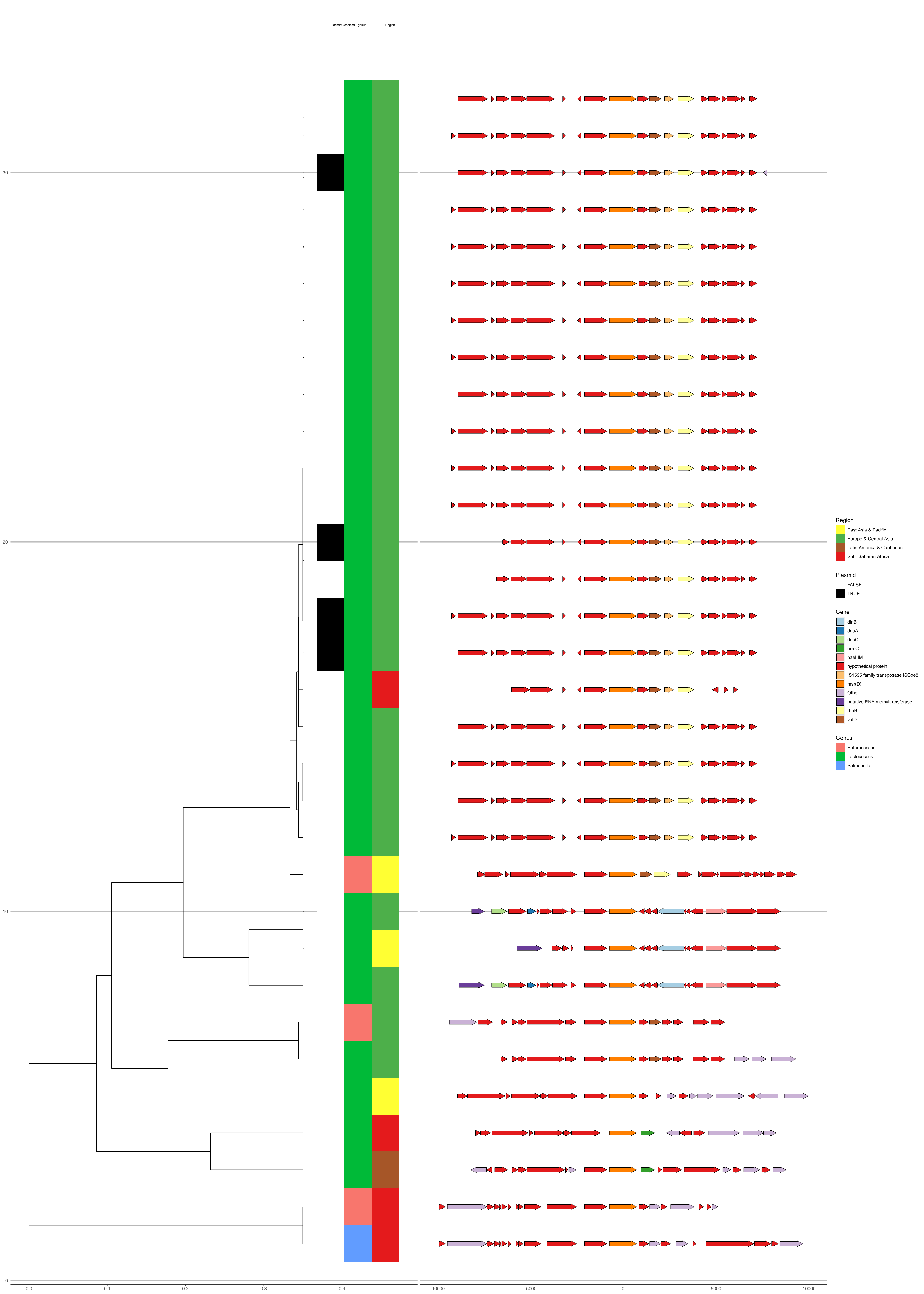

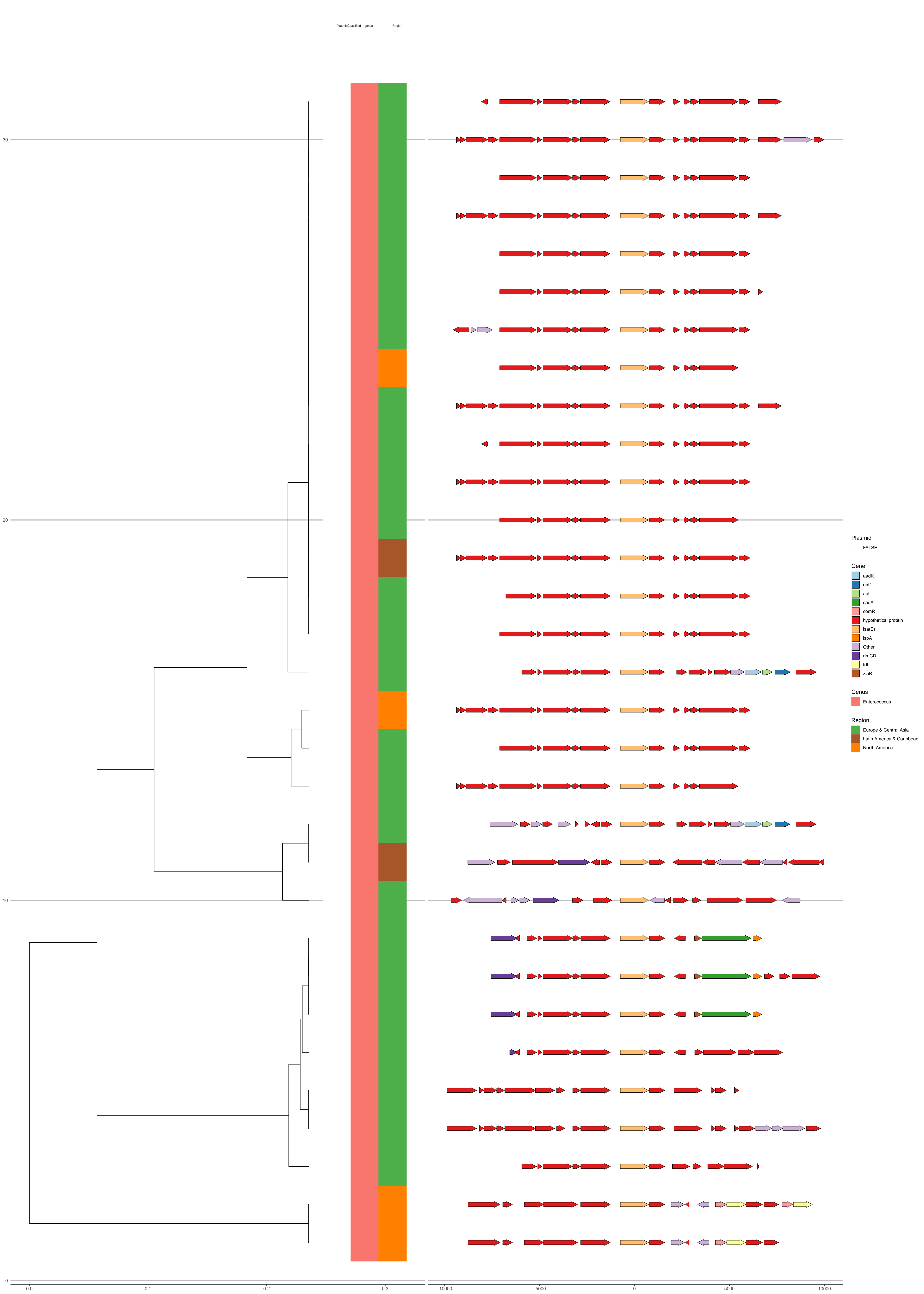

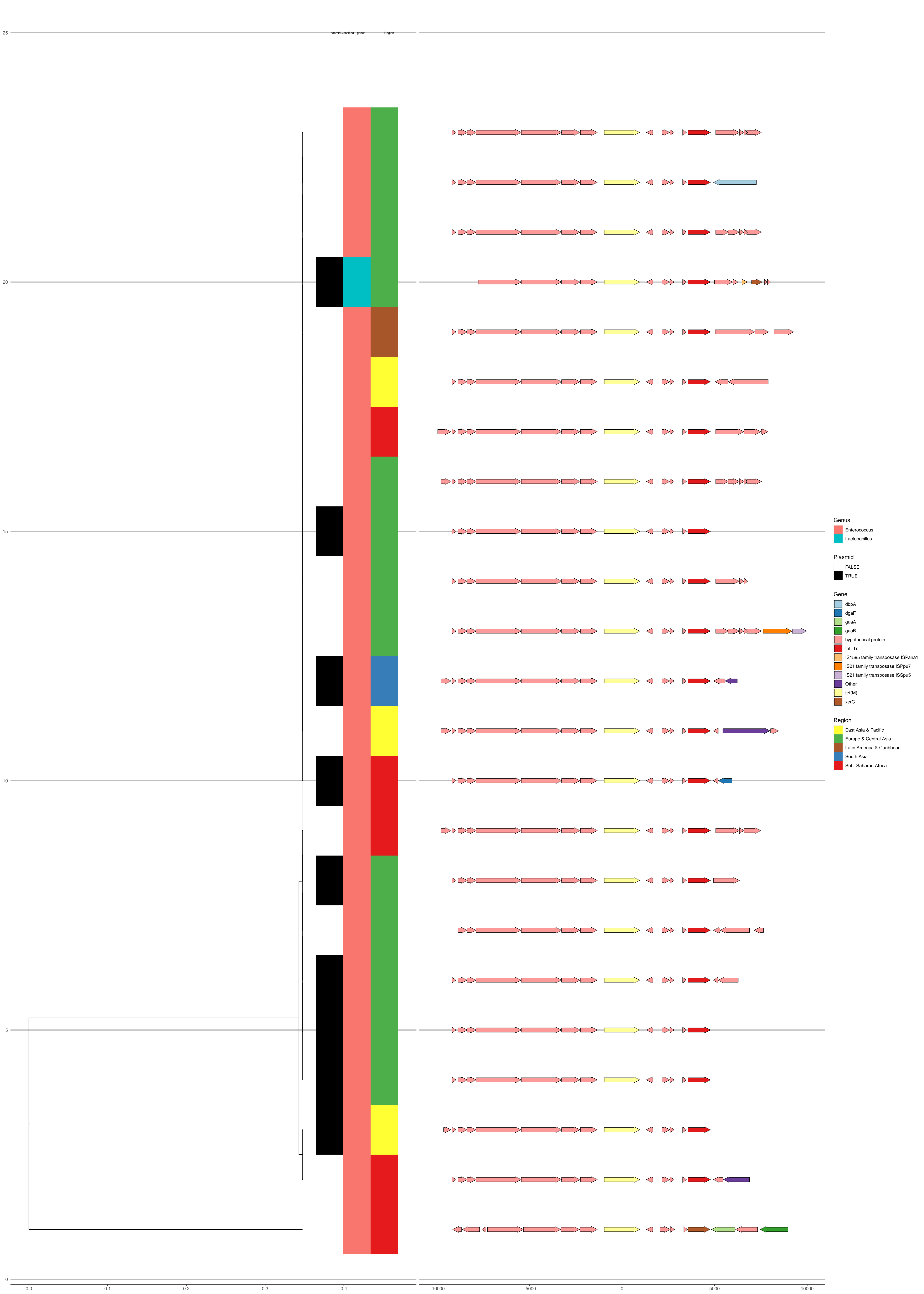

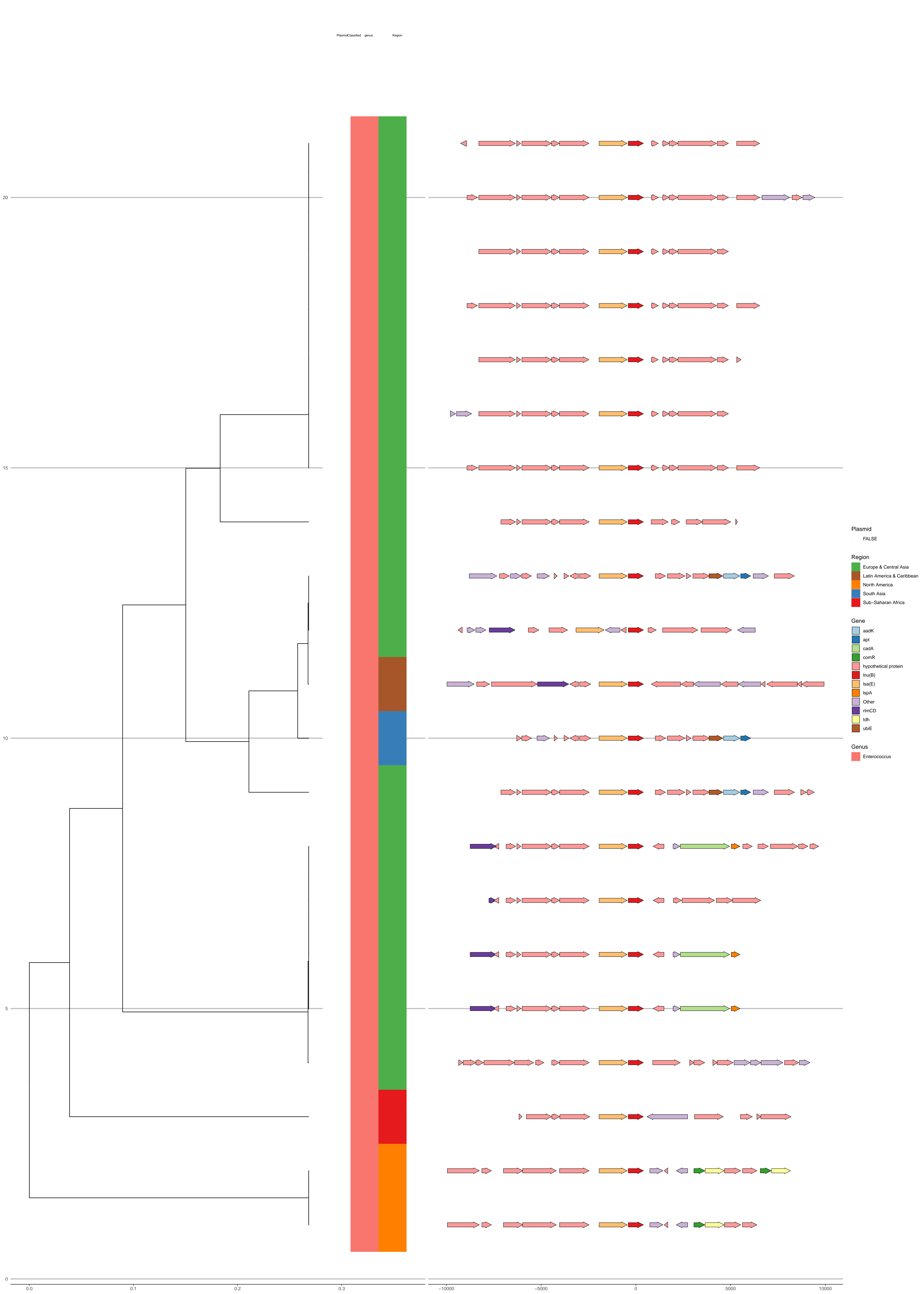

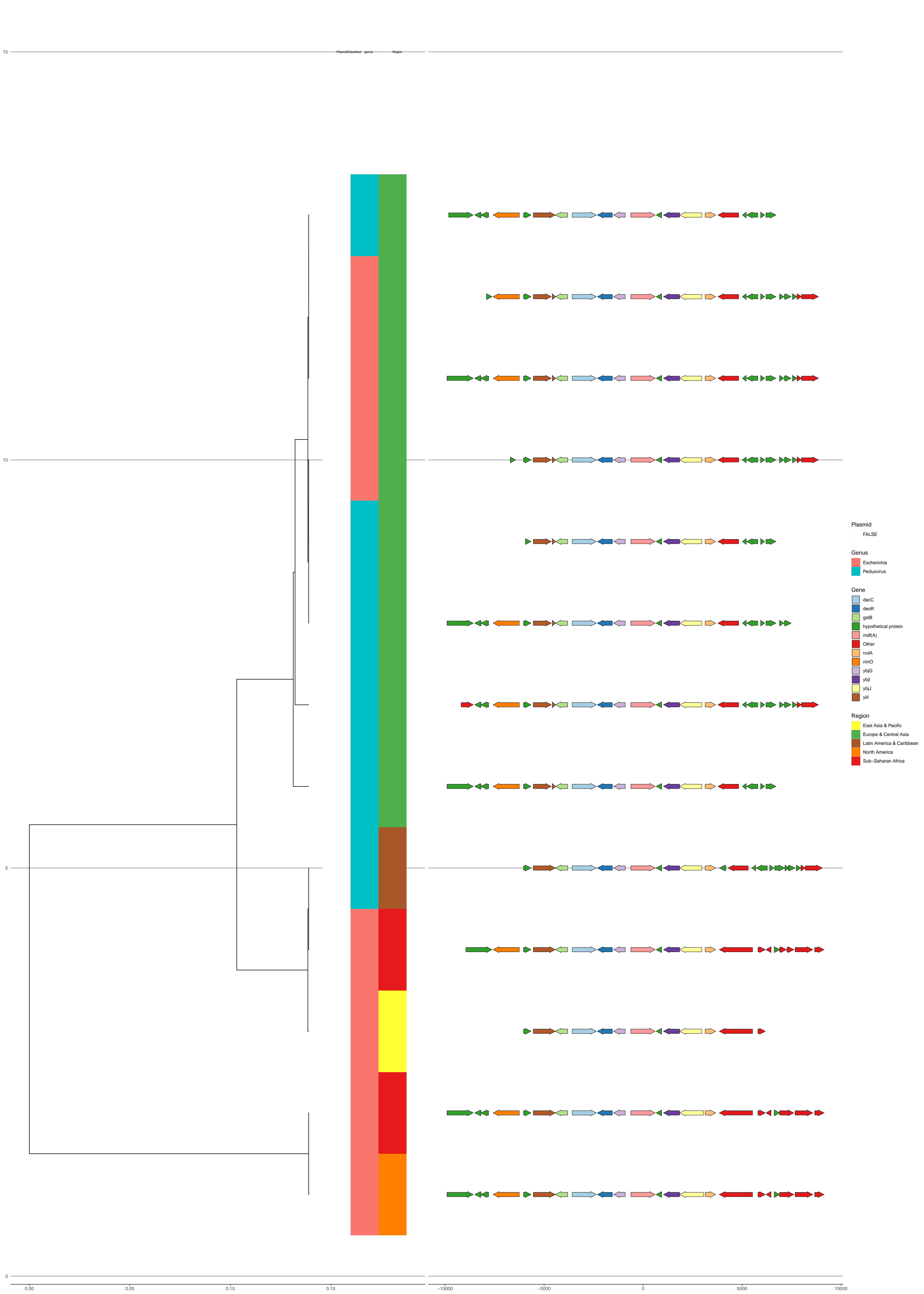

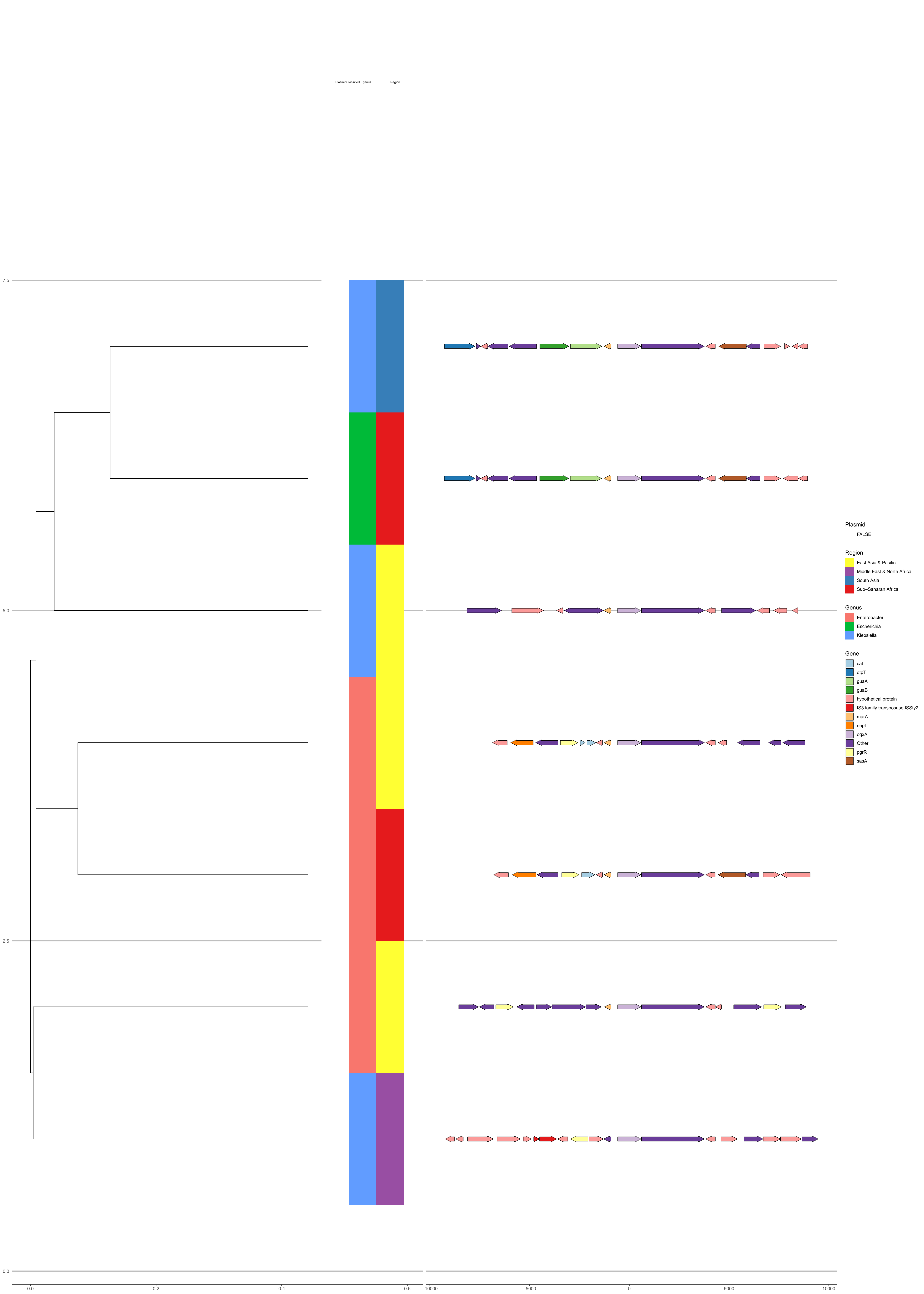

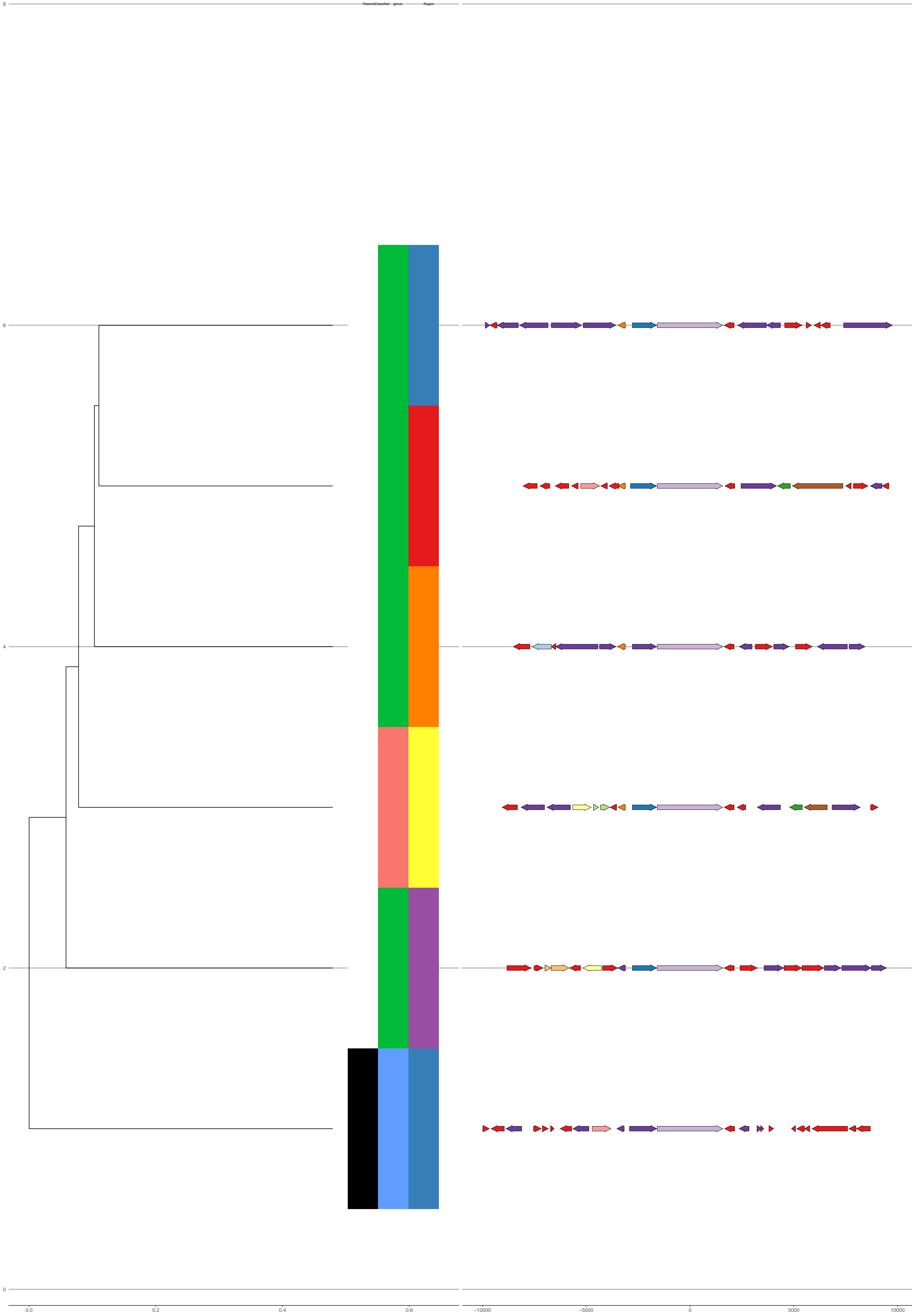

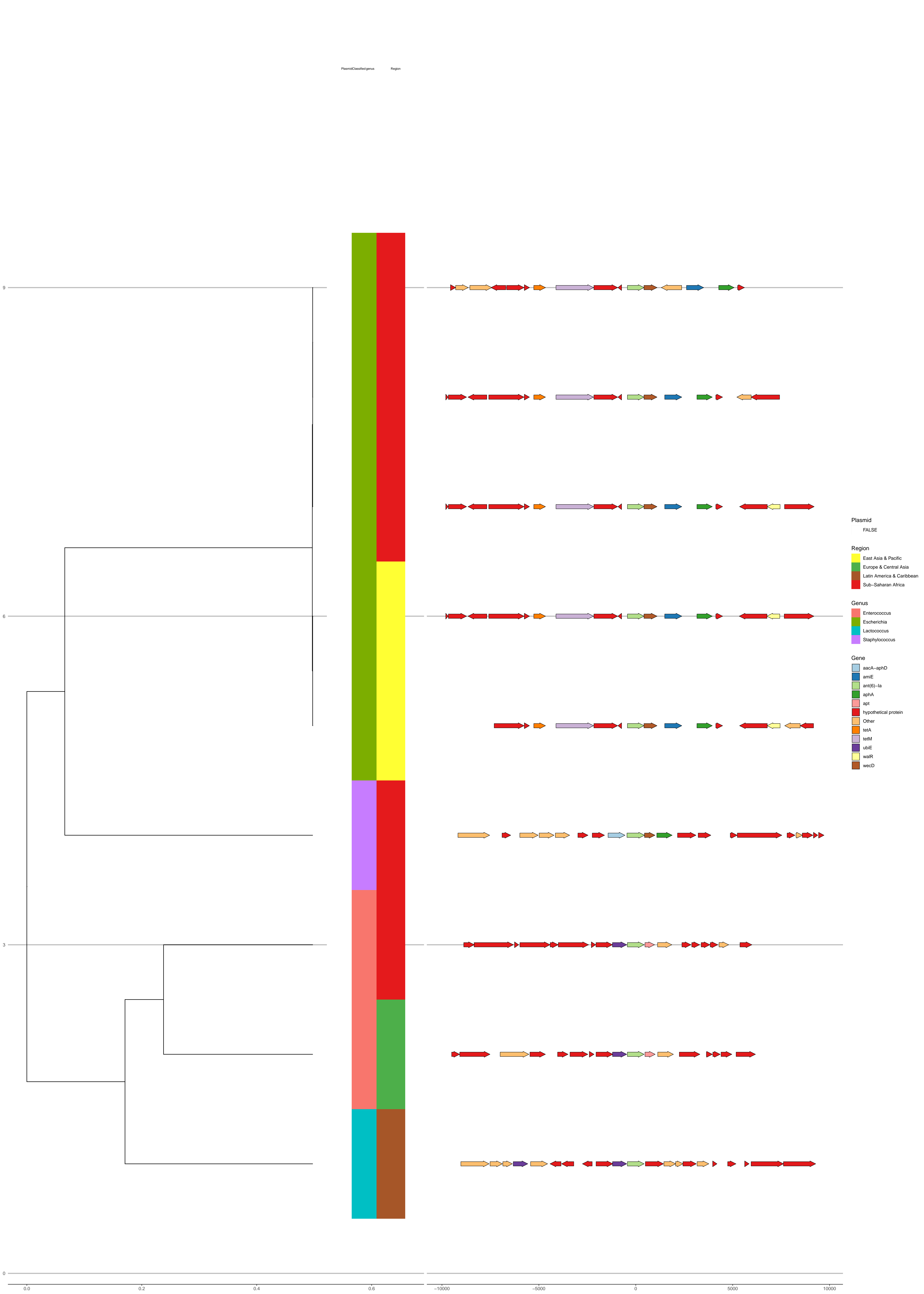

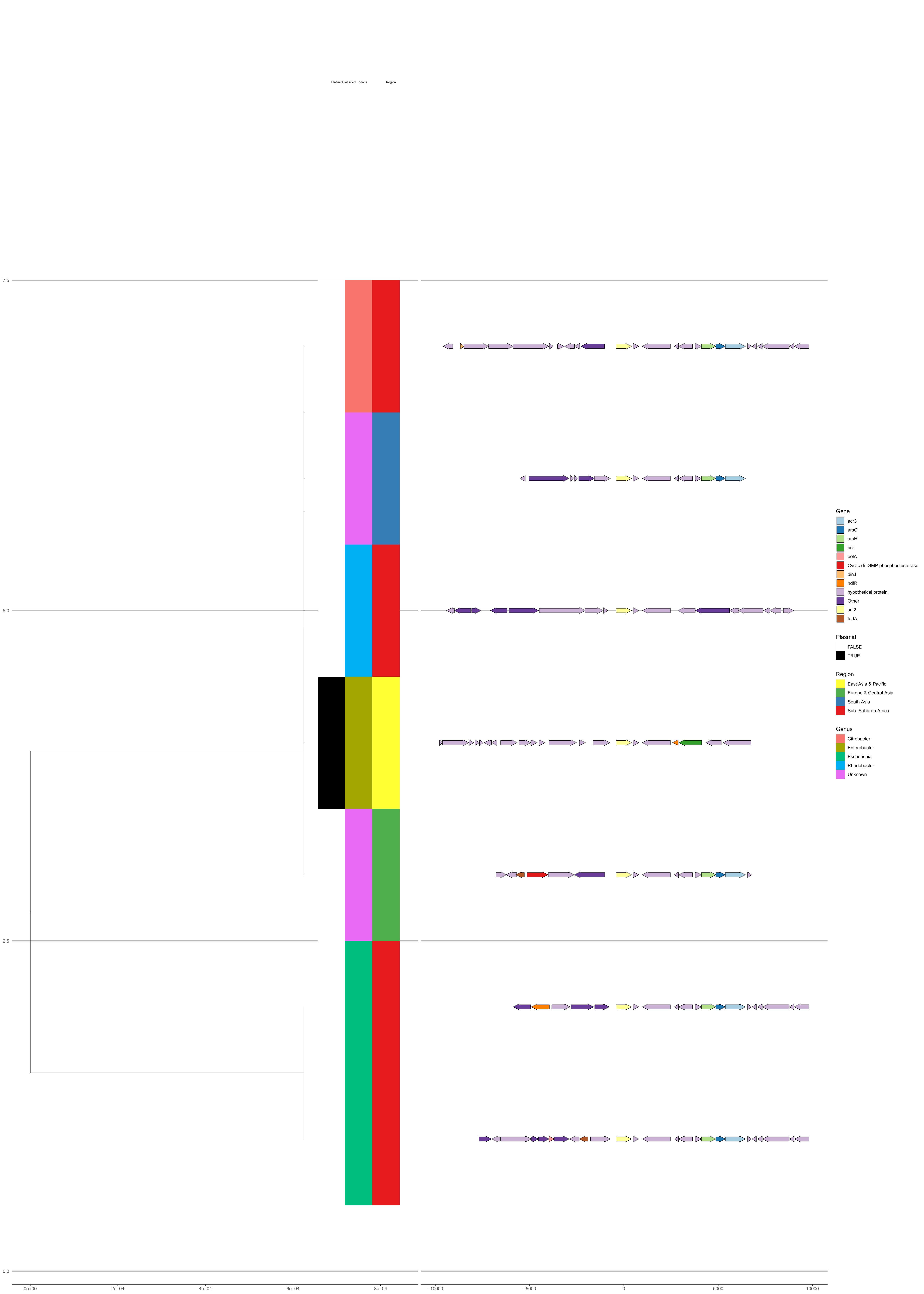

Supplement: Supplementary file 4 — Supplementary Dataset 3, Supplementary Dataset 4, Supplementary Dataset 5, Supplementary Dataset 6 [file 41467_2022_34312_MOESM4_ESM.zip › Supplementary Data 6 - ARG Gene 5Kb Cluster Synteny.pdf]
