# Supplementary material for: Genomic analysis of sewage from 101 countries reveals global landscape of antimicrobial resistance
Source: Nat Commun. 2022 Dec 1;13:7251. doi: 10.1038/s41467-022-34312-7 (PMC9715550; doi:10.1038/s41467-022-34312-7)
Supplement: Supplementary file 5 — Reporting Summary [file 41467_2022_34312_MOESM5_ESM.pdf]

nature portfolio

Corresponding author(s): Patrick Munk

Last updated by author(s): Oct 6, 2022

Reporting Summary

Nature Portfolio wishes to improve the reproducibility of the work that we publish. This form provides structure for consistency and transparency in reporting. For further information on Nature Portfolio policies, see our Editorial Policies and the Editorial Policy Checklist.

Statistics

For all statistical analyses, confirm that the following items are present in the figure legend, table legend, main text, or Methods section.

n/a	Confirmed	
☐	☒	The exact sample size (*n*) for each experimental group/condition, given as a discrete number and unit of measurement
☐	☒	A statement on whether measurements were taken from distinct samples or whether the same sample was measured repeatedly
☐	☒	The statistical test(s) used AND whether they are one- or two-sided<br>*Only common tests should be described solely by name; describe more complex techniques in the Methods section.*
☐	☒	A description of all covariates tested
☐	☒	A description of any assumptions or corrections, such as tests of normality and adjustment for multiple comparisons
☐	☒	A full description of the statistical parameters including central tendency (e.g. means) or other basic estimates (e.g. regression coefficient) AND variation (e.g. standard deviation) or associated estimates of uncertainty (e.g. confidence intervals)
☐	☒	For null hypothesis testing, the test statistic (e.g. *F*, *t*, *r*) with confidence intervals, effect sizes, degrees of freedom and *P* value noted<br>*Give P values as exact values whenever suitable.*
☒	☐	For Bayesian analysis, information on the choice of priors and Markov chain Monte Carlo settings
☒	☐	For hierarchical and complex designs, identification of the appropriate level for tests and full reporting of outcomes
☐	☒	Estimates of effect sizes (e.g. Cohen's *d*, Pearson's *r*), indicating how they were calculated

Our web collection on statistics for biologists contains articles on many of the points above.

Software and code

Policy information about availability of computer code

Data collection	No software was used
Data analysis	R 4.1.1<br>Python 3.7<br>ResFinder 3<br>BBmap 36.49<br>BEDTools 2.28.0<br>KMA 1.2.17a<br>KMA 1.3.3<br>Spades 3.13.0<br>Kraken 2.0.8<br>USEARCH 11.0.7 (free 32bit version)<br>vegan R package 2.6.2<br>ggraph R package 2.0.5<br>dendextend R package 1.14.0<br>PPR-Meta 1.1<br>PyCoDa (commit a7b3f62). https://bitbucket.org/genomicepidemiology/pycoda

For manuscripts utilizing custom algorithms or software that are central to the research but not yet described in published literature, software must be made available to editors and reviewers. We strongly encourage code deposition in a community repository (e.g. GitHub). See the Nature Portfolio guidelines for submitting code & software for further information.

Data

Policy information about availability of data

All manuscripts must include a data availability statement. This statement should provide the following information, where applicable:

- Accession codes, unique identifiers, or web links for publicly available datasets
- A description of any restrictions on data availability
- For clinical datasets or third party data, please ensure that the statement adheres to our policy

Data availability

The raw sequencing data (FASTQ) generated in this study has been deposited in the European Nucleotide Archive and can be accessed without restrictions. The data from major sampling rounds ha have the following project accession numbers: PRJEB40798 [ebi.ac.uk/ena/browser/view/PRJEB40798], PRJEB40816 [ebi.ac.uk/ena/browser/view/PRJEB40816], PRJEB40815 [ebi.ac.uk/ena/browser/view/PRJEB40815] and PRJEB27621 [ebi.ac.uk/ena/browser/view/PRJEB27621]. Sequencing data from the longitudinal city sampling sites are deposited under PRJEB51229 [ebi.ac.uk/ena/browser/view/PRJEB51229], while the included datasets from the previous study are deposited under ERP015409 [ebi.ac.uk/ena/browser/view/ERP015409]. See Supplementary Data 1 for exact sample, experiment and run accessions. Source data are provided with this paper.

This study also utilized the publicly available databases of ResFinder [https://bitbucket.org/genomicepidemiology/resfinder_db], PLSDB [https://ccb-microbe.cs.uni-saarland.de/plsdb/plasmids/download/], Silva [https://www.arb-silva.de/download/arb-files/] and Kraken 2 [https://benlangmead.github.io/aws-indexes/k2].

Field-specific reporting

Please select the one below that is the best fit for your research. If you are not sure, read the appropriate sections before making your selection.

☐ Life sciences ☐ Behavioural & social sciences ☒ Ecological, evolutionary & environmental sciences

For a reference copy of the document with all sections, see nature.com/documents/nr-reporting-summary-flat.pdf

Ecological, evolutionary & environmental sciences study design

All studies must disclose on these points even when the disclosure is negative.

Study description	The study is an observational study of genomic material in untreated sewage samples, with a focus on antimicrobial resistance genes. Worldwide participants were invited to participate. Participants were instructed as in the pilot study (Hendriksen et al., 2019) and were sent bottles and with pre-paid shipping of the samples back Denmark for analysis.
Research sample	The research samples were untreated sewage samples from cities in 100+ countries. The DNA within each sample reflects the microbiome with all the genes present at that site and the human population that contributed to the samples through defecation. The samples are meant to capture the global differences in the human-impacted urban environments globally, but also contain environmental contribution. Untreated sewage has previously been shown to be a cost-effective and ethical way of surveying AMR globally (Hendriksen et al., 2019).
Sampling strategy	We advertised the study widely and invited everyone in and out of our network to participate samples to the study. No exact sample size calculations were performed prior to the study, but we aimed to cover as much of the world is possible. In that sense, samples were convenience samples and we could not know the global interest.<br><br>Each enrolled participant was instructed to sample ~ 1L untreated community sewage before any potential treatment plant using either sampling over 24H or an approach of 3 x 300 mL approach at least 5 min aparts. The protocol was described in Hendriksen et al. (2019). Protocol, instructions and relevant appendencies can be found in Supplementary Data 8 of that study. The pilot study found significant regional effects at all investigated levels, despite much fewer samples.
Data collection	Collaborating partners in 100+ countries were instructed in how to take local waste water samples, freeze them and ship them back to Denmark at DTUs expense. Sampling bottles etc. were shipped to partners. The individual partners at the discrete sampling sites were responsible for taking pictures, filling out the metadata and submit it to DTU through Survey Monkey, as well as shipping the frozen water samples as previously explained in Supplementary Data 8 and its supplements (Hendriksen et al, 2019). Anne Seyfarth was responsible for gathering and compiling the submitted data and match it to the incomming samples. Christina Aaby Svendsen was responsible for DNA extraction and Patrick Munk was responsible for organizing DNA external DNA sequencing and gathering the in silico data.
Timing and spatial scale	In addition to the 2016 pilot study, bi-annual sampling campaigns in both Winter (November) and Summer (June samples), were done to avoid seasonal biases based on Earth's hemispheres. Longitudinal sampling campaigns in select cities across all inhabited continents were also conducted. All the new non-pilot samples were collected between January 1st of 2017 and May 22th, 2019.<br><br>The spatial scope of the study was meant to exceed earlier AMR monitoring efforts and provide a unique dataset that could both be used for AMR surveillance, but also for other pathogen surveillance. The exact studied samples were convenience samples and the scope could not be pre-determined. We aimed to include all countries in the world to best map out the problem of AMR, but were ultimately limited by our abilities to recruit inside and outside of our network.

	We aimed to sample biannually avoid the bias that might be associated with always sampling one hemisphere in winter and another in summer. The resulting exact frequency was however also influenced by the consortium's busy and changing schedules during the COVID pandemic. In 2016 during the pilot study, we only had a single June sampling campaign, and there is thus a longer stretch with lower coverage before the June 2017 sampling campaign.
Data exclusions	Data exclusion criteria were not established prior to the study. The samples and datasets that were excluded from the previous study with 2016 samples were still excluded. Based on metadata and followup discussions, a number of samples received from participants were discovered to not represent whole-city sewage. These include sewage samples from schools, farms, hospitals and hotels.
Reproducibility	As its often the case in metagenomics studies, no attempts at classical replication were carried out. The pilot study (Hendriksen et al, 2019, Nat. Communications) included reproducibility analysis on a number of samples taken 1 day apart at the same site (supplementary figure 2), which were used to validate our study design. Technical replicate in the form of re-sequencing the same library multiple times was also done.<br><br>The workflow with an identical DNA extraction, sequencing strategy and ResFinder-based quantification has also been performed with actual replicates in pig farm feces, where replicated samples cluster with the highest similarity (Munk et al., 2018, Nature Microbiology).<br><br>Parts of this study could be considered a replication of the 2016 sampling campaign (Hendriksen et al., 2019, Nature communications) that found the same regional resistome cluster patterns that we see in the subsequent years. We have thus managed to replicate all the pilot study findings we attempted to reproduce. One might also consider each sampling round or year successful replication attempts.
Randomization	No treatment groups as such were used, so randomization was not needed. Samples were organized based on World Bank regions for illustrative purposes and calculating regional effects.
Blinding	This study is observational, rather than experimental. No attempts of blinding individual persons were done. The software analyzing the genomic sequences were "blind" to the metadata associated with each sample.

Did the study involve field work? ☐ Yes ☒ No

Reporting for specific materials, systems and methods

We require information from authors about some types of materials, experimental systems and methods used in many studies. Here, indicate whether each material, system or method listed is relevant to your study. If you are not sure if a list item applies to your research, read the appropriate section before selecting a response.

Materials & experimental systems

n/a	Involved in the study
☒	☐ Antibodies
☒	☐ Eukaryotic cell lines
☒	☐ Palaeontology and archaeology
☒	☐ Animals and other organisms
☒	☐ Human research participants
☒	☐ Clinical data
☒	☐ Dual use research of concern

Methods

n/a	Involved in the study
☒	☐ ChIP-seq
☒	☐ Flow cytometry
☒	☐ MRI-based neuroimaging
